# Treatment with albumin-hydroxyoleic acid complex restores sensorimotor function in rats with spinal cord injury: Efficacy and gene expression regulation

**DOI:** 10.1371/journal.pone.0189151

**Published:** 2017-12-15

**Authors:** Gerardo Avila-Martin, Manuel Mata-Roig, Iriana Galán-Arriero, Julian S. Taylor, Xavier Busquets, Pablo V. Escribá

**Affiliations:** 1 Hospital Nacional de Parapléjicos, Toledo, Spain; 2 Department of Pathology, University of Valencia, Valencia, Spain; 3 Stoke Mandeville Spinal Research, National Spinal Injuries Centre, Buckinghamshire Healthcare Trust, NHS, Aylesbury, United Kingdom; 4 Harris Manchester College, University of Oxford, Oxford, United Kingdom; 5 Laboratory of Molecular Cell Biomedicine, University of the Balearic Islands, Palma de Mallorca, Spain; Stony Brook University, UNITED STATES

## Abstract

Sensorimotor dysfunction following incomplete spinal cord injury (SCI) is often characterized by paralysis, spasticity and pain. Previously, we showed that intrathecal (i.t.) administration of the albumin-oleic acid (A-OA) complex in rats with SCI produced partial improvement of these symptoms and that oral 2-hydroxyoleic acid (HOA, a non-hydrolyzable OA analogue), was efficacious in the modulation and treatment of nociception and pain-related anxiety, respectively. Here we observed that intrathecal treatment with the complex albumin-HOA (A-HOA) every 3 days following T9 spinal contusion injury improved locomotor function assessed with the Rotarod and inhibited TA noxious reflex activity in Wistar rats. To investigate the mechanism of action of A-HOA, microarray analysis was carried out in the spinal cord lesion area. Representative genes involved in pain and neuroregeneration were selected to validate the changes observed in the microarray analysis by quantitative real-time RT-PCR. Comparison of the expression between healthy rats, SCI rats, and SCI treated with A-HOA rats revealed relevant changes in the expression of genes associated with neuronal morphogenesis and growth, neuronal survival, pain and inflammation. Thus, treatment with A-HOA not only induced a significant overexpression of *growth and differentiation factor 10 (GDF10)*, *tenascin C (TNC)*, *aspirin (ASPN) and sushi-repeat-containing X-linked 2 (SRPX2)*, but also a significant reduction in the expression of *prostaglandin E synthase (PTGES) and phospholipases A1 and A2 (PLA1/2)*. Currently, SCI has very important unmet clinical needs. A-HOA downregulated genes involved with inflammation and upregulated genes involved in neuronal growth, and may serve to promote recovery of function after experimental SCI.

## Introduction

Spinal cord injury (SCI) leads to multiple cellular and molecular alterations each following a broad spatiotemporal pattern [1–3]. Although mechanical injury to the spinal cord causes immediate damage to neurons, several pathophysiological changes are induced following the initial acute phase. Mechanical spinal injury also leads to disrupted blood flow associated with bleeding within the immediate vicinity of the injury and ischemia [4], with release of free radicals and toxicity induced by hemoglobin [5]. Acute SCI also involves activation of microglia and astrocytes, and immune cells such as neutrophils (6–24 h), macrophages (24 h to 2 weeks) and T cells [6]. The ensuing neuroimmune response present during the primary and secondary SCI processes, which includes both pro-inflammatory and anti-inflammatory processes, is a relevant component of SCI pathophysiology [7,8] Balanced activity of inflammatory cell types, such as microglia and macrophages, have been shown to improve morphological and functional parameters of SCI [9]. Indeed, microglia and macrophages can change from pro-inflammatory, cytotoxic phenotypes to anti-inflammatory, pro-repair cells types [10], mediated for example by interleukin-4 that facilitates microglia and macrophages to a pro-inflammatory state after SCI [11]. Sometimes, inflamory response improves the regeneration after spinal cord injury. Intraspinal application of diferent proinflammatory drugs, potenciate axonal regeneration [12, 13]. Microglia/macrophages in the injured spinal cord show a M1-like activation state facilitating the proinflammatory state [14].

Comprehensive characterization of the cellular processes activated after SCI and their modification by new therapeutic potential agents, that may ameliorate secondary damage and promote adaptive sensorimotor neuroplasticity, can be achieved using differential gene expression analysis using microarray technology (DNA chips) [15–17]. These studies examine gene expression changes from pooled RNA samples from animals with SCI [18–21] and contribute to our understanding of SCI pathophysiology, including initial upregulation of transcription factors and pro-inflammatory genes, and downregulation of some structural proteins, neurotransmitter receptors and transporters [3].

SCI involves several changes in sensorimotor function below the injury level, including varying degrees of paralysis, and the development of debilitating symptoms and spasticity [22–26]. In addition, spinal injury can cause changes in pain processing, some of which are generated by local pathophysiological mechanisms [27–30]. Taken together these symptoms interfere with successful rehabilitation of residual voluntary motor function following incomplete spinal cord injury [31] and lead to lower quality of life [25, 27–32]. Due to the multiple spinal pathophysiological mechanisms triggered by SCI, novel treatments should be designed to control neuroinflammation and promote growth of residual descending control systems across the lesion [33–38]. In this context, some symptoms of sensorimotor dysfunction following SCI have been related to glial reactivity at the injury site [39, 40], while the restoration of constitutive serotonin and noradrenaline receptors has been reported to be essential for restoring residual motor function [41–43]. Recently, we reported partial recovery of sensorimotor function following T9 contusion SCI in the rat after intrathecal treatment with albumin and ω-9 oleic acid (A-OA) [24]. Immunohistochemical analysis of the lumbar spinal cord revealed that A-OA treatment strongly increased lumbar serotoninergic innervation, and reduced microglia activation and glutamate receptor phosphorylation [24]. Intrathecal injections of A-OA also reduce lesion-induced PPARα immunoreactivity in glia cells [44, 45]. In this context, the modified ω-9 fatty acid molecule, 2-hydroxy OA (HOA), undergoes a slower metabolization compared to OA, due to the fact that hydroxylation of the alpha carbon impairs its degradation through the beta-oxidation pathway [46–47]. Furthermore, oral administration of HOA demonstrated safety and efficacy in the control of cell proliferation and blood pressure in models of cancer and hypertension, respectively [48,49]. Moreover, oral HOA administration inhibits mechanical and thermal hypersensitivity accompanied by a reduction of microglia reactivity in lumbar spinal dorsal horn following peripheral nerve injury [50].

In the present study, the effect of intrathecal administration of A-HOA on residual lower limb motor function and TA noxious reflex activity up to 28 days following T9 contusion SCI is described. Moreover, injured spinal cord tissue gene expression was analysed using DNA microarray analysis confirmed by RT-PCR analysis in A-HOA and saline-treated treated *Wistar* male rats 1 and 7 days after SCI. This novel treatment induced a marked recovery of the sensorymotor function and pain reduction in rats with SCI. In connection with these effects, we observed downregulation of neuroinflammation-related genes and upregulation of growth factors involved in neurogenesis, among other changes induced by A-HOA treatment. The present study demonstrates that the synthetic lipid HOA is a promising candidate to cover unmet clinical needs of patients with SCI.

## Methods

Ten week old male *Wistar* rats (*HsdHan®*:*WIST*, Harlan Laboratories, 250–300 g) with free access to food and water were used. Animals were randomly assigned to different groups following SCI, each of which was administered with an intrathecal bolus. The following 5 experimental groups were planned for microarrays determinations: Control Group without lesion (n = 5), T9 vertebral region (T8 medullar) moderate contusion group treated 1 day or 7 days with saline vehicle (intrathecal, i.t., n = 5), T9 moderate contusion group treated 1 day or 7 days with an A-HOA bolus for 1 day or 7 days (80:0.4 nanomole of HOA and Albumin, respectively, i.t., n = 5). The compounds were administered by local injection in a volume of 10 μl [72] as previously described, immediately following the SCI and every 3 days. For behavioral and electrophysiological reflex analysis, animals were treated during 28 days (10 μl every 3 days, i.t.) as described below [24].

All experimental procedures were approved by the institutional animal experimentation ethical committee [National Hospital for Paraplegic Animal Experimentation Ethical Committee (Register n° V-45-168-296)]. The experiments adhered to the guidelines of the Committee for Research and Ethical Issues of IASP published in PAIN 1983; 16:109–110.

### Preparation of the A-HOA complex

The complex was prepared with 20% human albumin (Grifols®), by adding HOA (kindly donated by Lipopharma Therapeutics S.L.). 2-Hidroxyoleic Acid/Albumin solution was diluted to a concentration of 80:0.4 nanomoles in saline (0.9%), as previously described [24].

### Experimental animal surgery

Rats were anesthetized with pentobarbital (i.p., 65 mg/kg) and xylazine (i.p., 10 mg/kg). Approximately 90 minutes later, during the experimental surgery process, they received an additional dose containing of 20 mg/kg pentobarbital and 3 mg/kg xylazine. In addition, 0.1 ml of antibiotic was administered (2.5% Baytril, Enrofloxacin, Bayer) after surgery, followed by daily doses during 3 days after SCI.

Commercially available rat intrathecal catheters (ALZT7740Z, Charles River Laboratories, Spain) were implanted (see below) and externalized accordingly [84]. Immediately before surgical implantation, the catheter was re-sterilized with absolute ethanol, and thoroughly washed with sterile 0.9% saline. Following skin incision and blunt dissection of the muscle layers overlying the vertebrae, a small hemi-laminectomy at the vertebral T10 level was performed. The exposed dura-mater was subjected to a small durectomy with iris-type scissors so that the tip of the i.t. catheter could be inserted rostrally and medially on top of the spinal cord with a final position just below the intended T9 contusion site. The area was cleaned to permit catheter fixture with acrylate cement to the T11 vertebrae. The percutaneous end of the i.t. catheter was finally secured by inserting it through a small cutaneous incision at the base of the cranium, whereupon it was filled with 0.9% sterile saline and tapped with a custom-made nylon filament.

Following intrathecal catheter implantation, a spinal T9 contusion was performed [85]. A bilateral T9 vertebral laminectomy enabled spinal contusion by allowing an 11-gram weight to fall from a height of 12 mm onto a cylindrical flat-tipped impactor with a 2.5 mm diameter placed centrally over the exposed spinal cord above the intact dura. Once the contusion was performed, artificial dura mater was placed onver the injury area (Neuropatch, B. Braun) and the overlying muscle layers were reapposed with a continuous suture stitch and the skin was finally closed with a subdermal suture, both with a 4–0 reabsorbable thread. Rats were carefully observed during recovery, and the bladder was manually expressed daily until recovery of function.

### Tissue collection

Tissue was extracted at two specific time points after SCI: at 1 and 7 days after injury. Animals were deeply anesthetized with pentobarbital (Dolethal, 65 mg/kg, i.p., Ref: 737). Dorsal laminectomy was performed to extract thoracic spinal tissue (T7-T9). Spinal tissue was first disected and placed on a petri-dish on dry ice and median sagittally sectioned with a scalpel blade. The spinal tissue was placed in a 2-ml cryotube (479–0821, VWR International Eurolab SL, Spain) whereupon the sample was homogenized with the aid of a scalpel in 0.5ml of TriZol® Reagent (15596–026, Invitrogen SA, Spain), and then rapidly frozen in liquid nitrogen. The total tissue collection time was no longer than 10 minutes. All the spinal tissue was stored at -80°C until use.

### DNA microarray analyses (Affymetrix, rat genome 230 2.0 arrays)

DNA microarray analyses were performed as described [86]. First, RNA was extracted from each cord sample individually using TriZol® Reagent (Invitrogen, Spain) as described [87]. Spinal cord samples from the contusion area were collected 1 day or 7 days after contusion in animals that had been submitted to treatment with saline vehicle (SCI controls, n = 4) or A-HOA (n = 4) as indicated above. The same type of sample (spinal cord area and amount of tissue) was collected from healthy rats (healthy controls, n = 4). One hundred nanograms of total RNA was used to synthesize double stranded cDNA by reverse transcription and subsequently, biotinylated cRNA was transcribed in vitro and it was fragmented as detailed by the manufacturer (Affymetrix, CA, U.S.A.).

Global RNA analysis profiles were studied using Affymetrix rat genome 230 2.0 arrays (Affymetrix, CA, USA) as previously described [86]. Total RNA was extracted from each cord sample individually using TriZol® Reagent (Invitrogen, Spain), as described [87]. Spinal cord samples from the contusion area were collected 1 day or 7 days after contusion in animals that had been submitted to treatment with saline vehicle (SCI controls, n = 4) or A-HOA (n = 4) as indicated above. The same type of sample (spinal cord area and amount of tissue) was collected from healthy rats (healthy controls, n = 4). Amplification, labeling, hybridization, staining, washing, and scanning of the microarrays followed standardized protocols, with manufacturer-recommended reagents and instruments.

DNA Chip Analysis Software (Cheng Li Laboratory, Department of Biostatistics, Harvard University, Boston, MA, USA) was used to analyze the data. The CEL files were normalized by the invariant sets method [88, 89], and model-based expression values were obtained using the perfect match/mismatch difference model. Images were inspected for imperfections, and the quality of the data was verified with the outlier detection algorithm as described [88].

Analysis of variance (ANOVA) was used to test for significant differences between experimental groups. The False Discovery Rate tool included in dCHIP was used to detect false positives. Significant changes were identified using the following filtering criteria: statistical significance of p<0.05, of which those with ≥ and 4-fold change (absolute value) were selected for further analysis; differences of intensities over 100 between baseline and experimental means; detection call of “Present” in the experimental group. Only those genes whose expression met all these criteria were considered regulated with respect to their corresponding group. Non-agglomerative two-dimensional hierarchical clustering was used to analyze the data expression profiles. The Euclidean distance was used to generate clusters, and probe sets were grouped according to similar expression values.

### RT-PCR analyses

For the present study, additional real-time RT-PCR was performed to validate additional genes from several major functional classes altered by injury. The same animal samples and RNA extractions used for microarray analyses were used for RT-PCR. RT-PCR was performed for the following genes: *PTGES*, *PLA2GA2*, *PLA1A*, *GDF10*, *TNC*, *ASPN*, *TIMP1*, *FABP4*, *LCN2*, *IL1B*, *EMR1*, *PLTP*, *MOBP*, *COMT*, *CRYAB*, *ARSB*, *NAAA*, *PTPRC*, *AXL and PTAFR*. cDNA synthesis was performed using 100 ng total RNA and the TaqMan Reverse Transcription Reagents kit (Applied Biosystems, Carlsbad, CA, USA). Real-time PCR was carried out in a 7900 HT thermocycler (Applied Biosystems) using 2× Gene Expression Master Mix and Assays on Demand (Applied Biosystems). For comparative analysis, the 2^-δδCt^ method was used [86].

### Motor activity determination

Voluntary hindlimb motor function before and after T9 contusion injury was analyzed in all experimental groups using a Rota-Rod device (4600, Ugo Basile), similarly as described [24]. Briefly, prior to contusion injury, each animal was trained for three days to remain upon a cylindrical surface which rotated at 5 rpm for at least 5 minutes. On the day before SCI control data were obtained by subjecting the rats to the Rota-Rod test, but with the cylinder rotating at a steadily accelerating speed from 5 to 15 rpm during the 5 minutes test duration. Following SCI, rats were tested on day 4 and then weekly thereafter up to 28 days with the Rotarod cylinder rotating at a steadily accelerating speed from 5 to 15 rpm during the 5 minutes test duration, to follow general voluntary motor recovery and the effect of the different treatments strategies.

### Tibialis anterior noxious reflex

The methodological protocol for the measurement of TA noxious reflex activity has been described [24]. Briefly, four weeks after spinal cord injury, the rats were anesthetised with isoflurane (2%) in medicinal air (17% oxygen, at 2 l/min, Synthetic medical air, Carburos Metalicos, Spain). The nose was then inserted into a plexiglass adapter (Cibertec S.A., Spain) to administer the isoflurane-air mixture, and atropine was subcutaneously administered. The animal was placed in a supine position on an electric blanket maintained at 37°C (RTC1 Thermal Regulator, Cibertec S.A.). Hair over the left TA muscle and at the mid-thoracic level was removed and both the trunk and the hindlimbs were extended and fixed into a neutral position with adhesive tape. Bipolar electromyographic responses were recorded using two multi-stranded Teflon-coated steel electrodes (Cooner Wire, USA) subcutaneaously inserted ca. 0.5 cm into the belly of the Tibialis anterior (TA) muscle of the left limb. In addition, two platinum subdermal electrodes (Astro-Med Inc., Grass Instruments, USA) were inserted into the tip of the fourth toe and secured with adhesive tape. Finally, an earth electrode was inserted subcutaneously between the stimulation electrode and the recording electrode at the level of the left ankle. Prior to beginning reflex EMG measurements, the isoflurane anesthesia level was lowered to 1.2% MAC in medicinal air (1 l/min). Reflex threshold was identified by characterizing the minimal current intensity (mA) required to evoke a clear nociceptive TA reflex EMG response between 0.2 and 1.0 s after stimulation, in over half of ten stimuli. Nociceptive TA reflex activity and temporal summation was evoked during a train of 16 stimuli applied at 1 Hz. Electromyographic data were integrated using the modulus function of the analysis software (Spike 2, CED, UK) between 0.2 and 0.6 s after the stimulus. Integrated reflex EMG data were analyzed after each stimulus and normalised as a percentage of the first reflex response.

## Results

### A-HOA promotes sensorimotor function recovery in rats with SCI

**Fig 1:**
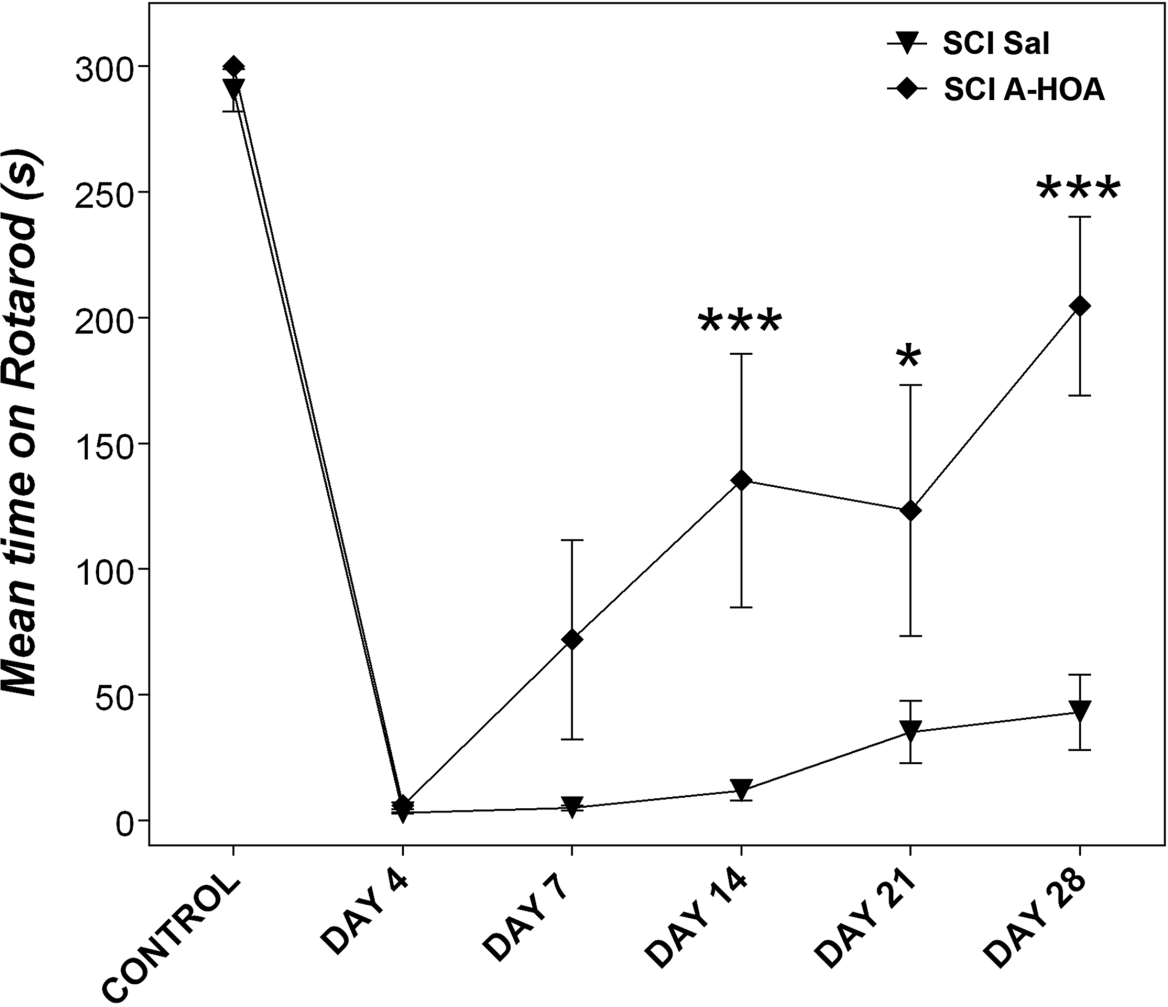
A-HOA promotes early recovery of motor function following T9 spinal cord injury. Longitudinal analysis of the mean (±SEM) time spent on the rotarod following contusion SCI from 4 to 28 days revealed that intrathecal administration of A-HOA (SCI A-HOA, ♦) induced locomotor recovery in contrast to saline vehicle treatment (SCI Sal, ▼). Statistical analysis was performed using a two-way ANOVA. *p<0.05; ***p<0.001. For further details see the materials and methods section.

Four days after T9 contusion SCI in animals treated with saline, motor function (as assessed on the rotarod) was reduced to 1.1±0.1% compared with the pre-lesion control value (100±3%, Fig 1). The experimental SCI group treated with A-HOA also showed similar reduction in the motor activity during the first days after lesion. However, animals treated with A-HOA showed a marked and significant increase in the rate and extent of recovery of voluntary motor function (p<0.01, Fig 1). Thus, A-HOA induced a recovery of ca. 70% in motor function after 28 days of treatment. In contrast, rats treated with saline only showed use of the rotarod to below 10% (Fig 1).

### Inhibition of noxious TA reflex activity with A-HOA treatment after SCI

**Fig 2:**
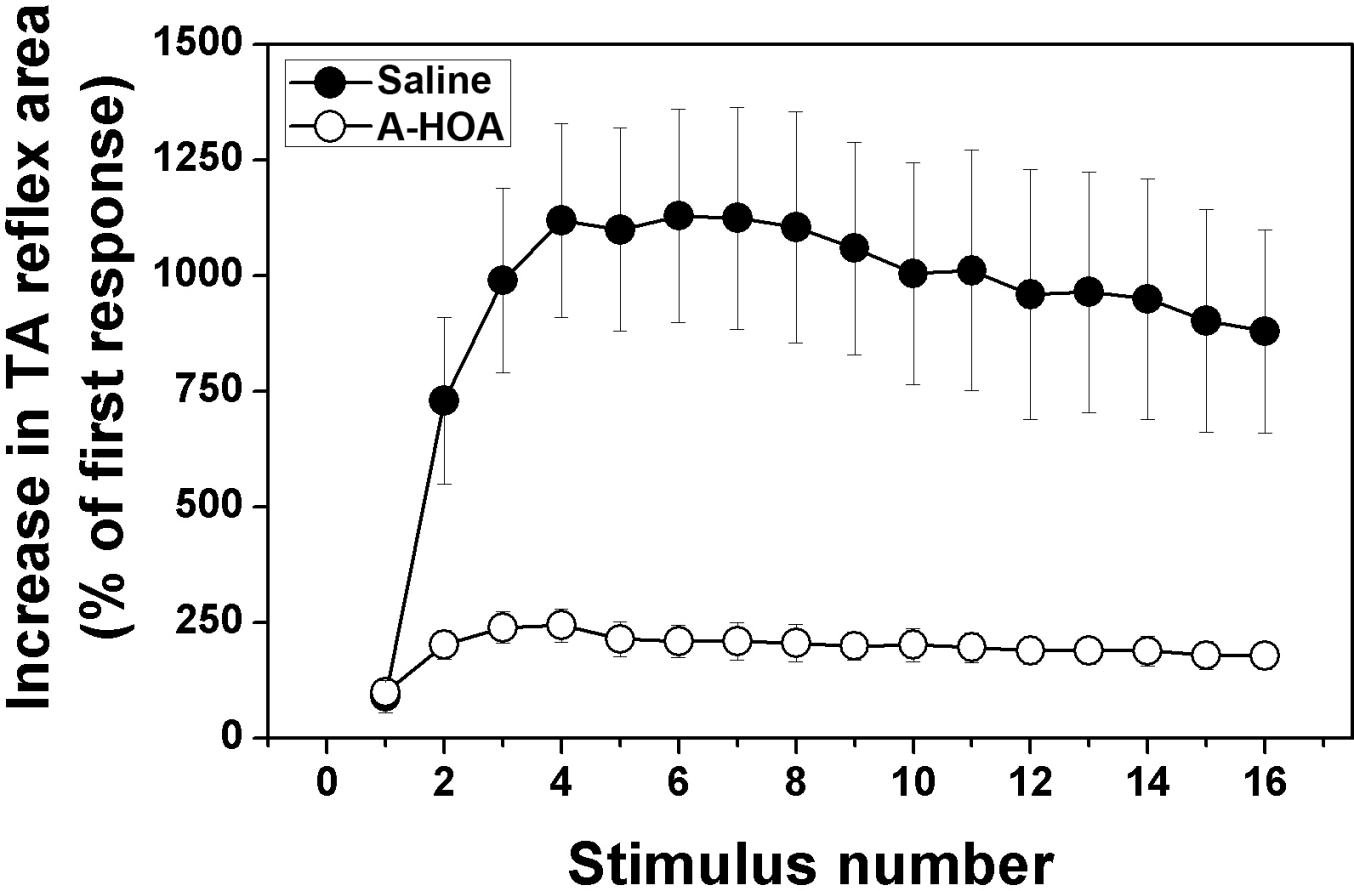
Inhibition of Tibialis Anterior noxious reflex activity in rats with SCI. Quantitative analysis of Tibialis Anterior (TA) noxious reflex temporal summation at 28 days following spinal contusion. Significant (p<0.001) inhibition of noxious TA temporal summation in animals with contusion SCI was observed after A-HOA treatment when compared with the group treated with saline vehicle. For further details see the materials and methods section.

TA reflex EMG activity, recorded in response to noxious electrical stimuli, was present in animals with experimental T9 contusion SCI treated with saline vehicle (Fig 2). In animals with SCI treated with saline vehicle alone, the temporal summation of the nociceptive TA flexor reflex was observed up to a maximal value of 1150±200% when compared to the first reflex response (Fig 2). A-HOA had a strong inhibitory effect on temporal summation (Fig 2); thus, post-hoc analysis revealed that temporal summation of the TA nociceptive reflex was inhibited in rats with SCI following treatment with A-HOA. In these A-HOA-treated animals, the maximal TA temporal summation observed was 210±30%.

### Gene expression analysis in the spinal contusion area in rats with SCI

Whole-genome expression analysis was performed independently on 4 animal samples (spinal cord T8-T10 contusion area) from each group: control, SCI after 1 day, SCI after 7 days, SCI treated with A-HOA after 1 day, SCI treated with A-HOA after 7 days.

**Fig 3:**
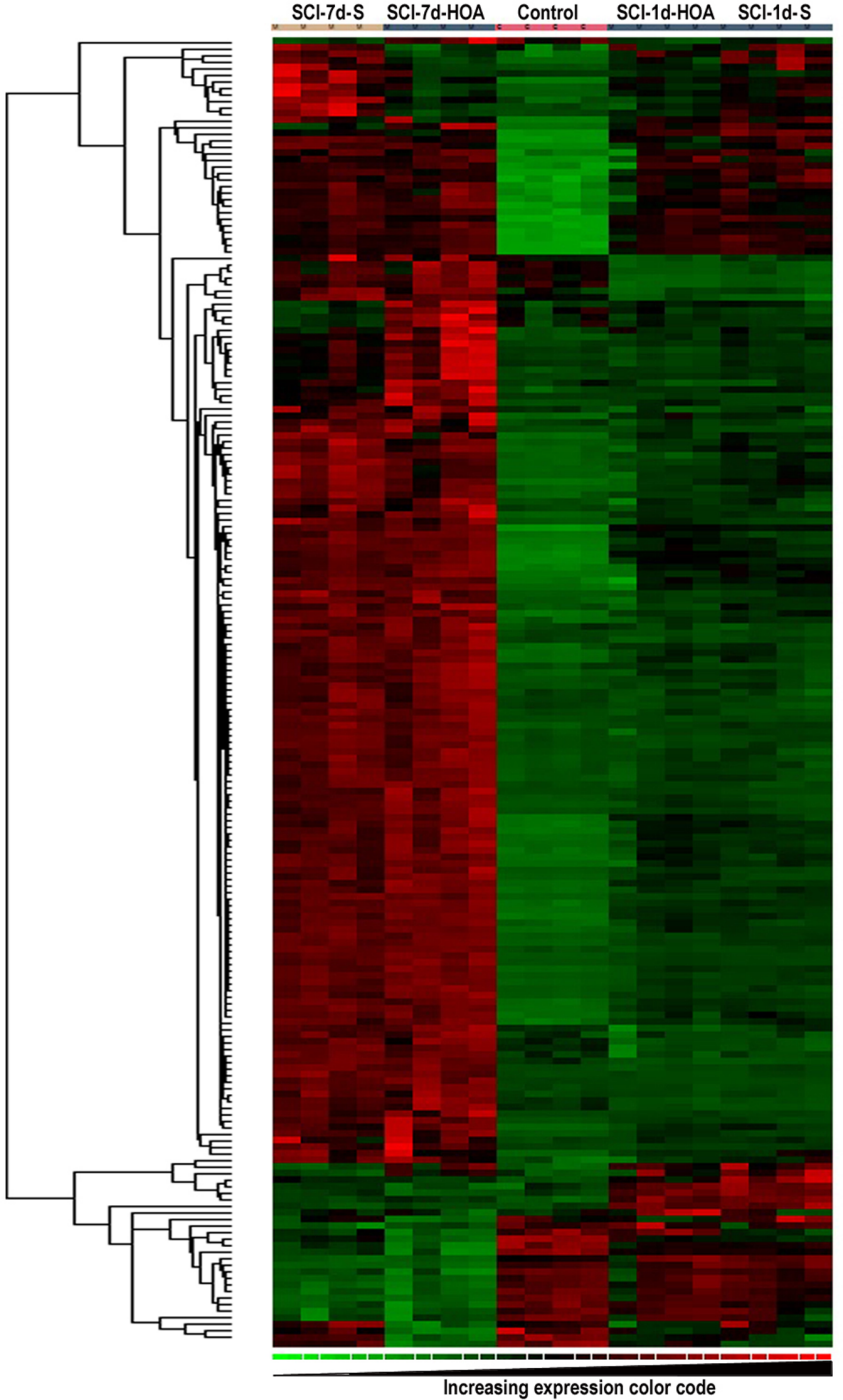
mRNAs differentially expressed in the spinal cord lesion region in rats with SCI. After contusion, total RNA was extracted from the lesion region of rats with SCI or from healthy non-injured controls (Control) 1 day or 7 days (1d and 7d, respectively) and treated with saline vehicle (S) or A-HOA (HOA). mRNA was quantified by microarray analysis. ANOVA following the false discovery rate (FDR) P value correction used to detect significant changes. The figure shows hierarchical clustering in the 5 experimental groups showing the expression levels from green (low) to red (high). Expression levels using the color code indicated at the bottom of the graph is shown for all four animals from each experimental group.

Upon application of the quantification criteria detailed above, DNA microarray analysis revealed marked differences in the gene expression pattern between healthy non-injured rats and those with SCI in the T9 area of the spinal cord both after 1 and 7 days post-lesion (Fig 3). In contrast, rats with SCI and treated with saline showed differences with respect to those that received A-HOA treatment both at 1 and 7 days after lesion (Fig 3). In this context, SCI induced changes in the expression of a very high number of genes (S1 Table). Moreover, ca. 600 genes showed an expression altered over 4-fold with respect to healthy rats (Tables 1 and 2). Interestingly, only 43 genes showed an expression 4-fold lower than healthy controls (Table 2) whereas ca. 550 genes appeared to be overexpressed (Table 1) 1 week after the lesion.

**Table 1:** Overexpressed genes in the lesion of rats with SCI (7 days post trauma) compared with non-injured rats.

mRNA species	Fold Change	p
*secretory leukocyte peptidase inhibitor*	255.25	0.013924
*lipocalin 2*	177.51	0.005869
*CD8a molecule*	149.04	0.002310
*chemokine (C-X-C motif) ligand 1 (melanoma growth stimulating*, *alpha)*	101.89	0.018744
*chemokine (C-X-C motif) ligand 13*	73.05	0.005268
*Similar to Serum amyloid A-3 protein precursor*	67.32	0.033560
*Fc receptor-like S*, *scavenger receptor*	57.85	0.000117
*Cd68 molecule*	54.98	0.000051
*chemokine (C-C motif) ligand 2*	51.22	0.016908
*leukocyte immunoglobulin-like receptor*, *subfamily B*, *member 4*	49.05	0.006810
*apolipoprotein B mRNA editing enzyme*, *catalytic polypept 1*	46.37	0.00006
*phospholipase A2*, *group IIA (platelets*, *synovial fluid)*	42.60	0.024865
*interferon induced transmembrane protein 1*	42.34	0.000046
*chemokine (C-C motif) ligand 20*	40.94	0.036242
*killer cell lectin-like receptor*, *subfamily A*, *member 5 /// Ly49 stimulatory receptor 7*	38.31	0.000047
*chemokine (C-X-C motif) ligand 11*	38.23	0.005826
*interleukin 1 beta*	36.87	0.003612
*C-type lectin domain family 7*, *member a*	33.76	0.000075
*killer cell lectin-like receptor*, *subfamily A*, *member 5*	31.27	0.000007
*CD8a molecule*	30.33	0.005512
*lipopolysaccharide binding protein*	28.75	0.001551
*Rn*.*82246*.*1*	28.44	0.001277
*cytochrome P450*, *family 2*, *subfamily d*, *polypeptide 1 /// cytochrome P450*, *family 2*, *subfamily d*, *polypeptide 5*	28.28	0.019884
*folate receptor 2 (fetal)*	27.34	0.001246
*chemokine (C-C motif) ligand 9*	25.80	0.011754
*chemokine (C-C motif) ligand 3*	25.79	0.000761
*immunoglobulin superfamily*, *member 6*	25.78	0.001191
*interferon activated gene 204*	25.64	0.020116
*chemokine (C-C motif) ligand 7*	25.41	0.049355
*centromere protein F*	25.00	0.002765
*ribonucleotide reductase M2*	24.37	0.013762
*CDC28 protein kinase regulatory subunit 2*	24.14	0.002132
*tumor necrosis factor receptor superfamily*, *member 1b*	23.86	0.000471
*leukocyte immunoglobulin-like receptor*, *subfamily B (with TM and ITIM domains)*, *member 3*	23.34	0.010143
*C-type lectin domain family 4*, *member a3*	23.25	0.000736
*RT1 class I*, *locus CE5*	23.24	0.023436
*epithelial cell transforming sequence 2 oncogene*	23.16	0.003377
*Rn*.*17927*.*1*	22.62	0.010525
*C-type lectin domain family 12*, *member A*	22.59	0.001627
*ribonucleotide reductase M2*	22.58	0.001574
*Rn*.*25444*.*1*	22.56	0.005194
*ubiquitin-conjugating enzyme E2C*	21.97	0.002465
*kinesin family member 20A*	21.72	0.001638
*Rn*.*11988*.*1*	21.46	0.000510
*DEP domain containing 1*	21.28	0.004470
*CD8b molecule*	21.04	0.000303
*Rn*.*43019*.*1*	20.53	0.012316
*Rn*.*23777*.*1*	19.72	0.001785
*cyclin-dependent kinase 1*	19.72	0.001136
*topoisomerase (DNA) II alpha*	19.48	0.001635
*kinesin family member 2C*	19.20	0.006305
*cathepsin C*	19.19	0.003236
*matrix metallopeptidase 9*	19.11	0.010366
*membrane-spanning 4-domains*, *subfamily A*, *member 7*	19.07	0.003380
*phospholipase B domain containing 1*	18.73	0.000655
*membrane-spanning 4-domains*, *subfamily A*, *member 6B*	18.62	0.002629
*cell division cycle associated 3*	18.58	0.003405
*complement component 1*, *q subcomponent*, *C chain*	18.18	0.000637
*complement factor properdin*	17.90	0.025026
*kininogen 1 /// kininogen 1-like 1 /// kininogen 2*	17.89	0.003902
*killer cell lectin-like receptor*, *subfamily A*, *member 17 /// immunoreceptor Ly49si3-like /// hypothetical protein LOC497796 /// similar to immunoreceptor Ly49si1 /// Ly49 inhibitory receptor 5 /// immunoreceptor Ly49si1 /// immunoreceptor Ly49si2 /// immunoreceptor Ly49si3 /// similar to immunoreceptor Ly49si3*	17.72	0.006798
*Rn*.*43961*.*1*	17.69	0.003668
*chemokine (C-X-C motif) ligand 2*	17.60	0.018680
*toll-like receptor 2*	17.42	0.000143
*complement component 2*	17.23	0.005947
*chemokine (C-X-C motif) ligand 9*	17.15	0.010375
*regulator of G-protein signaling 1*	17.11	0.000034
*cystatin F (leukocystatin)*	17.02	0.010998
*interleukin 6*	16.89	0.005156
*complement factor D (adipsin)*	16.74	0.003805
*interleukin 2 receptor*, *gamma*	16.46	0.001355
*guanine nucleotide binding protein (G protein)*, *gamma transducing activity polypeptide 2*	16.19	0.000590
*syndecan 1*	15.98	0.003205
*nucleolar and spindle associated protein 1*	15.87	0.009738
*similar to paired immunoglobin-like type 2 receptor beta /// similar to cell surface receptor FDFACT*	15.86	0.002416
*Rn*.*46917*.*1*	15.81	0.000073
*plasminogen activator*, *urokinase*	15.76	0.002905
*SLAM family member 9*	15.72	0.000256
*ATP-binding cassette*, *sub-family A (ABC1)*, *member 1*	15.50	0.000880
*topoisomerase (DNA) II alpha*	15.43	0.000894
*carcinoembryonic antigen-related cell adhesion molecule 1 (biliary glycoprotein) /// carcinoembryonic antigen-related cell adhesion molecule 10*	15.19	0.016720
*Rn*.*13512*.*1*	14.98	0.001033
*EGF-like module containing*, *mucin-like*, *hormone receptor-like 1*	14.90	0.000140
*budding uninhibited by benzimidazoles 1 homolog*, *beta (S*. *cerevisiae)*	14.62	0.004846
*NS5A (hepatitis C virus) transactivated protein 9*	14.39	0.004346
*Rn*.*43624*.*1*	14.31	0.029857
*stabilin 1*	14.25	0.003902
*ATP-binding cassette*, *sub-family A (ABC1)*, *member 1*	14.23	0.000984
*family with sequence similarity 64*, *member A*	14.17	0.001604
*periostin*, *osteoblast specific factor*	13.96	0.023501
*hypothetical protein LOC689399*	13.87	0.004450
*membrane-spanning 4-domains*, *subfamily A*, *member 11*	13.87	0.001427
*cyclin A2*	13.78	0.006265
*myosin IF*	13.57	0.001748
*Rn*.*19507*.*1*	13.56	0.000119
*activating transcription factor 3*	13.34	0.000099
*complement component 2*	13.33	0.001978
*CD14 molecule*	13.32	0.011782
*protein lyl-1-like // lymphoblastic leukemia derived sequence 1*	13.19	0.000007
*hypothetical LOC298077*	13.14	0.012059
*complement component 1*, *q subcomponent*, *B chain*	13.05	0.007157
*family with sequence similarity 111*, *member A*	12.95	0.018706
*Rn*.*41848*.*1*	12.79	0.003937
*acid phosphatase 5*, *tartrate resistant*	12.78	0.000007
*T-cell receptor beta chain*	12.74	0.013690
*B-cell leukemia/lymphoma 2 related protein A1d*	12.73	0.000147
*syndecan 1*	12.72	0.001009
*CCAAT/enhancer binding protein (C/EBP)*, *delta*	12.70	0.008457
*CD36 molecule (thrombospondin receptor)*	12.62	0.005720
*desmocollin 2*	12.48	0.007327
*Rn*.*15505*.*1*	12.16	0.005645
*cyclin B1*	12.07	0.002048
*Rn*.*8244*.*1*	12.02	0.001165
*stefin A2-like 3*	11.98	0.036969
*signal transducing adaptor family member 1*	11.96	0.004210
*complement component 2*	11.91	0.001246
*S100 calcium binding protein A11 (calizzarin)*	11.88	0.007999
*neutrophil cytosolic factor 4*	11.86	0.002619
*paired immunoglobin-like type 2 receptor alpha*	11.81	0.003926
*protein regulator of cytokinesis 1*	11.80	0.000531
*mesothelin*	11.76	0.003398
*ADP-ribosylation factor-like 5C*	11.73	0.002995
*metallothionein 1a*	11.72	0.000003
*DnaJ (Hsp40) homolog*, *subfamily C*, *member 22*	11.60	0.007681
*DEAD (Asp-Glu-Ala-Asp) box polypeptide 60*	11.47	0.000193
*chemokine (C-C motif) ligand 4*	11.20	0.001643
*phospholipase A1 member A*	11.18	0.004211
*prostaglandin E synthase*	11.16	0.014772
*schlafen 3*	11.10	0.012101
*protein tyrosine phosphatase*, *receptor type*, *C*	11.09	0.004743
*Rn*.*8136*.*1*	11.05	0.000548
*hematopoietic prostaglandin D synthase*	11.00	0.002281
*Rho GTPase activating protein 8*	10.99	0.029394
*cyclin B2*	10.90	0.002157
*platelet factor 4*	10.89	0.003908
*Rn*.*34220*.*1*	10.83	0.003238
*maternal embryonic leucine zipper kinase*	10.82	0.003993
*RNA binding motif protein 47*	10.82	0.004257
*hemopoietic cell kinase*	10.74	0.000585
*tumor necrosis factor receptor superfamily*, *member 14 (herpesvirus entry mediator)*	10.73	0.005281
*chemokine (C-C motif) ligand 6*	10.67	0.000429
*ADP-ribosylation factor-like 11*	10.67	0.000120
*family with sequence similarity 105*, *member A*	10.65	0.000859
*nucleolar and spindle associated protein 1*	10.56	0.012334
*bone marrow stromal cell antigen 1*	10.55	0.002871
*v-maf musculoaponeurotic fibrosarcoma oncogene homolog B (avian)*	10.43	0.015517
*Rn*.*6731*.*1*	10.37	0.000049
*Fc fragment of IgG*, *low affinity IIa*, *receptor (CD32) /// Fc fragment of IgG*, *low affinity IIb*, *receptor (CD32)*	10.30	0.002587
*kinesin family member 18B /// kinesin-like protein KIF18B-like*	10.33	0.004672
*cytochrome P450*, *family 1*, *subfamily b*, *polypeptide 1*	10.28	0.004820
*leukocyte immunoglobulin-like receptor*, *subfamily B (with TM and ITIM domains)*, *member 3-like*	10.15	0.001043
*phospholipid scramblase 1*	10.12	0.002010
*placenta-specific 8*	10.09	0.000391
*Rn*.*17891*.*1*	10.08	0.000220
*triggering receptor expressed on myeloid cells 2*	10.07	0.000413
*chemokine (C-C motif) ligand 5*	10.05	0.008970
*matrix metallopeptidase 19*	10.03	0.000182
*tumor necrosis factor*, *alpha-induced protein 8-like 2*	9.96	0.002763
*Fc fragment of IgG*, *low affinity IIa*, *receptor (CD32) /// Fc gamma receptor II beta*	9.90	0.000597
*Cd69 molecule*	9.85	0.008057
*pituitary tumor-transforming 1*	9.80	0.001701
*cancer susceptibility candidate 5*	9.72	0.002957
*complement factor B*	9.58	0.001416
*Granulocyte-macrophage colony stimulating receptor alpha*	9.54	0.002564
*Fc fragment of IgG*, *low affinity IIIa*, *receptor*	9.43	0.003885
*collagen triple helix repeat containing 1*	9.34	0.028215
*tumor necrosis factor alpha induced protein 6*	9.28	0.016817
*neuralized homolog 3 (Drosophila)*	9.26	0.00192
*Rn*.*55535*.*1*	9.25	0.000028
*unc-93 homolog B1 (C*. *elegans)*	9.23	0.000179
*Rn*.*23529*.*1*	9.22	0.000635
*prostaglandin-endoperoxide synthase 2*	9.21	0.029610
*Rn*.*3724*.*1*	9.20	0.006151
*glucagon receptor*	9.20	0.021510
*GLI pathogenesis-related 1*	9.05	0.000001
*C-type (calcium dependent*, *carbohydrate recognition domain) lectin*, *superfamily member 6*	9.03	0.001373
*CCAAT/enhancer binding protein (C/EBP)*, *delta*	9.03	0.000440
*Rn*.*21147*.*1*	9.01	0.004375
*filamin binding LIM protein 1*	8.99	0.000696
*plasminogen activator*, *urokinase receptor*	8.98	0.001881
*hematopoietic cell signal transducer*	8.96	0.000691
*Rn*.*15077*.*1*	8.92	0.000090
*Rn*.*24230*.*1*	8.91	0.000671
*zinc finger CCCH type containing 12A*	8.90	0.000698
*solute carrier family 7 (cationic amino acid transporter*, *y+ system)*, *member 7*	8.87	0.000121
*schlafen 2*	8.84	0.000340
*coagulation factor V (proaccelerin*, *labile factor)*	8.83	0.022265
*mannose receptor*, *C type 1*	8.77	0.009812
*similar to paired immunoglobin-like type 2 receptor beta /// similar to cell surface receptor FDFACT*	8.77	0.004090
*nuclear antigen Sp100-like*	8.64	0.001008
*similar to hypothetical protein MGC34760*	8.58	0.006961
*retinol binding protein 1*, *cellular*	8.51	0.000292
*CD86 molecule*	8.49	0.000222
*stimulated by retinoic acid gene 6*	8.49	0.009655
*complement component 1*, *q subcomponent*, *A chain*	8.43	0.000065
*phospholipase D family*, *member 4*	8.42	0.005256
*TRAF-interacting protein with forkhead-associated domain*, *family member B*	8.39	0.010299
*interferon gamma inducible protein 30*	8.36	0.000000
*hypothetical LOC302884*	8.35	0.003418
*stimulated by retinoic acid gene 6*	8.34	0.011267
*pigeon homolog (Drosophila)*	8.31	0.002078
*Rn*.*37608*.*1*	8.24	0.001213
*strawberry notch homolog 2 (Drosophila)*	8.19	0.007075
*Rn*.*34740*.*1*	8.19	0.001824
*vav 1 guanine nucleotide exchange factor*	8.15	0.000017
*kinesin family member 23*	8.15	0.002259
*phosphorylase*, *glycogen*, *liver*	8.14	0.000040
*crystallin*, *mu*	8.09	0.005020
*Rn*.*63919*.*1*	8.04	0.001672
*Rn*.*16262*.*1*	8.02	0.000590
*triggering receptor expressed on myeloid cells 2*	8.02	0.000065
*RT1 class I*, *locus CE12*	8.01	0.022370
*similar to Shc SH2-domain binding protein 1*	7.98	0.005740
*Rn*.*79975*.*1*	7.92	0.000304
*Rn*.*14817*.*1*	7.92	0.017410
*leukocyte immunoglobulin-like receptor*, *subfamily B (with TM and ITIM domains)*, *member 3-like /// similar to paired-Ig-like receptor B /// similar to paired-Ig-like receptor A11*	7.92	0.017781
*carnosine dipeptidase 1 (metallopeptidase M20 family)*	7.90	0.000048
*cyclin-dependent kinase inhibitor 3*	7.89	0.000257
*Rn*.*22374*.*1*	7.88	0.000658
*chemokine (C-X-C motif) ligand 9*	7.88	0.024321
*toll-like receptor 7*	7.86	0.001658
*oxidized low density lipoprotein (lectin-like) receptor 1*	7.83	0.000996
*membrane-spanning 4-domains*, *subfamily A*, *member 11*	7.81	0.010059
*Rn*.*13339*.*1*	7.81	0.001245
*Rn*.*41691*.*1*	7.77	0.002347
*Rn*.*22530*.*1*	7.75	0.000064
*Rn*.*12095*.*1*	7.69	0.000684
*similar to RIKEN cDNA 1600029D21*	7.69	0.016664
*FYVE*, *RhoGEF and PH domain containing 2*	7.61	0.000224
*Solute carrier family 37 (glycerol-3-phosphate transporter)*, *member 2*	7.61	0.000005
*phospholipid transfer protein*	7.59	0.000159
*Rn*.*39365*.*1*	7.58	0.004913
*TRAF4 associated factor 1*	7.57	0.010165
*glutathione peroxidase 2*	7.57	0.007010
*proteasome (prosome*, *macropain) subunit*, *beta type 8 (large multifunctional peptidase 7)*	7.56	0.015366
*dipeptidase 2*	7.52	0.002268
*fermitin family homolog 3 (Drosophila)*	7.50	0.000056
*budding uninhibited by benzimidazoles 1 homolog (S*. *cerevisiae)*	7.47	0.002175
*stimulated by retinoic acid gene 6*	7.45	0.029079
*Rn*.*37608*.*2*	7.43	0.000278
*thyrotropin releasing hormone*	7.43	0.000139
*intercellular adhesion molecule 1*	7.40	0.000958
*suppression of tumorigenicity 14 (colon carcinoma)*	7.33	0.001224
*sterol O-acyltransferase 1*	7.32	0.002610
*mitogen-activated protein kinase kinase kinase 8*	7.18	0.001481
*guanylate binding protein 4*	7.15	0.000187
*Fc fragment of IgE*, *high affinity I*, *receptor for; α-polypeptide*	7.14	0.000300
*similar to Myeloid cell surface antigen CD33 precursor (Siglec-3)*	7.13	0.001428
*UDP-Gal*:*betaGlcNAc beta 1*,*4- galactosyltransferase*, *polypeptide 1*	7.13	0.000955
*CD36 molecule (thrombospondin receptor)*	7.03	0.003678
*Rn*.*12905*.*1*	7.02	0.000052
*5-hydroxytryptamine (serotonin) receptor 2B*	7.01	0.001990
*cytoskeleton associated protein 2*	6.97	0.000213
*Rn*.*20457*.*1*	6.95	0.003250
*family with sequence similarity 38*, *member A*	6.95	0.004140
*NCK associated protein 1 like*	6.94	0.000144
*solute carrier family 15*, *member 3*	6.93	0.002008
*docking protein 3*	6.90	0.000116
*N-acetylneuraminate pyruvate lyase*	6.86	0.000044
*coxsackie virus and adenovirus receptor*	6.85	0.011197
*Bruton agammaglobulinemia tyrosine kinase*	6.85	0.000017
*H2*.*0-like homeobox*	6.85	0.000012
*alanyl (membrane) aminopeptidase*	6.85	0.000034
*Rn*.*35760*.*1*	6.83	0.005121
*T-cell receptor beta chain*	6.76	0.005428
*baculoviral IAP repeat-containing 3*	6.76	0.002429
*thromboxane A synthase 1*, *platelet*	6.74	0.000001
*Rn*.*17796*.*1*	6.73	0.002191
*Fc fragment of IgG*, *high affinity Ia*, *receptor (CD64)*	6.72	0.000680
*leukocyte specific transcript 1*	6.70	0.000022
*cytotoxic T lymphocyte-associated protein 2 alpha*	6.68	0.000987
*lectin*, *galactoside-binding*, *soluble*, *3 binding protein*	6.66	0.001503
*CD8a molecule*	6.63	0.001398
*kinesin family member 11*	6.62	0.002488
*RAB32*, *member RAS oncogene family*	6.62	0.017143
*complement component 5a receptor 1*	6.62	0.000004
*extra spindle pole bodies homolog 1 (S*. *cerevisiae)*	6.61	0.008779
*chemokine (C-C motif) ligand 6*	6.60	0.046912
*Rn*.*17556*.*2*	6.57	0.000563
*heme oxygenase (decycling) 1*	6.57	0.001143
*complement factor properdin*	6.56	0.000869
*glia maturation factor*, *gamma*	6.55	0.000210
*Rn*.*12486*.*1*	6.55	0.006555
*Mediterranean fever*	6.51	0.001733
*Rn*.*15124*.*1*	6.49	0.000029
*tumor necrosis factor alpha induced protein 6*	6.49	0.014032
*antigen identified by monoclonal antibody Ki-67*	6.48	0.000633
*Rn*.*17858*.*1*	6.46	0.005273
*membrane bound O-acyltransferase domain containing 1*	6.44	0.002281
*leucine rich repeat (in FLII) interacting protein 1*	6.41	0.000842
*cytochrome b-245*, *alpha polypeptide*	6.39	0.000611
*cathepsin Z*	6.36	0.000395
*collagen*, *type XVIII*, *alpha 1*	6.32	0.003553
*transforming*, *acidic coiled-coil containing protein 3*	6.29	0.000330
*nuclear factor of kappa light polypeptide gene enhancer in B-cells inhibitor*, *zeta*	6.26	0.000425
*transporter 1*, *ATP-binding cassette*, *sub-family B (MDR/TAP)*	6.25	0.000142
*deoxyribonuclease II Alpha*	6.25	0.001119
*matrix metallopeptidase 9*	6.24	0.011841
*adipose differentiation related protein*	6.19	0.000353
*v-maf musculoaponeurotic fibrosarcoma oncogene homolog F (avian)*	6.18	0.003324
*feline sarcoma oncogene*	6.15	0.000002
*Rn*.*15220*.*1*	6.14	0.003527
*thrombospondin 2*	6.14	0.018333
*Src-like adaptor*	6.14	0.001732
*Rn*.*9477*.*1*	6.14	0.001820
*Rn*.*13529*.*1*	6.13	0.000375
*MHC class I RT1*.*O type 149 processed pseudogene*	6.13	0.012003
*colony stimulating factor 3 receptor (granulocyte)*	6.13	0.002592
*bridging integrator 2*	6.12	0.012643
*adenylate cyclase 4*	6.11	0.003223
*basic leucine zipper transcription factor*, *ATF-like*	6.08	0.000157
*SP140 nuclear body protein*	6.07	0.003669
*Rn*.*18190*.*1*	6.05	0.001546
*kinesin family member C1*	6.05	0.003165
*ubiquitin-conjugating enzyme E2T (putative)*	6.03	0.000782
*transmembrane protein 176*^*a*^	6.03	0.000778
*solute carrier family 16*, *member 3 (monocarboxylic acid transporter 4)*	6.03	0.003752
*Rho GTPase activating protein 9*	6.02	0.000443
*Rn*.*37608*.*2*	5.98	0.000032
*transmembrane protein 106*^*a*^	5.97	0.000769
*Rn*.*3765*.*1*	5.96	0.000275
*Rn*.*64479*.*1*	5.95	0.000379
*Rn*.*42802*.*1*	5.95	0.007488
*translocator protein*	5.90	0.000213
*Rn*.*32174*.*1*	5.89	0.000638
*Rn*.*17187*.*1*	5.85	0.000419
*protein tyrosine phosphatase*, *non-receptor type 18*	5.82	0.000003
*angiopoietin-like 4*	5.81	0.000952
*FXYD domain-containing ion transport regulator 2*	5.79	0.000120
*lysosomal protein transmembrane 5*	5.78	0.000543
*aldo-keto reductase family 1*, *member B8*	5.78	0.002604
*Ttk protein kinase*	5.77	0.002488
*serine (or cysteine) peptidase inhibitor*, *clade G*, *member 1*	5.75	0.000012
*toll-like receptor 1*	5.75	0.010590
*immunoglobulin superfamily*, *member 7 /// similar to CLM3 /// similar to dendritic cell-derived immunoglobulin(Ig)-like receptor 1*, *DIgR1—mouse*	5.74	0.000011
*caspase 1*	5.71	0.000852
*Rn*.*6416*.*1*	5.70	0.003004
*chemokine (C-X-C motif) ligand 10*	5.69	0.027229
*ectonucleoside triphosphate diphosphohydrolase 6*	5.67	0.007890
*ectonucleotide pyrophosphatase/phosphodiesterase 3*	5.66	0.004302
*chemokine (C-X-C motif) receptor 4*	5.65	0.000004
*tissue factor pathway inhibitor 2*	5.64	0.000770
*myxovirus (influenza virus) resistance 1*	5.63	0.000013
*schlafen 8*	5.62	0.008481
*poliovirus receptor*	5.61	0.002727
*phosphoinositide-3-kinase adaptor protein 1*	5.61	0.000218
*lymphocyte cytosolic protein 2*	5.54	0.000317
*Rn*.*50688*.*1*	5.53	0.000624
*tubulin*, *beta 6*	5.53	0.001832
*pleckstrin and Sec7 domain containing 4*	5.50	0.000073
*caspase 8*	5.48	0.000226
*minichromosome maintenance complex component 5*	5.47	0.003464
*Rn*.*24916*.*2*	5.47	0.000026
*family with sequence similarity 55*, *member B*	5.46	0.000039
*Similar to paired immunoglobin-like type 2 receptor alpha*	5.46	0.002178
*serine (or cysteine) proteinase inhibitor*, *clade B*, *member 1a*	5.45	0.009948
*peptidylprolyl isomerase C*	5.44	0.004347
*similar to interferon-inducible GTPase*	5.44	0.001651
*RT1 class I*, *locus CE11-like /// RT1 class I*, *locus A3 /// RT1 class I*, *locus CE10 /// RT1 class I*, *locus CE2 /// RT1 class Ib*, *locus EC2*	5.42	0.006253
*paraoxonase 1*	5.42	0.000231
*Rn*.*7834*.*1*	5.41	0.001241
*Rn*.*2721*.*1*	5.41	0.002768
*complement component 4*, *gene 2 /// complement component 4B (Chido blood group)*	5.38	0.000681
*Rn*.*61067*.*1*	5.36	0.009763
*Rn*.*13320*.*1*	5.36	0.000665
*centromere protein E*	5.35	0.006419
*Rn*.*18506*.*1*	5.34	0.002755
*Rn*.*46497*.*1*	5.33	0.000184
*metallothionein 2*^*a*^	5.32	0.000001
*Cell division cycle 20 homolog (S*. *cerevisiae)*	5.31	0.000164
*interleukin 1 receptor*, *type II*	5.29	0.004606
*family with sequence similarity 198*, *member B*	5.28	0.000437
*antisense RNA overlapping MCH*	5.27	0.002693
*hypothetical protein LOC308990*	5.27	0.001784
*Rn*.*4301*.*1*	5.27	0.000002
*Rn*.*7958*.*1*	5.24	0.003443
*interferon regulatory factor 7*	5.24	0.000233
*Rn*.*2548*.*1*	5.23	0.000438
*dedicator of cytokinesis 8*	5.21	0.000069
*Rn*.*35619*.*1*	5.19	0.030130
*SH3-domain binding protein 1*	5.18	0.000191
*Tenascin C*	5.18	0.027698
*NUF2*, *NDC80 kinetochore complex component*, *homolog (S*. *cerevisiae)*	5.17	0.000714
*cholesterol 25-hydroxylase*	5.16	0.000514
*potassium inwardly-rectifying channel*, *subfamily J*, *member 4*	5.16	0.046190
*chemokine (C-C motif) receptor-like 2*	5.13	0.000619
*RAS protein activator like 3*	5.13	0.000006
*Rn*.*37608*.*1*	5.12	0.031019
*Rn*.*13650*.*1*	5.11	0.008676
*ninjurin 1*	5.11	0.000037
*zinc finger*, *FYVE domain containing 1*	5.08	0.003275
*glycoprotein*, *alpha-galactosyltransferase 1*	5.07	0.000122
*amidohydrolase domain containing 2*	5.07	0.001029
*Rn*.*27718*.*1*	5.06	0.000006
*Sterol O-acyltransferase 1*	5.06	0.000990
*ER degradation enhancer*, *mannosidase alpha-like 1*	5.05	0.002228
*SPC25*, *NDC80 kinetochore complex component*, *homolog (S*. *cerevisiae)*	5.04	0.001127
*2'-5'-oligoadenylate synthetase-like*	5.03	0.000043
*similar to Putative protein C21orf45*	5.03	0.004235
*STEAP family member 4*	5.03	0.000295
*TGFB-induced factor homeobox 1*	5.03	0.000095
*T-cell*, *immune regulator 1*, *ATPase*, *H+ transporting*, *lysosomal V0 subunit A3*	5.00	0.000003
*dual specificity phosphatase 2*	5.00	0.000215
*bone morphogenetic protein 7*	4.99	0.004166
*integrin*, *alpha M*	4.98	0.016479
*asp (abnormal spindle) homolog*, *microcephaly associated (Drosophila)*	4.98	0.005363
*CKLF-like MARVEL transmembrane domain containing 3*	4.97	0.000391
*cathepsin K*	4.97	0.000478
*capping protein (actin filament)*, *gelsolin-like*	4.95	0.000073
*Tyro protein tyrosine kinase binding protein*	4.95	0.000002
*v-yes-1 Yamaguchi sarcoma viral related oncogene homolog*	4.95	0.012878
*DSN1*, *MIND kinetochore complex component*, *homolog (S*. *cerevisiae)*	4.94	0.001287
*Rn*.*52525*.*1*	4.94	0.023854
*lectin*, *galactoside-binding*, *soluble*, *3*	4.92	0.000033
*kelch-like 6 (Drosophila)*	4.91	0.000003
*RT1 class Ib*, *locus EC2*	4.91	0.002921
*receptor-interacting serine-threonine kinase 3*	4.90	0.002183
*Epstein-Barr virus induced 3*	4.89	0.000157
*apolipoprotein B*	4.89	0.001464
*similar to 2310014H01Rik protein*	4.88	0.000290
*Rac GTPase-activating protein 1*	4.87	0.001871
*G protein-coupled receptor 84*	4.86	0.001056
*Fc fragment of IgG*, *receptor*, *transporter*, *alpha*	4.86	0.019037
*IQ motif containing GTPase activating protein 3*	4.84	0.000471
*chemokine (C-X-C motif) receptor 4*	4.84	0.000035
*Rn*.*40577*.*1*	4.84	0.002763
*RT1 class II*, *locus DMa*	4.83	0.003158
*interleukin 2 receptor*, *beta*	4.82	0.024770
*myosin IG*	4.82	0.000030
*actin related protein 2/3 complex*, *subunit 1B*	4.82	0.000044
*hypothetical protein LOC689399*	4.82	0.000397
*DnaJ (Hsp40) homolog*, *subfamily B*, *member 12*	4.81	0.000511
*similar to Antxr2 protein*	4.81	0.005512
*RT1 class Ib*, *locus EC2*	4.81	0.004160
*sterol O-acyltransferase 1*	4.78	0.000261
*ring finger protein 213*	4.78	0.006053
*Rn*.*26537*.*1*	4.78	0.027254
*TRAF3 interacting protein 3*	4.78	0.001619
*cell division cycle 20 homolog (S*. *cerevisiae)*	4.78	0.001964
*damage-regulated autophagy modulator*	4.77	0.004459
*Rn*.*65520*.*2*	4.77	0.022947
*Rn*.*12670*.*1*	4.76	0.000443
*DEXH (Asp-Glu-X-His) box polypeptide 58*	4.76	0.000643
*transcription factor 19*	4.75	0.001845
*CCAAT/enhancer binding protein (C/EBP)*, *beta*	4.74	0.000165
*disabled homolog 2 (Drosophila)*	4.74	0.013469
*Rn*.*50630*.*1*	4.71	0.001946
*Rn*.*48053*.*1*	4.71	0.004482
*serine (or cysteine) proteinase inhibitor*, *clade B*, *member 1a*	4.70	0.000003
*Rn*.*47647*.*1*	4.69	0.000116
*PR domain containing 1*, *with ZNF domain*	4.69	0.000179
*Rn*.*19395*.*1*	4.66	0.000117
*leucine rich repeat containing 33*	4.65	0.003258
*collagen*, *type IV*, *alpha 1*	4.65	0.003226
*poly (ADP-ribose) polymerase family*, *member 14*	4.64	0.009531
*procollagen*, *type VII*, *alpha 1*	4.64	0.000676
*interleukin 13 receptor*, *alpha 1*	4.63	0.001698
*immunoglobulin joining chain*	4.63	0.038881
*transmembrane protein 37*	4.63	0.000398
*Rn*.*18088*.*1*	4.62	0.011206
*signal transducer and activator of transcription 1 /// signal transducer and activator of transcription 4*	4.62	0.024167
*RT1 class II*, *locus DMb*	4.61	0.000056
*rCG32064-like*	4.61	0.001592
*CD40 molecule*, *TNF receptor superfamily member 5*	4.60	0.001388
*collagen*, *type IV*, *alpha 1*	4.59	0.008011
*lymphocyte antigen 86*	4.56	0.001124
*Rn*.*16900*.*1*	4.56	0.000006
*protein tyrosine phosphatase*, *non-receptor type 18*	4.56	0.000296
*SHC (Src homology 2 domain containing) transforming protein 1*	4.55	0.000236
*NDC80 homolog*, *kinetochore complex component (S*. *cerevisiae)*	4.53	0.026631
*Rn*.*2746*.*1*	4.51	0.009740
*interferon induced transmembrane protein 3*	4.51	0.000855
*epithelial stromal interaction 1 (breast)*	4.50	0.000081
*Rn*.*43557*.*1*	4.50	0.012087
*septin 6*	4.50	0.000066
*proteolipid protein 2 (colonic epithelium-enriched)*	4.50	0.001055
*Rn*.*12513*.*1*	4.49	0.000389
*RT1 class I*, *locus CE5 /// RT1 class Ib*, *locus EC2*	4.48	0.045842
*cysteine-rich intestinal protein*	4.48	0.007099
*interferon-induced protein with tetratricopeptide repeats 3*	4.47	0.000004
*transglutaminase 2*, *C polypeptide*	4.47	0.001958
*Rn*.*43420*.*1*	4.47	0.003614
*leucine-rich alpha-2-glycoprotein 1*	4.46	0.004151
*vanin 1*	4.45	0.000182
*Rn*.*35620*.*1*	4.44	0.000197
*proteasome (prosome*, *macropain) subunit*, *beta type 9 (large multifunctional peptidase 2)*	4.44	0.002524
*syntaxin 11*	4.43	0.005689
*metallothionein 1*^*a*^	4.42	0.017051
*nuclear receptor subfamily 1*, *group H*, *member 3*	4.42	0.000147
*Rn*.*33681*.*1*	4.41	0.002894
*toll-like receptor 4*	4.39	0.001768
*proline-serine-threonine phosphatase-interacting protein 1*	4.39	0.000187
*Rn*.*3724*.*2*	4.36	0.005443
*tropomyosin 4*	4.36	0.007533
*gasdermin D*	4.36	0.002804
*transmembrane protein 86*^*a*^	4.35	0.000839
*chloride intracellular channel 1*	4.34	0.000191
*Rn*.*23216*.*2*	4.32	0.004487
*T-cell*, *immune regulator 1*, *ATPase*, *H+ transporting*, *lysosomal V0 subunit A3*	4.32	0.000124
*fibrillin 1*	4.31	0.003306
*transmembrane protein 176B*	4.29	0.000154
*NCK associated protein 1 like*	4.27	0.000003
*tumor necrosis factor (TNF superfamily*, *member 2)*	4.27	0.009351
*UDP glucuronosyltransferase 1 family*, *polypeptide A1*, *A2*, *A3*, *A4*, *A5*, *A6*, *A7*, *A8*, *and A9*	4.27	0.001679
*chemokine (C-X-C motif) receptor 4*	4.26	0.000092
*purinergic receptor P2X*, *ligand-gated ion channel 4*	4.26	0.000379
*Rn*.*47453*.*1*	4.25	0.008502
*Friend leukemia virus integration 1*	4.25	0.010038
*Rn*.*41974*.*1*	4.25	0.001107
*integrin*, *beta 2*	4.24	0.001368
*platelet derived growth factor C*	4.23	0.020623
*Rn*.*23216*.*1*	4.23	0.000418
*microsomal glutathione S-transferase 2*	4.22	0.000185
*Phosphoinositide-3-kinase*, *regulatory subunit 6*	4.22	0.000266
*G protein-coupled receptor*, *family C*, *group 5*, *member A*	4.20	0.010505
*collagen*, *type XV*, *alpha 1*	4.20	0.003684
*ADAM metallopeptidase domain 8*	4.20	0.001320
*six transmembrane epithelial antigen of the prostate 1*	4.20	0.004441
*B-cell linker*	4.19	0.000092
*xanthine dehydrogenase*	4.19	0.003713
*Leupaxin*	4.19	0.000488
*Rn*.*24916*.*1*	4.18	0.009254
*troponin T type 1 (skeletal*, *slow)*	4.18	0.004580
*RGD1565926*	4.16	0.003588
*Rn*.*19846*.*1*	4.14	0.002039
*cannabinoid receptor 2 (macrophage)*	4.14	0.021660
*poly (ADP-ribose) polymerase family*, *member 14*	4.14	0.000863
*protein tyrosine phosphatase-like A domain containing 2*	4.12	0.001265
*similar to Protein C8orf4 (Thyroid cancer protein 1) (TC-1)*	4.12	0.035565
*Pleckstrin*	4.12	0.001972
*plexin B2*	4.12	0.000105
*matrix metallopeptidase 7*	4.11	0.001393
*G-protein signaling modulator 3 (AGS3-like*, *C*. *elegans)*	4.11	0.000001
*tubulin*, *beta 5*	4.11	0.015952
*cellular retinoic acid binding protein 2*	4.11	0.010182
*bridging integrator 2*	4.11	0.000080
*Transgelin*	4.10	0.009167
*growth arrest specific 7*	4.10	0.007335
*B-cell CLL/lymphoma 3*	4.09	0.000302
*UDP glucuronosyltransferase 1 family*, *polypeptide A1; UDP glucuronosyl- transferase 1 family*, *polypeptide A2; UDP glycosyltransferase 1 family*, *polypeptide A3; UDP glucuronosyltransferase 1 family*, *polypeptide A5; UDP glucuronosyltransferase 1 family*, *polypeptide A6; UDP glucuronosyl- transferase 1 family*, *polypeptide A7C /// UDP glycosyltransferase 1 family*, *polypeptide A8; UDP glucuronosyltransferase 1 family*, *polypeptide A9*	4.08	0.000178
*Rn*.*19771*.*1*	4.07	0.002784
*Rn*.*3212*.*1*	4.06	0.015501
*Granulin*	4.06	0.000117
*kinesin family member 20B*	4.05	0.003643
*RT1 class Ib*, *locus S3*	4.05	0.003206
*glucosaminyl (N-acetyl) transferase 1*, *core 2 (beta-1*,*6-N-acetylglucosaminyltransferase)*	4.05	0.005215
*Rn*.*20328*.*1*	4.04	0.000013
*ferric-chelate reductase 1*	4.04	0.007403
*chemokine (C-X-C motif) ligand 14*	4.04	0.000385
*Rn*.*8685*.*1*	4.03	0.000060
*RT1 class Ib*, *locus S3*	4.02	0.000175
*PYD and CARD domain containing*	4.02	0.000058
*serine/threonine kinase 10*	4.01	0.000887
*similar to CG3880-PA*	4.01	0.005699
*Glucosamine (N-acetyl)-6-sulfatase*	4.01	0.006479
*CKLF-like MARVEL transmembrane domain containing 6*	4.01	0.000490
*purinergic receptor P2Y*, *G-protein coupled*, *14*	4.00	0.000299
*lymphocyte cytosolic protein 1*	4.00	0.000091
*peroxisome proliferator-activated receptor gamma*, *coactivator-related 1*	4.00	0.002901

**Table 2:** Downregulated genes in the region of the spinal lesion of rats with SCI (7 days post trauma) compared with non-injured rats.

Gene name	Fold Change	P
*NFKB inhibitor interacting Ras-like 1*	-4.00	0.003430
*Rn*.*20701*.*1*	-4.01	0.038684
*Rn*.*46464*.*1*	-4.13	0.001306
*protein phosphatase 1*, *regulatory (inhibitor) subunit 14c*	-4.15	0.003595
*Rn*.*51610*.*1*	-4.20	0.001321
*Rn*.*60179*.*1*	-4.23	0.004754
*peroxisomal biogenesis factor 5-like*	-4.26	0.000456
*Rn*.*18590*.*1*	-4.28	0.000839
*Rn*.*55394*.*1*	-4.34	0.004764
*Rn*.*50930*.*1*	-4.37	0.005187
*Rn*.*32812*.*1*	-4.37	0.000528
*smooth muscle and non-muscle myosin alkali light chain 6B-like*	-4.40	0.002352
*ATPase*, *Ca++ transporting*, *plasma membrane 2*	-4.46	0.010895
*ryanodine receptor 2*, *cardiac*	-4.48	0.003419
*Rn*.*62287*.*1*	-4.52	0.000192
*Rn*.*58970*.*1*	-4.53	0.019291
*glutamate receptor*, *ionotropic*, *N-methyl-D-aspartate 3A*	-4.67	0.000241
*G protein-coupled receptor 61*	-4.70	0.018862
*Hedgehog-interacting protein*	-4.77	0.010322
*Rn*.*51548*.*1*	-4.78	0.001893
*synaptotagmin XII*	-4.81	0.002296
*Hypothetical protein LOC688535*	-4.90	0.008575
*Rn*.*57513*.*1*	-5.04	0.000046
*Rn*.*60594*.*1*	-5.07	0.027516
*Rn*.*50664*.*2*	-5.14	0.004178
*Rn*.*71359*.*1*	-5.23	0.003486
*rCG32052-like*	-5.48	0.001538
*PNMA-like 2*	-5.48	0.019266
*Rn*.*46840*.*1*	-5.78	0.000847
*serpin peptidase inhibitor*, *clade E (nexin*, *plasminogen activator inhibitor type 1)*, *member 3*	-5.85	0.000694
*Rn*.*49823*.*1*	-5.93	0.008502
*Rn*.*46754*.*1*	-6.13	0.001703
*Rn*.*32352*.*1*	-6.17	0.000210
*glycine receptor*, *alpha 1*	-6.22	0.007860
*peroxisomal biogenesis factor 5-like*	-6.27	0.015086
*outer dense fiber of sperm tails 3*	-6.33	0.014749
*Rn*.*59729*.*1*	-6.34	0.004101
*Rn*.*42032*.*1*	-6.71	0.040806
*solute carrier family 12 (potassium/chloride transporters)*, *member 7*	-6.89	0.001097
*Rn*.*20545*.*1*	-7.13	0.000156
*potassium voltage gated channel*, *Shaw-related subfamily*, *member 3*	-7.18	0.037598
*potassium voltage gated channel*, *Shaw-related subfamily*, *member 3*	-7.60	0.031703
*activin A receptor*, *type IC*	-9.13	0.016778

In contrast, treatment with A-HOA only induced changes in the expression of 41 genes, 20 of them overexpressed and 21 underexpressed, in SCI rats (Table 3). Six of these genes were expressed with a difference of more than 4 folds in A-HOA treated rats with respect to saline treated rats (3 genes were overexpressed and 3 underexpressed). Clustering analysis of the data is shown in Fig 3, which graphically represents the differential distribution of samples accrding to the covariance of the expression values obtained for the filtered genes.

**Table 3:** Gene expression modulation in the lesion area of rats with SCI treated with A-HOA compared with saline-treated rats with SCI (7 days post trauma).

Gene Symbol	Gene Name	Baseline mean	Experiment mean	Fold change	P value
*Lum*	*Lumican*	2648.25	5066.18	1.91	0.010056
*Aldh1a2*	*aldehyde dehydrogenase 1 family*, *member A2*	1041.77	556.89	-1.87	0.000260
*Ptges*	*prostaglandin E synthase*	596.47	205.06	-2.91	0.026784
*Pla2g2a*	*phospholipase A2*, *group IIA (platelets*, *synovial fluid)*	594.04	122.13	-4.86	0.035830
*Dusp1*	*dual specificity phosphatase 1*	901.48	530.79	-1.70	0.000711
*Sfrp4*	*secreted frizzled-related protein 4*	415.85	908.59	2.18	0.007671
*Gdf10*	*growth differentiation factor 10*	38.04	151.32	3.98	0.002760
*Apoc1*	*apolipoprotein C-I*	631.65	1449.22	2.29	0.026384
*Slc6a20*	*solute carrier family 6 (proline IMINO transporter)*, *member 20*	404.28	154.00	-2.63	0.016295
*Slc6a20*	*solute carrier family 6 (proline IMINO transporter)*, *member 20*	2448.44	747.27	-3.28	0.007328
*Cyp2d1*	*cytochrome P450*, *family 2*, *subfamily d*, *polypeptide 1 /// cytochrome P450*, *family 2*, *subfamily d*, *polypeptide 5*	195.66	32.11	-6.09	0.022354
*Pla1a*	*phospholipase A1 member A*	826.48	398.31	-2.07	0.014396
*Mx1*	*myxovirus (influenza virus) resistance 1*	694.35	1388.75	2.00	0.016593
*Kng2*	*kininogen 2*	17.58	5.79	-3.04	0.007851
*Mfap4*	*microfibrillar-associated protein 4*	150.67	284.34	1.89	0.002309
*Tpm2*	*tropomyosin 2*, *beta*	100.74	281.72	2.80	0.035562
	*Rn*.*3291*.*1*	428.68	206.19	-2.08	0.004151
*Aoc3*	*amine oxidase*, *copper containing 3 (vascular adhesion protein 1)*	57.19	172.80	3.02	0.025730
*Srpx2*	*sushi-repeat-containing protein*, *X-linked 2*	16.37	82.21	5.02	0.044530
*Tnc*	*Tenascin C*	530.03	1428.34	2.69	0.019260
	*Rn*.*30828*.*1*	40.14	105.15	2.62	0.017407
	*Rn*.*11906*.*1*	613.35	1579.46	2.58	0.037547
*LOC363060*	*similar to RIKEN cDNA 1600029D21*	120.51	43.81	-2.75	0.034124
*Arhgap8*	*Rho GTPase activating protein 8*	28.65	7.32	-3.91	0.048155
*Smarcad1*	*SWI/SNF-related*, *matrix-associated actin-dependent regulator of chromatin*, *subfamily a*, *containing DEAD/H box 1*	19.38	40.18	2.07	0.008265
*Aspn*	*Asporin*	102.11	861.26	8.43	0.004732
*Kng1*	*kininogen 1 /// kininogen 1-like 1 /// kininogen 2*	156.12	46.97	-3.32	0.004672
*Cxcl1*	*chemokine (C-X-C motif) ligand 1 (melanoma growth stimulating activity*, *alpha)*	653.77	144.62	-4.52	0.030424
	*Rn*.*18275*.*1*	206.25	100.05	-2.06	0.002566
	*Rn*.*12277*.*1*	448.85	177.91	-2.52	0.001260
	*Rn*.*20685*.*1*	30.95	68.69	2.22	0.021731
	*Rn*.*42991*.*1*	835.99	466.74	-1.79	0.000244
	*Rn*.*29413*.*1*	25.16	10.48	-2.40	0.012320
*Aspn*	*Asporin*	318.16	1833.62	5.76	0.001719
*Coch*	*coagulation factor C homolog*, *cochlin (Limulus polyphemus)*	329.19	102.74	-3.20	0.029028
*Shisa3*	*shisa homolog 3 (Xenopus laevis)*	137.10	390.53	2.85	0.004579
	*Rn*.*49714*.*1*	291.54	125.38	-2.33	0.000343
*Gpr182*	*G protein-coupled receptor 182*	198.86	91.33	-2.18	0.003590
*P4ha3*	*Procollagen-proline*, *2-oxoglutarate 4-dioxygenase (proline 4-hydroxylase)*, *alpha polypeptide III*	73.12	163.65	2.24	0.040062
*C1qtnf7*	*C1q and tumor necrosis factor related protein 7*	38.47	95.80	2.49	0.003539
	*Rn*.*72710*.*1*	166.69	335.04	2.01	0.012002

**Fig 4:**
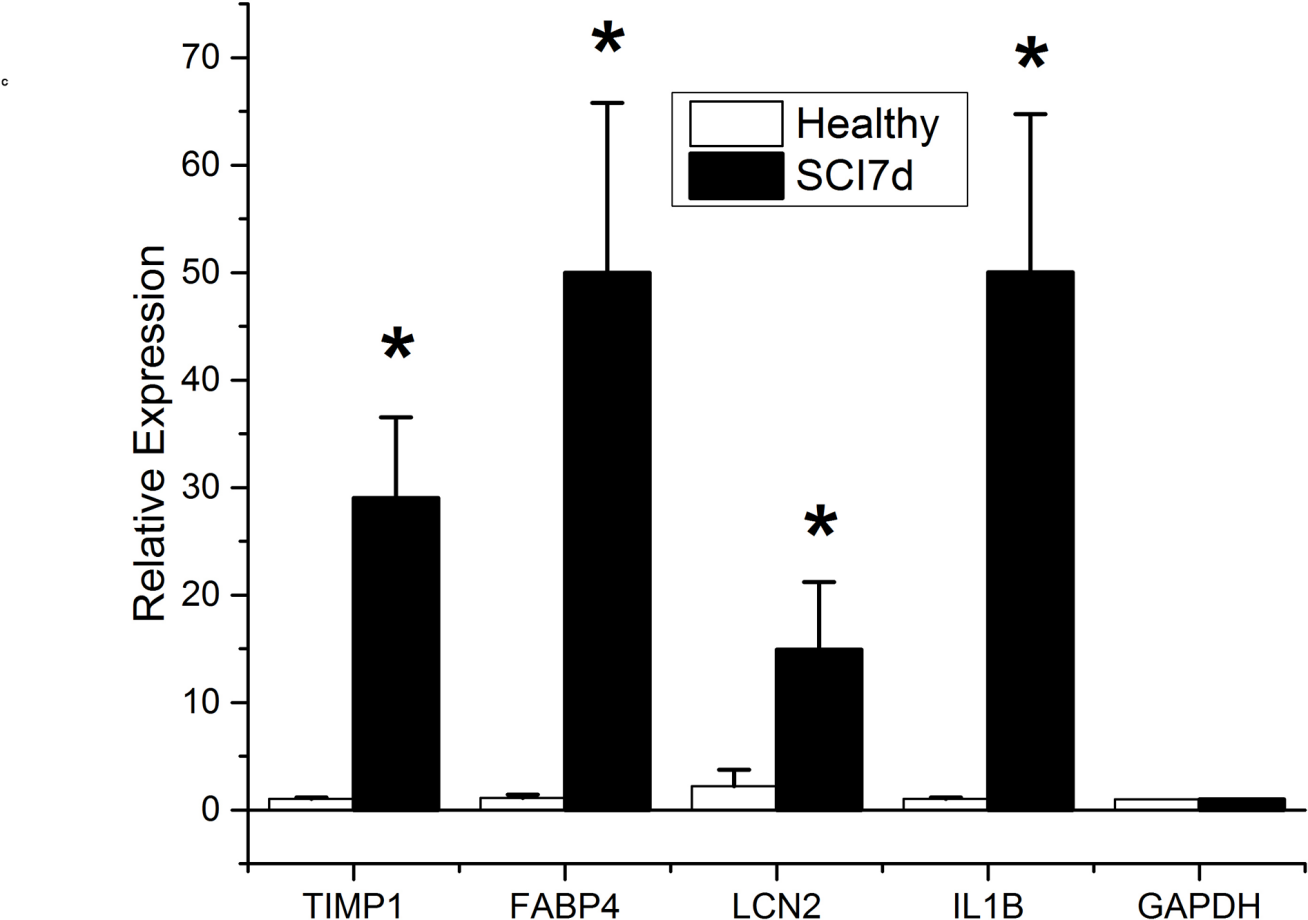
Relative gene expression in SCI rats 7 days after lesion. Levels of the mRNA species indicated were quantified by qRT-PCR in the spinal cord of healthy non-injured rats (open bars) and SCI rats 7 days after contusion (solid bars). The relative expression was calculated from 4 animals using triplicate samples. The samples used were the same as those used for microarray analysis. The relative expression for each gene was calculated with respect to the expression of the housekeeping gene *GAPDH*. *p<0.01.

**Fig 5:**
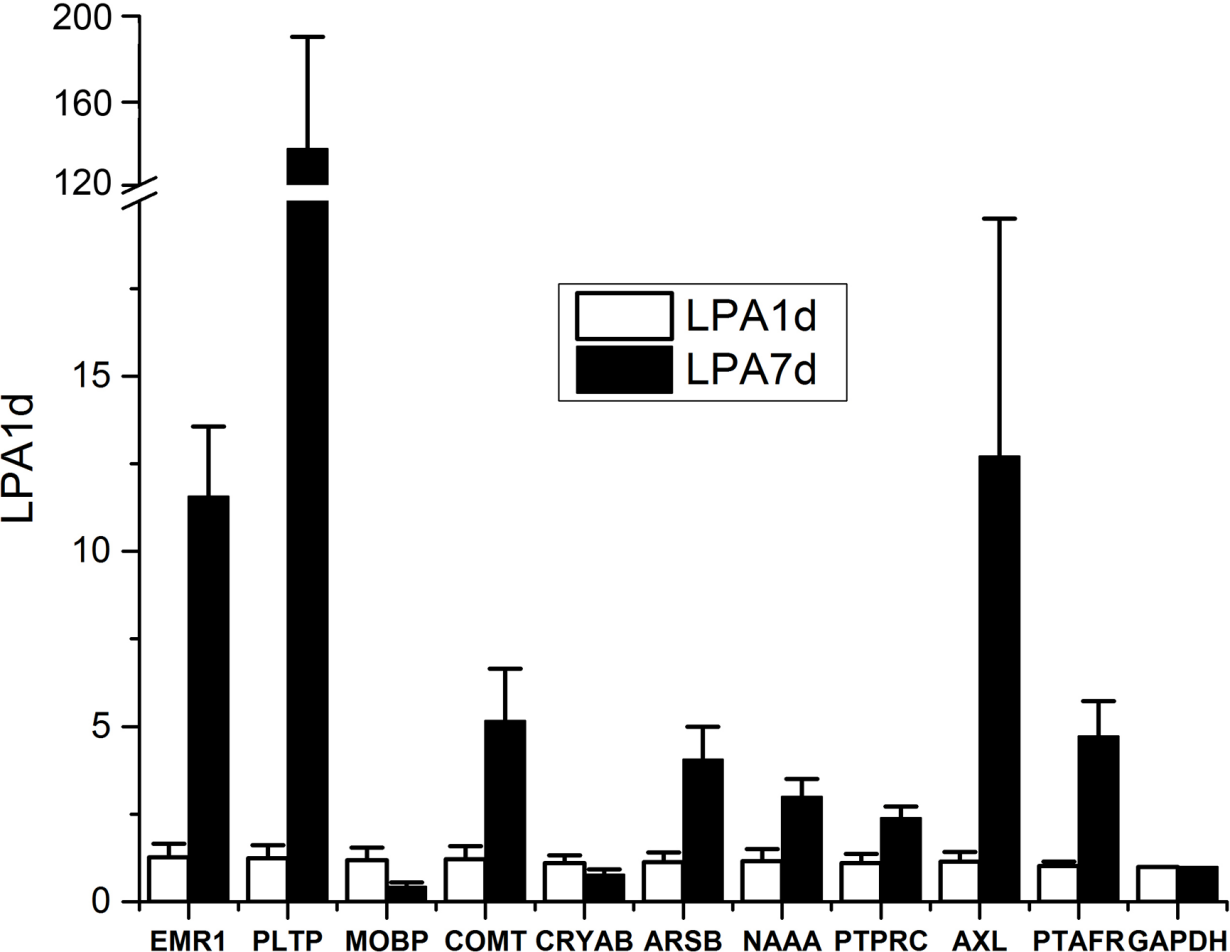
Relative gene expression in SCI rats 1 and 7 days after lesion. Levels of mRNA species quantified by qRT-PCR in the spinal cord of SCI rats 7 days after contusion (solid bars) relative to 1 day expression. The relative expression was calculated from 4 animals using triplicate samples. The samples used were the same as those used for microarray analysis. The relative expression for each gene was calculated with respect to the expression of the housekeeping gene *GAPDH*. *p<0.01.

**Fig 6:**
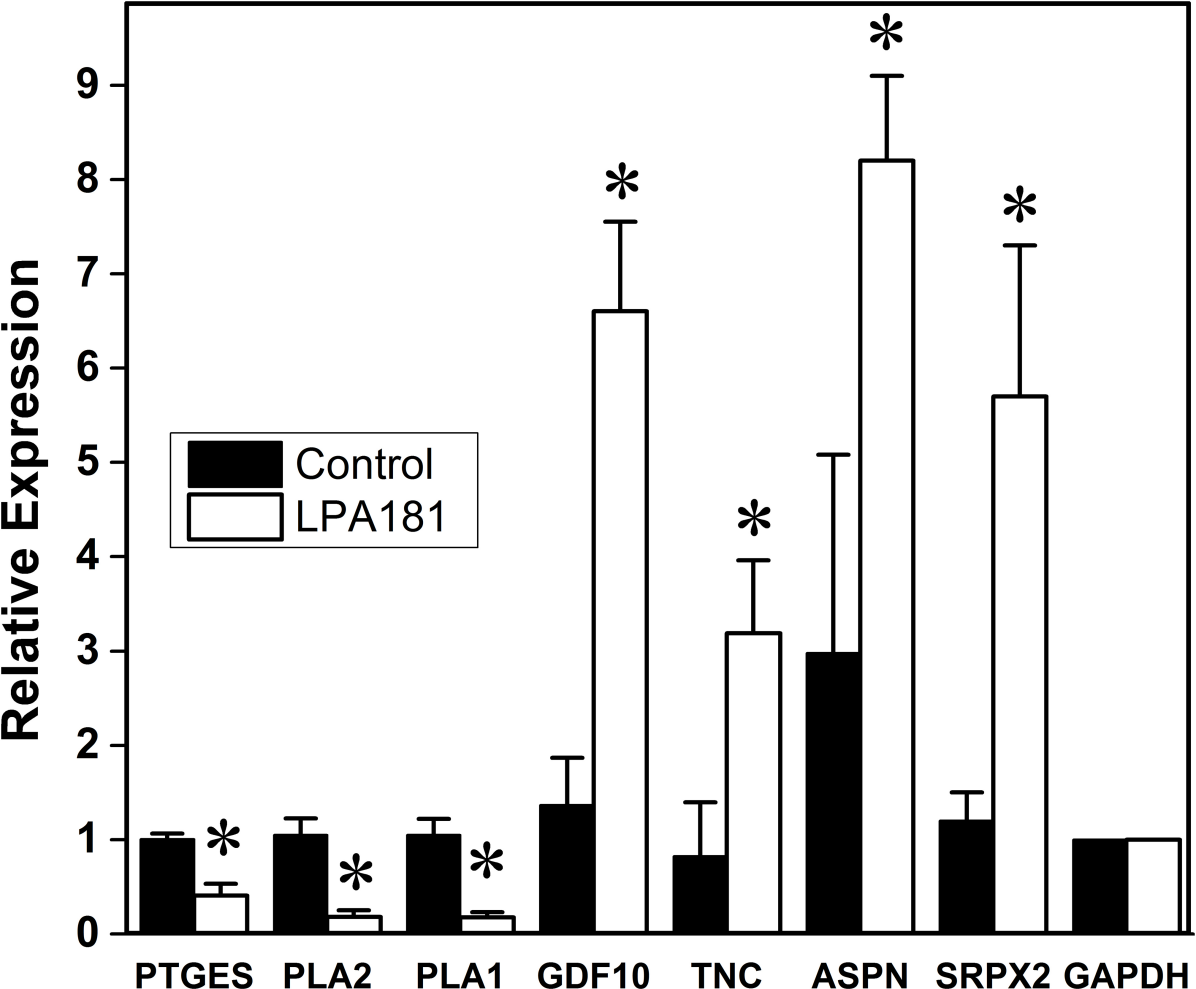
Effect of A-HOA on the relative gene expression of SCI rats 7 days after lesion. Levels of the mRNA species were quantified by qRT-PCR in the spinal cord of SCI rats 7 days after contusion treated with saline vehicle (solid bars) or A-HOA. The relative expression was calculated from 4 animals using triplicate samples. The samples used were the same as those used for microarray analysis. The relative expression for each gene was calculated with respect to the expression of the housekeeping gene *GAPDH*. *p<0.01.

To further validate the changes observed in microarray analyses, we also measured the expression of a number of genes relevant in the context of SCI and the therapeutic effects of A-HOA in nociception control and motor activity (Figs 4–6). In this context, genes such as *TIMP1*, *LCN2 and IL1B* were significantly increased after SCI in the spinal cord lesion area (Fig 4).

In addition to the low number of genes altered in SCI rats treated with A-HOA (Table 3), some of them showed an expression opposite to that of non-treated (Control) SCI rats (Fig 5). An example is the inflammation-related protein, *prostaglandin E (PGE) synthase (PTGES)*, whose expression is markedly and significantly increased in SCI rats (Table 1) but significantly decreased SCI rats after treatement with A-HOA (Table 3, Fig 5). In contrast, *growth differentiation factor 10 (GDF10)* was significantly increased only in SCI rats treated with A-HOA.

## Discussion

Spinal injuries have a prevalence ranging from 250–900 patients per million inhabitants in different countries and regional areas [51], and over 90% of them are affected by important losses in voluntary mobility, while spasticity and neuropathic pain affects over 80% of patients with SCI [25, 26, 52]. In this context, there are unmet clinical needs to treat this condition and the symptoms associated with it [51].

In the present study, we showed that intrathecal administration of the A-HOA complex (every third day during 28 days) induced a marked and significant recovery of the voluntary motor function (ca. 70%, Fig 1). Moreover, A-HOA induced a marked and significant reduction with a concomitant inhibition of cutaneous noxious reflex activity and central sensitization to noxious stimuli, which indicates a possible application for spasticity and neuropathic pain (Fig 2). These results are in agreement with the previous studies showing that A-HOA could reduce pain in rats with SCI [24]. Therefore, A-HOA could constitute a potential treatment for paralysis, spasticity and pain in patients with SCI.

In this context, we observed a dramatic modification of genes within the damaged spinal tissue (Fig 3, Tables 1, 2 and 3 and S1 Table). Thus, the expression of almost 4,000 genes was significantly altered by SCI, in most cases showing upregulation (S1 Table). Considering a 4-fold threshold, ca. 550 gene products were significantly overexpressed (Table 1), whereas only 43 were underexpressed (Table 2). These results indicate that cells in the area of the spinal injury respond by activating several signaling mechanisms. By contrast, treatment with A-HOA induced a limited gene expression regulation. In this context, only 41 genes were significantly up- (20 gene products) or downregulated (21 gene products) in rats with SCI treated with A-HOA with respect to rats treated with saline, with 3 genes being overexpressed more than 4-fold and another 3 gene products downregulated to a similar extent 7 days after injury (Table 3). These results indicate that treatment with A-HOA had targeted only a few regulatory mechanisms over a week after SCI involved in the therapeutic effects mediated by A-HOA.

In the search for the mechanisms involved in SCI pathophysiology and therapy, and also as a means for the validation of the technique, we further evaluated the expression of selected genes using real-time qRT-PCR. We found that all genes whose expression appeared to be higher or lower in DNA microarray experiments also showed the same expression change trend after qRT-PCR quantification, although the absolute values were not identical. These results indicate that the microarray approach used was appropriate to accurately evaluate gene expression alterations. In this context, our results on the pathophysiological alterations induced by SCI agree with previous studies showing relevant expression modulation in genes which regulate diverse functions: stress and apoptosis, inflammation, cytoskeletal proteins, metal response elements, growth factors and receptors, cell cycle and neurotransmission [53]. In this scenario, the relevance of the results obtained in treated rats after SCI also resides in the number of genes regulated by A-HOA associated with motor activity regulation, such as *Aspn (Asporin)*. This gene encodes an extracellular matrix member of the small leucine-rich proteoglycan protein family involved in regulation of cartilage and bones and is altered in patients with vertebral pathologies. Moreover, Aspn has been associated with development of the CNS and therefore it could play a crucial role in the neural damage recovery after SCI and the therapeutic effects of A-HOA [54]. More specifically, this gene could be involved in the recovery of the extracellular matrix of the tissue damaged after SCI. In fact, Aspn expression already is high (ca. 3-fold) in animals treated with saline and treatment with A-HOA causes further increases (ca. 6–8 fold), which indicates the relevance of this gene product in the physiological and pharmacological recovery after spinal injury.

Similarly, *Growth Differentiation Factor 10 (GDF10)* is a member of the bone morphogenetic protein family and the TGF-α superfamily, which is involved in the anti-inflammatory activity of certain cytokines and in alleviating nerve injury-induced neuropathic pain in rats [55]. Moreover, *GDF10* has been recently reported to be a signal for axonal sprouting and neuron functional recovery after stroke [56]. The members of this family are regulators of cell growth and differentiation in both embryonic and adult tissues. Interestingly, this protein is also expressed in adipocytes, where it inhibits adipogenesis [57]. Because the CNS has very high lipid content, it is feasible that this protein could be involved in the lipid metabolism and nerve regeneration. Therefore, the neurotrophic, anti-inflammatory, analgesic and metabolic roles of *GDF10* could play critical roles in the recovery from SCI.

Another protein overexpressed in A-HOA-treated SCI rats was *tenascin C (TNC)*. This protein is involved in regulating the proliferation of both oligodendrocyte precursor cells and astrocytes. *TNC* is present in central nervous system injuries and gliomas [58]. In this context, in *TNC* defficient mice improved axonal sprouting has been observed, suggesting that this protein may interfere with nerve recovery after SCI. However, the fact that A-HOA induces *TNC* overexpression followed by generalized motor recovery could indicate that this is one of the molecular cell events associated with recovery from SCI. *TNC* has also been related to extracellular matrix alterations, accelerated leukocyte infiltration and enhanced axonal sprouting after spinal cord hemisection in *tenascin-C*-deficient mice [58].

Another gene with a markedly and significantly increased expression in A-HOA-treated SCI rats with respect to saline-treated rats was *sushi-repeat-containing protein X-linked 2 (SRPX2)*. This gene encodes a secreted protein with 3 sushi repeats, and has a relevant role in cognitive activities, such as speech and language, as well as in angiogenesis [59,60]. Moreover, alterations in the *SRPX2* gene are associated with bilateral perisylvian polymicrogyria, rolandic epilepsy, speech dyspraxia and mental retardation. In addition, it participates in cell migration and adhesion, activates angiogenesis and promotes synapse formation [61]. These roles suggest that *SRPX2* may play an important role to restablish vascularization and recover synapse loss associated with SCI. In fact, mutations in *SRPX2* have been linked to neurological syndromes with altered neuronal migration [62]. In summary, this evidence suggests that *SRPX2* could play a role in functional recovery in rats with SCI.

Some kind of lipids are able to regulate inflammatory mediators through complex mechanisms to promote or inhibit inflammation [63–68]. In our study, genes related to inflammation, such as *PTGES*, *PLA1 and PLA2* were repressed at least 4-fold. In this context, *PTGES* gene encodes for a glutathione-dependent PGE synthase. The expression of this gene has been shown to be induced by proinflammatory cytokine *interleukin 1 beta (IL-1B) and by tumor suppressor protein TP53*, and may be involved in *TP53*-induced apoptosis. Knockout studies in mice suggest that this gene may contribute to the pathogenesis of collagen-induced arthritis and mediate acute pain during inflammatory responses. In agreement with this, it has been seen that intrathecal *PGE2* administration induces hyperalgesia and allodynia, the latter tactile hypersensitivity effect observed in rats being often associated with neuropathic pain in patients [69, 70]. Moreover, knockout mice lacking the membrane enzyme that produces *PGE2 (mPGES-1*^*-/-*^*)* did not exhibit mechanical allodynia, while retained normal nociceptive responses after spinal nerve transection, which demonstrates the involvement of this protein in neuropathic and inflammatory pain [71,72] and in the therapeutic effects mediated by A-HOA. In addition, *PGE2* inhibits microglial migration in the spinal cord, which could further interfere with SCI therapy [72], so that *PTGES* inhibition by A-HOA would also permit glial cell trafficking [40].

The other two genes, *PLA1 and PLA2*, encode for *phospholipases A1 and A2*, respectively. These two enzymes produce lysophospholipids and fatty acids, such as arachidonic acid, a well-known inflammatory mediator that causes hyperalges**i**a [73]. On the one hand, it has been reported that *PLA1* plays a relevant role in 1-oleoyl-2-palmitoyl-phosphatidylcholine turnover in neurons, a lipid that regulates localization of signaling proteins to defined synaptic areas [74]. Furthermore, *PLA2* induction after SCI or intrathecal *PLA2* injection itself can cause axon demyelination and focal hemorrhagic pathology, suggesting that inhibition of *PLA2* might be associated with remyelination in the spinal contusion area after treatment with A-HOA [75]. Therefore, the reduction in animals of *PLA2* following treatment with A-HOA may contribute to reduced inflammation, nociception and cell death in the area of SCI.

Inhibition of *PLA2* by Annexins produces a post-traumatic anti-inflammatory effect, suggesting that the therapeutic effect of A-HOA could also be related to the inhibition of progressive tissue damage after SCI, due in part to repression of *PLA1/2* expression [76]. Further studies are required to assess the role played by these target genes in the pro and anti-inflammatory effects related to SCI.

In line with these results, significant changes were found in the spinal lesion area of A-HOA rats treated for 1 and 7 days, respectively (Fig 5). One of the most relevant changes was the alteration in the expression of the *phospholipid transfer protein*, *PLTP*, whose expression was found increased after 7 days of treatment with respect to the first day of treatment both after DNA microarray (13.6-fold; p<0.001) or qRT-PCR (over 100-fold change; p<0.001) quantification. This result further indicates the relevance of lipids in the pathophysiology and therapy of SCI. However, it should be ruled out the possibility that fatty acids in general could have therapeutic effects against SCI. In this context, it has been clearly shown that cis-monounsaturated fatty acids, such as HOA and its analog, oleic acid, induce changes in the structure and function of membrane lipids and proteins that are not paralleled by other fatty acids with identical (e.g., elaidic acid) or similar (e.g., stearic acid) chemical composition but with different structure [77,78]. Thus, the structure of fatty acids is crucial to modulate the structure of membranes and ensuing signaling events [48, 79, 80]. Membrane-drug interactions play critical roles in the efficacy of certain compounds [81] and the general mechanisms underlying the effects of synthetic fatty acids and related compunds (e.g., A-HOA) on the cell’s physiology and gene expression have been summarized elsewhere [82,83].

In summary, in the present study we showed that the complex made between the lipid binding protein, albumin, and the synthetic lipid, 2-hydroxyoleic acid, showed a high efficacy to promote sensorimotor recovery after SCI, having been identified by DNA microarray and RT-PCR analyses a number of genes with a potentially relevant role for therapy. This therapeutic complex (A-HOA) could be of clinical interest for the treatment of motor function, the spasticity syndrome and the control of neuropathic pain in patients with spinal cord injury. Further experimental studies are required using behavioural and histological techniques to identify the role of the new gene targets modulated by A-HOA in this study.

## Supporting information

S1 Table Gene expression modulation in the lesion area of rats with SCI (7 days post trauma) compared with non-injured rats. This table presents approximately 3,900 genes/transcripts that undergo changes over 2 fold when gene expression of SCI (7 days post injury) and non injured animals were compared.
(DOCX)

## References

[1] Hulsebosch CE (2002) Recent advances in pathophysiology and treatment of spinal cord injury. Adv Physiol Educ 26:238–255. 12443996 10.1152/advan.00039.2002

[2] Rossignol S, Schwab M, Schwartz M, Fehlings MG (2007) Spinal cord injury: time to move?. J Neurosci 27:11782–11792. doi: 10.1523/JNEUROSCI.3444-07.2007 17978014 10.1523/JNEUROSCI.3444-07.2007 PMC6673354

[3] Bareyre FM, Schwab ME (2003) Inflammation, degeneration and regeneration in the injured spinal cord: insights from DNA microarrays. Trends Neurosci 26: 555–563. doi: 10.1016/j.tins.2003.08.004 14522149 10.1016/j.tins.2003.08.004

[4] Sandler AN, Tator CH (1976) Review of the measurement of normal spinal cord blood flow. Brain Res 118:181–198. 826305 10.1016/0006-8993(76)90707-1

[5] Simard JM, Popovich PG, Tsymbalyuk O, Gerzanich V (2012) Spinal cord injury with unilateral versus bilateral primary hemorrhage-effects of glibenclamide. Exp Neurol 233:829–835. doi: 10.1016/j.expneurol.2011.11.048 22197047 10.1016/j.expneurol.2011.11.048 PMC3272086

[6] Bethea JR, Dietrich WD (2002) Targeting the host inflammatory response in traumatic spinal cord injury. Curr Opin Neurol 15:355–360. 12045737 10.1097/00019052-200206000-00021

[7] Alexander JK. Popovich PG (2009) Neuroinflammation in spinal cord injury: therapeutic targets for neuroprotection and regeneration. Prog Brain Res 175:125–137. doi: 10.1016/S0079-6123(09)17508-8 19660652 10.1016/S0079-6123(09)17508-8

[8] Benowitz LI, Popovich PG (2011) Inflammation and axon regeneration. Curr Opin Neurol 24:577–583. doi: 10.1097/WCO.0b013e32834c208d 21968547 10.1097/WCO.0b013e32834c208d

[9] Bollaerts I, Van Houcke J, Andries L, De Groef L, Moons L (2017) Neuroinflammation as Fuel for Axonal Regeneration in the Injured Vertebrate Central Nervous System. Mediators Inflamm. 2017:9478542 doi: 10.1155/2017/9478542 28203046 10.1155/2017/9478542 PMC5288536

[10] David S, Greenhalgh AD, Kroner A (2015) Macrophage and microglial plasticity in the injured spinal cord. Neuroscience. 10 29;307:311–8. doi: 10.1016/j.neuroscience.2015.08.064 26342747 10.1016/j.neuroscience.2015.08.064

[11] Francos-Quijorna I, Amo-Aparicio J, Martinez-Muriana A, López-Vales R (2016) IL-4 drives microglia and macrophages toward a phenotype conducive for tissue repair and functional recovery after spinal cord injury. Glia. 12;64(12):2079–2092. doi: 10.1002/glia.23041 27470986 10.1002/glia.23041

[12] Gensel J. C., Nakamura S., Guan Z., van Rooijen N., Ankeny D. P., and Popovich P. G. (2009) Macrophages promote axon regeneration with concurrent neurotoxicity. Journal of Neuroscience, vol. 29, no. 12, pp. 3956–3968. doi: 10.1523/JNEUROSCI.3992-08.2009 19321792 10.1523/JNEUROSCI.3992-08.2009 PMC2693768

[13] Stirling D. P., Cummins K., Mishra M., Teo W., Yong V.W., and Stys P. (2014) Toll-like receptor 2-mediated alternative activation of microglia is protective after spinal cord injury. Brain, vol. 137, no. 3, pp. 707–723. 24369381 10.1093/brain/awt341

[14] Kigerl K. A., Gensel J. C., Ankeny D. P., Alexander J. K., Donnelly D. J., and Popovich P. G. (2009) Identification of two distinct macrophage subsets with divergent effects causing either neurotoxicity or regeneration in the injured mouse spinal cord. Journal of Neuroscience, vol. 29, no. 43, pp. 13435–13444. doi: 10.1523/JNEUROSCI.3257-09.2009 19864556 10.1523/JNEUROSCI.3257-09.2009 PMC2788152

[15] Carmel JB, Galante A, Steropoulos P, Tolias P, Recce M, Young W et al (2001) Gene expression profiling of acute spinal cord injury reveals spreading inflammatory signals and neuron loss. Physiol Genomics 7:201–213. doi: 10.1152/physiolgenomics.00074.2001 11773606 10.1152/physiolgenomics.00074.2001

[16] Song G, Cechvala C, Resnick DK, Dempsey RJ, Rao VL (2001) GeneChip analysis after acute spinal cord injury in rat. J Neurochem 79:804–815. 11723173 10.1046/j.1471-4159.2001.00626.x

[17] Nishimura S, Sasaki T, Shimizu A, Yoshida K, Iwai H, Koya I et al (2014) Global gene expression analysis following spinal cord injury in non-human primates. Exp Neurol 261:171–179. doi: 10.1016/j.expneurol.2014.05.021 24873731 10.1016/j.expneurol.2014.05.021

[18] Torres-Espin A, Hernandez J, Navarro X (2013) Gene expression changes in the injured spinal cord following transplantation of mesenchymal stem cells or olfactory ensheathing cells. PLoS One 8:e76141 doi: 10.1371/journal.pone.0076141 24146830 10.1371/journal.pone.0076141 PMC3795752

[19] Gris P, Tighe A, Thawer S, Hemphill A, Oatway M, Weaver L et al (2009) Gene expression profiling in anti-CD11d mAb-treated spinal cord-injured rats. J Neuroimmunol 209:104–113. doi: 10.1016/j.jneuroim.2009.02.002 19250688 10.1016/j.jneuroim.2009.02.002

[20] Nesic O, Svrakic NM, Xu GY, McAdoo D, Westflund KN, Hulsebosch CE et al (2002) DNA microarray analysis of the contused spinal cord: effect of NMDA receptor inhibition. J Neurosci Res 68:406–423. doi: 10.1002/jnr.10171 11992467 10.1002/jnr.10171

[21] Santiago JM, Rosas O, Torrado AI, González MM, Kalyan-Masih PO, Miranda JD (2009) Molecular, anatomical, physiological, and behavioral studies of rats treated with buprenorphine after spinal cord injury. J Neurotrauma 26:1783–1793. doi: 10.1089/neu.2007.0502 19653810 10.1089/neu.2007.0502 PMC2864459

[22] Skold C, Levi R, Seiger A (1999) Spasticity after traumatic spinal cord injury: nature, severity, and location. Arch Phys Med Rehabil 80:1548–1557. 10597805 10.1016/s0003-9993(99)90329-5

[23] Dietz V, Sinkjaer T (2007) Spastic movement disorder: impaired reflex function and altered muscle mechanics. Lancet Neurol 6:725–733. doi: 10.1016/S1474-4422(07)70193-X 17638613 10.1016/S1474-4422(07)70193-X

[24] Avila-Martin G, Galan-Arriero I, Gómez-Soriano J, Taylor J (2011) Treatment of rat spinal cord injury with the neurotrophic factor albumin-oleic acid: translational application for paralysis, spasticity and pain. PLoS One 6:e26107 doi: 10.1371/journal.pone.0026107 22046257 10.1371/journal.pone.0026107 PMC3202524

[25] Bravo-Esteban E, Taylor J, Abián-Vicén J, Albu S, Simón-Martínez C, Torricelli D et al (2013) Impact of specific symptoms of spasticity on voluntary lower limb muscle function, gait and daily activities during subacute and chronic spinal cord injury. NeuroRehabilitation 33:531–543. doi: 10.3233/NRE-131000 24018366 10.3233/NRE-131000

[26] Gómez-Soriano J, Bravo-Esteban E, Pérez-Rizo E, Ávila-Martín GM, Galán-Arriero I, Simón-Martínez C et al (2016) Abnormal cutaneous flexor reflex activity during controlled isometric plantar flexion in human spinal cord injury spasticity syndrome. Spinal Cord 54:687–94. doi: 10.1038/sc.2016.9 26902460 10.1038/sc.2016.9

[27] Finnerup NB, Jensen TS (2004) Spinal cord injury pain-mechanisms and treatment. Eur J Neurol 11:73–82. 14748766 10.1046/j.1351-5101.2003.00725.x

[28] Finnerup NB, Sorensen L, Biering-Sorensen F, Johannesen IL, Jensen TS (2007) Segmental hypersensitivity and spinothalamic function in spinal cord injury pain. Exp Neurol 207:139–149. doi: 10.1016/j.expneurol.2007.06.001 17628539 10.1016/j.expneurol.2007.06.001

[29] Wasner G, Lee BB, Engel S, McLachlan E (2008) Residual spinothalamic tract pathways predict development of central pain after spinal cord injury. Brain 131:2387–2400. doi: 10.1093/brain/awn169 18669485 10.1093/brain/awn169

[30] Gomez-Soriano J, Goiriena E, Florensa-Vila J, Gómez-Arguelles JM, Mauderli A, Vierck CJ Jr (2012) Sensory function after cavernous haemangioma: a case report of thermal hypersensitivity at and below an incomplete spinal cord injury. Spinal Cord 50:711–715. doi: 10.1038/sc.2012.69 22733175 10.1038/sc.2012.69

[31] Dietz V, Grillner S, Trepp A, Hubli M, Bollinger M (2009) Changes in spinal reflex and locomotor activity after a complete spinal cord injury: a common mechanism? Brain 132:2196–2205. doi: 10.1093/brain/awp124 19460795 10.1093/brain/awp124

[32] Taylor J, Huelbes S, Albu S, Gómez-Soriano J, Peñacoba C, Poole HM (2012) Neuropathic pain intensity, unpleasantness, coping strategies, and psychosocial factors after spinal cord injury: an exploratory longitudinal study during the first year. Pain Med 13:1457–1468. doi: 10.1111/j.1526-4637.2012.01483.x 22994208 10.1111/j.1526-4637.2012.01483.x

[33] Kwon BK, Okon EB, Plunet W, Baptiste D, Fouad K, Hillyer J et al (2011) A systematic review of directly applied biologic therapies for acute spinal cord injury. J Neurotrauma 28:1589–1610. doi: 10.1089/neu.2009.1150 20082560 10.1089/neu.2009.1150 PMC3143411

[34] Hagg T, Oudega M (2006) Degenerative and spontaneous regenerative processes after spinal cord injury. J Neurotrauma 23:264–280. doi: 10.1089/neu.2006.23.263 16629615 10.1089/neu.2006.23.263

[35] Kakulas BA (2004) Neuropathology: the foundation for new treatments in spinal cord injury. Spinal Cord 42:549–563. doi: 10.1038/sj.sc.3101670 15346131 10.1038/sj.sc.3101670

[36] Dietz V, Curt A (2006) Neurological aspects of spinal-cord repair: promises and challenges. Lancet Neurol 5:688–694. doi: 10.1016/S1474-4422(06)70522-1 16857574 10.1016/S1474-4422(06)70522-1

[37] Bradbury EJ, McMahon SB (2006) Spinal cord repair strategies: why do they work? Nat Rev Neurosci 7:644–653. doi: 10.1038/nrn1964 16858392 10.1038/nrn1964

[38] Gomez-Soriano J, Goiriena E, Taylor J (2010) Spasticity therapy reacts to astrocyte GluA1 receptor upregulation following spinal cord injury. Br J Pharmacol 161:972–975. doi: 10.1111/j.1476-5381.2010.00964.x 20662840 10.1111/j.1476-5381.2010.00964.x PMC2998679

[39] Hefferan MP, Kucharva K, Kinjo K, Kakinohana O, Sekerkova G, Nakamura S (2007) Spinal astrocyte glutamate receptor 1 overexpression after ischemic insult facilitates behavioral signs of spasticity and rigidity. J Neurosci 27:11179–11191. doi: 10.1523/JNEUROSCI.0989-07.2007 17942713 10.1523/JNEUROSCI.0989-07.2007 PMC6673044

[40] Hains BC, Waxman SG (2006) Activated microglia contribute to the maintenance of chronic pain after spinal cord injury. J Neurosci 26:4308–4317. doi: 10.1523/JNEUROSCI.0003-06.2006 16624951 10.1523/JNEUROSCI.0003-06.2006 PMC6674010

[41] Fouad K, Rank MM, Vavrek R, Murray KC, Sanelli L, Bennett DJ (2010). Locomotion after spinal cord injury depends on constitutive activity in serotonin receptors. J Neurophysiol 104:2975–2984. doi: 10.1152/jn.00499.2010 20861436 10.1152/jn.00499.2010 PMC3007654

[42] Rank MM, Murray KC, Stephens MJ, D'Amico J, Gorassini MA, Bennett DJ. Adrenergic receptors modulate motoneuron excitability, sensory synaptic transmission and muscle spasms after chronic spinal cord injury. J Neurophysiol 105:410–422. doi: 10.1152/jn.00775.2010 21047936 10.1152/jn.00775.2010 PMC3023364

[43] Courtine G, Gerasimenko Y, van den Brand R, Yew A, Musienko P, Zhong H et al (2009) Transformation of nonfunctional spinal circuits into functional states after the loss of brain input. Nat Neurosci 12:1333–1342. doi: 10.1038/nn.2401 19767747 10.1038/nn.2401 PMC2828944

[44] Fandel D, Wasmuht D, Avila-Martin G, Taylor JS, Galan-Arriero I, Mey J (2013) Spinal cord injury induced changes of nuclear receptors PPARα and LXRβ and modulation with oleic acid/albumin treatment. Brain Res 1535:89–105. doi: 10.1016/j.brainres.2013.08.022 23958344 10.1016/j.brainres.2013.08.022

[45] Mandrekar-Colucci S, Sauerbeck A, Popovich PG, McTigue DM (2013) PPAR agonists as therapeutics for CNS trauma and neurological diseases. ASN Neuro 5:e00129 doi: 10.1042/AN20130030 24215544 10.1042/AN20130030 PMC3866683

[46] Alemany R., Terés S, Baamonde C, Benet M, Vögler O, Escribá PV (2004) 2-hydroxyoleic acid: a new hypotensive molecule. Hypertension 43:249–254. doi: 10.1161/01.HYP.0000107778.85528.b5 14662651 10.1161/01.HYP.0000107778.85528.b5

[47] Vogler O, López-Bellan A, Alemany R, Tofé S, González M, Quevedo J et l (2008) Structure-effect relation of C18 long-chain fatty acids in the reduction of body weight in rats. Int J Obes 32:464–473. doi: 10.1038/sj.ijo.0803768 18059405 10.1038/sj.ijo.0803768

[48] Martinez J, Vögler O, Casas J, Barceló F, Alemany R, Prades J et al (2005) Membrane structure modulation, protein kinase C alpha activation, and anticancer activity of minerval. Mol Pharmacol 67:531–540. doi: 10.1124/mol.104.000778 15531732 10.1124/mol.104.000778

[49] Alemany R, Vögler O, Terés S, Egea C, Baamonde C, Barceló F et al (2006) Antihypertensive action of 2-hydroxyoleic acid in SHRs via modulation of the protein kinase A pathway and Rho kinase. J Lipid Res 47:1762–1770. doi: 10.1194/jlr.M500520-JLR200 16687663 10.1194/jlr.M500520-JLR200

[50] Avila-Martin G, Galan-Arriero I, Ferrer-Donato A, Busquets X, Gomez-Soriano J, Escribá PV et al (2015) Oral 2-hydroxyoleic acid inhibits reflex hypersensitivity and open-field-induced anxiety after spared nerve injury. Eur J Pain 19:111–122. doi: 10.1002/ejp.528 24824524 10.1002/ejp.528

[51] Singh A, Tetreault L, Kalsi-Ryan S, Nouri A, Fehlings MG (2014) Global prevalence and incidence of traumatic spinal cord injury. Clin Epidemiol 6:309–331. doi: 10.2147/CLEP.S68889 25278785 10.2147/CLEP.S68889 PMC4179833

[52] Siddall PJ, Middleton JW (2006) A proposed althorithm for the management of pain following spinal cord injury. Spinal Cord 44:67–77. doi: 10.1038/sj.sc.3101824 16116488 10.1038/sj.sc.3101824

[53] Yip PK and Malaspina A (2012) Spinal cord trauma and the molecular point of no return. Mol Neurodegener 7:6 doi: 10.1186/1750-1326-7-6 22315999 10.1186/1750-1326-7-6 PMC3299607

[54] Dangaria SJ, Ito Y, Luan X, Diekwisch TG (2011) Differentiation of neural-crest-derived intermediate pluripotent progenitors into committed periodontal populations involves unique molecular signature changes, cohort shifts, and epigenetic modifications. Stem Cells Dev 20:39–52. doi: 10.1089/scd.2010.0180 20604680 10.1089/scd.2010.0180 PMC3128775

[55] Echeverry S, Shi XQ, Haw A, Liu H, Zhang ZW, Zhang J (2009) Transforming growth factor-β_1_ impairs neuropathic pain through pleiotropic effects. Mol Pain 5:16 doi: 10.1186/1744-8069-5-16 19327151 10.1186/1744-8069-5-16 PMC2669449

[56] Li S, Nie EH, Yin Y, Benowitz LI, Tung S, Vinters HV (2015) GDF10 is a signal for axonal sprouting and functional recovery after stroke. Nat Neurosci 18:1737–1745. doi: 10.1038/nn.4146 26502261 10.1038/nn.4146 PMC4790086

[57] Hino J, Miyazawa T, Miyazato M, Kangawa K (2012) Bone morphogenetic protein-3b (BMP-3b) is expressed in adipocytes and inhibits adipogenesis as a unique complex. Int J Obes 36:725–734. doi: 10.1038/ijo.2011.124 21712809 10.1038/ijo.2011.124 PMC3348488

[58] Schreiber J, Schachner M, Schumacher U, Lorke DE (2013) Extracellular matrix alterations, accelerated leukocyte infiltration and enhanced axonal sprouting after spinal cord hemisection in tenascin-C-deficient mice. Acta Histochem 115:865–878. doi: 10.1016/j.acthis.2013.04.009 23701962 10.1016/j.acthis.2013.04.009

[59] Roll P, Rudolf G, Pereira S, Royer B, Scheffer IE, Massacrier A et al (2006) SRPX2 mutations in disorders of language cortex and cognition. Hum Mol Genet 15:1195–1207. doi: 10.1093/hmg/ddl035 16497722 10.1093/hmg/ddl035

[60] Miljkovic-Licina M, Hammel P, Garrido-Urbani S, Bradfield PF, Szepetowski P, Imhof BA (2009) Sushi repeat protein X-linked 2, a novel mediator of angiogenesis. FASEB J 23:4105–4116. doi: 10.1096/fj.09-135202 19667118 10.1096/fj.09-135202

[61] Gao Z, Zhang J, Bi M, Han X, Han Z, Wang H et al (2015) SRPX2 promotes cell migration and invasion via FAK dependent pathway in pancreatic cancer. Int J Clin Exp Pathol 8:4791–4798. 26191169 PMC4503041

[62] Spalice A, Parisi P, Nicita F, Pizzardi G, DelBalzo P, Iannetti P (2009) Neuronal migration disorders: clinical, neuroradiologic and genetics aspects. Acta Pædiatrica 98: 421–433. doi: 10.1111/j.1651-2227.2008.01160.x 19120042 10.1111/j.1651-2227.2008.01160.x

[63] Kulinski JM, Munoz-Cano R and Olivera A (2016) Sphingosine-1-phosphate and other lipid mediators generated by mast cells as critical players in allergy and mast cell function. Eur J Pharmacol; 778: 56–67. doi: 10.1016/j.ejphar.2015.02.058 25941085 10.1016/j.ejphar.2015.02.058 PMC4630215

[64] Wang Z, Fan H, Xie R, Yang J, Ren Y, Yang Y and Li W (2015) The Effect of Sphingosine 1-Phosphate/Sphingosine 1-Phosphate Receptor on Neutrophil Function and the Relevant Signaling Pathway. Acta Haematol; 134: 49–56. doi: 10.1159/000369291 25872153 10.1159/000369291

[65] Oskeritzian CA (2015) Mast cell plasticity and sphingosine-1-phosphate in immunity, inflammation and cancer. Mol Immunol; 63: 104–112. doi: 10.1016/j.molimm.2014.03.018 24766823 10.1016/j.molimm.2014.03.018 PMC4226394

[66] Arlt O, Schwiebs A, Japtok L, Ruger K, Katzy E, Kleuser B and Radeke HH (2014) Sphingosine-1-phosphate modulates dendritic cell function: focus on non-migratory effects in vitro and in vivo. Cell Physiol Biochem; 34: 27–44. doi: 10.1159/000362982 24977479 10.1159/000362982

[67] Keul P, Lucke S, von Wnuck Lipinski K, Bode C, Graler M, Heusch G and Levkau B (2011) Sphingosine-1-phosphate receptor 3 promotes recruitment of monocyte/macrophages in inflammation and atherosclerosis. Circ Res; 108: 314–323. doi: 10.1161/CIRCRESAHA.110.235028 21164103 10.1161/CIRCRESAHA.110.235028

[68] Nayak D, Huo Y, Kwang WX, Pushparaj PN, Kumar SD, Ling EA and Dheen ST (2011) Sphingosine kinase 1 regulates the expression of proinflammatory cytokines and nitric oxide in activated microglia. Neuroscience; 166: 132–144 10.1016/j.neuroscience.2009.12.020 20036321

[69] Minami T, Nishihara I, Uda R, Ito S, Hyodo M, Hayaishi O (1994) Characterization of EP-receptor subtypes involved in allodynia and hyperalgesia induced by intrathecal administrationof prostaglandin E2 to mice. Br J Pharmacol 112:735–740. 7921597 10.1111/j.1476-5381.1994.tb13139.x PMC1910214

[70] Minami T, Okuda-Ashitaka E, Hori Y, Sakuma S, Sugimoto T, Sakimura K et al (1999) Involvement of primary afferent C-fibres in touch-evoked pain (allodynia) induced by prostaglandin E2. Eur J Neurosci 11:1849–1856. 10336652 10.1046/j.1460-9568.1999.00602.x

[71] Mabuchi T, Kojima H, Abe T, Takagi K, Sakurai M, Ohmiya Y et al (2004) Membrane-Associated prostaglandin E synthase-1 is required for neuropathic pain. Neuroreport 15:1395–1398. 15194860 10.1097/01.wnr.0000129372.89000.31

[72] Kunori S, Matsumara S, Okuda-Ashitaka E, Katano T, Audoly LP, Urade Y et al (2011) A novel role for prostaglandin E in neuropathic pain: blockade of microglial migration in the spinal cord. Glia 59:208–218. doi: 10.1002/glia.21090 21125641 10.1002/glia.21090

[73] Svensson CI, Lucas KK, Hua XY, Powell HC, Dennis EA, Yaksh TL (2005) Spinal phospholipase A2 in inflammatory hyperalgesia: role of the small, secretory phospholipase A2. Neuroscience 133:543–553. doi: 10.1016/j.neuroscience.2005.01.024 15885922 10.1016/j.neuroscience.2005.01.024

[74] Kuge H, Akahori K, Yagyu K-i, Honke K (2014) Functional compartimentalization of the plasma membrane of neurons by by a unique acyl chain composition of phospholipids. J Biol Chem 289:26783–26793. doi: 10.1074/jbc.M114.571075 25096572 10.1074/jbc.M114.571075 PMC4175321

[75] Liu NK, Titsworth WL, Zhang YP, Xhafa AI, Shields CB, Xu XM (2011) Characterizing phospholipase A2-induced spinal cord injury-a comparison with contusive spinal cord injury in adult rats. Transl Stroke Res 2:608–618. doi: 10.1007/s12975-011-0089-x 23585818 10.1007/s12975-011-0089-x PMC3622278

[76] Liu N, Han S, Lu PH, Xu XM (2004) Upregulation of annexins I, II and V after traumatic spinal cord injury in adult rats. J Neurosci Res 77:391–401. doi: 10.1002/jnr.20167 15248295 10.1002/jnr.20167

[77] Funari SS, Barceló F, Escribá PV (2003) Effects of oleic acid and its congeners, elaidic and stearic acids, on the structural properties of phosphatidylethanolamine membranes. J Lipid Res 44:567–575. doi: 10.1194/jlr.M200356-JLR200 12562874 10.1194/jlr.M200356-JLR200

[78] Yang Q, Alemany R, Casas J, Kitajka K, Lanier SM, Escribá PV (2005) Influence of the membrane lipid structure on signal processing via G protein-coupled receptors. Mol Pharmacol 68:210–217. doi: 10.1124/mol.105.011692 15837842 10.1124/mol.105.011692

[79] Ibarguren M, López DJ, Encinar JA, González-Ros JM, Busquets X, Escribá PV (2013) Partitioning of liquid-ordered/liquid-disordered membrane microdomains induced by the fluidifying effect of 2-hydroxylated fatty acid derivatives. Biochim Biophys Acta 1828:2553–2563. doi: 10.1016/j.bbamem.2013.06.014 23792066 10.1016/j.bbamem.2013.06.014

[80] Ibarguren M, López DJ, Escribá PV (2014) The effect of natural and synthetic fatty acids on membrane structure, microdomain organization, cellular functions and human health. Biochim Biophys Acta 1838:1518–1528. doi: 10.1016/j.bbamem.2013.12.021 24388951 10.1016/j.bbamem.2013.12.021

[81] Escribá PV, Ferrer-Montiel AV, Ferragut JA, Gonzalez-Ros JM (1990) Role of membrane lipids in the interaction of daunomycin with plasma membranes from tumor cells: implications in drug-resistance phenomena. Biochemistry 29:7275–7282. 2207106 10.1021/bi00483a017

[82] Escribá PV (2006) Membrane-lipid therapy: a new approach in molecular medicine. Trends Mol Med 12:34–43. doi: 10.1016/j.molmed.2005.11.004 16325472 10.1016/j.molmed.2005.11.004

[83] Escribá PV, Busquets X, Inokuchi J, Balogh G, Török Z, Horváth I et al (2015) Membrane lipid therapy: Modulation of the cell membrane composition and structure as a molecular base for drug discovery and new disease treatment. Prog Lipid Res 59:38–53. doi: 10.1016/j.plipres.2015.04.003 25969421 10.1016/j.plipres.2015.04.003

[84] Jasmin L, Ohara PT (2001) Long-term intrathecal catheterization in the rat. J Neurosci Methods 110:81–89. 11564527 10.1016/s0165-0270(01)00420-4

[85] Young W (2002) Spinal cord contusion models. Prog Brain Res 137:231–255. 12440371 10.1016/s0079-6123(02)37019-5

[86] Sarrion I, Milian L, Juan G, Ramon M, Furest I, Carda C et al (2015) Role of circulating miRNAs as biomarkers in idiopathic pulmonary arterial hypertension: possible relevance of miR-23a. Oxid Med Cell Longev 2015:792846 doi: 10.1155/2015/792846 25815108 10.1155/2015/792846 PMC4357130

[87] Mata M, Ruíz A, Cerdá M, Martinez-Losa M, Cortijo J, Santangelo F (2003) Oral N-acetylcysteine reduces bleomycin-induced lung damage and mucin Muc5ac expression in rats. Eur Respir J 22:900–905. 14680076 10.1183/09031936.03.00018003

[88] Li C, Wong WH (2001) Model-based analysis of oligonucleotide arrays: expression index computation and outlier detection. Proc Natl Acad Sci USA 98:31–36. doi: 10.1073/pnas.98.1.31 11134512 10.1073/pnas.011404098 PMC14539

[89] Mata M, Pallardo F, Morcillo EJ, Cortijo J (2012) Piclamilast inhibits the pro-apoptotic and anti-proliferative responses of A549 cells exposed to H_2_O_2_ via mechanisms involving AP-1 activation. Free Radic Res 46:690–699. doi: 10.3109/10715762.2012.669040 22360706 10.3109/10715762.2012.669040

