# Supplementary material for: Treatment with albumin-hydroxyoleic acid complex restores sensorimotor function in rats with spinal cord injury: Efficacy and gene expression regulation
Source: PLoS One. 2017 Dec 15;12(12):e0189151. doi: 10.1371/journal.pone.0189151 (PMC5731767; doi:10.1371/journal.pone.0189151)
Supplement: S1 Table — This table presents approximately 3,900 genes/transcripts that undergo changes over 2 fold when gene expression of SCI (7 days post injury) and non injured animals were compared. (DOCX) [file pone.0189151.s001.docx]

**Supporting information**

***S1 Table. Gene expression modulation in the lesion area of rats with SCI (7 days post trauma) compared with non-injured rats.***

| **Gene**  **Symbol** | **Gene Name** | **baseline mean** | **experiment mean** | **fold change** | **P value** |
| --- | --- | --- | --- | --- | --- |
| Ube2d3 | ubiquitin-conjugating enzyme E2D 3 (UBC4/5 homolog, yeast) | 1499.32 | 2505.97 | 1.67 | 0.000388 |
| Alb | albumin | 39.98 | 15.24 | -2.62 | 0.022100 |
| Ftl | ferritin, light polypeptide | 2833.41 | 6077.50 | 2.14 | 0.000018 |
| Rplp0 | ribosomal protein, large, P0 | 2499.32 | 4411.34 | 1.77 | 0.000633 |
| Rpsa | ribosomal protein SA | 1491.37 | 3071.42 | 2.06 | 0.000017 |
| Tagln | transgelin | 112.40 | 461.16 | 4.10 | 0.009167 |
| Vim | vimentin | 2753.29 | 7272.28 | 2.64 | 0.000022 |
| Gpx1 | glutathione peroxidase 1 | 1975.03 | 4633.45 | 2.35 | 0.000003 |
| Hspb1 | heat shock protein 1 | 1068.68 | 3698.02 | 3.46 | 0.000033 |
| Spp1 | secreted phosphoprotein 1 | 1200.65 | 4009.60 | 3.34 | 0.001179 |
| Rpl29 | ribosomal protein L29 | 2231.91 | 3681.84 | 1.65 | 0.000521 |
| Anxa2 | annexin A2 | 813.78 | 2693.97 | 3.31 | 0.001311 |
| Ldha | lactate dehydrogenase A | 521.92 | 1321.39 | 2.53 | 0.003091 |
| Bgn | biglycan | 71.64 | 278.73 | 3.89 | 0.000159 |
| B2m | beta-2 microglobulin | 3877.82 | 7643.72 | 1.97 | 0.000013 |
| Cited2 | Cbp/p300-interacting transactivator, with Glu/Asp-rich carboxy-terminal domain, 2 | 285.84 | 529.58 | 1.85 | 0.001197 |
| Pfn1 | profilin 1 | 371.76 | 1038.73 | 2.79 | 0.002579 |
| Mgst1 | microsomal glutathione S-transferase 1 | 1646.50 | 2779.76 | 1.69 | 0.000453 |
| Anxa1 | annexin A1 | 771.13 | 2986.50 | 3.87 | 0.000141 |
| Lgals1 | lectin, galactoside-binding, soluble, 1 | 874.93 | 2351.67 | 2.69 | 0.005260 |
| Glul | glutamate-ammonia ligase (glutamine synthetase) | 244.87 | 567.07 | 2.32 | 0.027084 |
| P4hb | prolyl 4-hydroxylase, beta polypeptide | 1458.48 | 3228.80 | 2.21 | 0.000442 |
| LOC100360710 | ribosomal protein S2-like /// ribosomal protein S2 | 2420.89 | 4876.88 | 2.01 | 0.000024 |
| Ctsd | cathepsin D | 733.20 | 2909.39 | 3.97 | 0.000001 |
| Tmsb10 | thymosin, beta 10 | 749.90 | 1833.89 | 2.45 | 0.014706 |
| Btg1 | B-cell translocation gene 1, anti-proliferative | 1485.92 | 3158.30 | 2.13 | 0.002373 |
| Fabp3 | fatty acid binding protein 3, muscle and heart | 360.63 | 210.45 | -1.71 | 0.000316 |
| S100a6 | S100 calcium binding protein A6 | 2344.67 | 4482.56 | 1.91 | 0.000287 |
| Psme1 | proteasome (prosome, macropain) activator subunit 1 | 1077.69 | 2951.78 | 2.74 | 0.000043 |
| Fdps | farnesyl diphosphate synthase (farnesyl pyrophosphate synthetase, dimethylallyltranstransferase, geranyltranstransferase) | 2238.88 | 1077.74 | -2.08 | 0.000081 |
| Cib1 | calcium and integrin binding 1 (calmyrin) | 408.59 | 1110.95 | 2.72 | 0.000131 |
| Hmgb2 | high mobility group box 2 | 436.09 | 1536.35 | 3.52 | 0.000166 |
| Cd151 | CD151 molecule (Raph blood group) | 1178.53 | 1905.55 | 1.62 | 0.000027 |
| Kpna2 | karyopherin alpha 2 | 341.84 | 985.08 | 2.88 | 0.000838 |
| Cd36 | CD36 molecule (thrombospondin receptor) | 13.46 | 169.78 | 12.62 | 0.005720 |
| Ssr4 | signal sequence receptor, delta | 1279.93 | 2395.30 | 1.87 | 0.000479 |
| Prkcdbp | protein kinase C, delta binding protein | 540.64 | 1083.20 | 2.00 | 0.000397 |
| Ifitm2 | interferon induced transmembrane protein 2 | 513.58 | 2666.99 | 5.19 | 0.000376 |
| sep-09 | septin 9 | 743.75 | 1327.19 | 1.78 | 0.000539 |
| Fmod | fibromodulin | 268.54 | 947.91 | 3.53 | 0.007645 |
| Fasn | fatty acid synthase | 885.06 | 522.40 | -1.69 | 0.002613 |
| Cd63 | Cd63 molecule | 2191.64 | 5159.59 | 2.35 | 0.000030 |
| Psme2 | proteasome (prosome, macropain) activator subunit 2 | 733.01 | 1479.13 | 2.02 | 0.000777 |
| Timp1 | TIMP metallopeptidase inhibitor 1 | 146.15 | 5357.97 | 36.66 | 0.000118 |
| Tnfrsf1a | tumor necrosis factor receptor superfamily, member 1a | 346.57 | 1141.92 | 3.29 | 0.000013 |
| Dpp7 | dipeptidylpeptidase 7 | 1405.05 | 2963.91 | 2.11 | 0.000797 |
| Sh2b3 | SH2B adaptor protein 3 | 196.02 | 612.58 | 3.13 | 0.000077 |
| Atp6v0e1 | ATPase, H+ transporting, lysosomal 9kDa, V0 subunit e1 | 687.88 | 1380.12 | 2.01 | 0.001020 |
| Gnb1 | guanine nucleotide binding protein (G protein), beta polypeptide 1 | 650.95 | 1357.87 | 2.09 | 0.000073 |
| Fuca1 | fucosidase, alpha-L- 1, tissue | 1128.28 | 2333.62 | 2.07 | 0.000006 |
| Lum | lumican | 1030.25 | 2648.25 | 2.57 | 0.002442 |
| Ccng1 | cyclin G1 | 1094.05 | 1899.82 | 1.74 | 0.000050 |
| Tcn2 | transcobalamin 2 | 419.19 | 1044.83 | 2.49 | 0.000619 |
| Cdk1 | cyclin-dependent kinase 1 | 30.75 | 606.60 | 19.72 | 0.001136 |
| Furin | furin (paired basic amino acid cleaving enzyme) | 226.64 | 484.19 | 2.14 | 0.000038 |
| Pttg1 | pituitary tumor-transforming 1 | 18.41 | 180.52 | 9.80 | 0.001701 |
| Clu | clusterin | 1970.68 | 3907.00 | 1.98 | 0.003780 |
| Psmb8 | proteasome (prosome, macropain) subunit, beta type 8 (large multifunctional peptidase 7) | 124.64 | 942.28 | 7.56 | 0.015366 |
| Ica1 | islet cell autoantigen 1 | 480.70 | 218.28 | -2.20 | 0.000034 |
| A2m | alpha-2-macroglobulin | 981.44 | 4910.52 | 5.00 | 0.002025 |
| Plat | plasminogen activator, tissue | 1137.57 | 2418.98 | 2.13 | 0.000057 |
| Sgk1 | serum/glucocorticoid regulated kinase 1 | 793.22 | 2187.57 | 2.76 | 0.001086 |
| Gls | glutaminase | 354.20 | 157.90 | -2.24 | 0.011031 |
| Plod1 | procollagen-lysine 1, 2-oxoglutarate 5-dioxygenase 1 | 454.77 | 821.37 | 1.81 | 0.005712 |
| Sptbn2 | spectrin, beta, non-erythrocytic 2 | 346.53 | 149.64 | -2.32 | 0.000638 |
| Nfe2l2 | nuclear factor, erythroid derived 2, like 2 | 673.03 | 1269.32 | 1.89 | 0.002742 |
| Tp53 | tumor protein p53 | 97.33 | 192.92 | 1.98 | 0.000040 |
| Psmc5 | proteasome (prosome, macropain) 26S subunit, ATPase, 5 | 2770.80 | 1627.48 | -1.70 | 0.000106 |
| Fdft1 | farnesyl diphosphate farnesyl transferase 1 | 1538.96 | 523.37 | -2.94 | 0.000074 |
| S100a4 | S100 calcium-binding protein A4 | 747.22 | 3325.28 | 4.45 | 0.000085 |
| Nupr1 | nuclear protein, transcriptional regulator, 1 | 703.10 | 1549.35 | 2.20 | 0.001566 |
| Sdc1 | syndecan 1 | 33.13 | 421.36 | 12.72 | 0.001009 |
| Fcgr2a | Fc fragment of IgG, low affinity IIa, receptor (CD32) /// Low affinity immunoglobulin gamma Fc region receptor III-like /// Fc gamma receptor II beta | 355.74 | 2681.88 | 7.54 | 0.000674 |
| Pfkm | phosphofructokinase, muscle | 1473.78 | 875.54 | -1.68 | 0.001143 |
| Sirpa | signal-regulatory protein alpha | 968.22 | 1739.25 | 1.80 | 0.000258 |
| Insig1 | insulin induced gene 1 | 2172.35 | 869.76 | -2.50 | 0.000027 |
| Gusb | glucuronidase, beta | 206.40 | 695.69 | 3.37 | 0.003454 |
| Enpp3 | ectonucleotide pyrophosphatase/phosphodiesterase 3 | 95.99 | 543.20 | 5.66 | 0.004302 |
| Dcxr | dicarbonyl L-xylulose reductase | 81.87 | 208.86 | 2.55 | 0.008659 |
| Ltbp1 | latent transforming growth factor beta binding protein 1 | 204.32 | 455.45 | 2.23 | 0.011395 |
| Emp3 | epithelial membrane protein 3 | 249.49 | 847.36 | 3.40 | 0.000811 |
| Plcd1 | phospholipase C, delta 1 | 96.47 | 172.20 | 1.79 | 0.000536 |
| Fez1 | fasciculation and elongation protein zeta 1 (zygin I) | 5047.82 | 2955.17 | -1.71 | 0.000254 |
| Mvp | major vault protein | 287.51 | 867.26 | 3.02 | 0.000105 |
| Ptbp1 | polypyrimidine tract binding protein 1 | 245.27 | 640.44 | 2.61 | 0.000805 |
| Hmgcs1 | 3-hydroxy-3-methylglutaryl-Coenzyme A synthase 1 (soluble) | 5331.21 | 2067.38 | -2.58 | 0.000053 |
| Stk10 | serine/threonine kinase 10 | 317.57 | 1273.13 | 4.01 | 0.000887 |
| Rbp1 | retinol binding protein 1, cellular | 228.67 | 1945.17 | 8.51 | 0.000292 |
| Cxcr7 | chemokine (C-X-C motif) receptor 7 | 298.12 | 670.88 | 2.25 | 0.005240 |
| Acp5 | acid phosphatase 5, tartrate resistant | 34.21 | 437.32 | 12.78 | 0.000007 |
| Chst10 | carbohydrate sulfotransferase 10 | 554.23 | 324.79 | -1.71 | 0.000503 |
| Atox1 | ATX1 antioxidant protein 1 homolog (yeast) | 765.22 | 1507.42 | 1.97 | 0.003118 |
| Ccl2 | chemokine (C-C motif) ligand 2 | 38.68 | 1981.46 | 51.22 | 0.016908 |
| Anxa3 | annexin A3 | 1862.22 | 4576.78 | 2.46 | 0.000007 |
| Anxa3 | annexin A3 | 198.08 | 491.60 | 2.48 | 0.001058 |
| Cyp51 | cytochrome P450, family 51 | 4885.72 | 2293.23 | -2.13 | 0.000851 |
| Scg5 | secretogranin V | 2969.73 | 1471.85 | -2.02 | 0.000420 |
| Slpi | secretory leukocyte peptidase inhibitor | 7.97 | 2034.46 | 255.25 | 0.013924 |
| C3 | complement component 3 | 756.78 | 3938.21 | 5.20 | 0.000324 |
| Aldh1a2 | aldehyde dehydrogenase 1 family, member A2 | 324.70 | 1041.77 | 3.21 | 0.000027 |
| Laptm5 | lysosomal protein transmembrane 5 | 313.83 | 1813.29 | 5.78 | 0.000543 |
| Ptpn6 | protein tyrosine phosphatase, non-receptor type 6 | 138.08 | 748.88 | 5.42 | 0.000115 |
| Tep1 | telomerase associated protein 1 | 253.54 | 680.00 | 2.68 | 0.000063 |
| Ptges | prostaglandin E synthase | 53.45 | 596.47 | 11.16 | 0.014772 |
| Mvd | mevalonate (diphospho) decarboxylase | 211.79 | 75.28 | -2.81 | 0.001203 |
| Tbxas1 | thromboxane A synthase 1, platelet | 94.52 | 637.18 | 6.74 | 0.000001 |
| Gnai3 | guanine nucleotide binding protein (G protein), alpha inhibiting 3 | 1230.90 | 2018.02 | 1.64 | 0.000261 |
| Chgb | chromogranin B | 2054.02 | 900.51 | -2.28 | 0.000201 |
| Slc31a1 | solute carrier family 31 (copper transporters), member 1 | 127.76 | 292.99 | 2.29 | 0.000335 |
| Ccnl1 | cyclin L1 | 715.62 | 1190.45 | 1.66 | 0.000115 |
| Lmna | lamin A | 83.30 | 158.22 | 1.90 | 0.007888 |
| Crym | crystallin, mu | 99.54 | 805.24 | 8.09 | 0.005020 |
| Ddc | dopa decarboxylase (aromatic L-amino acid decarboxylase) | 323.16 | 172.27 | -1.88 | 0.009915 |
| Irf1 | interferon regulatory factor 1 | 100.25 | 689.54 | 6.88 | 0.004919 |
| Lipa | lipase A, lysosomal acid, cholesterol esterase | 546.85 | 1448.79 | 2.65 | 0.000607 |
| Esm1 | endothelial cell-specific molecule 1 | 191.18 | 981.11 | 5.13 | 0.008695 |
| Lss | lanosterol synthase (2,3-oxidosqualene-lanosterol cyclase) | 62.68 | 20.86 | -3.01 | 0.035468 |
| Pde2a | phosphodiesterase 2A, cGMP-stimulated | 432.05 | 230.81 | -1.87 | 0.000508 |
| Oplah | 5-oxoprolinase (ATP-hydrolysing) | 130.32 | 505.78 | 3.88 | 0.000166 |
| Rab7l1 | RAB7, member RAS oncogene family-like 1 | 311.09 | 600.08 | 1.93 | 0.000511 |
| Abcg1 | ATP-binding cassette, sub-family G (WHITE), member 1 | 856.73 | 1485.97 | 1.73 | 0.000934 |
| Fgf13 | fibroblast growth factor 13 | 1206.18 | 530.15 | -2.28 | 0.000026 |
| Inpp5j | inositol polyphosphate-5-phosphatase J | 358.79 | 173.60 | -2.07 | 0.000404 |
| Nell1 | NEL-like 1 (chicken) | 559.74 | 213.19 | -2.63 | 0.000112 |
| Aacs | acetoacetyl-CoA synthetase | 298.88 | 125.75 | -2.38 | 0.002694 |
| Pla2g2a | phospholipase A2, group IIA (platelets, synovial fluid) | 13.95 | 594.04 | 42.60 | 0.024865 |
| Il4ra | interleukin 4 receptor, alpha | 68.39 | 222.90 | 3.26 | 0.000518 |
| Ninj2 | ninjurin 2 | 402.31 | 205.99 | -1.95 | 0.003322 |
| Anxa7 | annexin A7 | 293.34 | 638.72 | 2.18 | 0.000009 |
| Rgs2 | regulator of G-protein signaling 2 | 210.97 | 373.71 | 1.77 | 0.003619 |
| Pcp4 | Purkinje cell protein 4 | 2256.97 | 1252.34 | -1.80 | 0.000050 |
| Dusp1 | dual specificity phosphatase 1 | 404.47 | 901.48 | 2.23 | 0.000200 |
| Ngfr | nerve growth factor receptor (TNFR superfamily, member 16) | 27.64 | 68.70 | 2.49 | 0.010436 |
| Slc27a2 | solute carrier family 27 (fatty acid transporter), member 2 | 195.21 | 82.24 | -2.37 | 0.000279 |
| Camkk1 | calcium/calmodulin-dependent protein kinase kinase 1, alpha | 1028.38 | 544.53 | -1.89 | 0.000010 |
| Stmn3 | stathmin-like 3 | 3908.58 | 2158.90 | -1.81 | 0.000255 |
| Ctse | cathepsin E | 110.41 | 307.86 | 2.79 | 0.010573 |
| Lox | lysyl oxidase | 146.70 | 771.60 | 5.26 | 0.012620 |
| Acsl3 | acyl-CoA synthetase long-chain family member 3 | 1986.63 | 1198.62 | -1.66 | 0.000124 |
| Crlf2 | cytokine receptor-like factor 2 | 98.01 | 225.81 | 2.30 | 0.001397 |
| Acsl6 | acyl-CoA synthetase long-chain family member 6 | 1701.58 | 807.92 | -2.11 | 0.000211 |
|  | Rn.10943.1 | 252.81 | 105.50 | -2.40 | 0.000058 |
| Syk | spleen tyrosine kinase | 78.55 | 350.84 | 4.47 | 0.000003 |
| Gpnmb | glycoprotein (transmembrane) nmb | 263.97 | 6414.15 | 24.30 | 0.000158 |
| Hpd | 4-hydroxyphenylpyruvate dioxygenase | 8.75 | 29.50 | 3.37 | 0.024514 |
| Dhcr7 | 7-dehydrocholesterol reductase | 657.08 | 351.56 | -1.87 | 0.001772 |
| Ren | renin | 71.40 | 139.28 | 1.95 | 0.003165 |
| Cxcr3 | chemokine (C-X-C motif) receptor 3 | 31.49 | 69.78 | 2.22 | 0.012378 |
| Oprk1 | opioid receptor, kappa 1 | 123.59 | 40.84 | -3.03 | 0.000675 |
| Dab2 | disabled homolog 2 (Drosophila) | 421.64 | 1998.92 | 4.74 | 0.013469 |
| Cfi | complement factor I | 21.85 | 52.05 | 2.38 | 0.010643 |
| Fxyd5 | FXYD domain-containing ion transport regulator 5 | 496.17 | 1543.82 | 3.11 | 0.000353 |
| Tpp1 | tripeptidyl peptidase I | 1543.82 | 2817.05 | 1.82 | 0.000020 |
| Adamts1 | ADAM metallopeptidase with thrombospondin type 1 motif, 1 | 213.84 | 815.83 | 3.82 | 0.011963 |
| Slc28a2 | solute carrier family 28 (sodium-coupled nucleoside transporter), member 2 | 68.59 | 271.73 | 3.96 | 0.013051 |
| Mvk | mevalonate kinase | 442.64 | 240.14 | -1.84 | 0.001458 |
| Reg3b | regenerating islet-derived 3 beta | 26.38 | 74.73 | 2.83 | 0.005819 |
| Lrp3 | low density lipoprotein receptor-related protein 3 | 198.94 | 100.57 | -1.98 | 0.000344 |
| Prkcb | protein kinase C, beta | 280.74 | 136.55 | -2.06 | 0.000635 |
| Cds1 | CDP-diacylglycerol synthase 1 | 1086.58 | 508.37 | -2.14 | 0.000026 |
| Jak3 | Janus kinase 3 | 80.57 | 210.50 | 2.61 | 0.000709 |
| Serpini1 | serine (or cysteine) peptidase inhibitor, clade I, member 1 | 1244.27 | 583.21 | -2.13 | 0.002797 |
| Ptgs1 | prostaglandin-endoperoxide synthase 1 | 133.22 | 274.88 | 2.06 | 0.014493 |
| Aurkb | aurora kinase B | 109.09 | 482.70 | 4.42 | 0.002560 |
| Nrxn3 | neurexin 3 | 1409.25 | 605.27 | -2.33 | 0.000482 |
| Apobec1 | apolipoprotein B mRNA editing enzyme, catalytic polypeptide 1 | 25.96 | 1203.78 | 46.37 | 0.000060 |
| Fabp4 | fatty acid binding protein 4, adipocyte | 9.32 | 212.50 | 22.79 | 0.007067 |
| Got1 | glutamic-oxaloacetic transaminase 1, soluble (aspartate aminotransferase 1) | 1684.65 | 867.57 | -1.94 | 0.000208 |
| Sc4mol | sterol-C4-methyl oxidase-like | 4448.48 | 2123.73 | -2.09 | 0.001171 |
| Ctsc | cathepsin C | 42.81 | 821.32 | 19.19 | 0.003236 |
| Plvap | plasmalemma vesicle associated protein | 160.52 | 89.18 | -1.80 | 0.000636 |
| Chn1 | chimerin (chimaerin) 1 | 1291.24 | 647.26 | -1.99 | 0.001579 |
| Cyr61 | cysteine-rich, angiogenic inducer, 61 | 100.13 | 217.58 | 2.17 | 0.007380 |
| Birc2 | baculoviral IAP repeat-containing 2 | 330.87 | 626.29 | 1.89 | 0.000222 |
| Dnm1 | dynamin 1 | 1545.05 | 833.93 | -1.85 | 0.000092 |
| Slco2b1 | solute carrier organic anion transporter family, member 2b1 | 235.83 | 731.19 | 3.10 | 0.000008 |
| Casp6 | caspase 6 | 98.92 | 187.28 | 1.89 | 0.001687 |
| Ggt7 | gamma-glutamyltransferase 7 | 281.12 | 153.41 | -1.83 | 0.000679 |
| Myc | myelocytomatosis oncogene | 93.94 | 324.12 | 3.45 | 0.009481 |
| Ggcx | gamma-glutamyl carboxylase | 141.08 | 310.34 | 2.20 | 0.000158 |
| Entpd6 | ectonucleoside triphosphate diphosphohydrolase 6 | 35.06 | 198.90 | 5.67 | 0.007890 |
| Sod3 | superoxide dismutase 3, extracellular | 177.45 | 323.02 | 1.82 | 0.000386 |
| Brca2 | breast cancer 2 | 63.49 | 127.51 | 2.01 | 0.001385 |
| Slc12a9 | solute carrier family 12 (potassium/chloride transporters), member 9 | 66.81 | 130.12 | 1.95 | 0.001415 |
| Slc22a6 | solute carrier family 22 (organic anion transporter), member 6 | 76.97 | 37.67 | -2.04 | 0.006124 |
| Ctbs | chitobiase, di-N-acetyl- | 470.70 | 954.77 | 2.03 | 0.000785 |
| Gbp2 | guanylate binding protein 2 | 141.69 | 1509.38 | 10.65 | 0.006027 |
| Gad1 | glutamate decarboxylase 1 | 1202.27 | 561.42 | -2.14 | 0.000070 |
| Stx1b | syntaxin 1B | 481.97 | 217.14 | -2.22 | 0.000009 |
| Erap1 | endoplasmic reticulum aminopeptidase 1 | 714.22 | 1661.27 | 2.33 | 0.000363 |
| Ptpn2 | protein tyrosine phosphatase, non-receptor type 2 | 333.83 | 666.04 | 2.00 | 0.000025 |
| Asgr2 | asialoglycoprotein receptor 2 | 101.55 | 243.15 | 2.39 | 0.004670 |
| Adcy4 | adenylate cyclase 4 | 24.88 | 152.12 | 6.11 | 0.003223 |
| Rgs7 | regulator of G-protein signaling 7 | 948.47 | 414.93 | -2.29 | 0.000094 |
| Scarb2 | scavenger receptor class B, member 2 | 424.28 | 775.88 | 1.83 | 0.000414 |
| Crtac1 | cartilage acidic protein 1 | 264.83 | 112.15 | -2.36 | 0.000135 |
| S100a3 | S100 calcium binding protein A3 | 67.10 | 353.61 | 5.27 | 0.002360 |
| Cd93 | CD93 molecule | 54.87 | 178.40 | 3.25 | 0.020383 |
| Sfrp4 | secreted frizzled-related protein 4 | 148.23 | 415.85 | 2.81 | 0.002287 |
| Hpse | heparanase | 66.13 | 225.96 | 3.42 | 0.000010 |
| Map2 | microtubule-associated protein 2 | 426.44 | 149.23 | -2.86 | 0.009432 |
| Ptpro | protein tyrosine phosphatase, receptor type, O | 75.62 | 240.21 | 3.18 | 0.000015 |
| Cp | ceruloplasmin | 391.43 | 3278.14 | 8.37 | 0.000786 |
| Cp | ceruloplasmin | 756.28 | 3089.38 | 4.08 | 0.000281 |
| Cp | ceruloplasmin | 216.42 | 4147.23 | 19.16 | 0.000920 |
| Ikbkb | inhibitor of kappa light polypeptide gene enhancer in B-cells, kinase beta | 79.50 | 193.64 | 2.44 | 0.017081 |
| Caskin1 | CASK interacting protein 1 | 212.60 | 87.06 | -2.44 | 0.000158 |
| Lgmn | legumain | 1734.38 | 4766.90 | 2.75 | 0.000148 |
| Nat1 | N-acetyltransferase 1 (arylamine N-acetyltransferase) | 18.08 | 49.79 | 2.75 | 0.002658 |
| Slc3a1 | solute carrier family 3, member 1 | 302.33 | 153.80 | -1.97 | 0.008518 |
| Msln | mesothelin | 13.95 | 164.07 | 11.76 | 0.003398 |
| Ltbp2 | latent transforming growth factor beta binding protein 2 | 15.81 | 166.76 | 10.55 | 0.001681 |
| Slc2a5 | solute carrier family 2 (facilitated glucose/fructose transporter), member 5 | 116.15 | 414.59 | 3.57 | 0.000102 |
| Clec10a | C-type lectin domain family 10, member A | 122.42 | 350.11 | 2.86 | 0.031704 |
| Accn1 | amiloride-sensitive cation channel 1, neuronal | 283.47 | 136.86 | -2.07 | 0.000518 |
| Vcam1 | vascular cell adhesion molecule 1 | 684.77 | 1291.34 | 1.89 | 0.002178 |
| Bcl2a1d | B-cell leukemia/lymphoma 2 related protein A1d | 89.81 | 1143.46 | 12.73 | 0.000147 |
| Nfil3 | nuclear factor, interleukin 3 regulated | 43.40 | 107.39 | 2.47 | 0.001691 |
| Cd14 | CD14 molecule | 116.56 | 1552.77 | 13.32 | 0.011782 |
| Hpgds | hematopoietic prostaglandin D synthase | 15.84 | 174.31 | 11.00 | 0.002281 |
| Rgs4 | regulator of G-protein signaling 4 | 232.00 | 126.01 | -1.84 | 0.000533 |
| Epb4.1l3 | erythrocyte protein band 4.1-like 3 | 2459.70 | 1189.92 | -2.07 | 0.000016 |
| Cd53 | Cd53 molecule | 327.79 | 2254.43 | 6.88 | 0.000589 |
| Timeless | timeless homolog (Drosophila) | 57.48 | 145.39 | 2.53 | 0.005681 |
| Cadps | Ca++-dependent secretion activator | 2017.02 | 906.29 | -2.23 | 0.000046 |
| Kcnc1 | potassium voltage gated channel, Shaw-related subfamily, member 1 | 1434.02 | 604.21 | -2.37 | 0.000075 |
| Ptgs2 | prostaglandin-endoperoxide synthase 2 | 41.92 | 386.25 | 9.21 | 0.029610 |
| Mmp12 | matrix metallopeptidase 12 | 8.31 | 209.49 | 25.21 | 0.007026 |
| Emb | embigin homolog (mouse) | 290.83 | 793.26 | 2.73 | 0.004446 |
| Pnlip | pancreatic lipase | 182.82 | 644.38 | 3.52 | 0.007308 |
| Vegp1 | von Ebners gland protein 1 /// von Ebners gland protein 2 | 16.26 | 53.71 | 3.30 | 0.003271 |
| Aif1 | allograft inflammatory factor 1 | 692.95 | 3133.47 | 4.52 | 0.000486 |
| Kcnj5 | potassium inwardly-rectifying channel, subfamily J, member 5 | 46.37 | 90.91 | 1.96 | 0.001889 |
| Abcd2 | ATP-binding cassette, sub-family D (ALD), member 2 | 342.94 | 113.51 | -3.02 | 0.000009 |
| Sult4a1 | sulfotransferase family 4A, member 1 | 2956.12 | 1531.80 | -1.93 | 0.000039 |
| Slc17a6 | solute carrier family 17 (sodium-dependent inorganic phosphate cotransporter), member 6 | 2270.05 | 1075.61 | -2.11 | 0.000007 |
| Grin1 | glutamate receptor, ionotropic, N-methyl D-aspartate 1 | 163.96 | 89.56 | -1.83 | 0.004189 |
| Cplx2 | complexin 2 | 585.62 | 214.38 | -2.73 | 0.011906 |
| Apoc1 | apolipoprotein C-I | 90.18 | 631.65 | 7.00 | 0.001305 |
| Cd1d1 | CD1d1 molecule | 17.13 | 65.94 | 3.85 | 0.001616 |
| Mmp24 | matrix metallopeptidase 24 | 205.99 | 109.65 | -1.88 | 0.001277 |
| Sik1 | salt-inducible kinase 1 | 143.56 | 300.10 | 2.09 | 0.002498 |
| Mefv | Mediterranean fever | 27.89 | 181.61 | 6.51 | 0.001733 |
| Sh2b2 | SH2B adaptor protein 2 | 25.79 | 79.99 | 3.10 | 0.002568 |
| Mca32 | mast cell antigen 32 | 10.04 | 103.74 | 10.33 | 0.047746 |
| Grp | gastrin releasing peptide | 77.17 | 39.14 | -1.97 | 0.001875 |
| Mlxipl | MLX interacting protein-like | 50.86 | 138.11 | 2.72 | 0.003117 |
| Grb14 | growth factor receptor bound protein 14 | 491.68 | 227.22 | -2.16 | 0.000241 |
| Ddx25 | DEAD (Asp-Glu-Ala-Asp) box polypeptide 25 | 266.01 | 135.02 | -1.97 | 0.000305 |
| Card9 | caspase recruitment domain family, member 9 | 52.16 | 150.42 | 2.88 | 0.001794 |
| Wnt4 | wingless-type MMTV integration site family, member 4 | 142.45 | 349.55 | 2.45 | 0.036485 |
| Ptpn1 | protein tyrosine phosphatase, non-receptor type 1 | 113.14 | 316.78 | 2.80 | 0.000057 |
| Grk6 | G protein-coupled receptor kinase 6 | 75.13 | 162.66 | 2.17 | 0.003324 |
| Srgn | serglycin | 810.97 | 2040.06 | 2.52 | 0.000022 |
| Mmp3 | matrix metallopeptidase 3 | 13.16 | 61.32 | 4.66 | 0.019465 |
| Ucp2 | uncoupling protein 2 (mitochondrial, proton carrier) | 20.58 | 68.82 | 3.34 | 0.000822 |
| Srpx | sushi-repeat-containing protein, X-linked | 86.51 | 269.36 | 3.11 | 0.004352 |
| Pygl | phosphorylase, glycogen, liver | 65.65 | 534.49 | 8.14 | 0.000040 |
| Lyn | v-yes-1 Yamaguchi sarcoma viral related oncogene homolog | 21.37 | 105.74 | 4.95 | 0.012878 |
| Olr1 | oxidized low density lipoprotein (lectin-like) receptor 1 | 11.73 | 91.78 | 7.83 | 0.000996 |
| Grm4 | glutamate receptor, metabotropic 4 | 315.53 | 132.19 | -2.39 | 0.000060 |
| Gria3 | glutamate receptor, ionotrophic, AMPA 3 | 444.41 | 138.15 | -3.22 | 0.005882 |
| Fgr | Gardner-Rasheed feline sarcoma viral (v-fgr) oncogene homolog | 27.26 | 192.78 | 7.07 | 0.003266 |
| Fxyd7 | FXYD domain-containing ion transport regulator 7 | 981.92 | 573.09 | -1.71 | 0.001204 |
| Atp2b2 | ATPase, Ca++ transporting, plasma membrane 2 | 1389.55 | 563.67 | -2.47 | 0.000026 |
| Dscam | Down syndrome cell adhesion molecule /// Down syndrome cell adhesion molecule-like | 332.40 | 144.36 | -2.30 | 0.000449 |
| Pawr | PRKC, apoptosis, WT1, regulator | 95.81 | 215.46 | 2.25 | 0.013625 |
| Drd2 | dopamine receptor D2 | 94.38 | 42.09 | -2.24 | 0.002764 |
| Ppp1r14c | protein phosphatase 1, regulatory (inhibitor) subunit 14c | 30.43 | 7.34 | -4.15 | 0.003595 |
| Lat | linker for activation of T cells | 19.83 | 50.20 | 2.53 | 0.005849 |
| P2ry12 | purinergic receptor P2Y, G-protein coupled, 12 | 109.60 | 239.98 | 2.19 | 0.001620 |
| Tap2 | transporter 2, ATP-binding cassette, sub-family B (MDR/TAP) | 76.98 | 258.97 | 3.36 | 0.012835 |
| Trpv2 | transient receptor potential cation channel, subfamily V, member 2 | 98.77 | 191.67 | 1.94 | 0.014532 |
| P2rx6 | purinergic receptor P2X, ligand-gated ion channel, 6 | 287.24 | 123.71 | -2.32 | 0.000706 |
| C5ar1 | complement component 5a receptor 1 | 117.62 | 779.23 | 6.62 | 0.000004 |
| Kcns3 | potassium voltage-gated channel, delayed-rectifier, subfamily S, member 3 | 724.69 | 263.30 | -2.75 | 0.000032 |
| Tacr3 | tachykinin receptor 3 | 24.50 | 8.97 | -2.73 | 0.026382 |
| P2ry6 | pyrimidinergic receptor P2Y, G-protein coupled, 6 | 145.26 | 803.05 | 5.53 | 0.000518 |
| Cacng2 | calcium channel, voltage-dependent, gamma subunit 2 | 308.49 | 106.36 | -2.90 | 0.000221 |
| Cxcl2 | chemokine (C-X-C motif) ligand 2 | 11.41 | 200.84 | 17.60 | 0.018680 |
| Mmp7 | matrix metallopeptidase 7 | 18.74 | 76.92 | 4.11 | 0.001393 |
| Gcnt1 | glucosaminyl (N-acetyl) transferase 1, core 2 (beta-1,6-N-acetylglucosaminyltransferase) | 108.10 | 438.04 | 4.05 | 0.005215 |
| Sulf1 | sulfatase 1 | 25.66 | 93.98 | 3.66 | 0.000160 |
| Slc4a3 | solute carrier family 4 (anion exchanger), member 3 | 463.81 | 252.13 | -1.84 | 0.004669 |
| Icos | inducible T-cell co-stimulator | 18.62 | 65.29 | 3.51 | 0.001972 |
| Birc5 | baculoviral IAP repeat-containing 5 | 40.99 | 131.33 | 3.20 | 0.007038 |
| Cxxc4 | CXXC finger 4 | 217.39 | 57.11 | -3.81 | 0.003465 |
| Cap1 | CAP, adenylate cyclase-associated protein 1 (yeast) | 598.26 | 1018.97 | 1.70 | 0.001290 |
| Lmnb1 | lamin B1 | 23.11 | 72.52 | 3.14 | 0.007869 |
| Cebpd | CCAAT/enhancer binding protein (C/EBP), delta | 18.40 | 233.75 | 12.70 | 0.008457 |
| Psme4 | proteasome (prosome, macropain) activator subunit 4 | 356.04 | 695.60 | 1.95 | 0.025551 |
| Itgb1 | integrin, beta 1 | 1197.36 | 2162.20 | 1.81 | 0.003514 |
| Comt | catechol-O-methyltransferase | 686.48 | 2080.48 | 3.03 | 0.000666 |
| Fbn1 | fibrillin 1 | 380.20 | 1636.93 | 4.31 | 0.003306 |
| Stat1 | signal transducer and activator of transcription 1 /// signal transducer and activator of transcription 4 | 495.02 | 1173.01 | 2.37 | 0.002331 |
| Tpm4 | tropomyosin 4 | 93.39 | 407.46 | 4.36 | 0.007533 |
| Tmem176b | transmembrane protein 176B | 894.58 | 3838.70 | 4.29 | 0.000154 |
| Ets1 | v-ets erythroblastosis virus E26 oncogene homolog 1 (avian) | 297.05 | 645.92 | 2.17 | 0.007911 |
| Vsnl1 | visinin-like 1 | 1895.29 | 768.01 | -2.47 | 0.000523 |
| Vsnl1 | visinin-like 1 | 4582.51 | 2292.80 | -2.00 | 0.000015 |
| Jak2 | Janus kinase 2 | 99.26 | 267.40 | 2.69 | 0.001863 |
| Idi1 | isopentenyl-diphosphate delta isomerase 1 | 2028.92 | 782.75 | -2.59 | 0.000008 |
|  | Rn.17491.1 | 175.00 | 454.94 | 2.60 | 0.009609 |
|  | Rn.11397.1 | 4245.12 | 1968.63 | -2.16 | 0.010816 |
| Pak3 | p21 protein (Cdc42/Rac)-activated kinase 3 | 666.70 | 287.96 | -2.32 | 0.004236 |
| Anxa4 | annexin A4 | 239.98 | 508.56 | 2.12 | 0.000856 |
| Trh | thyrotropin releasing hormone | 36.53 | 271.42 | 7.43 | 0.000139 |
| Runx1 | runt-related transcription factor 1 | 162.74 | 487.21 | 2.99 | 0.003291 |
| Kmo | kynurenine 3-monooxygenase (kynurenine 3-hydroxylase) | 22.77 | 63.40 | 2.78 | 0.001360 |
| Cd44 | Cd44 molecule | 144.38 | 549.47 | 3.81 | 0.003529 |
| Esyt1 | extended synaptotagmin-like protein 1 | 60.41 | 122.92 | 2.03 | 0.002129 |
| Kcnn4 | potassium intermediate/small conductance calcium-activated channel, subfamily N, member 4 | 29.89 | 118.41 | 3.96 | 0.003316 |
| Adarb1 | adenosine deaminase, RNA-specific, B1 | 1321.97 | 465.82 | -2.84 | 0.011430 |
| Ntrk3 | neurotrophic tyrosine kinase, receptor, type 3 | 540.26 | 206.60 | -2.61 | 0.001556 |
| Gadd45a | growth arrest and DNA-damage-inducible, alpha | 203.70 | 606.90 | 2.98 | 0.000819 |
| Ebf1 | early B-cell factor 1 | 220.29 | 101.52 | -2.17 | 0.000671 |
| Pacsin1 | protein kinase C and casein kinase substrate in neurons 1 | 208.96 | 62.74 | -3.33 | 0.000556 |
| Mmp23 | matrix metallopeptidase 23 | 31.31 | 69.85 | 2.23 | 0.004541 |
| Lrrn3 | leucine rich repeat neuronal 3 | 268.22 | 142.10 | -1.89 | 0.000057 |
| Slc16a3 | solute carrier family 16, member 3 (monocarboxylic acid transporter 4) | 17.00 | 102.48 | 6.03 | 0.003752 |
| Ntrk2 | neurotrophic tyrosine kinase, receptor, type 2 | 581.56 | 221.99 | -2.62 | 0.001057 |
| Cd38 | CD38 molecule | 27.01 | 71.30 | 2.64 | 0.003002 |
| Cyp1b1 | cytochrome P450, family 1, subfamily b, polypeptide 1 | 94.13 | 967.57 | 10.28 | 0.004820 |
| Odz2 | odz, odd Oz/ten-m homolog 2 (Drosophila) | 118.79 | 46.43 | -2.56 | 0.000654 |
| Soat1 | sterol O-acyltransferase 1 | 3.35 | 24.56 | 7.32 | 0.002610 |
| Rab26 | RAB26, member RAS oncogene family | 357.58 | 177.78 | -2.01 | 0.000811 |
| Hk2 | hexokinase 2 | 9.67 | 28.26 | 2.92 | 0.015714 |
| Chek2 | CHK2 checkpoint homolog (S. pombe) | 46.81 | 108.39 | 2.32 | 0.015669 |
| Foxm1 | forkhead box M1 | 16.75 | 59.10 | 3.53 | 0.011105 |
| Cd5 | Cd5 molecule | 25.87 | 60.10 | 2.32 | 0.007621 |
| Plscr1 | phospholipid scramblase 1 | 31.18 | 315.54 | 10.12 | 0.002010 |
| Il18bp | interleukin 18 binding protein | 72.84 | 189.32 | 2.60 | 0.006131 |
| Kcnj6 | potassium inwardly-rectifying channel, subfamily J, member 6 | 360.87 | 157.43 | -2.29 | 0.000054 |
| Kcna4 | potassium voltage-gated channel, shaker-related subfamily, member 4 | 122.28 | 47.71 | -2.56 | 0.000475 |
| Syt6 | synaptotagmin VI | 47.80 | 14.54 | -3.29 | 0.016640 |
| S1pr1 | sphingosine-1-phosphate receptor 1 | 29.02 | 9.76 | -2.97 | 0.021187 |
| Asah1 | N-acylsphingosine amidohydrolase (acid ceramidase) 1 | 308.92 | 676.85 | 2.19 | 0.001949 |
| Nxph1 | neurexophilin 1 | 181.75 | 90.13 | -2.02 | 0.000501 |
| Prkg2 | protein kinase, cGMP-dependent, type II | 91.17 | 28.79 | -3.17 | 0.000661 |
| Gucy1b3 | guanylate cyclase 1, soluble, beta 3 | 741.44 | 394.92 | -1.88 | 0.004428 |
| Mapk10 | mitogen activated protein kinase 10 | 277.36 | 143.74 | -1.93 | 0.000494 |
| RT1-EC2 | RT1 class Ib, locus EC2 | 155.66 | 763.77 | 4.91 | 0.002921 |
| Grem1 | gremlin 1, cysteine knot superfamily, homolog (Xenopus laevis) | 66.32 | 22.98 | -2.89 | 0.041347 |
| Adrb2 | adrenergic, beta-2-, receptor, surface | 26.17 | 54.29 | 2.07 | 0.007619 |
| Kcnc3 | potassium voltage gated channel, Shaw-related subfamily, member 3 | 737.55 | 102.69 | -7.18 | 0.037598 |
| Kcnc3 | potassium voltage gated channel, Shaw-related subfamily, member 3 | 599.25 | 78.87 | -7.60 | 0.031703 |
| Dlk1 | delta-like 1 homolog (Drosophila) | 83.96 | 38.40 | -2.19 | 0.004719 |
| Cntn4 | contactin 4 | 103.84 | 53.35 | -1.95 | 0.002048 |
| Casr | calcium-sensing receptor | 69.03 | 196.04 | 2.84 | 0.029220 |
| Trpc3 | transient receptor potential cation channel, subfamily C, member 3 | 84.00 | 37.97 | -2.21 | 0.000478 |
| Mmp9 | matrix metallopeptidase 9 | 14.28 | 89.15 | 6.24 | 0.011841 |
| Cybb | cytochrome b-245, beta polypeptide | 85.04 | 192.73 | 2.27 | 0.020711 |
| Casp1 | caspase 1 | 113.55 | 648.00 | 5.71 | 0.000852 |
| Il6 | interleukin 6 | 3.57 | 60.25 | 16.89 | 0.005156 |
| Apaf1 | apoptotic peptidase activating factor 1 | 117.83 | 292.39 | 2.48 | 0.000028 |
| Nt5e | 5' nucleotidase, ecto | 116.09 | 351.53 | 3.03 | 0.002245 |
| Wif1 | Wnt inhibitory factor 1 | 156.77 | 84.25 | -1.86 | 0.000500 |
| Hck | hemopoietic cell kinase | 54.92 | 589.60 | 10.74 | 0.000585 |
| Scn1a | sodium channel, voltage-gated, type I, alpha | 2500.72 | 999.31 | -2.50 | 0.000003 |
| L1cam | L1 cell adhesion molecule | 494.14 | 228.40 | -2.16 | 0.000515 |
| Npy5r | neuropeptide Y receptor Y5 | 28.75 | 13.28 | -2.17 | 0.018528 |
| Casp8 | caspase 8 | 29.01 | 159.14 | 5.48 | 0.000226 |
| Atf3 | activating transcription factor 3 | 31.94 | 425.95 | 13.34 | 0.000099 |
| Ccr5 | chemokine (C-C motif) receptor 5 | 66.56 | 164.32 | 2.47 | 0.038669 |
| Bst1 | bone marrow stromal cell antigen 1 | 41.40 | 436.73 | 10.55 | 0.002871 |
| Klrd1 | killer cell lectin-like receptor, subfamily D, member 1 | 11.31 | 38.24 | 3.38 | 0.042468 |
| Fhl2 | four and a half LIM domains 2 | 105.53 | 295.51 | 2.80 | 0.012040 |
| Akap6 | A kinase (PRKA) anchor protein 6 | 59.92 | 22.55 | -2.66 | 0.012473 |
| Inpp4b | inositol polyphosphate-4-phosphatase, type II | 77.94 | 26.19 | -2.98 | 0.003787 |
| Hcrtr2 | hypocretin (orexin) receptor 2 | 202.82 | 72.79 | -2.79 | 0.000247 |
| Gria4 | glutamate receptor, ionotropic, AMPA4 | 108.21 | 31.65 | -3.42 | 0.000673 |
| Vav1 | vav 1 guanine nucleotide exchange factor | 39.95 | 325.70 | 8.15 | 0.000017 |
| Map3k8 | mitogen-activated protein kinase kinase kinase 8 | 39.73 | 285.24 | 7.18 | 0.001481 |
| Ttpa | tocopherol (alpha) transfer protein | 53.46 | 23.44 | -2.28 | 0.001900 |
| Htr2b | 5-hydroxytryptamine (serotonin) receptor 2B | 29.77 | 208.71 | 7.01 | 0.001990 |
| Spdya | speedy homolog A (Xenopus laevis) | 90.69 | 47.45 | -1.91 | 0.015976 |
| Wisp2 | WNT1 inducible signaling pathway protein 2 | 185.33 | 473.13 | 2.55 | 0.002946 |
| Kcnj4 | potassium inwardly-rectifying channel, subfamily J, member 4 | 5.50 | 28.38 | 5.16 | 0.046190 |
| Dao | D-amino-acid oxidase | 344.74 | 146.33 | -2.36 | 0.005691 |
| Kcnk1 | potassium channel, subfamily K, member 1 | 69.90 | 32.46 | -2.15 | 0.016327 |
| Samsn1 | SAM domain, SH3 domain and nuclear localization signals, 1 | 21.80 | 78.80 | 3.62 | 0.003632 |
| Cd47 | Cd47 molecule | 380.10 | 667.30 | 1.76 | 0.000848 |
| Sv2b | synaptic vesicle glycoprotein 2b | 253.93 | 106.41 | -2.39 | 0.009849 |
| LOC100365624 | myosin IC-like | 234.29 | 542.22 | 2.31 | 0.000136 |
| Cxcl12 | chemokine (C-X-C motif) ligand 12 (stromal cell-derived factor 1) | 622.52 | 2350.01 | 3.77 | 0.008963 |
| Thy1 | Thy-1 cell surface antigen | 118.76 | 45.46 | -2.61 | 0.001554 |
| Thy1 | Thy-1 cell surface antigen | 2740.09 | 1267.20 | -2.16 | 0.002159 |
| Scn2a1 | sodium channel, voltage-gated, type II, alpha 1 | 444.91 | 205.30 | -2.17 | 0.000192 |
| Il18 | interleukin 18 | 233.19 | 792.47 | 3.40 | 0.000163 |
| Alox5ap | arachidonate 5-lipoxygenase activating protein | 60.17 | 227.12 | 3.77 | 0.009512 |
| P2rx5 | purinergic receptor P2X, ligand-gated ion channel, 5 | 50.74 | 19.76 | -2.57 | 0.000890 |
| Cnr1 | cannabinoid receptor 1 (brain) | 998.01 | 459.73 | -2.17 | 0.000018 |
| Kcnab3 | potassium voltage-gated channel, shaker-related subfamily, beta member 3 | 44.01 | 21.22 | -2.07 | 0.011538 |
| Nsf | N-ethylmaleimide-sensitive factor | 262.59 | 104.91 | -2.50 | 0.001542 |
| Nsf | N-ethylmaleimide-sensitive factor | 2089.66 | 990.24 | -2.11 | 0.005835 |
| RragB | Ras-related GTP binding B | 709.23 | 348.74 | -2.03 | 0.002867 |
| Abcc3 | ATP-binding cassette, sub-family C (CFTR/MRP), member 3 | 54.59 | 133.11 | 2.44 | 0.000840 |
| Slc6a20 | solute carrier family 6 (proline IMINO transporter), member 20 | 119.78 | 404.28 | 3.38 | 0.012869 |
| Slc6a20 | solute carrier family 6 (proline IMINO transporter), member 20 | 670.94 | 2448.44 | 3.65 | 0.008262 |
| Nmu | neuromedin U | 92.65 | 48.01 | -1.93 | 0.002003 |
| Adap2 | ArfGAP with dual PH domains 2 | 122.15 | 476.58 | 3.90 | 0.007753 |
| Tapbp | TAP binding protein | 91.85 | 232.65 | 2.53 | 0.001551 |
| Kcnj3 | potassium inwardly-rectifying channel, subfamily J, member 3 | 21.80 | 7.27 | -3.00 | 0.029572 |
| P2rx4 | purinergic receptor P2X, ligand-gated ion channel 4 | 94.25 | 401.26 | 4.26 | 0.000379 |
| Trhr | thyrotropin releasing hormone receptor | 404.90 | 132.70 | -3.05 | 0.000064 |
| B3gat2 | beta-1,3-glucuronyltransferase 2 (glucuronosyltransferase S) | 74.22 | 38.96 | -1.91 | 0.003175 |
| Ptger2 | prostaglandin E receptor 2 (subtype EP2) | 11.40 | 36.26 | 3.18 | 0.000652 |
| Sstr1 | somatostatin receptor 1 | 236.46 | 108.98 | -2.17 | 0.000079 |
| Shank2 | SH3 and multiple ankyrin repeat domains 2 | 282.26 | 119.75 | -2.36 | 0.000104 |
| Grm7 | glutamate receptor, metabotropic 7 | 32.58 | 15.32 | -2.13 | 0.002594 |
| Jun | Jun oncogene | 871.56 | 1648.49 | 1.89 | 0.002373 |
| Abat | 4-aminobutyrate aminotransferase | 232.03 | 108.56 | -2.14 | 0.000755 |
| Ccl20 | chemokine (C-C motif) ligand 20 | 7.13 | 291.89 | 40.94 | 0.036242 |
| Ccl3 | chemokine (C-C motif) ligand 3 | 26.39 | 680.69 | 25.79 | 0.000761 |
| Prkch | protein kinase C, eta | 11.39 | 43.00 | 3.77 | 0.003680 |
| Cnr2 | cannabinoid receptor 2 (macrophage) | 8.11 | 33.54 | 4.14 | 0.021660 |
| F10 | coagulation factor X | 55.15 | 182.65 | 3.31 | 0.000293 |
| St8sia1 | ST8 alpha-N-acetyl-neuraminide alpha-2,8-sialyltransferase 1 | 17.10 | 6.25 | -2.74 | 0.039436 |
| Cd8a | CD8a molecule | 6.48 | 196.43 | 30.33 | 0.005512 |
| Gabrb3 | gamma-aminobutyric acid (GABA) A receptor, beta 3 | 124.43 | 37.97 | -3.28 | 0.001366 |
| Crhbp | corticotropin releasing hormone binding protein | 40.37 | 20.33 | -1.99 | 0.011687 |
| Tmem150a | transmembrane protein 150A | 208.34 | 451.16 | 2.17 | 0.000299 |
| Dap | death-associated protein | 334.86 | 835.89 | 2.50 | 0.000661 |
| Tgm2 | transglutaminase 2, C polypeptide | 566.00 | 2530.65 | 4.47 | 0.001958 |
| Ctsk | cathepsin K | 168.79 | 838.50 | 4.97 | 0.000478 |
| Cdk4 | cyclin-dependent kinase 4 | 343.53 | 688.50 | 2.00 | 0.000819 |
| Pabpc1 | poly(A) binding protein, cytoplasmic 1 | 3745.17 | 6407.05 | 1.71 | 0.000860 |
| Ifngr1 | interferon gamma receptor 1 | 820.62 | 2380.54 | 2.90 | 0.000066 |
| Zfp36l1 | zinc finger protein 36, C3H type-like 1 | 1340.80 | 3619.33 | 2.70 | 0.000626 |
| Coro1a | coronin, actin binding protein 1A | 350.56 | 1079.46 | 3.08 | 0.000044 |
| Vamp8 | vesicle-associated membrane protein 8 | 461.75 | 1251.10 | 2.71 | 0.000047 |
| Xdh | xanthine dehydrogenase | 150.11 | 628.44 | 4.19 | 0.003713 |
| Skap2 | src kinase associated phosphoprotein 2 | 421.92 | 1143.80 | 2.71 | 0.000439 |
| Ccl5 | chemokine (C-C motif) ligand 5 | 30.40 | 305.61 | 10.05 | 0.008970 |
| Sec11a | SEC11 homolog A (S. cerevisiae) | 695.35 | 1213.50 | 1.75 | 0.000645 |
| Camk2g | calcium/calmodulin-dependent protein kinase II gamma | 527.29 | 248.22 | -2.12 | 0.005131 |
| Arf6 | ADP-ribosylation factor 6 | 361.62 | 635.61 | 1.76 | 0.000522 |
| Nucb2 | nucleobindin 2 | 362.97 | 681.74 | 1.88 | 0.000314 |
| Rab8a | RAB8A, member RAS oncogene family | 307.52 | 571.50 | 1.86 | 0.000849 |
| Ptgis | prostaglandin I2 (prostacyclin) synthase | 54.74 | 133.94 | 2.45 | 0.014036 |
| Sult1a1 | sulfotransferase family, cytosolic, 1A, phenol-preferring, member 1 | 275.60 | 545.50 | 1.98 | 0.009287 |
| Gja4 | gap junction protein, alpha 4 | 39.29 | 133.44 | 3.40 | 0.008385 |
| Dlgap1 | Discs, large (Drosophila) homolog-associated protein 1 | 516.52 | 184.89 | -2.79 | 0.000346 |
| Cdkn2c | cyclin-dependent kinase inhibitor 2C (p18, inhibits CDK4) | 108.57 | 324.53 | 2.99 | 0.000431 |
| Ly6c | Ly6-C antigen | 47.46 | 157.38 | 3.32 | 0.007877 |
| Nefl | neurofilament, light polypeptide | 4049.67 | 2017.97 | -2.01 | 0.000130 |
| Slc12a5 | solute carrier family 12 (potassium-chloride transporter), member 5 | 3446.47 | 1710.30 | -2.02 | 0.000223 |
| Ada | adenosine deaminase | 96.33 | 240.98 | 2.50 | 0.000156 |
| Lin7b | lin-7 homolog b (C. elegans) | 367.50 | 121.11 | -3.03 | 0.000033 |
| Hmox1 | heme oxygenase (decycling) 1 | 327.16 | 2148.08 | 6.57 | 0.001143 |
| Ccr1 | chemokine (C-C motif) receptor 1 | 31.21 | 100.90 | 3.23 | 0.008518 |
| Ppargc1a | peroxisome proliferator-activated receptor gamma, coactivator 1 alpha | 159.32 | 36.81 | -4.33 | 0.000841 |
| Lcp2 | lymphocyte cytosolic protein 2 | 41.56 | 313.02 | 7.53 | 0.000425 |
| Prf1 | perforin 1 (pore forming protein) | 7.38 | 27.27 | 3.70 | 0.015229 |
| Cxcr4 | chemokine (C-X-C motif) receptor 4 | 40.38 | 228.21 | 5.65 | 0.000004 |
| Hcn1 | hyperpolarization-activated cyclic nucleotide-gated potassium channel 1 | 173.47 | 75.48 | -2.30 | 0.000480 |
| Fgf18 | fibroblast growth factor 18 | 60.64 | 27.47 | -2.21 | 0.008035 |
| Lin7a | lin-7 homolog a (C. elegans) | 71.28 | 32.52 | -2.19 | 0.001169 |
| Kcnk4 | potassium channel, subfamily K, member 4 | 103.22 | 34.08 | -3.03 | 0.001473 |
| Birc3 | baculoviral IAP repeat-containing 3 | 35.35 | 238.99 | 6.76 | 0.002429 |
| Slc7a10 | solute carrier family 7, (neutral amino acid transporter, y+ system) member 10 | 414.36 | 161.85 | -2.56 | 0.000357 |
| sep-03 | septin 3 | 37.84 | 15.39 | -2.46 | 0.018640 |
| Lst1 | leukocyte specific transcript 1 | 48.46 | 324.79 | 6.70 | 0.000022 |
| LOC363410 | ras-related protein Rab-27B-like /// RAB27B, member RAS oncogene family | 106.62 | 46.26 | -2.30 | 0.006681 |
| Pold1 | polymerase (DNA directed), delta 1, catalytic subunit | 29.27 | 64.53 | 2.20 | 0.040692 |
| Trpc6 | transient receptor potential cation channel, subfamily C, member 6 | 51.91 | 174.68 | 3.37 | 0.000313 |
|  | Rn.44219.1 | 78.29 | 271.45 | 3.47 | 0.001261 |
| Lyz2 | lysozyme 2 | 1795.35 | 6244.31 | 3.48 | 0.000048 |
| Col1a2 | collagen, type I, alpha 2 | 1153.76 | 2594.11 | 2.25 | 0.012224 |
| Myh10 | myosin, heavy chain 10, non-muscle | 1677.11 | 957.07 | -1.75 | 0.000669 |
| Smarcd2 | SWI/SNF related, matrix associated, actin dependent regulator of chromatin, subfamily d, member 2 | 279.62 | 519.73 | 1.86 | 0.001707 |
| Ppp4r1 | protein phosphatase 4, regulatory subunit 1 | 163.58 | 313.80 | 1.92 | 0.000809 |
| Sod2 | superoxide dismutase 2, mitochondrial | 649.43 | 2193.73 | 3.38 | 0.009667 |
| Trak2 | trafficking protein, kinesin binding 2 | 3335.40 | 1316.86 | -2.53 | 0.008068 |
| PVR | poliovirus receptor | 20.73 | 116.25 | 5.61 | 0.002727 |
| Cacnb2 | calcium channel, voltage-dependent, beta 2 subunit | 181.25 | 97.89 | -1.85 | 0.000701 |
| Ptprn2 | protein tyrosine phosphatase, receptor type, N polypeptide 2 | 188.22 | 99.10 | -1.90 | 0.004897 |
| Dyrk1a | dual-specificity tyrosine-(Y)-phosphorylation regulated kinase 1A | 42.29 | 91.22 | 2.16 | 0.004060 |
| Cntnap1 | contactin associated protein 1 | 262.39 | 133.84 | -1.96 | 0.003333 |
| Psmb9 | proteasome (prosome, macropain) subunit, beta type 9 (large multifunctional peptidase 2) | 331.30 | 1469.79 | 4.44 | 0.002524 |
| Snap23 | synaptosomal-associated protein 23 | 319.52 | 559.83 | 1.75 | 0.000998 |
| Pvalb | parvalbumin | 237.74 | 90.65 | -2.62 | 0.048449 |
| C1qb | complement component 1, q subcomponent, B chain | 182.68 | 2384.68 | 13.05 | 0.007157 |
| Cyba | cytochrome b-245, alpha polypeptide | 273.36 | 1746.32 | 6.39 | 0.000611 |
| Scpep1 | serine carboxypeptidase 1 | 822.39 | 2541.51 | 3.09 | 0.000166 |
| Cstb | cystatin B (stefin B) | 1797.32 | 2993.23 | 1.67 | 0.000100 |
| Fn1 | fibronectin 1 | 1296.95 | 4202.40 | 3.24 | 0.000276 |
| Ctsl1 | cathepsin L1 | 2678.12 | 5532.05 | 2.07 | 0.000029 |
|  | Rn.1294.1 | 656.77 | 1233.58 | 1.88 | 0.002122 |
| Tspo | translocator protein | 307.73 | 1814.84 | 5.90 | 0.000213 |
| Ube2i | ubiquitin-conjugating enzyme E2I (UBC9 homolog, yeast) | 447.41 | 921.61 | 2.06 | 0.000207 |
| Fzd1 | frizzled homolog 1 (Drosophila) | 175.18 | 437.79 | 2.50 | 0.001151 |
|  | Rn.41556.1 | 29.55 | 70.75 | 2.39 | 0.006414 |
| Tubb5 | tubulin, beta 5 | 346.76 | 1426.84 | 4.11 | 0.015952 |
| Cdc20 | cell division cycle 20 homolog (S. cerevisiae) | 34.50 | 164.96 | 4.78 | 0.001964 |
| Plk1 | polo-like kinase 1 (Drosophila) | 66.12 | 160.79 | 2.43 | 0.012518 |
| Mmp2 | matrix metallopeptidase 2 | 127.29 | 494.40 | 3.88 | 0.000103 |
| Unc13d | unc-13 homolog D (C. elegans) | 12.85 | 33.75 | 2.63 | 0.026150 |
| Ccnb1 | cyclin B1 | 30.23 | 364.88 | 12.07 | 0.002048 |
| Ccnb1 | cyclin B1 | 80.04 | 186.68 | 2.33 | 0.007535 |
| Pdlim7 | PDZ and LIM domain 7 | 54.29 | 182.90 | 3.37 | 0.000351 |
| Ninj1 | ninjurin 1 | 144.82 | 739.87 | 5.11 | 0.000037 |
| Scd1 | stearoyl-Coenzyme A desaturase 1 | 1136.80 | 394.18 | -2.88 | 0.000064 |
| Ceacam1 | carcinoembryonic antigen-related cell adhesion molecule 1 (biliary glycoprotein) /// carcinoembryonic antigen-related cell adhesion molecule 10 | 91.28 | 1386.49 | 15.19 | 0.016720 |
| Rasd2 | RASD family, member 2 | 112.11 | 43.88 | -2.55 | 0.000135 |
| Gls2 | glutaminase 2 (liver, mitochondrial) | 585.82 | 206.05 | -2.84 | 0.000037 |
| Cyp2d1 | cytochrome P450, family 2, subfamily d, polypeptide 1 /// cytochrome P450, family 2, subfamily d, polypeptide 5 | 6.92 | 195.66 | 28.28 | 0.019884 |
| Pnrc1 | proline-rich nuclear receptor coactivator 1 | 618.84 | 1119.88 | 1.81 | 0.000824 |
| Prlr | prolactin receptor | 74.35 | 33.65 | -2.21 | 0.001480 |
| Slc9a5 | solute carrier family 9 (sodium/hydrogen exchanger), member 5 | 72.82 | 37.68 | -1.93 | 0.007770 |
| Crabp2 | cellular retinoic acid binding protein 2 | 284.88 | 1171.80 | 4.11 | 0.010182 |
| RT1-N3 | RT1 class Ib, locus N3 | 33.60 | 123.39 | 3.67 | 0.017677 |
| Cd55 | Cd55 molecule | 22.55 | 84.90 | 3.77 | 0.023484 |
| Nid67 | putative small membrane protein NID67 | 270.24 | 787.40 | 2.91 | 0.000005 |
| Tnnt1 | troponin T type 1 (skeletal, slow) | 36.22 | 151.31 | 4.18 | 0.004580 |
| Ripk3 | receptor-interacting serine-threonine kinase 3 | 73.00 | 357.84 | 4.90 | 0.002183 |
| Gna15 | guanine nucleotide binding protein, alpha 15 | 32.98 | 109.46 | 3.32 | 0.000257 |
| Syn2 | synapsin II | 453.40 | 242.60 | -1.87 | 0.000343 |
| Hsd3b7 | hydroxy-delta-5-steroid dehydrogenase, 3 beta- and steroid delta-isomerase 7 | 142.07 | 273.70 | 1.93 | 0.000514 |
| Nos1ap | nitric oxide synthase 1 (neuronal) adaptor protein | 164.29 | 75.93 | -2.16 | 0.003951 |
| Kcnc2 | potassium voltage gated channel, Shaw-related subfamily, member 2 | 784.30 | 345.00 | -2.27 | 0.000033 |
| Dnase2a | deoxyribonuclease II alpha | 101.11 | 631.82 | 6.25 | 0.001119 |
| Cacna1b | calcium channel, voltage-dependent, N type, alpha 1B subunit | 429.85 | 193.17 | -2.23 | 0.000041 |
| Pla1a | phospholipase A1 member A | 73.89 | 826.48 | 11.18 | 0.004211 |
| P2ry14 | purinergic receptor P2Y, G-protein coupled, 14 | 50.05 | 200.07 | 4.00 | 0.000299 |
| Tpm3 | tropomyosin 3, gamma | 280.62 | 152.27 | -1.84 | 0.000437 |
| Olfm3 | olfactomedin 3 | 668.90 | 253.50 | -2.64 | 0.000009 |
| Hmmr | hyaluronan mediated motility receptor (RHAMM) | 26.47 | 75.88 | 2.87 | 0.012056 |
| RT1-CE16 | RT1 class I, locus CE16 | 312.99 | 1194.22 | 3.82 | 0.023010 |
| Lhx5 | LIM homeobox 5 | 21.23 | 8.46 | -2.51 | 0.016269 |
| Ptpn7 | protein tyrosine phosphatase, non-receptor type 7 | 21.14 | 79.04 | 3.74 | 0.002746 |
| Thrb | thyroid hormone receptor beta | 97.79 | 37.44 | -2.61 | 0.020378 |
| Cd244 | Cd244 molecule, natural killer cell receptor 2B4 /// similar to transmembrane NK cell receptor 2B4 /// similar to transmembrane NK cell receptor 2B4 | 30.38 | 108.71 | 3.58 | 0.000206 |
| Lilrb3l | leukocyte immunoglobulin-like receptor, subfamily B (with TM and ITIM domains), member 3-like /// similar to paired-Ig-like receptor B /// similar to paired-Ig-like receptor A11 | 8.75 | 69.29 | 7.92 | 0.017781 |
| Klrb1a | killer cell lectin-like receptor subfamily B, member 1A | 5.44 | 21.03 | 3.86 | 0.017399 |
| Mobp | myelin-associated oligodendrocyte basic protein | 1533.77 | 587.93 | -2.61 | 0.029500 |
| Epb4.1l3 | erythrocyte protein band 4.1-like 3 | 1289.78 | 513.77 | -2.51 | 0.000425 |
| Slc15a3 | solute carrier family 15, member 3 | 47.26 | 327.54 | 6.93 | 0.002008 |
| Nptx1 | neuronal pentraxin 1 | 1874.60 | 872.41 | -2.15 | 0.000146 |
| Gcgr | glucagon receptor | 2.34 | 21.52 | 9.20 | 0.021510 |
| Pld1 | phospholipase D1 | 357.66 | 752.36 | 2.10 | 0.000104 |
| Pld1 | phospholipase D1 | 223.28 | 475.21 | 2.13 | 0.000053 |
| Acvr1c | activin A receptor, type IC | 40.44 | 4.43 | -9.13 | 0.016778 |
| Myt1l | myelin transcription factor 1-like | 500.59 | 178.56 | -2.80 | 0.000273 |
| Kcna1 | potassium voltage-gated channel, shaker-related subfamily, member 1 | 1685.94 | 699.98 | -2.41 | 0.000908 |
| Unc13c | unc-13 homolog C (C. elegans) | 567.90 | 219.40 | -2.59 | 0.000088 |
| Kcnc2 | potassium voltage gated channel, Shaw-related subfamily, member 2 | 69.54 | 25.14 | -2.77 | 0.011264 |
| A3galt2 | alpha 1,3-galactosyltransferase 2 | 20.35 | 76.13 | 3.74 | 0.001953 |
| Fgf14 | fibroblast growth factor 14 | 94.56 | 43.91 | -2.15 | 0.000757 |
| Grin3a | glutamate receptor, ionotropic, N-methyl-D-aspartate 3A | 208.03 | 44.54 | -4.67 | 0.000241 |
| Ptprc | protein tyrosine phosphatase, receptor type, C | 13.80 | 152.99 | 11.09 | 0.004743 |
| Nrg1 | neuregulin 1 | 246.82 | 85.40 | -2.89 | 0.005486 |
| Slc16a7 | solute carrier family 16, member 7 (monocarboxylic acid transporter 2) | 34.13 | 120.78 | 3.54 | 0.005104 |
| Ugt1a1 | UDP glucuronosyltransferase 1 family, polypeptide A1 /// UDP glucuronosyltransferase 1 family, polypeptide A2 /// UDP glycosyltransferase 1 family, polypeptide A3 /// UDP glucuronosyltransferase 1 family, polypeptide A5 /// UDP glucuronosyltransferase 1 family, polypeptide A6 /// UDP glucuronosyltransferase 1 family, polypeptide A7C /// UDP glycosyltransferase 1 family, polypeptide A8 /// UDP glucuronosyltransferase 1 family, polypeptide A9 | 331.55 | 1352.55 | 4.08 | 0.000178 |
| Fgl2 | fibrinogen-like 2 | 75.95 | 215.76 | 2.84 | 0.003482 |
| Cdh6 | cadherin 6 | 206.09 | 87.02 | -2.37 | 0.004539 |
| Dnm3 | dynamin 3 | 164.52 | 47.27 | -3.48 | 0.000404 |
| Stau2 | staufen, RNA binding protein, homolog 2 (Drosophila) | 548.67 | 193.59 | -2.83 | 0.000479 |
| Lilrb3l | leukocyte immunoglobulin-like receptor, subfamily B (with TM and ITIM domains), member 3-like | 38.59 | 391.67 | 10.15 | 0.001043 |
| Egfr | epidermal growth factor receptor /// peptidase D | 1195.74 | 2283.87 | 1.91 | 0.000025 |
| Pcyt1b | phosphate cytidylyltransferase 1, choline, beta | 37.77 | 16.91 | -2.23 | 0.012518 |
| Klra5 | killer cell lectin-like receptor, subfamily A, member 5 | 2.96 | 92.53 | 31.27 | 0.000007 |
| Fgf9 | fibroblast growth factor 9 | 380.13 | 99.05 | -3.84 | 0.000002 |
| Mapre1 | microtubule-associated protein, RP/EB family, member 1 | 69.83 | 143.79 | 2.06 | 0.002720 |
| Gabarap | GABA(A) receptor-associated protein | 2125.52 | 3633.36 | 1.71 | 0.000070 |
| Ccl4 | chemokine (C-C motif) ligand 4 | 30.44 | 340.93 | 11.20 | 0.001643 |
| Pex5l | peroxisomal biogenesis factor 5-like | 605.99 | 96.72 | -6.27 | 0.015086 |
| Entpd2 | ectonucleoside triphosphate diphosphohydrolase 2 | 291.52 | 741.01 | 2.54 | 0.000321 |
| Camk2n1 | calcium/calmodulin-dependent protein kinase II inhibitor 1 | 472.35 | 236.05 | -2.00 | 0.001942 |
| Nexn | nexilin (F actin binding protein) | 157.59 | 65.48 | -2.41 | 0.001775 |
| Acta2 | smooth muscle alpha-actin | 31.08 | 78.59 | 2.53 | 0.011993 |
| Apoe | apolipoprotein E | 3195.03 | 6576.37 | 2.06 | 0.000048 |
| Col1a1 | collagen, type I, alpha 1 | 454.82 | 1503.82 | 3.31 | 0.013007 |
| Hnrnpa3 | heterogeneous nuclear ribonucleoprotein A3 | 1733.69 | 2965.34 | 1.71 | 0.003276 |
| Ezr | ezrin | 408.43 | 899.95 | 2.20 | 0.000488 |
| LOC100360501 | ribonuclease inhibitor-like /// ribonuclease/angiogenin inhibitor 1 | 444.23 | 792.78 | 1.78 | 0.003515 |
| RT1-DMb | RT1 class II, locus DMb | 361.43 | 1665.85 | 4.61 | 0.000056 |
| RT1-Da | RT1 class II, locus Da | 607.60 | 1763.98 | 2.90 | 0.047996 |
| Ctsz | cathepsin Z | 350.73 | 2230.23 | 6.36 | 0.000395 |
| Cd48 | Cd48 molecule | 1137.32 | 2669.83 | 2.35 | 0.000761 |
| C4-2 | complement component 4, gene 2 /// complement component 4B (Chido blood group) | 196.03 | 1054.24 | 5.38 | 0.000681 |
| Col5a2 | collagen, type V, alpha 2 | 547.62 | 1295.16 | 2.37 | 0.035942 |
| Akr1b8 | aldo-keto reductase family 1, member B8 | 31.70 | 183.32 | 5.78 | 0.002604 |
| LOC679885 | similar to N-myc downstream regulated gene 3 /// N-myc downstream regulated gene 3 | 1875.54 | 1070.43 | -1.75 | 0.000071 |
| RT1-DMa | RT1 class II, locus DMa | 140.47 | 678.68 | 4.83 | 0.003158 |
| Tec | tec protein tyrosine kinase | 96.62 | 260.45 | 2.70 | 0.000143 |
| Nme6 | non-metastatic cells 6, protein expressed in (nucleoside-diphosphate kinase) | 44.78 | 104.62 | 2.34 | 0.003071 |
| Tcrb | T-cell receptor beta chain | 6.22 | 79.20 | 12.74 | 0.013690 |
| Atp9a | ATPase, class II, type 9A /// ATPase, class II, type 9B | 2286.39 | 1382.73 | -1.65 | 0.000132 |
| Litaf | lipopolysaccharide-induced TNF factor | 1385.46 | 2433.91 | 1.76 | 0.000456 |
| Lrp4 | low density lipoprotein receptor-related protein 4 | 461.31 | 833.55 | 1.81 | 0.001865 |
| Pdxp | pyridoxal (pyridoxine, vitamin B6) phosphatase | 556.43 | 265.54 | -2.10 | 0.000136 |
| Marcks | myristoylated alanine rich protein kinase C substrate | 810.67 | 2069.37 | 2.55 | 0.000030 |
| Col3a1 | collagen, type III, alpha 1 | 1267.89 | 4845.11 | 3.82 | 0.002379 |
| Gas7 | growth arrest specific 7 | 113.01 | 463.34 | 4.10 | 0.007335 |
| RGD1564318 | similar to immunoglobulin light chain variable region | 19.01 | 62.22 | 3.27 | 0.022523 |
| Nfkb1 | nuclear factor of kappa light polypeptide gene enhancer in B-cells 1 | 274.15 | 688.90 | 2.51 | 0.001573 |
| Kcnj14 | potassium inwardly-rectifying channel, subfamily J, member 14 | 399.20 | 240.30 | -1.66 | 0.000352 |
| RT1-CE5 | RT1 class I, locus CE5 | 39.63 | 141.38 | 3.57 | 0.012238 |
| Pygm | phosphorylase, glycogen, muscle | 134.13 | 76.39 | -1.76 | 0.002890 |
| Rasgrf1 | RAS protein-specific guanine nucleotide-releasing factor 1 | 3085.63 | 1868.41 | -1.65 | 0.000085 |
| Spag5 | sperm associated antigen 5 | 328.48 | 164.11 | -2.00 | 0.000343 |
| Map1b | microtubule-associated protein 1B | 220.88 | 65.95 | -3.35 | 0.021938 |
| Epha5 | EphA5 | 287.70 | 119.21 | -2.41 | 0.004076 |
| Mx1 | myxovirus (influenza virus) resistance 1 | 123.38 | 694.35 | 5.63 | 0.000013 |
|  | Rn.10427.1 | 33.32 | 70.30 | 2.11 | 0.019688 |
| Arsb | arylsulfatase B | 72.62 | 237.75 | 3.27 | 0.009278 |
| Pros1 | protein S (alpha) | 347.56 | 1023.40 | 2.94 | 0.004751 |
| Cebpg | CCAAT/enhancer binding protein (C/EBP), gamma | 54.61 | 145.30 | 2.66 | 0.000390 |
| Pon1 | paraoxonase 1 | 11.90 | 64.47 | 5.42 | 0.000231 |
| Gabra5 | gamma-aminobutyric acid (GABA) A receptor, alpha 5 | 876.93 | 322.20 | -2.72 | 0.000135 |
| Pcdha13 | protocadherin alpha 13 | 353.04 | 114.55 | -3.08 | 0.009454 |
| B4galt1 | UDP-Gal:betaGlcNAc beta 1,4- galactosyltransferase, polypeptide 1 | 32.60 | 232.39 | 7.13 | 0.000955 |
| Mcm6 | minichromosome maintenance complex component 6 | 85.93 | 335.07 | 3.90 | 0.001781 |
| RT1-EC2 | RT1 class Ib, locus EC2 | 65.74 | 166.22 | 2.53 | 0.000215 |
| Fcgr2a | Fc fragment of IgG, low affinity IIa, receptor (CD32) /// Fc fragment of IgG, low affinity IIb, receptor (CD32) | 208.31 | 2145.89 | 10.30 | 0.002587 |
| Rapgef4 | Rap guanine nucleotide exchange factor (GEF) 4 | 849.31 | 371.64 | -2.29 | 0.008689 |
| Ptprf | protein tyrosine phosphatase, receptor type, F | 185.97 | 65.41 | -2.84 | 0.002097 |
| LOC360231 | MHC class I RT1.O type 149 processed pseudogene | 73.94 | 453.43 | 6.13 | 0.012003 |
| RT1-S3 | RT1 class Ib, locus S3 | 120.77 | 444.84 | 3.68 | 0.003463 |
| Txnip | thioredoxin interacting protein | 1209.88 | 2182.12 | 1.80 | 0.006776 |
| Ank3 | ankyrin 3, epithelial | 588.49 | 296.19 | -1.99 | 0.007083 |
| Prkar2b | protein kinase, cAMP dependent regulatory, type II beta | 406.03 | 216.46 | -1.88 | 0.000421 |
| Slc25a27 | solute carrier family 25, member 27 | 111.93 | 37.81 | -2.96 | 0.002118 |
|  | Rn.10293.2 | 56.43 | 29.79 | -1.89 | 0.002002 |
| Tfec | transcription factor EC | 14.63 | 57.79 | 3.95 | 0.001254 |
| Ina | internexin neuronal intermediate filament protein, alpha | 1778.59 | 568.77 | -3.13 | 0.001204 |
| Oas1a | 2'-5' oligoadenylate synthetase 1A | 55.33 | 129.87 | 2.35 | 0.001687 |
| Apob | apolipoprotein B | 10.53 | 51.43 | 4.89 | 0.001464 |
| Mrgprx3 | MAS-related GPR, member X3 | 34.72 | 102.72 | 2.96 | 0.005132 |
| Nos3 | nitric oxide synthase 3, endothelial cell | 10.80 | 27.38 | 2.53 | 0.013219 |
| Rpsa | ribosomal protein SA | 1413.77 | 2721.41 | 1.92 | 0.000009 |
| Tnfaip6 | tumor necrosis factor alpha induced protein 6 | 9.19 | 59.63 | 6.49 | 0.014032 |
| Tnfaip6 | tumor necrosis factor alpha induced protein 6 | 70.36 | 652.96 | 9.28 | 0.016817 |
| RT1-CE5 | RT1 class I, locus CE5 | 4.68 | 108.84 | 23.24 | 0.023436 |
| RT1-CE5 | RT1 class I, locus CE5 /// RT1 class Ib, locus EC2 | 8.11 | 36.34 | 4.48 | 0.045842 |
| Mt1a | metallothionein 1a | 472.24 | 5533.52 | 11.72 | 0.000003 |
| Tpm3 | tropomyosin 3, gamma | 27.15 | 100.46 | 3.70 | 0.007707 |
| Aprt | adenine phosphoribosyl transferase | 1345.46 | 2686.34 | 2.00 | 0.000075 |
| Sprr1al | small proline-rich protein 1A-like | 21.14 | 69.88 | 3.31 | 0.001640 |
| Xbp1 | X-box binding protein 1 | 1071.22 | 1831.30 | 1.71 | 0.002925 |
| Pf4 | platelet factor 4 | 106.11 | 1155.49 | 10.89 | 0.003908 |
| Ptpn18 | protein tyrosine phosphatase, non-receptor type 18 | 57.82 | 263.95 | 4.56 | 0.000296 |
| Serf2 | small EDRK-rich factor 2 | 1842.49 | 3389.91 | 1.84 | 0.000100 |
| Serpinh1 | serine (or cysteine) peptidase inhibitor, clade H, member 1 | 500.89 | 1171.18 | 2.34 | 0.013454 |
| Lamc1 | laminin, gamma 1 | 448.82 | 874.12 | 1.95 | 0.014571 |
| Ctsa | cathepsin A | 1504.50 | 3778.71 | 2.51 | 0.000102 |
|  | Rn.40577.1 | 137.40 | 664.95 | 4.84 | 0.002763 |
| Igfbp7 | insulin-like growth factor binding protein 7 | 1299.57 | 3469.18 | 2.67 | 0.000292 |
| Arhgdia | Rho GDP dissociation inhibitor (GDI) alpha | 568.24 | 1143.35 | 2.01 | 0.013092 |
| Sec61a1 | Sec61 alpha 1 subunit (S. cerevisiae) | 326.80 | 688.08 | 2.11 | 0.000194 |
| Col6a2 | collagen, type VI, alpha 2 | 67.93 | 237.72 | 3.50 | 0.001512 |
| Flna | filamin A, alpha | 245.18 | 766.44 | 3.13 | 0.001196 |
| Plxnb2 | plexin B2 | 295.73 | 1217.09 | 4.12 | 0.000105 |
| Vat1 | vesicle amine transport protein 1 homolog (T californica) | 221.69 | 707.50 | 3.19 | 0.012554 |
| Naca | nascent polypeptide-associated complex alpha subunit | 1327.00 | 2352.71 | 1.77 | 0.003105 |
| Epb4.1 | Erythrocyte membrane protein band 4.1 | 299.00 | 636.71 | 2.13 | 0.008983 |
|  | Rn.2199.1 | 2133.74 | 4293.40 | 2.01 | 0.000010 |
| Mapkapk2 | mitogen-activated protein kinase-activated protein kinase 2 | 302.75 | 785.22 | 2.59 | 0.000345 |
| Plac8 | placenta-specific 8 | 270.23 | 2727.32 | 10.09 | 0.000391 |
| Ubl7 | ubiquitin-like 7 (bone marrow stromal cell-derived) | 234.11 | 715.62 | 3.06 | 0.009290 |
| Igfbp4 | insulin-like growth factor binding protein 4 | 57.53 | 196.50 | 3.42 | 0.002944 |
| Reep5 | receptor accessory protein 5 | 1434.14 | 2544.09 | 1.77 | 0.000022 |
| Sh3bgrl3 | SH3 domain binding glutamic acid-rich protein-like 3 | 676.67 | 1446.65 | 2.14 | 0.002286 |
| Nid1 | Nidogen 1 | 537.25 | 1439.39 | 2.68 | 0.003808 |
| Sin3b | SIN3 homolog B, transcription regulator (yeast) | 543.30 | 1269.34 | 2.34 | 0.000156 |
| Slc12a7 | solute carrier family 12 (potassium/chloride transporters), member 7 | 95.45 | 300.55 | 3.15 | 0.003594 |
| Emp1 | epithelial membrane protein 1 | 751.39 | 1674.72 | 2.23 | 0.003435 |
| Ripk1 | receptor (TNFRSF)-interacting serine-threonine kinase 1 | 125.85 | 289.14 | 2.30 | 0.000039 |
| Carhsp1 | calcium regulated heat stable protein 1 | 464.44 | 896.13 | 1.93 | 0.004095 |
| Brsk1 | BR serine/threonine kinase 1 | 1015.34 | 492.13 | -2.06 | 0.000280 |
| Tuba4a | tubulin, alpha 4A | 1915.78 | 1082.94 | -1.77 | 0.000041 |
| Snx12 | sorting nexin 12 | 396.18 | 766.39 | 1.93 | 0.000036 |
| Nadk | NAD kinase | 367.45 | 801.02 | 2.18 | 0.002787 |
| Sncb | synuclein, beta | 1983.80 | 1054.66 | -1.88 | 0.000019 |
|  | Rn.9529.1 | 907.03 | 309.71 | -2.93 | 0.000005 |
|  | Rn.2762.1 | 1009.65 | 2146.63 | 2.13 | 0.000038 |
| Erlin1 | ER lipid raft associated 1 | 224.02 | 392.69 | 1.75 | 0.000594 |
|  | Rn.8145.1 | 1759.23 | 3429.47 | 1.95 | 0.004907 |
| Dgat2 | diacylglycerol O-acyltransferase homolog 2 (mouse) | 330.06 | 622.04 | 1.88 | 0.002997 |
| LOC100363662 | 6-phosphogluconate dehydrogenase, decarboxylating-like | 630.39 | 1084.49 | 1.72 | 0.000474 |
|  | Rn.3923.1 | 697.50 | 2214.79 | 3.18 | 0.001837 |
| Rhoc | ras homolog gene family, member C | 511.36 | 1101.99 | 2.15 | 0.000032 |
| Pxn | paxillin | 388.66 | 1119.23 | 2.88 | 0.000609 |
| Smarcd3 | SWI/SNF related, matrix associated, actin dependent regulator of chromatin, subfamily d, member 3 | 348.88 | 178.16 | -1.96 | 0.000084 |
| Rxra | Retinoid X receptor alpha | 330.82 | 572.46 | 1.73 | 0.000103 |
| Rarres2 | retinoic acid receptor responder (tazarotene induced) 2 | 529.59 | 1402.89 | 2.65 | 0.000568 |
| Mllt11 | myeloid/lymphoid or mixed-lineage leukemia (trithorax homolog, Drosophila); translocated to, 11 | 2100.33 | 995.54 | -2.11 | 0.000027 |
| Rexo2 | REX2, RNA exonuclease 2 homolog (S. cerevisiae) | 1099.69 | 1831.13 | 1.67 | 0.000013 |
| Myh9 | Myosin, heavy chain 9, non-muscle | 438.06 | 1060.80 | 2.42 | 0.000174 |
| Glb1 | galactosidase, beta 1 | 381.30 | 960.06 | 2.52 | 0.001682 |
|  | Rn.53871.1 | 95.86 | 41.65 | -2.30 | 0.001492 |
| Slc25a25 | solute carrier family 25 (mitochondrial carrier, phosphate carrier), member 25 | 776.73 | 352.30 | -2.20 | 0.000320 |
| Scamp2 | secretory carrier membrane protein 2 | 343.82 | 1076.42 | 3.13 | 0.000005 |
| Sat1 | spermidine/spermine N1-acetyl transferase 1 | 1370.88 | 3781.23 | 2.76 | 0.000003 |
| Pabpc4 | poly(A) binding protein, cytoplasmic 4 | 573.34 | 983.51 | 1.72 | 0.001070 |
| Stat3 | signal transducer and activator of transcription 3 | 105.50 | 267.25 | 2.53 | 0.000074 |
| Nipsnap3b | nipsnap homolog 3B (C. elegans) | 235.51 | 435.94 | 1.85 | 0.000812 |
| Tnfrsf12a | tumor necrosis factor receptor superfamily, member 12a | 150.26 | 315.80 | 2.10 | 0.002225 |
| Surf4 | surfeit 4 | 235.73 | 489.88 | 2.08 | 0.000736 |
| LOC100365370 | nuclear LIM interactor-interacting factor 2-like | 940.91 | 1589.63 | 1.69 | 0.001738 |
| Gm2a | GM2 ganglioside activator | 541.42 | 1666.43 | 3.08 | 0.000068 |
| Cdadc1 | cytidine and dCMP deaminase domain containing 1 | 680.58 | 394.56 | -1.72 | 0.000053 |
| Isyna1 | inositol-3-phosphate synthase 1 | 217.58 | 588.76 | 2.71 | 0.001697 |
|  | Rn.3856.1 | 698.33 | 1418.53 | 2.03 | 0.001046 |
| Bri3 | brain protein I3 | 602.22 | 1112.72 | 1.85 | 0.000012 |
| Rab5c | RAB5C, member RAS oncogene family | 308.18 | 630.57 | 2.05 | 0.023992 |
| Crtap | cartilage associated protein | 186.71 | 429.32 | 2.30 | 0.000265 |
| Pnrc2 | proline-rich nuclear receptor coactivator 2 | 1568.25 | 2743.95 | 1.75 | 0.000003 |
| Rrm1 | ribonucleotide reductase M1 | 317.74 | 592.72 | 1.87 | 0.001132 |
| Manba | mannosidase, beta A, lysosomal | 312.60 | 633.13 | 2.03 | 0.024778 |
| Dexi | dexamethasone-induced transcript | 1042.62 | 647.36 | -1.61 | 0.000138 |
| Maml1 | mastermind like 1 (Drosophila) | 139.40 | 270.65 | 1.94 | 0.000066 |
| Gns | Glucosamine (N-acetyl)-6-sulfatase | 1074.60 | 2826.32 | 2.63 | 0.000209 |
| Nxt1 | NTF2-like export factor 1 | 293.56 | 514.02 | 1.75 | 0.000912 |
|  | Rn.18190.1 | 73.80 | 446.71 | 6.05 | 0.001546 |
| Tgfbi | transforming growth factor, beta induced | 434.34 | 1239.52 | 2.85 | 0.002849 |
| Car12 | Carbonic anyhydrase 12 | 229.65 | 88.37 | -2.60 | 0.000139 |
| Olfml3 | olfactomedin-like 3 | 144.29 | 347.43 | 2.41 | 0.000118 |
| Cdca8 | cell division cycle associated 8 | 62.04 | 161.26 | 2.60 | 0.003225 |
| Qars | glutaminyl-tRNA synthetase /// similar to glutaminyl-tRNA synthetase | 580.15 | 989.06 | 1.70 | 0.001685 |
| Eif4a1 | eukaryotic translation initiation factor 4A1 | 1034.56 | 1890.64 | 1.83 | 0.002466 |
| RGD1303130 | kidney predominant protein NCU-G1 | 404.79 | 1378.01 | 3.40 | 0.000046 |
| Bzw1 | basic leucine zipper and W2 domains 1 | 392.85 | 656.21 | 1.67 | 0.000224 |
| Fhl2 | four and a half LIM domains 2 | 388.11 | 1039.14 | 2.68 | 0.014325 |
| Pld3 | phospholipase D family, member 3 | 273.06 | 715.77 | 2.62 | 0.005929 |
|  | Rn.6899.1 | 782.43 | 1508.11 | 1.93 | 0.005153 |
| Fam111a | family with sequence similarity 111, member A | 10.21 | 132.25 | 12.95 | 0.018706 |
| Phyhd1 | phytanoyl-CoA dioxygenase domain containing 1 | 871.96 | 1506.95 | 1.73 | 0.000483 |
| Arpc3 | actin related protein 2/3 complex, subunit 3 | 2026.27 | 3789.27 | 1.87 | 0.000139 |
| Man1a1 | mannosidase, alpha, class 1A, member 1 | 380.08 | 989.57 | 2.60 | 0.000569 |
| Arhgap1 | Rho GTPase activating protein 1 | 153.53 | 272.66 | 1.78 | 0.001934 |
|  | Rn.1477.1 | 763.69 | 1361.25 | 1.78 | 0.001749 |
| Mobkl1b | MOB1, Mps One Binder kinase activator-like 1B (yeast) | 998.71 | 2406.35 | 2.41 | 0.000022 |
| Hebp1 | heme binding protein 1 | 336.17 | 874.52 | 2.60 | 0.000203 |
|  | Rn.954.1 | 144.37 | 519.01 | 3.59 | 0.005776 |
|  | Rn.9477.1 | 354.32 | 2174.92 | 6.14 | 0.001820 |
| Dhcr24 | 24-dehydrocholesterol reductase | 1499.01 | 763.92 | -1.96 | 0.000700 |
| Ifitm1 | interferon induced transmembrane protein 1 | 99.10 | 4195.93 | 42.34 | 0.000046 |
|  | Rn.19473.1 | 132.05 | 53.07 | -2.49 | 0.010171 |
|  | Rn.9506.1 | 269.25 | 609.37 | 2.26 | 0.001349 |
|  | Rn.2548.1 | 414.75 | 2170.70 | 5.23 | 0.000438 |
| Parp14 | poly (ADP-ribose) polymerase family, member 14 | 121.91 | 504.71 | 4.14 | 0.000863 |
| Mknk2 | MAP kinase-interacting serine/threonine kinase 2 | 456.70 | 1000.01 | 2.19 | 0.004843 |
| Cmtm3 | CKLF-like MARVEL transmembrane domain containing 3 | 135.40 | 672.47 | 4.97 | 0.000391 |
| RGD1310348 | similar to Ser/Thr-rich protein T10 in DGCR region | 884.43 | 487.40 | -1.81 | 0.000018 |
| Glt25d1 | glycosyltransferase 25 domain containing 1 | 190.59 | 477.74 | 2.51 | 0.000719 |
| Cmtm6 | CKLF-like MARVEL transmembrane domain containing 6 | 312.27 | 1252.96 | 4.01 | 0.000490 |
| Cxcl16 | chemokine (C-X-C motif) ligand 16 | 165.28 | 779.55 | 4.72 | 0.000256 |
| Art3 | ADP-ribosyltransferase 3 | 1611.83 | 907.65 | -1.78 | 0.001591 |
| Ifi30 | interferon gamma inducible protein 30 | 423.12 | 3539.02 | 8.36 | 0.000000 |
| Necap2 | NECAP endocytosis associated 2 | 481.54 | 895.17 | 1.86 | 0.000122 |
| Iah1 | isoamyl acetate-hydrolyzing esterase 1 homolog (S. cerevisiae) | 383.90 | 883.87 | 2.30 | 0.001239 |
| Ppfibp1 | PTPRF interacting protein, binding protein 1 (liprin beta 1) | 656.19 | 1218.33 | 1.86 | 0.001061 |
|  | Rn.13742.1 | 573.54 | 268.91 | -2.13 | 0.000191 |
|  | Rn.3765.1 | 233.84 | 1393.21 | 5.96 | 0.000275 |
| Ehd4 | EH-domain containing 4 | 317.32 | 668.83 | 2.11 | 0.000582 |
| Trim5 | tripartite motif-containing 5 | 247.27 | 756.35 | 3.06 | 0.000221 |
| Nsg2 | neuron specific gene family member 2 | 139.43 | 65.12 | -2.14 | 0.001869 |
| Gpx7 | glutathione peroxidase 7 | 158.59 | 423.86 | 2.67 | 0.001315 |
| Cndp2 | CNDP dipeptidase 2 (metallopeptidase M20 family) | 288.21 | 929.45 | 3.22 | 0.002608 |
| Rras2 | related RAS viral (r-ras) oncogene homolog 2 | 417.35 | 1115.38 | 2.67 | 0.000012 |
| Chchd6 | coiled-coil-helix-coiled-coil-helix domain containing 6 | 586.67 | 344.28 | -1.70 | 0.000405 |
| Cux1 | cut-like homeobox 1 | 257.61 | 665.72 | 2.58 | 0.000348 |
| Ccdc28a | coiled-coil domain containing 28A | 523.21 | 288.27 | -1.82 | 0.000063 |
| Acss1 | acyl-CoA synthetase short-chain family member 1 | 282.78 | 501.60 | 1.77 | 0.001314 |
| Snrpg | small nuclear ribonucleoprotein polypeptide G | 1070.42 | 1922.46 | 1.80 | 0.000313 |
| Top2a | topoisomerase (DNA) II alpha | 13.35 | 206.06 | 15.43 | 0.000894 |
| Prkd3 | protein kinase D3 | 488.64 | 847.38 | 1.73 | 0.001250 |
| Akap10 | A kinase (PRKA) anchor protein 10 | 225.68 | 435.82 | 1.93 | 0.000204 |
| Tnnc2 | troponin C type 2 (fast) | 405.08 | 101.85 | -3.98 | 0.000273 |
| Gpsm3 | G-protein signaling modulator 3 (AGS3-like, C. elegans) | 112.26 | 461.28 | 4.11 | 0.000000 |
|  | Rn.23814.1 | 1267.30 | 547.71 | -2.31 | 0.000279 |
| Nod1 | nucleotide-binding oligomerization domain containing 1 | 173.41 | 450.03 | 2.60 | 0.000029 |
| Ostf1 | osteoclast stimulating factor 1 | 1067.54 | 2004.72 | 1.88 | 0.000149 |
| Ddost | dolichyl-diphosphooligosaccharide-protein glycosyltransferase | 583.11 | 1107.91 | 1.90 | 0.000220 |
| Serping1 | serine (or cysteine) peptidase inhibitor, clade G, member 1 | 590.14 | 3391.68 | 5.75 | 0.000012 |
| Crip | cysteine-rich intestinal protein | 208.31 | 932.68 | 4.48 | 0.007099 |
| Gypc | glycophorin C (Gerbich blood group) | 142.87 | 339.79 | 2.38 | 0.004976 |
| Slc25a12 | solute carrier family 25 (mitochondrial carrier, Aralar), member 12 | 1026.27 | 557.18 | -1.84 | 0.000032 |
| Pxdn | peroxidasin homolog (Drosophila) | 220.19 | 442.05 | 2.01 | 0.027066 |
| Sh3bgr | SH3 domain binding glutamic acid-rich protein | 224.38 | 110.62 | -2.03 | 0.000786 |
| Aebp1 | AE binding protein 1 | 641.30 | 1337.81 | 2.09 | 0.000202 |
|  | Rn.8110.1 | 164.14 | 382.10 | 2.33 | 0.001188 |
| Isoc1 | isochorismatase domain containing 1 | 1092.07 | 627.92 | -1.74 | 0.000205 |
| Gnpda1 | glucosamine-6-phosphate deaminase 1 | 180.81 | 579.24 | 3.20 | 0.000473 |
| Ehd1 | EH-domain containing 1 | 482.33 | 827.70 | 1.72 | 0.000326 |
|  | Rn.7416.1 | 758.13 | 358.92 | -2.11 | 0.001503 |
| LOC100365743 | protein lyl-1-like /// lymphoblastic leukemia derived sequence 1 | 34.23 | 451.52 | 13.19 | 0.000007 |
| Emilin1 | elastin microfibril interfacer 1 | 127.05 | 261.06 | 2.05 | 0.002702 |
| Slc2a3 | Solute carrier family 2 (facilitated glucose transporter), member 3 | 398.88 | 217.72 | -1.83 | 0.000440 |
|  | Rn.13655.1 | 288.91 | 568.21 | 1.97 | 0.000117 |
| LOC682105 | similar to receptor expression enhancing protein 2 | 522.09 | 262.86 | -1.99 | 0.000889 |
| LOC100188932 | dolichyl-diphosphooligosaccharide--protein glycosyltransferase subunit 4 | 743.58 | 1340.64 | 1.80 | 0.003876 |
| Marveld1 | MARVEL domain containing 1 | 164.02 | 399.74 | 2.44 | 0.009818 |
|  | Rn.17126.1 | 3185.22 | 1468.59 | -2.17 | 0.000110 |
| Rin2 | Ras and Rab interactor 2 | 771.79 | 1808.22 | 2.34 | 0.000238 |
| Ddx41 | DEAD (Asp-Glu-Ala-Asp) box polypeptide 41 | 42.36 | 112.51 | 2.66 | 0.006314 |
| RGD1561067 | similar to RNA binding protein gene with multiple splicing | 16.13 | 40.73 | 2.52 | 0.005595 |
|  | Rn.16382.1 | 4195.83 | 2312.03 | -1.81 | 0.000073 |
| Ier2 | immediate early response 2 | 137.68 | 343.26 | 2.49 | 0.030188 |
| Rac2 | ras-related C3 botulinum toxin substrate 2 (rho family, small GTP binding protein Rac2) | 278.63 | 2087.54 | 7.49 | 0.000018 |
| Mcm3 | minichromosome maintenance complex component 3 | 30.02 | 112.86 | 3.76 | 0.000682 |
| C1qtnf6 | C1q and tumor necrosis factor related protein 6 | 57.37 | 104.81 | 1.83 | 0.001310 |
| Aga | aspartylglucosaminidase | 951.12 | 1558.72 | 1.64 | 0.000020 |
| Cip98 | CASK-interacting protein CIP98 | 569.64 | 278.35 | -2.05 | 0.000370 |
| Col4a1 | collagen, type IV, alpha 1 | 94.32 | 432.65 | 4.59 | 0.008011 |
| Vasp | vasodilator-stimulated phosphoprotein | 294.54 | 983.72 | 3.34 | 0.000017 |
| Acat2 | acetyl-Coenzyme A acetyltransferase 2 | 1195.18 | 573.39 | -2.08 | 0.000508 |
|  | Rn.2705.1 | 1085.24 | 2906.54 | 2.68 | 0.000289 |
| Cd97 | CD97 molecule | 68.29 | 205.87 | 3.01 | 0.004702 |
| Cmtm7 | CKLF-like MARVEL transmembrane domain containing 7 | 289.03 | 1148.00 | 3.97 | 0.000039 |
|  | Rn.2721.1 | 215.78 | 1167.64 | 5.41 | 0.002768 |
| RGD1565591 | similar to Ski protein | 585.05 | 1070.79 | 1.83 | 0.000372 |
| Cldn10 | claudin 10 | 855.64 | 492.44 | -1.74 | 0.001200 |
| Tmod3 | tropomodulin 3 | 611.85 | 1368.02 | 2.24 | 0.000600 |
| Tnfsf12 | tumor necrosis factor ligand superfamily member 12 | 195.62 | 451.90 | 2.31 | 0.000040 |
| Rac1 | ras-related C3 botulinum toxin substrate 1 | 616.79 | 1244.32 | 2.02 | 0.000465 |
| Kif22 | kinesin family member 22 | 85.93 | 333.98 | 3.89 | 0.000364 |
| Mcl1 | myeloid cell leukemia sequence 1 | 333.27 | 1045.32 | 3.14 | 0.001554 |
| Rtn2 | reticulon 2 | 392.93 | 168.46 | -2.33 | 0.000304 |
| Edem1 | ER degradation enhancer, mannosidase alpha-like 1 | 461.27 | 1090.90 | 2.37 | 0.001553 |
| Mapkapk3 | mitogen-activated protein kinase-activated protein kinase 3 | 116.12 | 203.37 | 1.75 | 0.000186 |
| Tmem88 | transmembrane protein 88 | 127.01 | 243.45 | 1.92 | 0.003107 |
|  | Rn.7337.1 | 336.69 | 614.05 | 1.82 | 0.000799 |
| Htra3 | HtrA serine peptidase 3 | 697.23 | 1906.89 | 2.73 | 0.001150 |
| Fhl3 | four and a half LIM domains 3 | 126.16 | 338.63 | 2.68 | 0.000022 |
| Arpc4 | actin related protein 2/3 complex, subunit 4 | 403.69 | 781.40 | 1.94 | 0.000280 |
| Eng | endoglin | 100.33 | 282.31 | 2.81 | 0.000136 |
|  | Rn.13877.1 | 1195.89 | 543.26 | -2.20 | 0.000375 |
|  | Rn.12486.1 | 224.17 | 1468.55 | 6.55 | 0.006555 |
| Mgst2 | microsomal glutathione S-transferase 2 | 115.66 | 488.31 | 4.22 | 0.000185 |
|  | Rn.2438.1 | 107.18 | 272.44 | 2.54 | 0.009636 |
| Aoc3 | amine oxidase, copper containing 3 (vascular adhesion protein 1) | 142.79 | 57.19 | -2.50 | 0.025511 |
| Fam176b | family with sequence similarity 176, member B | 188.16 | 413.15 | 2.20 | 0.001001 |
| Ano6 | anoctamin 6 | 342.20 | 794.26 | 2.32 | 0.015489 |
| Tk1 | thymidine kinase 1, soluble | 118.62 | 271.45 | 2.29 | 0.000191 |
| LOC683751 | similar to trophinin isoform 1 | 1561.34 | 762.46 | -2.05 | 0.000015 |
| Alpk3 | Alpha-kinase 3 | 120.92 | 271.21 | 2.24 | 0.000136 |
| LOC100363743 | proline arginine-rich end leucine-rich repeat protein-like | 308.27 | 853.13 | 2.77 | 0.000076 |
|  | Rn.63325.1 | 171.62 | 75.89 | -2.26 | 0.000108 |
| Qprt | quinolinate phosphoribosyltransferase | 149.13 | 277.11 | 1.86 | 0.005140 |
| RGD1307218 | similar to RIKEN cDNA 2810432L12 | 556.25 | 319.63 | -1.74 | 0.001300 |
| Cdkn3 | cyclin-dependent kinase inhibitor 3 | 45.59 | 359.59 | 7.89 | 0.000257 |
| Upp1 | uridine phosphorylase 1 | 96.27 | 353.64 | 3.67 | 0.013916 |
|  | Rn.18757.1 | 1004.25 | 578.50 | -1.74 | 0.000082 |
| Hexb | hexosaminidase B | 1834.14 | 3242.71 | 1.77 | 0.000229 |
|  | Rn.7891.1 | 822.54 | 1385.09 | 1.68 | 0.000613 |
| Plscr2 | phospholipid scramblase 2 | 99.43 | 265.90 | 2.67 | 0.001520 |
| Hist1h4b | histone cluster 1, H4b | 243.59 | 681.90 | 2.80 | 0.000004 |
| Procr | protein C receptor, endothelial | 77.96 | 267.65 | 3.43 | 0.001463 |
| Smagp | small trans-membrane and glycosylated protein | 43.72 | 159.56 | 3.65 | 0.000799 |
| Tbc1d2b | TBC1 domain family, member 2B | 275.70 | 631.39 | 2.29 | 0.000217 |
| Tspan4 | tetraspanin 4 | 174.62 | 613.92 | 3.52 | 0.000488 |
| Mal2 | mal, T-cell differentiation protein 2 | 347.48 | 153.57 | -2.26 | 0.000013 |
| Stat1 | signal transducer and activator of transcription 1 /// signal transducer and activator of transcription 4 | 831.98 | 1416.14 | 1.70 | 0.001434 |
| LOC683667 | similar to sorcin | 359.40 | 817.66 | 2.28 | 0.000881 |
| Slc39a1 | solute carrier family 39 (zinc transporter), member 1 | 981.77 | 2267.47 | 2.31 | 0.000090 |
|  | Rn.13492.1 | 1861.04 | 789.75 | -2.36 | 0.000665 |
| Magoh | mago-nashi homolog, proliferation-associated (Drosophila) | 461.70 | 824.83 | 1.79 | 0.008744 |
| Plekhf2 | pleckstrin homology domain containing, family F (with FYVE domain) member 2 | 366.59 | 764.35 | 2.09 | 0.001762 |
| Rhoj | ras homolog gene family, member J | 212.20 | 421.78 | 1.99 | 0.005601 |
| RGD1309410 | LOC363020 | 68.62 | 139.03 | 2.03 | 0.007058 |
| Cybasc3 | cytochrome b, ascorbate dependent 3 | 136.61 | 251.35 | 1.84 | 0.003650 |
| Acn9 | ACN9 homolog (S. cerevisiae) | 426.61 | 224.19 | -1.90 | 0.000078 |
| RGD1310269 | hypothetical LOC314472 | 841.74 | 331.72 | -2.54 | 0.000022 |
| LOC100151767 | hypothetical LOC100151767 | 237.78 | 424.74 | 1.79 | 0.001869 |
| Ptprcap | protein tyrosine phosphatase, receptor type, C-associated protein | 35.96 | 108.34 | 3.01 | 0.004504 |
| Rela | v-rel reticuloendotheliosis viral oncogene homolog A (avian) | 198.83 | 359.97 | 1.81 | 0.000604 |
| Kdelr3 | KDEL (Lys-Asp-Glu-Leu) endoplasmic reticulum protein retention receptor 3 | 132.01 | 308.64 | 2.34 | 0.009966 |
| Tacc3 | transforming, acidic coiled-coil containing protein 3 | 51.45 | 323.46 | 6.29 | 0.000330 |
|  | Rn.4119.1 | 72.73 | 267.68 | 3.68 | 0.023738 |
|  | Rn.11930.1 | 805.64 | 2142.31 | 2.66 | 0.000004 |
| Plod2 | procollagen lysine, 2-oxoglutarate 5-dioxygenase 2 | 151.45 | 467.05 | 3.08 | 0.005793 |
| Nhlrc3 | NHL repeat containing 3 | 168.30 | 363.68 | 2.16 | 0.007813 |
| Kif18b | kinesin family member 18B /// kinesin-like protein KIF18B-like | 9.03 | 93.29 | 10.33 | 0.004672 |
| Mobkl2a | MOB1, Mps One Binder kinase activator-like 2A (yeast) | 138.28 | 298.62 | 2.16 | 0.014843 |
| Ltbr | lymphotoxin beta receptor (TNFR superfamily, member 3) | 329.68 | 582.13 | 1.77 | 0.000101 |
| Prodh | proline dehydrogenase | 536.35 | 262.89 | -2.04 | 0.005373 |
| Sirt3 | sirtuin (silent mating type information regulation 2 homolog) 3 (S. cerevisiae) | 691.15 | 401.04 | -1.72 | 0.000274 |
| Sp110 | SP110 nuclear body protein | 186.85 | 642.65 | 3.44 | 0.000507 |
| Tmem119 | transmembrane protein 119 | 191.69 | 477.08 | 2.49 | 0.016296 |
| sep-11 | Septin 11 | 667.11 | 1231.70 | 1.85 | 0.001754 |
| Cables2 | Cdk5 and Abl enzyme substrate 2 | 638.96 | 326.84 | -1.95 | 0.000069 |
|  | Rn.24230.1 | 6.92 | 61.65 | 8.91 | 0.000671 |
| Nme4 | non-metastatic cells 4, protein expressed in | 30.11 | 103.30 | 3.43 | 0.001938 |
| Mfsd2 | major facilitator superfamily domain containing 2 | 413.24 | 215.31 | -1.92 | 0.008641 |
| Lss | Lanosterol synthase (2,3-oxidosqualene-lanosterol cyclase) | 914.20 | 375.55 | -2.43 | 0.000870 |
|  | Rn.44131.1 | 252.54 | 97.40 | -2.59 | 0.000146 |
| Lor | loricrin | 66.97 | 29.35 | -2.28 | 0.004472 |
|  | Rn.38688.1 | 247.79 | 489.45 | 1.98 | 0.000137 |
| C1qc | complement component 1, q subcomponent, C chain | 151.82 | 2760.29 | 18.18 | 0.000637 |
|  | Rn.18387.1 | 62.56 | 192.65 | 3.08 | 0.005603 |
| Ryk | receptor-like tyrosine kinase | 106.19 | 199.04 | 1.87 | 0.001899 |
|  | Rn.3212.1 | 120.91 | 490.45 | 4.06 | 0.015501 |
| Ube2l6 | ubiquitin-conjugating enzyme E2L 6 | 96.33 | 271.04 | 2.81 | 0.004820 |
| Sdf2l1 | stromal cell-derived factor 2-like 1 | 421.33 | 853.32 | 2.03 | 0.004618 |
|  | Rn.20664.1 | 294.54 | 812.78 | 2.76 | 0.000022 |
| Ptpn18 | protein tyrosine phosphatase, non-receptor type 18 | 196.43 | 1142.90 | 5.82 | 0.000003 |
|  | Rn.40480.1 | 476.78 | 892.04 | 1.87 | 0.002182 |
| Plagl2 | pleiomorphic adenoma gene-like 2 | 43.98 | 110.12 | 2.50 | 0.000331 |
|  | Rn.8191.1 | 440.51 | 752.59 | 1.71 | 0.004003 |
| Mta2 | metastasis associated 1 family, member 2 | 88.65 | 181.70 | 2.05 | 0.004531 |
| Zfp36l2 | zinc finger protein 36, C3H type-like 2 | 822.00 | 1922.68 | 2.34 | 0.000005 |
| March8 | membrane-associated ring finger (C3HC4) 8 | 1387.77 | 2265.60 | 1.63 | 0.000385 |
|  | Rn.18597.1 | 265.28 | 613.73 | 2.31 | 0.002745 |
| Klc2 | kinesin light chain 2 | 420.11 | 225.53 | -1.86 | 0.000532 |
| Rcn3 | reticulocalbin 3, EF-hand calcium binding domain | 290.31 | 648.82 | 2.23 | 0.000662 |
| Myom2 | myomesin 2 | 44.23 | 21.58 | -2.05 | 0.018208 |
| Il6st | interleukin 6 signal transducer | 315.22 | 575.57 | 1.83 | 0.003668 |
| RGD1309621 | similar to hypothetical protein FLJ10652 | 282.17 | 519.72 | 1.84 | 0.002035 |
| LOC100360417 | RUN and SH3 domain containing 1-like | 379.77 | 221.64 | -1.71 | 0.000454 |
| Cpxm2 | carboxypeptidase X (M14 family), member 2 | 447.39 | 217.56 | -2.06 | 0.016383 |
| Armcx2 | armadillo repeat containing, X-linked 2 | 1299.90 | 790.88 | -1.64 | 0.000019 |
| Ppapdc1b | phosphatidic acid phosphatase type 2 domain containing 1B | 298.81 | 554.80 | 1.86 | 0.001642 |
|  | Rn.1300.1 | 251.55 | 605.04 | 2.41 | 0.000767 |
| Plbd1 | phospholipase B domain containing 1 | 40.65 | 761.52 | 18.73 | 0.000655 |
|  | Rn.34800.1 | 392.20 | 159.00 | -2.47 | 0.000120 |
|  | Rn.17903.1 | 1095.76 | 537.94 | -2.04 | 0.000913 |
| Scn4b | sodium channel, voltage-gated, type IV, beta | 3417.62 | 1547.10 | -2.21 | 0.000011 |
| Wdr60 | WD repeat domain 60 | 208.26 | 440.88 | 2.12 | 0.001187 |
| Efha2 | EF hand domain family, member A2 | 1518.27 | 810.70 | -1.87 | 0.000034 |
| Dnajc22 | DnaJ (Hsp40) homolog, subfamily C, member 22 | 5.44 | 63.13 | 11.60 | 0.007681 |
| Tmem176a | transmembrane protein 176A | 413.38 | 2492.70 | 6.03 | 0.000778 |
| Fndc3b | Fibronectin type III domain containing 3B | 365.01 | 1002.60 | 2.75 | 0.000111 |
|  | Rn.8190.1 | 468.23 | 851.53 | 1.82 | 0.002188 |
|  | Rn.48387.1 | 193.12 | 562.96 | 2.92 | 0.024990 |
| Fbxw4 | F-box and WD repeat domain containing 4 | 384.09 | 1221.98 | 3.18 | 0.000988 |
|  | Rn.23904.1 | 242.71 | 125.64 | -1.93 | 0.004478 |
| Hexa | hexosaminidase A | 846.91 | 2419.76 | 2.86 | 0.000003 |
|  | Rn.4067.2 | 1211.39 | 2272.11 | 1.88 | 0.001496 |
| RGD1308019 | similar to hypothetical protein FLJ20245 | 103.89 | 343.86 | 3.31 | 0.000001 |
| Lhfpl2 | lipoma HMGIC fusion partner-like 2 | 394.08 | 1147.21 | 2.91 | 0.000646 |
|  | Rn.34796.1 | 348.42 | 601.46 | 1.73 | 0.000099 |
| Dhrs3 | dehydrogenase/reductase (SDR family) member 3 | 235.90 | 676.27 | 2.87 | 0.003589 |
| Col4a1 | collagen, type IV, alpha 1 | 137.35 | 638.88 | 4.65 | 0.003226 |
| LOC300225 | Similar to formin-like 3 protein | 164.20 | 418.70 | 2.55 | 0.003067 |
|  | Rn.8614.4 | 2034.89 | 955.78 | -2.13 | 0.000026 |
| RGD1309543 | similar to 2310014H01Rik protein | 79.05 | 385.79 | 4.88 | 0.000290 |
| Zbed3 | zinc finger, BED-type containing 3 | 148.97 | 293.15 | 1.97 | 0.000968 |
| Fam107a | family with sequence similarity 107, member A /// downregulated in renal cell carcinoma-like | 875.38 | 2374.30 | 2.71 | 0.016548 |
| LOC691431 | similar to mitochondrial carrier protein MGC4399 | 723.79 | 369.10 | -1.96 | 0.000054 |
|  | Rn.14096.1 | 237.41 | 458.87 | 1.93 | 0.000274 |
| Fblim1 | filamin binding LIM protein 1 | 9.71 | 87.27 | 8.99 | 0.000696 |
| Tmem86a | transmembrane protein 86A | 127.37 | 554.19 | 4.35 | 0.000839 |
| Pnkd | paroxysmal nonkinesiogenic dyskinesia | 1235.99 | 715.03 | -1.73 | 0.000020 |
|  | Rn.67002.1 | 63.75 | 32.04 | -1.99 | 0.002478 |
|  | Rn.15193.1 | 722.81 | 341.61 | -2.12 | 0.014251 |
| Lrsam1 | leucine rich repeat and sterile alpha motif containing 1 | 372.17 | 217.51 | -1.71 | 0.000196 |
| Lmo4 | LIM domain only 4 | 1551.27 | 2677.73 | 1.73 | 0.001080 |
|  | Rn.2153.1 | 498.22 | 297.40 | -1.68 | 0.000334 |
| Mapre1 | microtubule-associated protein, RP/EB family, member 1 | 757.88 | 1339.55 | 1.77 | 0.000157 |
|  | Rn.9335.1 | 1317.33 | 547.78 | -2.40 | 0.000000 |
| Tnc | Tenascin C | 102.36 | 530.03 | 5.18 | 0.027698 |
| LOC684871 | similar to Protein C8orf4 (Thyroid cancer protein 1) (TC-1) | 32.74 | 134.79 | 4.12 | 0.035565 |
| Mef2c | myocyte enhancer factor 2C | 392.10 | 801.53 | 2.04 | 0.018494 |
| Tmem59l | transmembrane protein 59-like | 1047.61 | 544.72 | -1.92 | 0.000220 |
| Tgif1 | TGFB-induced factor homeobox 1 | 122.16 | 615.07 | 5.03 | 0.000095 |
| Ube2o | ubiquitin-conjugating enzyme E2O | 2034.04 | 1047.26 | -1.94 | 0.000087 |
|  | Rn.6499.1 | 388.49 | 677.40 | 1.74 | 0.005169 |
| Cast | calpastatin | 722.34 | 1216.42 | 1.68 | 0.000209 |
| Hspa12a | heat shock 70kDa protein 12A | 1130.80 | 640.25 | -1.77 | 0.000282 |
| Gmfg | glia maturation factor, gamma | 182.73 | 1197.22 | 6.55 | 0.000210 |
| Gba | glucosidase, beta, acid | 359.32 | 627.81 | 1.75 | 0.000694 |
| Glipr1 | GLI pathogenesis-related 1 | 136.55 | 1235.25 | 9.05 | 0.000001 |
| Rnf213 | ring finger protein 213 | 60.27 | 288.16 | 4.78 | 0.006053 |
| Fcgr3a | Fc fragment of IgG, low affinity IIIa, receptor | 138.40 | 1304.77 | 9.43 | 0.003885 |
| Pik3cd | phosphoinositide-3-kinase, catalytic, delta polypeptide | 95.40 | 302.51 | 3.17 | 0.000197 |
|  | Rn.1552.1 | 194.46 | 329.54 | 1.69 | 0.000218 |
| Cxcl9 | chemokine (C-X-C motif) ligand 9 | 9.48 | 162.66 | 17.15 | 0.010375 |
|  | Rn.22219.1 | 1772.71 | 840.93 | -2.11 | 0.000004 |
| Npepl1 | aminopeptidase-like 1 | 36.22 | 99.94 | 2.76 | 0.000177 |
| Cnpy4 | canopy 4 homolog (zebrafish) | 225.98 | 387.35 | 1.71 | 0.003002 |
| Fcer1g | Fc fragment of IgE, high affinity I, receptor for; gamma polypeptide | 691.69 | 4938.42 | 7.14 | 0.000300 |
|  | Rn.12046.1 | 676.59 | 1872.24 | 2.77 | 0.001504 |
| Trim2 | tripartite motif-containing 2 | 3135.40 | 1851.01 | -1.69 | 0.000123 |
|  | Rn.20373.1 | 1184.37 | 589.70 | -2.01 | 0.000005 |
| RGD1305664 | similar to KIAA0672 gene product | 695.55 | 301.92 | -2.30 | 0.000333 |
| Frmd8 | FERM domain containing 8 | 528.70 | 901.82 | 1.71 | 0.000500 |
| Stom | stomatin | 366.41 | 1080.48 | 2.95 | 0.000063 |
| Dnajc27 | DnaJ (Hsp40) homolog, subfamily C, member 27 | 660.51 | 341.99 | -1.93 | 0.000080 |
| Tmem43 | transmembrane protein 43 | 604.33 | 1134.54 | 1.88 | 0.001377 |
| RGD1310423 | similar to hypothetical protein FLJ31737 | 340.46 | 175.42 | -1.94 | 0.000043 |
| LOC100363275 | G protein-coupled receptor 124 | 276.69 | 579.94 | 2.10 | 0.010884 |
| Sec24d | SEC24 family, member D (S. cerevisiae) | 336.45 | 640.66 | 1.90 | 0.000897 |
| Il17ra | interleukin 17 receptor A | 235.01 | 698.44 | 2.97 | 0.000036 |
|  | Rn.15317.1 | 457.35 | 837.47 | 1.83 | 0.000014 |
| Chat | choline acetyltransferase | 361.31 | 149.22 | -2.42 | 0.000210 |
| Shmt1 | serine hydroxymethyltransferase 1 (soluble) | 39.50 | 100.26 | 2.54 | 0.000967 |
| Mypop | Myb-related transcription factor, partner of profilin | 198.98 | 96.39 | -2.06 | 0.002099 |
|  | Rn.23588.1 | 95.36 | 25.28 | -3.77 | 0.000676 |
| Paqr6 | progestin and adipoQ receptor family member VI | 1179.18 | 470.26 | -2.51 | 0.000030 |
| Slc31a2 | solute carrier family 31 (copper transporters), member 2 | 287.24 | 743.97 | 2.59 | 0.000165 |
| Racgap1 | Rac GTPase-activating protein 1 | 63.70 | 310.29 | 4.87 | 0.001871 |
| Cxcr4 | chemokine (C-X-C motif) receptor 4 | 90.48 | 437.89 | 4.84 | 0.000035 |
| Tor2a | torsin family 2, member A | 193.91 | 328.71 | 1.70 | 0.002118 |
|  | Rn.7615.1 | 451.33 | 240.17 | -1.88 | 0.000046 |
|  | Rn.13320.1 | 16.04 | 85.98 | 5.36 | 0.000665 |
| Kif20a | kinesin family member 20A | 17.19 | 373.34 | 21.72 | 0.001638 |
|  | Rn.13717.1 | 465.60 | 263.10 | -1.77 | 0.001560 |
| Spsb2 | splA/ryanodine receptor domain and SOCS box containing 2 | 177.07 | 321.46 | 1.82 | 0.000852 |
| Cnih2 | cornichon homolog 2 (Drosophila) | 381.10 | 220.59 | -1.73 | 0.000351 |
|  | Rn.1545.1 | 681.67 | 1185.51 | 1.74 | 0.000291 |
| Cend1 | cell cycle exit and neuronal differentiation 1 | 1657.76 | 819.30 | -2.02 | 0.000234 |
| Fam60a | family with sequence similarity 60, member A | 67.24 | 204.66 | 3.04 | 0.000033 |
| Pgm2 | phosphoglucomutase 2 | 283.89 | 535.25 | 1.89 | 0.000546 |
| Rit2 | Ras-like without CAAX 2 | 1509.41 | 683.11 | -2.21 | 0.000024 |
|  | Rn.8740.1 | 416.42 | 783.40 | 1.88 | 0.000515 |
|  | Rn.1141.1 | 297.15 | 871.83 | 2.93 | 0.001707 |
| Sbsn | suprabasin | 119.56 | 241.10 | 2.02 | 0.000465 |
| Pld4 | phospholipase D family, member 4 | 55.38 | 466.12 | 8.42 | 0.005256 |
| Lman2 | lectin, mannose-binding 2 | 408.09 | 765.84 | 1.88 | 0.000090 |
|  | Rn.13832.1 | 829.20 | 2608.62 | 3.15 | 0.000003 |
| Cks2 | CDC28 protein kinase regulatory subunit 2 | 20.15 | 486.36 | 24.14 | 0.002132 |
| LOC619574 | hypothetical protein LOC619574 | 108.44 | 239.42 | 2.21 | 0.002693 |
| Fut4 | fucosyltransferase 4 (alpha (1,3) fucosyltransferase, myeloid-specific) | 80.79 | 263.49 | 3.26 | 0.000141 |
| Srd5a3 | steroid 5 alpha-reductase 3 | 239.58 | 505.91 | 2.11 | 0.000859 |
| Snap91 | synaptosomal-associated protein 91 | 2461.18 | 1376.48 | -1.79 | 0.000057 |
| Soat1 | sterol O-acyltransferase 1 | 93.86 | 449.13 | 4.78 | 0.000261 |
| Eif4e2 | eukaryotic translation initiation factor 4E family member 2 | 167.22 | 420.23 | 2.51 | 0.000138 |
| Arhgdib | Rho, GDP dissociation inhibitor (GDI) beta | 188.93 | 722.40 | 3.82 | 0.000448 |
| Reep1 | receptor accessory protein 1 | 660.63 | 324.25 | -2.04 | 0.000704 |
| Igsf7 | immunoglobulin superfamily, member 7 /// similar to CLM3 /// similar to dendritic cell-derived immunoglobulin(Ig)-like receptor 1, DIgR1 - mouse | 256.68 | 1473.91 | 5.74 | 0.000011 |
| Zcchc12 | zinc finger, CCHC domain containing 12 | 1095.08 | 585.75 | -1.87 | 0.001889 |
| Rab31 | RAB31, member RAS oncogene family | 1190.13 | 1997.08 | 1.68 | 0.001676 |
|  | Rn.7596.1 | 1564.83 | 850.10 | -1.84 | 0.000332 |
| Lmnb1 | Lamin B1 | 173.58 | 437.87 | 2.52 | 0.004270 |
| Cdk2ap2 | CDK2-associated protein 2 | 258.23 | 503.11 | 1.95 | 0.007800 |
| Rcsd1 | RCSD domain containing 1 | 58.39 | 133.54 | 2.29 | 0.006495 |
|  | Rn.41951.1 | 132.16 | 300.10 | 2.27 | 0.000272 |
| Postn | periostin, osteoblast specific factor | 74.85 | 1044.76 | 13.96 | 0.023501 |
| Rdh10 | Retinol dehydrogenase 10 (all-trans) | 188.46 | 619.83 | 3.29 | 0.011156 |
|  | Rn.13512.1 | 85.48 | 1280.94 | 14.98 | 0.001033 |
| Nagk | N-acetylglucosamine kinase | 134.65 | 262.91 | 1.95 | 0.000047 |
|  | Rn.13860.1 | 192.67 | 353.26 | 1.83 | 0.000019 |
| Ipo5 | importin 5 | 309.15 | 566.35 | 1.83 | 0.000205 |
| Il33 | interleukin 33 | 624.99 | 1072.49 | 1.72 | 0.001816 |
| Inmt | indolethylamine N-methyltransferase | 45.71 | 116.40 | 2.55 | 0.015360 |
|  | Rn.17813.1 | 170.05 | 354.57 | 2.09 | 0.003880 |
|  | Rn.20493.1 | 95.52 | 50.24 | -1.90 | 0.001049 |
| Slc39a14 | solute carrier family 39 (zinc transporter), member 14 | 221.46 | 417.11 | 1.88 | 0.015240 |
| Pnmal2 | PNMA-like 2 | 1121.44 | 439.91 | -2.55 | 0.000276 |
|  | Rn.20036.1 | 489.14 | 1249.18 | 2.55 | 0.001980 |
| Kcnip2 | Kv channel-interacting protein 2 | 153.44 | 70.48 | -2.18 | 0.003036 |
| MGC108823 | similar to interferon-inducible GTPase | 152.37 | 829.31 | 5.44 | 0.001651 |
| Abcg1 | ATP-binding cassette, sub-family G (WHITE), member 1 | 68.23 | 126.85 | 1.86 | 0.001124 |
| LOC100365118 | rCG54747-like | 65.97 | 150.73 | 2.28 | 0.000462 |
| Psmb10 | proteasome (prosome, macropain) subunit, beta type 10 | 252.99 | 592.29 | 2.34 | 0.002747 |
| Rem2 | RAS (RAD and GEM) like GTP binding 2 | 31.87 | 11.82 | -2.70 | 0.019055 |
| Mcm2 | minichromosome maintenance complex component 2 | 94.91 | 279.11 | 2.94 | 0.002166 |
| Hs3st2 | heparan sulfate (glucosamine) 3-O-sulfotransferase 2 | 128.48 | 57.50 | -2.23 | 0.008456 |
| LOC100359980 | smooth muscle and non-muscle myosin alkali light chain 6B-like | 54.97 | 12.48 | -4.40 | 0.002352 |
| Erf | Ets2 repressor factor | 212.45 | 432.76 | 2.04 | 0.000138 |
| Gpx2 | glutathione peroxidase 2 | 167.59 | 1267.89 | 7.57 | 0.007010 |
| Dcun1d4 | DCN1, defective in cullin neddylation 1, domain containing 4 (S. cerevisiae) | 1219.43 | 753.53 | -1.62 | 0.000016 |
|  | Rn.46408.1 | 235.29 | 79.56 | -2.96 | 0.000104 |
|  | Rn.35282.2 | 619.06 | 286.78 | -2.16 | 0.000050 |
| Higd1a | HIG1 hypoxia inducible domain family, member 1A | 443.99 | 268.30 | -1.65 | 0.000735 |
|  | Rn.11601.1 | 1223.29 | 549.94 | -2.22 | 0.000005 |
| Baz1a | bromodomain adjacent to zinc finger domain, 1A | 128.55 | 327.66 | 2.55 | 0.001322 |
| Fbxl15 | F-box and leucine-rich repeat protein 15 | 219.06 | 110.94 | -1.97 | 0.000216 |
| Baiap2 | BAI1-associated protein 2 | 94.89 | 258.50 | 2.72 | 0.008055 |
| Elk3 | ELK3, member of ETS oncogene family | 130.64 | 342.56 | 2.62 | 0.000093 |
|  | Rn.1245.1 | 187.16 | 447.43 | 2.39 | 0.000024 |
| Cdr2 | cerebellar degeneration-related 2 | 654.74 | 313.96 | -2.09 | 0.000103 |
| RGD1565457 | similar to Rasa4 protein | 22.69 | 90.22 | 3.98 | 0.001151 |
|  | Rn.15453.1 | 600.67 | 317.67 | -1.89 | 0.000625 |
| RGD1308059 | similar to DNA segment, Chr 4, Brigham & Womens Genetics 0951 expressed | 271.70 | 145.27 | -1.87 | 0.021244 |
| Txndc11 | thioredoxin domain containing 11 | 123.96 | 308.27 | 2.49 | 0.000068 |
|  | Rn.24718.1 | 1444.62 | 746.63 | -1.93 | 0.000354 |
| Cd276 | Cd276 molecule | 112.44 | 263.57 | 2.34 | 0.003858 |
| Slc29a3 | solute carrier family 29 (nucleoside transporters), member 3 | 299.75 | 657.60 | 2.19 | 0.000169 |
| Purb | purine rich element binding protein B | 173.67 | 314.35 | 1.81 | 0.000320 |
| Slc29a3 | Solute carrier family 29 (nucleoside transporters), member 3 | 468.69 | 908.06 | 1.94 | 0.000133 |
| Slc22a18 | solute carrier family 22, member 18 | 93.38 | 173.83 | 1.86 | 0.004230 |
| Col7a1 | procollagen, type VII, alpha 1 | 13.84 | 64.29 | 4.64 | 0.000676 |
|  | Rn.26644.1 | 1111.22 | 451.68 | -2.46 | 0.000109 |
|  | Rn.43844.1 | 913.63 | 301.49 | -3.03 | 0.000048 |
| LOC100363145 | stabilin 1 | 44.50 | 634.31 | 14.25 | 0.003902 |
|  | Rn.51434.1 | 110.69 | 215.50 | 1.95 | 0.000716 |
|  | Rn.32615.1 | 556.78 | 304.31 | -1.83 | 0.000018 |
|  | Rn.27616.1 | 1096.28 | 471.81 | -2.32 | 0.000067 |
| Fitm2 | fat storage-inducing transmembrane protein 2 | 444.32 | 261.85 | -1.70 | 0.000232 |
| Cbln2 | cerebellin 2 precursor | 252.72 | 135.79 | -1.86 | 0.001491 |
| Rassf4 | Ras association (RalGDS/AF-6) domain family member 4 | 313.36 | 619.79 | 1.98 | 0.012961 |
| Zdhhc18 | zinc finger, DHHC-type containing 18 | 142.05 | 426.98 | 3.01 | 0.002442 |
|  | Rn.20014.1 | 795.54 | 360.09 | -2.21 | 0.000010 |
| Pik3r6 | Phosphoinositide-3-kinase, regulatory subunit 6 | 21.41 | 90.26 | 4.22 | 0.000266 |
| F5 | coagulation factor V (proaccelerin, labile factor) | 16.89 | 149.21 | 8.83 | 0.022265 |
|  | Rn.15595.1 | 920.09 | 418.53 | -2.20 | 0.000003 |
|  | Rn.8448.1 | 21.87 | 74.51 | 3.41 | 0.010052 |
| Rnf213 | ring finger protein 213 | 141.53 | 315.34 | 2.23 | 0.000401 |
| LOC100363739 | plasticity-related protein PRG-2-like /// lipid phosphate phosphatase-related protein type 3 | 286.25 | 108.93 | -2.63 | 0.000994 |
| Psca | prostate stem cell antigen | 12.82 | 39.32 | 3.07 | 0.029673 |
| Fam113b | family with sequence similarity 113, member B | 61.87 | 152.09 | 2.46 | 0.000337 |
| Aifm3 | apoptosis-inducing factor, mitochondrion-associated 3 | 327.15 | 152.06 | -2.15 | 0.000389 |
| Slc39a4 | solute carrier family 39 (zinc transporter), member 4 | 22.68 | 52.24 | 2.30 | 0.022136 |
|  | Rn.15631.1 | 299.99 | 146.97 | -2.04 | 0.000154 |
|  | Rn.2569.1 | 837.27 | 394.06 | -2.12 | 0.004867 |
| Gucy1b3 | guanylate cyclase 1, soluble, beta 3 | 881.25 | 462.69 | -1.90 | 0.011236 |
| Ehd4 | EH-domain containing 4 | 148.35 | 442.42 | 2.98 | 0.000777 |
| Snx2 | sorting nexin 2 | 935.36 | 1734.82 | 1.85 | 0.000144 |
| Efnb1 | ephrin B1 | 72.49 | 179.78 | 2.48 | 0.006074 |
| RGD1562952 | similar to Erbb2 interacting protein isoform 2 | 1338.32 | 2301.85 | 1.72 | 0.000075 |
|  | Rn.21031.2 | 490.97 | 254.74 | -1.93 | 0.000380 |
|  | Rn.13802.1 | 274.79 | 526.28 | 1.92 | 0.000792 |
| Gpr162 | G protein-coupled receptor 162 | 248.32 | 109.06 | -2.28 | 0.000402 |
| Cdca3 | cell division cycle associated 3 | 20.68 | 384.18 | 18.58 | 0.003405 |
|  | Rn.12980.1 | 446.65 | 1023.43 | 2.29 | 0.003852 |
|  | Rn.34966.1 | 851.84 | 444.70 | -1.92 | 0.000270 |
| Myd88 | myeloid differentiation primary response gene 88 | 238.34 | 907.55 | 3.81 | 0.000002 |
| Cpne8 | copine VIII | 234.65 | 559.70 | 2.39 | 0.000256 |
| Prrx2 | paired related homeobox 2 | 145.93 | 291.24 | 2.00 | 0.000466 |
| Fam96a | family with sequence similarity 96, member A | 637.91 | 1312.84 | 2.06 | 0.000330 |
|  | Rn.19441.1 | 167.15 | 607.20 | 3.63 | 0.000446 |
| Dram2 | DNA-damage regulated autophagy modulator 2 | 230.91 | 583.39 | 2.53 | 0.000809 |
| Chsy1 | chondroitin sulfate synthase 1 | 353.88 | 1075.15 | 3.04 | 0.000946 |
|  | Rn.8188.1 | 110.86 | 342.01 | 3.09 | 0.004858 |
| Cdca7 | cell division cycle associated 7 | 59.50 | 169.55 | 2.85 | 0.001405 |
| MGC112715 | platelet receptor Gi24 | 255.25 | 765.04 | 3.00 | 0.000003 |
| Mknk1 | MAP kinase-interacting serine/threonine kinase 1 | 141.50 | 265.51 | 1.88 | 0.000225 |
| Ifi35 | interferon-induced protein 35 | 81.05 | 308.10 | 3.80 | 0.000045 |
|  | Rn.17428.1 | 76.65 | 267.59 | 3.49 | 0.000030 |
| Prrc1 | proline-rich coiled-coil 1 | 71.82 | 136.19 | 1.90 | 0.000810 |
|  | Rn.2943.1 | 43.98 | 157.75 | 3.59 | 0.000140 |
| Dtx2 | deltex homolog 2 (Drosophila) | 110.64 | 219.18 | 1.98 | 0.001132 |
| Nek6 | NIMA (never in mitosis gene a)-related kinase 6 | 735.67 | 1959.13 | 2.66 | 0.000007 |
| Agpat2 | 1-acylglycerol-3-phosphate O-acyltransferase 2 (lysophosphatidic acid acyltransferase, beta) | 36.14 | 127.92 | 3.54 | 0.000474 |
| Kctd9 | potassium channel tetramerisation domain containing 9 | 829.11 | 460.13 | -1.80 | 0.000068 |
| Sumf1 | sulfatase modifying factor 1 | 686.66 | 1119.82 | 1.63 | 0.000023 |
| Prkce | protein kinase C, epsilon | 860.00 | 341.76 | -2.52 | 0.012694 |
| LOC363060 | similar to RIKEN cDNA 1600029D21 | 15.67 | 120.51 | 7.69 | 0.016664 |
| Hist1h4b | histone cluster 1, H4b | 145.98 | 530.35 | 3.63 | 0.000058 |
| Ifngr2 | interferon gamma receptor 2 | 550.74 | 1393.09 | 2.53 | 0.000003 |
|  | Rn.16122.1 | 397.34 | 788.19 | 1.98 | 0.000382 |
| Lrg1 | leucine-rich alpha-2-glycoprotein 1 | 32.84 | 146.58 | 4.46 | 0.004151 |
|  | Rn.35673.1 | 112.86 | 302.42 | 2.68 | 0.000261 |
| LOC689663 | Hypothetical protein LOC689663 | 2206.57 | 1116.24 | -1.98 | 0.000012 |
| Them4 | thioesterase superfamily member 4 | 257.54 | 138.20 | -1.86 | 0.000167 |
| Lmtk2 | lemur tyrosine kinase 2 | 606.69 | 334.35 | -1.81 | 0.000124 |
| Rasgrp2 | RAS guanyl releasing protein 2 (calcium and DAG-regulated) | 417.74 | 145.79 | -2.87 | 0.000286 |
| Fam73b | family with sequence similarity 73, member B | 441.59 | 260.55 | -1.69 | 0.000673 |
|  | Rn.47673.1 | 1040.71 | 2699.07 | 2.59 | 0.002021 |
| Arpp-21 | cyclic AMP-regulated phosphoprotein | 280.96 | 110.52 | -2.54 | 0.000053 |
| Aldh16a1 | aldehyde dehydrogenase 16 family, member A1 | 178.48 | 347.91 | 1.95 | 0.000472 |
|  | Rn.23465.1 | 514.36 | 294.72 | -1.75 | 0.000051 |
| Slc30a7 | solute carrier family 30 (zinc transporter), member 7 | 53.46 | 172.68 | 3.23 | 0.000068 |
|  | Rn.22473.1 | 121.54 | 256.30 | 2.11 | 0.000574 |
| LOC687508 | similar to Cytochrome c oxidase polypeptide VIIa-heart, mitochondrial precursor (Cytochrome c oxidase subunit VIIa-H) (COX VIIa-M) | 113.77 | 57.06 | -1.99 | 0.005173 |
| Dtx3l | deltex 3-like (Drosophila) | 164.00 | 534.02 | 3.26 | 0.000837 |
| Mov10 | Moloney leukemia virus 10 | 44.55 | 102.90 | 2.31 | 0.001653 |
| Tyrobp | Tyro protein tyrosine kinase binding protein | 747.33 | 3702.23 | 4.95 | 0.000002 |
|  | Rn.22621.1 | 309.50 | 860.10 | 2.78 | 0.007871 |
| LOC680254 | hypothetical protein LOC680254 | 287.47 | 488.22 | 1.70 | 0.000423 |
| Arhgap4 | Rho GTPase activating protein 4 | 34.71 | 113.00 | 3.26 | 0.019646 |
| Cdca4 | cell division cycle associated 4 | 87.47 | 178.26 | 2.04 | 0.001533 |
| Inadl2 | InaD-like 2 (Drosophila) | 270.16 | 119.83 | -2.25 | 0.000579 |
| Shmt1 | serine hydroxymethyltransferase 1 (soluble) | 67.29 | 166.88 | 2.48 | 0.000818 |
| Mdfic | MyoD family inhibitor domain containing | 248.55 | 884.92 | 3.56 | 0.000272 |
|  | Rn.30593.1 | 14.47 | 42.17 | 2.91 | 0.010039 |
| Asah1 | N-acylsphingosine amidohydrolase (acid ceramidase) 1 | 1445.70 | 2484.42 | 1.72 | 0.000317 |
| Serp2 | stress-associated endoplasmic reticulum protein family member 2 | 173.27 | 70.58 | -2.46 | 0.000949 |
| Mki67 | antigen identified by monoclonal antibody Ki-67 | 90.12 | 584.11 | 6.48 | 0.000633 |
|  | Rn.8136.1 | 124.93 | 1380.65 | 11.05 | 0.000548 |
|  | Rn.23559.1 | 412.74 | 223.38 | -1.85 | 0.000054 |
| Kif15 | kinesin family member 15 | 27.83 | 89.58 | 3.22 | 0.003393 |
| Ncapd2 | non-SMC condensin I complex, subunit D2 | 135.99 | 285.06 | 2.10 | 0.008514 |
| Haus4 | HAUS augmin-like complex, subunit 4 | 193.46 | 382.78 | 1.98 | 0.000462 |
| Dscc1 | defective in sister chromatid cohesion 1 homolog (S. cerevisiae) | 34.34 | 94.87 | 2.76 | 0.000407 |
| Ndst2 | N-deacetylase/N-sulfotransferase (heparan glucosaminyl) 2 | 197.11 | 349.21 | 1.77 | 0.000384 |
| Pdia5 | protein disulfide isomerase family A, member 5 | 79.31 | 202.64 | 2.55 | 0.000765 |
| Athl1 | ATH1, acid trehalase-like 1 (yeast) | 74.28 | 219.57 | 2.96 | 0.000333 |
|  | Rn.24673.1 | 556.22 | 257.47 | -2.16 | 0.000087 |
| Sp140 | SP140 nuclear body protein | 54.12 | 328.61 | 6.07 | 0.003669 |
| Adamts7 | ADAM metallopeptidase with thrombospondin type 1 motif, 7 | 93.73 | 194.50 | 2.08 | 0.000515 |
|  | Rn.39113.1 | 490.42 | 163.57 | -3.00 | 0.000033 |
| Phyhip | phytanoyl-CoA 2-hydroxylase interacting protein | 267.54 | 101.92 | -2.62 | 0.000011 |
|  | Rn.7551.1 | 408.74 | 172.63 | -2.37 | 0.000198 |
| Mtmr7 | myotubularin related protein 7 | 762.76 | 390.85 | -1.95 | 0.000064 |
| Iqgap3 | IQ motif containing GTPase activating protein 3 | 21.46 | 103.79 | 4.84 | 0.000471 |
| Gcnt2 | glucosaminyl (N-acetyl) transferase 2, I-branching enzyme | 135.94 | 278.45 | 2.05 | 0.001143 |
| Kif2c | kinesin family member 2C | 6.27 | 120.35 | 19.20 | 0.006305 |
|  | Rn.45356.1 | 15.61 | 44.27 | 2.84 | 0.004956 |
| Ccdc126 | coiled-coil domain containing 126 | 333.99 | 167.77 | -1.99 | 0.001347 |
|  | Rn.66580.1 | 564.03 | 243.68 | -2.31 | 0.001013 |
| Amdhd2 | amidohydrolase domain containing 2 | 66.03 | 334.91 | 5.07 | 0.001029 |
| Tmem106a | transmembrane protein 106A | 27.43 | 163.76 | 5.97 | 0.000769 |
| Pmf1 | polyamine-modulated factor 1 | 32.57 | 84.95 | 2.61 | 0.022521 |
|  | Rn.15248.1 | 105.46 | 45.73 | -2.31 | 0.001921 |
| Dcx | doublecortin | 129.50 | 67.32 | -1.92 | 0.001740 |
| Soat1 | Sterol O-acyltransferase 1 | 152.42 | 770.70 | 5.06 | 0.000990 |
| Map3k11 | mitogen-activated protein kinase kinase kinase 11 | 135.80 | 267.59 | 1.97 | 0.000428 |
|  | Rn.63586.1 | 211.80 | 110.10 | -1.92 | 0.000098 |
|  | Rn.13120.1 | 276.66 | 679.38 | 2.46 | 0.000481 |
|  | Rn.23897.1 | 172.81 | 86.48 | -2.00 | 0.002898 |
| Cd68 | Cd68 molecule | 51.41 | 2826.37 | 54.98 | 0.000051 |
| Pdzd4 | PDZ domain containing 4 | 253.87 | 119.69 | -2.12 | 0.001796 |
| Rin3 | Ras and Rab interactor 3 | 128.29 | 495.73 | 3.86 | 0.000011 |
|  | Rn.22936.1 | 58.09 | 126.03 | 2.17 | 0.039276 |
| B3galt5 | UDP-Gal:betaGlcNAc beta 1,3-galactosyltransferase, polypeptide 5 | 1758.54 | 959.38 | -1.83 | 0.000100 |
| Pla2g15 | phospholipase A2, group XV | 186.23 | 720.09 | 3.87 | 0.001125 |
| Fos | FBJ osteosarcoma oncogene | 444.55 | 1002.52 | 2.26 | 0.035654 |
| RGD1563319 | similar to RIKEN cDNA 6330512M04 gene | 690.19 | 363.40 | -1.90 | 0.001395 |
| Slc25a39 | solute carrier family 25, member 39 | 647.11 | 1097.24 | 1.70 | 0.000044 |
| S100a11 | S100 calcium binding protein A11 (calizzarin) | 118.06 | 1402.74 | 11.88 | 0.007999 |
|  | Rn.8470.1 | 1420.76 | 866.55 | -1.64 | 0.000039 |
|  | Rn.22997.1 | 10.96 | 34.37 | 3.14 | 0.047320 |
| Osbpl11 | oxysterol binding protein-like 11 | 150.47 | 337.23 | 2.24 | 0.000508 |
| Rnaset2 | ribonuclease T2 | 1361.63 | 5378.44 | 3.95 | 0.000021 |
| LOC100360710 | ribosomal protein S2-like /// similar to 40S ribosomal protein S2 /// ribosomal protein S2 | 3566.43 | 6302.32 | 1.77 | 0.000002 |
| Tmx4 | thioredoxin-related transmembrane protein 4 | 2811.10 | 1663.79 | -1.69 | 0.001088 |
| RGD1563556 | similar to mKIAA1377 protein | 52.37 | 26.30 | -1.99 | 0.012487 |
| Ppic | peptidylprolyl isomerase C | 57.20 | 311.11 | 5.44 | 0.004347 |
|  | Rn.34330.1 | 103.40 | 205.33 | 1.99 | 0.000261 |
|  | Rn.17191.1 | 574.48 | 1025.40 | 1.78 | 0.001028 |
| Trim2 | tripartite motif-containing 2 | 1902.57 | 794.24 | -2.40 | 0.008946 |
| Snhg11 | small nucleolar RNA host gene 11 | 2733.09 | 1262.40 | -2.16 | 0.002564 |
|  | Rn.61632.1 | 226.06 | 102.86 | -2.20 | 0.013364 |
|  | Rn.63527.1 | 54.47 | 21.09 | -2.58 | 0.009013 |
|  | Rn.4288.1 | 781.81 | 1358.16 | 1.74 | 0.002650 |
| Cnpy2 | canopy 2 homolog (zebrafish) | 770.76 | 1304.29 | 1.69 | 0.000210 |
| RGD1563941 | similar to hypothetical protein FLJ20010 | 107.12 | 299.29 | 2.79 | 0.000878 |
| Pdlim2 | PDZ and LIM domain 2 | 79.25 | 151.58 | 1.91 | 0.003234 |
| Creg1 | cellular repressor of E1A-stimulated genes 1 | 430.01 | 870.71 | 2.02 | 0.015507 |
| Disp2 | dispatched homolog 2 (Drosophila) | 843.99 | 417.59 | -2.02 | 0.000065 |
| Naprt1 | nicotinate phosphoribosyltransferase domain containing 1 | 291.16 | 642.77 | 2.21 | 0.000093 |
|  | Rn.22083.1 | 888.36 | 380.60 | -2.33 | 0.000131 |
| Mapre1 | microtubule-associated protein, RP/EB family, member 1 | 692.00 | 1265.05 | 1.83 | 0.001212 |
| LOC686323 | similar to thyroid receptor-interacting protein 6 | 107.05 | 300.15 | 2.80 | 0.000877 |
| Agtrap | angiotensin II receptor-associated protein | 60.79 | 136.93 | 2.25 | 0.004194 |
| Ccdc92 | coiled-coil domain containing 92 | 723.30 | 334.44 | -2.16 | 0.000034 |
| Zzef1 | Zinc finger, ZZ-type with EF hand domain 1 | 6.52 | 17.88 | 2.74 | 0.016391 |
| Camk2b | calcium/calmodulin-dependent protein kinase II beta | 1501.96 | 696.41 | -2.16 | 0.000071 |
| Zfhx4 | zinc finger homeobox 4 | 108.45 | 39.42 | -2.75 | 0.004891 |
| Sfxn5 | sideroflexin 5 | 904.21 | 411.67 | -2.20 | 0.011158 |
|  | Rn.63066.1 | 41.66 | 19.94 | -2.09 | 0.004678 |
| Clic1 | chloride intracellular channel 1 | 617.69 | 2683.55 | 4.34 | 0.000191 |
| Ccdc53 | coiled-coil domain containing 53 | 551.02 | 994.22 | 1.80 | 0.000010 |
| RGD1560286 | similar to DNA segment, Chr 4, ERATO Doi 22, expressed | 154.32 | 268.99 | 1.74 | 0.000842 |
|  | Rn.7207.1 | 211.08 | 92.31 | -2.29 | 0.002262 |
|  | Rn.62756.1 | 61.91 | 173.09 | 2.80 | 0.008217 |
|  | Rn.21687.1 | 419.28 | 142.88 | -2.93 | 0.000199 |
|  | Rn.9056.1 | 222.42 | 596.58 | 2.68 | 0.000118 |
|  | Rn.38680.1 | 155.52 | 72.35 | -2.15 | 0.000753 |
|  | Rn.23254.1 | 200.35 | 87.84 | -2.28 | 0.008627 |
| LOC100361913 | rCG54286-like | 86.38 | 234.79 | 2.72 | 0.014044 |
| Sipa1 | signal-induced proliferation-associated 1 | 183.45 | 626.54 | 3.42 | 0.000048 |
|  | Rn.41745.1 | 293.67 | 151.39 | -1.94 | 0.000971 |
| Ehd4 | EH-domain containing 4 | 411.06 | 959.06 | 2.33 | 0.032336 |
|  | Rn.50930.1 | 99.72 | 22.80 | -4.37 | 0.005187 |
| Jund | jun D proto-oncogene | 35.49 | 10.30 | -3.45 | 0.027979 |
|  | Rn.73068.1 | 164.80 | 81.31 | -2.03 | 0.004047 |
| Tmem121 | transmembrane protein 121 | 120.18 | 64.89 | -1.85 | 0.002013 |
|  | Rn.7551.2 | 57.69 | 19.88 | -2.90 | 0.016357 |
| Gpr61 | G protein-coupled receptor 61 | 53.57 | 11.39 | -4.70 | 0.018862 |
| Klhl8 | kelch-like 8 (Drosophila) | 402.52 | 212.33 | -1.90 | 0.000186 |
| Hmgcr | 3-hydroxy-3-methylglutaryl-Coenzyme A reductase | 1657.52 | 704.36 | -2.35 | 0.000006 |
|  | Rn.7450.1 | 1946.70 | 1031.05 | -1.89 | 0.000041 |
| Myof | myoferlin | 211.11 | 444.49 | 2.11 | 0.049920 |
| Rbms1 | RNA binding motif, single stranded interacting protein 1 | 772.90 | 1502.67 | 1.94 | 0.004027 |
|  | Rn.63144.1 | 242.20 | 104.13 | -2.33 | 0.014630 |
| RGD1559442 | similar to SET binding factor 2 | 19.68 | 50.14 | 2.55 | 0.021074 |
| Elavl1 | ELAV (embryonic lethal, abnormal vision, Drosophila)-like 1 (Hu antigen R) | 331.45 | 761.46 | 2.30 | 0.003062 |
| RGD1561067 | Similar to RNA binding protein gene with multiple splicing | 67.79 | 148.94 | 2.20 | 0.001665 |
| Lilrb4 | leukocyte immunoglobulin-like receptor, subfamily B, member 4 | 21.63 | 1060.97 | 49.05 | 0.006810 |
| Plekho1 | pleckstrin homology domain containing, family O member 1 | 111.68 | 221.93 | 1.99 | 0.000229 |
| Dsc2 | desmocollin 2 | 20.84 | 260.10 | 12.48 | 0.007327 |
| Cp110 | CP110 protein | 1319.32 | 758.22 | -1.74 | 0.000142 |
| Acss2 | acyl-CoA synthetase short-chain family member 2 | 2235.08 | 1026.04 | -2.18 | 0.000052 |
| Thbd | thrombomodulin | 340.81 | 722.52 | 2.12 | 0.019717 |
| Ube2k | ubiquitin-conjugating enzyme E2K (UBC1 homolog, yeast) | 526.42 | 300.10 | -1.75 | 0.000661 |
|  | Rn.7966.1 | 66.00 | 145.00 | 2.20 | 0.014927 |
| Ctnna2 | catenin (cadherin associated protein), alpha 2 | 845.06 | 443.87 | -1.90 | 0.000653 |
|  | Rn.24922.1 | 901.68 | 1582.69 | 1.76 | 0.000077 |
| Nxn | nucleoredoxin | 248.77 | 517.31 | 2.08 | 0.000012 |
| Mamdc2 | MAM domain containing 2 | 183.19 | 98.43 | -1.86 | 0.004361 |
| Zfhx4 | zinc finger homeobox 4 | 174.63 | 57.25 | -3.05 | 0.000749 |
| Nfkb2 | nuclear factor of kappa light polypeptide gene enhancer in B-cells 2, p49/p100 | 75.97 | 162.32 | 2.14 | 0.000828 |
| RGD1312005 | similar to DD1 | 717.16 | 361.81 | -1.98 | 0.000019 |
|  | Rn.21679.1 | 591.54 | 332.45 | -1.78 | 0.000574 |
|  | Rn.46830.1 | 213.47 | 497.46 | 2.33 | 0.000129 |
|  | Rn.27760.1 | 33.39 | 10.15 | -3.29 | 0.011931 |
| LOC361188 | similar to WD repeat domain 17 /// similar to WD repeat domain 17 | 62.27 | 28.10 | -2.22 | 0.008012 |
|  | Rn.22059.1 | 1659.77 | 748.04 | -2.22 | 0.000091 |
|  | Rn.11705.1 | 305.66 | 714.35 | 2.34 | 0.001820 |
| Aurka | aurora kinase A | 59.91 | 181.67 | 3.03 | 0.000335 |
| Mcm5 | minichromosome maintenance complex component 5 | 38.50 | 210.45 | 5.47 | 0.003464 |
| LOC100362108 | poly (ADP-ribose) polymerase family, member 10 | 45.44 | 130.48 | 2.87 | 0.000625 |
| Sdc1 | syndecan 1 | 4.68 | 74.73 | 15.98 | 0.003205 |
| Rab11fip4 | RAB11 family interacting protein 4 (class II) | 644.38 | 331.33 | -1.94 | 0.000823 |
| Espl1 | extra spindle pole bodies homolog 1 (S. cerevisiae) | 11.24 | 74.27 | 6.61 | 0.008779 |
|  | Rn.18386.1 | 1536.53 | 627.88 | -2.45 | 0.000029 |
| Tubb6 | tubulin, beta 6 | 167.31 | 924.96 | 5.53 | 0.001832 |
| Tmbim1 | transmembrane BAX inhibitor motif containing 1 | 1216.70 | 2038.93 | 1.68 | 0.000210 |
|  | Rn.37610.1 | 318.04 | 176.78 | -1.80 | 0.000159 |
|  | Rn.48053.1 | 80.41 | 378.62 | 4.71 | 0.004482 |
| Parp9 | poly (ADP-ribose) polymerase family, member 9 | 183.56 | 553.38 | 3.01 | 0.000270 |
|  | Rn.23992.1 | 114.58 | 47.72 | -2.40 | 0.001814 |
| S1pr3 | sphingosine-1-phosphate receptor 3 | 190.90 | 610.40 | 3.20 | 0.000471 |
| LOC100365106 | rCG32755-like | 329.83 | 1154.03 | 3.50 | 0.006790 |
|  | Rn.28740.1 | 1267.14 | 594.79 | -2.13 | 0.000070 |
| Slc24a3 | solute carrier family 24 (sodium/potassium/calcium exchanger), member 3 | 854.75 | 455.58 | -1.88 | 0.000358 |
| RGD1308544 | LOC361192 | 97.10 | 45.64 | -2.13 | 0.002288 |
|  | Rn.4292.1 | 408.84 | 218.01 | -1.88 | 0.000063 |
| Abcg4 | ATP-binding cassette, sub-family G (WHITE), member 4 | 221.56 | 99.91 | -2.22 | 0.000063 |
|  | Rn.13850.1 | 1027.68 | 522.81 | -1.97 | 0.000020 |
| Lynx1 | Ly6/neurotoxin 1 | 180.62 | 98.97 | -1.82 | 0.001202 |
| Kifc1 | kinesin family member C1 | 25.85 | 156.46 | 6.05 | 0.003165 |
|  | Rn.11848.1 | 56.39 | 121.49 | 2.15 | 0.024556 |
| MGC72974 | hypothetical LOC316976 | 95.28 | 187.14 | 1.96 | 0.000150 |
|  | Rn.45542.1 | 1398.30 | 828.32 | -1.69 | 0.000146 |
|  | Rn.26560.1 | 297.58 | 117.54 | -2.53 | 0.013822 |
| Rundc3b | RUN domain containing 3B | 348.94 | 139.17 | -2.51 | 0.000014 |
| March11 | membrane-associated ring finger (C3HC4) 11 | 125.19 | 60.10 | -2.08 | 0.000183 |
| Sdr39u1 | short chain dehydrogenase/reductase family 39U, member 1 | 191.58 | 95.08 | -2.01 | 0.013425 |
| Elfn2 | Extracellular leucine-rich repeat and fibronectin type III domain containing 2 | 689.85 | 366.95 | -1.88 | 0.000591 |
| Gins1 | GINS complex subunit 1 (Psf1 homolog) | 54.88 | 121.55 | 2.21 | 0.001789 |
|  | Rn.45164.1 | 819.30 | 334.51 | -2.45 | 0.000017 |
|  | Rn.51226.1 | 189.93 | 96.97 | -1.96 | 0.002201 |
| LOC100365889 | KIAA1440-like | 184.60 | 318.73 | 1.73 | 0.000843 |
| Map4k1 | mitogen activated protein kinase kinase kinase kinase 1 | 26.20 | 95.14 | 3.63 | 0.000114 |
| LOC691289 | similar to serine (or cysteine) proteinase inhibitor, clade B, member 1a | 24.75 | 64.18 | 2.59 | 0.002705 |
|  | Rn.24686.1 | 463.72 | 238.63 | -1.94 | 0.000622 |
|  | Rn.27171.1 | 867.43 | 400.77 | -2.16 | 0.000228 |
|  | Rn.27890.1 | 139.89 | 56.84 | -2.46 | 0.002352 |
| LOC100361007 | rCG32064-like | 35.12 | 161.91 | 4.61 | 0.001592 |
| Arrdc1 | arrestin domain containing 1 | 106.38 | 231.67 | 2.18 | 0.000650 |
|  | Rn.19720.1 | 492.19 | 194.89 | -2.53 | 0.001269 |
|  | Rn.21810.1 | 75.24 | 245.81 | 3.27 | 0.002420 |
|  | Rn.46109.1 | 709.00 | 384.17 | -1.85 | 0.003219 |
| Ntng1 | netrin G1 | 160.37 | 77.03 | -2.08 | 0.001455 |
| Tpcn2 | two pore segment channel 2 | 69.79 | 201.51 | 2.89 | 0.000430 |
| Edem2 | ER degradation enhancer, mannosidase alpha-like 2 | 147.41 | 306.77 | 2.08 | 0.000162 |
|  | Rn.8449.1 | 275.40 | 126.58 | -2.18 | 0.002119 |
| Fam38a | family with sequence similarity 38, member A | 33.43 | 232.38 | 6.95 | 0.004140 |
| Tnfrsf14 | tumor necrosis factor receptor superfamily, member 14 (herpesvirus entry mediator) | 24.58 | 263.75 | 10.73 | 0.005281 |
| RGD1310819 | similar to putative protein (5S487) | 106.26 | 48.10 | -2.21 | 0.030563 |
|  | Rn.38226.1 | 469.24 | 242.57 | -1.93 | 0.002437 |
| Caly | calcyon neuron-specific vesicular protein | 613.40 | 311.47 | -1.97 | 0.000860 |
|  | Rn.47356.1 | 352.87 | 157.76 | -2.24 | 0.005459 |
| Bin2 | bridging integrator 2 | 28.37 | 173.52 | 6.12 | 0.012643 |
|  | Rn.62402.1 | 96.06 | 41.10 | -2.34 | 0.012300 |
| Cuedc2 | CUE domain containing 2 | 142.70 | 378.92 | 2.66 | 0.002677 |
| Diaph3 | diaphanous homolog 3 (Drosophila) | 20.62 | 63.12 | 3.06 | 0.004303 |
|  | Rn.62287.1 | 157.39 | 34.82 | -4.52 | 0.000192 |
| Ms4a11 | membrane-spanning 4-domains, subfamily A, member 11 | 19.72 | 273.43 | 13.87 | 0.001427 |
| Clec9a | C-type lectin domain family 9, member a | 11.44 | 31.40 | 2.75 | 0.024046 |
|  | Rn.15761.1 | 388.55 | 141.07 | -2.75 | 0.000047 |
|  | Rn.50887.1 | 79.07 | 24.37 | -3.24 | 0.003060 |
|  | Rn.28067.1 | 388.54 | 226.76 | -1.71 | 0.000546 |
|  | Rn.15539.1 | 1432.48 | 628.55 | -2.28 | 0.000005 |
| RGD1565772 | similar to hypothetical protein A430110N23 | 68.14 | 125.55 | 1.84 | 0.001911 |
| Aak1 | AP2 associated kinase 1 | 1335.20 | 647.49 | -2.06 | 0.000024 |
| Hrnbp3 | hexaribonucleotide binding protein 3 | 277.37 | 111.33 | -2.49 | 0.000380 |
|  | Rn.17336.1 | 299.07 | 152.58 | -1.96 | 0.002682 |
|  | Rn.62947.1 | 46.87 | 17.23 | -2.72 | 0.002477 |
|  | Rn.60823.1 | 58.03 | 24.10 | -2.41 | 0.011406 |
|  | Rn.23400.1 | 610.83 | 231.47 | -2.64 | 0.005546 |
|  | Rn.12470.1 | 233.61 | 96.56 | -2.42 | 0.000237 |
| Arhgap8 | Rho GTPase activating protein 8 | 2.61 | 28.65 | 10.99 | 0.029394 |
| Flywch1 | FLYWCH-type zinc finger 1 | 247.99 | 95.78 | -2.59 | 0.002960 |
| Ptpn3 | Protein tyrosine phosphatase, non-receptor type 3 | 60.30 | 30.99 | -1.95 | 0.012033 |
| RGD1565432 | similar to hypothetical protein | 958.42 | 488.02 | -1.96 | 0.000037 |
|  | Rn.58248.1 | 18.31 | 64.26 | 3.51 | 0.020116 |
| Sptbn4 | spectrin, beta, non-erythrocytic 4 | 318.91 | 117.79 | -2.71 | 0.000519 |
| Pde4a | Phosphodiesterase 4A, cAMP-specific (phosphodiesterase E2 dunce homolog, Drosophila) | 339.68 | 194.00 | -1.75 | 0.000330 |
| Klf2 | Kruppel-like factor 2 (lung) | 261.21 | 583.73 | 2.23 | 0.001431 |
|  | Rn.36337.3 | 353.63 | 785.31 | 2.22 | 0.002605 |
| Rab34 | RAB34, member RAS oncogene family | 266.90 | 533.88 | 2.00 | 0.000621 |
|  | Rn.17490.1 | 34.53 | 128.27 | 3.72 | 0.011204 |
| Dusp11 | dual specificity phosphatase 11 (RNA/RNP complex 1-interacting) | 134.69 | 301.48 | 2.24 | 0.002250 |
|  | Rn.6483.1 | 296.55 | 664.92 | 2.24 | 0.004460 |
|  | Rn.17606.1 | 477.02 | 227.45 | -2.10 | 0.000590 |
| Tmem179b | transmembrane protein 179B | 246.51 | 624.21 | 2.53 | 0.000009 |
| Atad2 | ATPase family, AAA domain containing 2 | 122.94 | 361.65 | 2.94 | 0.001398 |
| Pola1 | polymerase (DNA directed), alpha 1 | 81.01 | 173.36 | 2.14 | 0.009478 |
| LOC691862 | Hypothetical protein LOC691862 | 50.90 | 137.58 | 2.70 | 0.000114 |
| Lmcd1 | LIM and cysteine-rich domains 1 | 130.12 | 304.12 | 2.34 | 0.000322 |
| Tgfbr1 | transforming growth factor, beta receptor 1 | 1375.30 | 3839.47 | 2.79 | 0.000047 |
| C1qa | complement component 1, q subcomponent, A chain | 563.72 | 4752.37 | 8.43 | 0.000065 |
| Socs6 | suppressor of cytokine signaling 6 | 259.98 | 479.61 | 1.84 | 0.001406 |
|  | Rn.11988.1 | 35.32 | 757.87 | 21.46 | 0.000510 |
| Dlgap5 | discs, large (Drosophila) homolog-associated protein 5 | 165.60 | 336.36 | 2.03 | 0.002986 |
| LOC685020 | Similar to paired immunoglobin-like type 2 receptor alpha | 106.75 | 582.64 | 5.46 | 0.002178 |
| Lima1 | LIM domain and actin binding 1 | 530.15 | 999.97 | 1.89 | 0.004234 |
| Fam115c | Family with sequence similarity 115, member C | 60.16 | 173.71 | 2.89 | 0.000276 |
| Slc39a8 | solute carrier family 39 (zinc transporter), member 8 | 81.68 | 294.61 | 3.61 | 0.001336 |
|  | Rn.49289.1 | 100.06 | 274.98 | 2.75 | 0.009031 |
|  | Rn.12712.1 | 359.54 | 162.38 | -2.21 | 0.000065 |
|  | Rn.23529.1 | 31.56 | 291.12 | 9.22 | 0.000635 |
|  | Rn.37063.1 | 1186.35 | 492.12 | -2.41 | 0.000066 |
| Hook3 | hook homolog 3 (Drosophila) | 123.47 | 65.84 | -1.88 | 0.002561 |
|  | Rn.13859.1 | 54.90 | 145.73 | 2.65 | 0.043032 |
| Rarb | Retinoic acid receptor, beta | 188.47 | 383.16 | 2.03 | 0.010634 |
| Mro | maestro | 419.98 | 225.08 | -1.87 | 0.000949 |
| Fam103a1 | family with sequence similarity 103, member A1 | 365.16 | 190.85 | -1.91 | 0.000460 |
|  | Rn.10412.1 | 384.22 | 181.27 | -2.12 | 0.001438 |
| RGD1310773 | similar to hypothetical protein FLJ31810 | 210.98 | 62.70 | -3.36 | 0.000158 |
|  | Rn.30608.1 | 475.47 | 973.22 | 2.05 | 0.000200 |
| Pik3ap1 | phosphoinositide-3-kinase adaptor protein 1 | 93.40 | 523.82 | 5.61 | 0.000218 |
| Crlf1 | cytokine receptor-like factor 1 | 492.96 | 241.32 | -2.04 | 0.003964 |
|  | Rn.42977.1 | 88.01 | 39.31 | -2.24 | 0.002287 |
| Mphosph8 | M-phase phosphoprotein 8 /// poly (ADP-ribose) polymerase family, member 4 | 165.21 | 293.22 | 1.77 | 0.000526 |
| Lpcat3 | lysophosphatidylcholine acyltransferase 3 | 321.13 | 699.36 | 2.18 | 0.000073 |
|  | Rn.15992.1 | 28.52 | 81.31 | 2.85 | 0.007611 |
| Wtip | Wilms tumor 1 interacting protein | 66.01 | 185.78 | 2.81 | 0.000862 |
| Gprc5a | G protein-coupled receptor, family C, group 5, member A | 20.90 | 87.67 | 4.20 | 0.010505 |
|  | Rn.39005.1 | 250.28 | 106.23 | -2.36 | 0.001521 |
| Fam64a | family with sequence similarity 64, member A | 11.83 | 167.57 | 14.17 | 0.001604 |
|  | Rn.16238.1 | 209.70 | 491.94 | 2.35 | 0.000132 |
| Nfkbie | Nuclear factor of kappa light polypeptide gene enhancer in B-cells inhibitor, epsilon | 193.13 | 472.75 | 2.45 | 0.002340 |
| H6pd | hexose-6-phosphate dehydrogenase (glucose 1-dehydrogenase) | 39.14 | 113.84 | 2.91 | 0.000344 |
|  | Rn.3062.1 | 85.73 | 163.24 | 1.90 | 0.001988 |
| Ifi27l2b | interferon, alpha-inducible protein 27 like 2B | 363.23 | 1335.71 | 3.68 | 0.000769 |
| Ccl27 | chemokine (C-C motif) ligand 27 | 193.88 | 106.34 | -1.82 | 0.007328 |
| Nxn | Nucleoredoxin | 13.56 | 28.44 | 2.10 | 0.005842 |
|  | Rn.49261.1 | 45.00 | 16.66 | -2.70 | 0.005903 |
| LOC681196 | similar to paired immunoglobin-like type 2 receptor beta /// similar to paired immunoglobin-like type 2 receptor beta /// similar to cell surface receptor FDFACT /// similar to cell surface receptor FDFACT /// similar to cell surface receptor FDFACT /// similar to cell surface receptor FDFACT /// similar to paired immunoglobin-like type 2 receptor beta | 53.23 | 466.90 | 8.77 | 0.004090 |
|  | Rn.7829.1 | 80.33 | 178.79 | 2.23 | 0.002012 |
| Cbln1 | cerebellin 1 precursor | 398.37 | 129.84 | -3.07 | 0.002175 |
|  | Rn.46998.1 | 673.87 | 219.34 | -3.07 | 0.001228 |
|  | Rn.34212.1 | 161.37 | 511.74 | 3.17 | 0.001592 |
| Gria3 | glutamate receptor, ionotrophic, AMPA 3 | 998.32 | 448.04 | -2.23 | 0.000143 |
| Nrsn1 | neurensin 1 | 1514.25 | 736.14 | -2.06 | 0.000025 |
| Il16 | interleukin 16 | 53.17 | 168.39 | 3.17 | 0.000234 |
| Ifit3 | interferon-induced protein with tetratricopeptide repeats 3 | 70.61 | 315.64 | 4.47 | 0.000004 |
| Depdc1 | DEP domain containing 1 | 5.13 | 109.23 | 21.28 | 0.004470 |
| Mfsd6 | major facilitator superfamily domain containing 6 | 2372.17 | 1326.27 | -1.79 | 0.000059 |
| LOC500013 | similar to sterile alpha motif domain containing 9-like | 241.24 | 500.23 | 2.07 | 0.000109 |
|  | Rn.18692.1 | 39.58 | 16.61 | -2.38 | 0.013481 |
|  | Rn.23214.1 | 675.40 | 255.57 | -2.64 | 0.005396 |
| Slc24a6 | solute carrier family 24 (sodium/potassium/calcium exchanger), member 6 | 89.76 | 222.79 | 2.48 | 0.000079 |
| RGD1565927 | similar to 4631422O05Rik protein | 102.43 | 288.78 | 2.82 | 0.023328 |
|  | Rn.7907.1 | 57.78 | 212.32 | 3.67 | 0.000036 |
|  | Rn.25476.1 | 73.89 | 31.94 | -2.31 | 0.012333 |
|  | Rn.41821.1 | 573.57 | 292.44 | -1.96 | 0.000132 |
|  | Rn.23622.1 | 545.67 | 236.68 | -2.31 | 0.000090 |
|  | Rn.19771.1 | 14.15 | 57.63 | 4.07 | 0.002784 |
|  | Rn.46754.1 | 93.05 | 15.18 | -6.13 | 0.001703 |
| Stra6 | stimulated by retinoic acid gene 6 | 66.82 | 221.57 | 3.32 | 0.004655 |
| Dyrk2 | dual-specificity tyrosine-(Y)-phosphorylation regulated kinase 2 | 23.34 | 84.00 | 3.60 | 0.004324 |
| Ptger3 | Prostaglandin E receptor 3 | 22.76 | 66.80 | 2.94 | 0.029070 |
| Htr2c | 5-hydroxytryptamine (serotonin) receptor 2C | 1887.91 | 829.29 | -2.28 | 0.000012 |
| Lrrc49 | leucine rich repeat containing 49 | 488.82 | 212.68 | -2.30 | 0.000045 |
|  | Rn.38645.1 | 120.56 | 242.12 | 2.01 | 0.000060 |
| Nrcam | neuronal cell adhesion molecule | 578.95 | 346.19 | -1.67 | 0.000123 |
|  | Rn.8010.1 | 69.79 | 267.05 | 3.83 | 0.000016 |
|  | Rn.43686.1 | 24.73 | 72.63 | 2.94 | 0.009823 |
| Atp6v0a2 | ATPase, H+ transporting, lysosomal V0 subunit A2 | 107.02 | 191.60 | 1.79 | 0.000818 |
| Dusp2 | dual specificity phosphatase 2 | 26.74 | 133.62 | 5.00 | 0.000215 |
|  | Rn.27597.1 | 248.67 | 105.45 | -2.36 | 0.000168 |
| Serpinb1a | serine (or cysteine) proteinase inhibitor, clade B, member 1a | 472.81 | 2223.20 | 4.70 | 0.000003 |
|  | Rn.27870.1 | 355.08 | 165.46 | -2.15 | 0.000049 |
| Pnpla7 | patatin-like phospholipase domain containing 7 | 167.12 | 512.51 | 3.07 | 0.000083 |
|  | Rn.12240.1 | 52.62 | 27.44 | -1.92 | 0.006128 |
| LOC100366119 | Mpv17 transgene, kidney disease mutant-like (predicted)-like | 430.32 | 157.29 | -2.74 | 0.000380 |
|  | Rn.25232.1 | 37.01 | 72.39 | 1.96 | 0.003606 |
|  | Rn.41884.1 | 868.71 | 438.99 | -1.98 | 0.004476 |
| Slc37a2 | Solute carrier family 37 (glycerol-3-phosphate transporter), member 2 | 63.15 | 480.57 | 7.61 | 0.000005 |
| Car7 | carbonic anhydrase 7 | 93.23 | 30.03 | -3.10 | 0.000071 |
| C1qtnf3 | C1q and tumor necrosis factor related protein 3 | 79.62 | 27.66 | -2.88 | 0.001652 |
| Dock6 | dedicator of cytokinesis 6 | 204.79 | 368.26 | 1.80 | 0.002909 |
|  | Rn.41848.1 | 85.09 | 1088.70 | 12.79 | 0.003937 |
| Hrnbp3 | hexaribonucleotide binding protein 3 | 1172.53 | 405.75 | -2.89 | 0.000300 |
| Cox6b2 | cytochrome c oxidase subunit VIb polypeptide 2 | 44.79 | 101.29 | 2.26 | 0.002961 |
| Arl5c | ADP-ribosylation factor-like 5C | 36.89 | 432.77 | 11.73 | 0.002995 |
|  | Rn.3800.1 | 2439.89 | 1028.60 | -2.37 | 0.001072 |
| Rnasel | Ribonuclease L (2',5'-oligoisoadenylate synthetase-dependent) | 265.91 | 524.56 | 1.97 | 0.000105 |
| Dnajc6 | DnaJ (Hsp40) homolog, subfamily C, member 6 | 2452.06 | 1148.36 | -2.14 | 0.000090 |
|  | Rn.23617.1 | 497.41 | 252.58 | -1.97 | 0.000087 |
| Gpr98 | G protein-coupled receptor 98 /// similar to very large G-protein coupled receptor 1 | 436.83 | 228.66 | -1.91 | 0.001306 |
| Klhl6 | kelch-like 6 (Drosophila) | 55.31 | 271.34 | 4.91 | 0.000003 |
| Tcf7l2 | transcription factor 7-like 2 (T-cell specific, HMG-box) | 401.72 | 1094.03 | 2.72 | 0.001415 |
|  | Rn.19499.1 | 18.00 | 43.37 | 2.41 | 0.035909 |
|  | Rn.33757.1 | 575.37 | 290.09 | -1.98 | 0.020415 |
|  | Rn.23759.1 | 1728.12 | 1043.71 | -1.66 | 0.000122 |
| Apbb1ip | amyloid beta (A4) precursor protein-binding, family B, member 1 interacting protein | 453.61 | 1476.59 | 3.26 | 0.000002 |
| Gspt2 | G1 to S phase transition 2 | 279.24 | 159.84 | -1.75 | 0.000079 |
|  | Rn.19097.1 | 17.59 | 47.24 | 2.69 | 0.023926 |
|  | Rn.50824.1 | 179.58 | 88.04 | -2.04 | 0.000188 |
|  | Rn.20599.1 | 251.84 | 85.91 | -2.93 | 0.001034 |
| Magee1 | melanoma antigen, family E, 1 | 1953.57 | 978.94 | -2.00 | 0.000060 |
| Gimap4 | GTPase, IMAP family member 4 | 10.05 | 35.79 | 3.56 | 0.005514 |
| Lbxcor1 | LBXCOR1 homolog (mouse) | 115.62 | 48.21 | -2.40 | 0.000440 |
|  | Rn.2836.1 | 564.63 | 334.90 | -1.69 | 0.000118 |
|  | Rn.40264.1 | 275.09 | 491.73 | 1.79 | 0.002161 |
| Pgm2l1 | phosphoglucomutase 2-like 1 | 1071.97 | 580.38 | -1.85 | 0.000476 |
|  | Rn.18255.1 | 567.58 | 302.48 | -1.88 | 0.000334 |
| Tfpi2 | tissue factor pathway inhibitor 2 | 49.62 | 279.86 | 5.64 | 0.000770 |
|  | Rn.19629.1 | 682.30 | 282.62 | -2.41 | 0.000032 |
| Susd3 | sushi domain containing 3 | 55.91 | 131.46 | 2.35 | 0.000728 |
| Tnfsf13 | tumor necrosis factor (ligand) superfamily, member 13 | 368.93 | 697.23 | 1.89 | 0.002711 |
| Slc10a3 | solute carrier family 10 (sodium/bile acid cotransporter family), member 3 | 38.31 | 84.49 | 2.21 | 0.000707 |
|  | Rn.15176.1 | 356.92 | 162.84 | -2.19 | 0.000293 |
|  | Rn.66643.1 | 156.85 | 285.01 | 1.82 | 0.000646 |
| Cybrd1 | cytochrome b reductase 1 | 118.28 | 266.52 | 2.25 | 0.003390 |
|  | Rn.28718.1 | 428.87 | 213.75 | -2.01 | 0.000163 |
| Arhgap27 | Rho GTPase activating protein 27 | 67.01 | 171.56 | 2.56 | 0.000376 |
|  | Rn.45917.1 | 751.96 | 395.73 | -1.90 | 0.000002 |
| E2f8 | E2F transcription factor 8 | 27.30 | 71.49 | 2.62 | 0.007313 |
|  | Rn.22217.1 | 554.84 | 266.68 | -2.08 | 0.001901 |
| LOC363328 | Similar to TICAM-1 | 27.02 | 87.36 | 3.23 | 0.000321 |
| Dut | Deoxyuridine triphosphatase | 170.73 | 80.87 | -2.11 | 0.000761 |
| RGD1307648 | similar to CG13901-PA | 412.17 | 236.91 | -1.74 | 0.001307 |
| RGD1566401 | Similar to GTL2, imprinted maternally expressed untranslated | 1110.75 | 306.13 | -3.63 | 0.003423 |
|  | Rn.7661.1 | 801.40 | 372.46 | -2.15 | 0.000009 |
| Rprml | reprimo-like | 98.37 | 45.26 | -2.17 | 0.009984 |
| Oasl | 2'-5'-oligoadenylate synthetase-like | 37.57 | 188.83 | 5.03 | 0.000043 |
|  | Rn.61384.1 | 279.84 | 103.65 | -2.70 | 0.011031 |
| RGD1304737 | Similar to KIAA1086 protein | 228.36 | 107.83 | -2.12 | 0.000348 |
| LOC100363182 | UPF0585 protein C16orf13 homolog /// similar to CG18661-PA | 91.37 | 192.67 | 2.11 | 0.006884 |
|  | Rn.60793.1 | 41.57 | 135.76 | 3.27 | 0.010736 |
| Cd40 | CD40 molecule, TNF receptor superfamily member 5 | 39.63 | 182.23 | 4.60 | 0.001388 |
| Bid | BH3 interacting domain death agonist | 149.63 | 295.18 | 1.97 | 0.001457 |
|  | Rn.24387.1 | 362.29 | 128.15 | -2.83 | 0.000063 |
| Ccdc64 | coiled-coil domain containing 64 | 255.02 | 130.45 | -1.95 | 0.000151 |
| Dock8 | dedicator of cytokinesis 8 | 142.89 | 744.87 | 5.21 | 0.000069 |
| RGD1562284 | similar to Glutaminyl-peptide cyclotransferase precursor (QC) | 265.14 | 146.19 | -1.81 | 0.004383 |
| Arl11 | ADP-ribosylation factor-like 11 | 64.86 | 691.99 | 10.67 | 0.000120 |
| Cyba | cytochrome b-245, alpha polypeptide | 13.44 | 47.87 | 3.56 | 0.002364 |
| Ocln | occludin | 193.87 | 424.43 | 2.19 | 0.000198 |
| RGD1559690 | similar to hypothetical protein FLJ25416 | 8.18 | 29.40 | 3.59 | 0.012433 |
| Cfp | complement factor properdin | 47.17 | 309.34 | 6.56 | 0.000869 |
| Rbpj | recombination signal binding protein for immunoglobulin kappa J region | 193.45 | 705.30 | 3.65 | 0.000343 |
|  | Rn.7834.1 | 28.12 | 152.04 | 5.41 | 0.001241 |
|  | Rn.43678.1 | 322.42 | 157.06 | -2.05 | 0.000187 |
| Klhl14 | kelch-like 14 (Drosophila) | 70.13 | 25.38 | -2.76 | 0.001729 |
| Kif4 | kinesin family member 4 | 22.58 | 79.47 | 3.52 | 0.001101 |
|  | Rn.881.1 | 179.94 | 98.28 | -1.83 | 0.006124 |
|  | Rn.42056.1 | 428.78 | 202.36 | -2.12 | 0.003801 |
|  | Rn.19450.1 | 332.08 | 691.27 | 2.08 | 0.003981 |
| Apol3 | apolipoprotein L, 3 /// similar to apolipoprotein L, 3 | 89.43 | 281.03 | 3.14 | 0.038662 |
|  | Rn.46848.1 | 882.08 | 359.02 | -2.46 | 0.000031 |
| Sla | Src-like adaptor | 76.54 | 470.30 | 6.14 | 0.001732 |
| Kcnd2 | potassium voltage-gated channel, Shal-related subfamily, member 2 | 646.55 | 245.30 | -2.64 | 0.001056 |
| Asph | aspartate-beta-hydroxylase | 94.27 | 44.17 | -2.13 | 0.020177 |
| Oma1 | OMA1 homolog, zinc metallopeptidase (S. cerevisiae) | 102.43 | 238.97 | 2.33 | 0.000500 |
|  | Rn.49041.1 | 146.98 | 304.30 | 2.07 | 0.001245 |
|  | Rn.21416.1 | 735.92 | 1785.65 | 2.43 | 0.000318 |
|  | Rn.14588.1 | 183.31 | 86.59 | -2.12 | 0.000261 |
|  | Rn.18658.3 | 846.71 | 429.15 | -1.97 | 0.000122 |
|  | Rn.64254.1 | 112.16 | 53.09 | -2.11 | 0.001925 |
| Creg1 | cellular repressor of E1A-stimulated genes 1 | 817.33 | 1917.91 | 2.35 | 0.000227 |
| Brunol4 | bruno-like 4, RNA binding protein (Drosophila) | 1528.98 | 775.83 | -1.97 | 0.000218 |
|  | Rn.3224.1 | 616.16 | 1731.63 | 2.81 | 0.000036 |
| Gnb5 | guanine nucleotide binding protein (G protein), beta 5 | 1235.09 | 685.18 | -1.80 | 0.000018 |
| LOC498685 | similar to UPF0308 protein C9orf21 | 24.51 | 7.27 | -3.37 | 0.038346 |
| Tlr7 | toll-like receptor 7 | 54.06 | 424.90 | 7.86 | 0.001658 |
|  | Rn.16900.1 | 121.09 | 551.74 | 4.56 | 0.000006 |
| Fert2 | fer (fms/fps related) protein kinase, testis specific 2 | 52.75 | 100.14 | 1.90 | 0.002908 |
| Adfp | Adipose differentiation related protein | 397.96 | 1372.60 | 3.45 | 0.000306 |
| Lyve1 | lymphatic vessel endothelial hyaluronan receptor 1 | 162.20 | 390.54 | 2.41 | 0.020570 |
| Ryr2 | ryanodine receptor 2, cardiac | 193.54 | 43.17 | -4.48 | 0.003419 |
| Dusp6 | Dual specificity phosphatase 6 | 90.83 | 228.25 | 2.51 | 0.037121 |
|  | Rn.19936.1 | 74.04 | 38.16 | -1.94 | 0.003215 |
|  | Rn.64255.1 | 120.61 | 51.44 | -2.34 | 0.001408 |
|  | Rn.4117.1 | 1444.51 | 2640.25 | 1.83 | 0.002205 |
| RGD1310209 | similar to KIAA1324 protein | 50.23 | 26.53 | -1.89 | 0.015509 |
|  | Rn.3864.1 | 774.87 | 322.73 | -2.40 | 0.001432 |
| C1r | complement component 1, r subcomponent | 649.09 | 2340.87 | 3.61 | 0.000119 |
| Parp2 | poly (ADP-ribose) polymerase 2 | 167.04 | 289.28 | 1.73 | 0.000372 |
| Lck | lymphocyte-specific protein tyrosine kinase | 67.71 | 157.26 | 2.32 | 0.002446 |
|  | Rn.8380.1 | 1413.62 | 680.25 | -2.08 | 0.000338 |
| Pstpip1 | proline-serine-threonine phosphatase-interacting protein 1 | 49.29 | 216.34 | 4.39 | 0.000187 |
| LOC498685 | similar to UPF0308 protein C9orf21 | 101.37 | 50.43 | -2.01 | 0.000712 |
| Amigo1 | adhesion molecule with Ig like domain 1 | 519.20 | 278.79 | -1.86 | 0.000090 |
| Hk2 | Hexokinase 2 | 226.98 | 807.70 | 3.56 | 0.001319 |
| Irf7 | interferon regulatory factor 7 | 200.42 | 1049.49 | 5.24 | 0.000233 |
|  | Rn.32.1 | 535.27 | 271.63 | -1.97 | 0.000353 |
| Fn3k | fructosamine 3 kinase | 270.82 | 113.04 | -2.40 | 0.000518 |
| Asf1b | ASF1 anti-silencing function 1 homolog B (S. cerevisiae) | 30.62 | 81.85 | 2.67 | 0.005710 |
| Neurl3 | neuralized homolog 3 (Drosophila) | 17.37 | 160.92 | 9.26 | 0.001928 |
| LOC685045 | similar to Protein C6orf115 | 248.08 | 705.11 | 2.84 | 0.000023 |
| Bub1b | budding uninhibited by benzimidazoles 1 homolog, beta (S. cerevisiae) | 8.63 | 126.13 | 14.62 | 0.004846 |
| Vav2 | vav 2 guanine nucleotide exchange factor | 11.35 | 41.25 | 3.64 | 0.012185 |
|  | Rn.14817.1 | 63.57 | 503.61 | 7.92 | 0.017410 |
| Tesc | tescalcin | 295.36 | 96.33 | -3.07 | 0.000286 |
| Col11a1 | collagen, type XI, alpha 1 | 131.13 | 331.53 | 2.53 | 0.011075 |
|  | Rn.7284.1 | 272.73 | 108.94 | -2.50 | 0.000081 |
| Rdh10 | retinol dehydrogenase 10 (all-trans) | 112.22 | 336.04 | 2.99 | 0.010507 |
|  | Rn.40193.2 | 142.45 | 46.62 | -3.06 | 0.000733 |
| Mdm2 | Mdm2 p53 binding protein homolog (mouse) | 112.79 | 208.76 | 1.85 | 0.001039 |
|  | Rn.14792.1 | 144.50 | 277.95 | 1.92 | 0.000061 |
| LOC690418 | similar to Acidic proline-rich protein PRP25 precursor | 31.01 | 66.82 | 2.16 | 0.014384 |
| Cd3g | CD3 molecule, gamma polypeptide | 33.93 | 112.20 | 3.31 | 0.003471 |
|  | Rn.82.1 | 10.83 | 27.55 | 2.54 | 0.007868 |
| LOC685111 | similar to paired immunoglobin-like type 2 receptor beta /// similar to cell surface receptor FDFACT | 24.66 | 391.26 | 15.86 | 0.002416 |
|  | Rn.6545.3 | 942.24 | 2340.02 | 2.48 | 0.012746 |
|  | Rn.67973.1 | 66.92 | 21.60 | -3.10 | 0.007501 |
| Stra6 | stimulated by retinoic acid gene 6 | 109.05 | 925.77 | 8.49 | 0.009655 |
| Stra6 | stimulated by retinoic acid gene 6 | 22.98 | 171.23 | 7.45 | 0.029079 |
|  | Rn.895.1 | 689.45 | 347.77 | -1.98 | 0.000143 |
| Adam22 | a disintegrin and metalloprotease domain (ADAM) 22 | 17.13 | 42.98 | 2.51 | 0.001556 |
|  | Rn.34451.1 | 2343.35 | 1276.54 | -1.84 | 0.000511 |
| Il7r | Interleukin 7 receptor | 27.08 | 94.69 | 3.50 | 0.003498 |
|  | Rn.42138.1 | 229.43 | 111.06 | -2.07 | 0.002654 |
| Spta1 | spectrin, alpha, erythrocytic 1 (elliptocytosis 2) | 13.51 | 35.29 | 2.61 | 0.015116 |
|  | Rn.43507.1 | 842.92 | 443.51 | -1.90 | 0.000063 |
| Tomm34 | Translocase of outer mitochondrial membrane 34 | 204.28 | 114.49 | -1.78 | 0.000801 |
|  | Rn.14624.1 | 29.86 | 85.24 | 2.85 | 0.000312 |
|  | Rn.45420.2 | 150.57 | 74.11 | -2.03 | 0.000796 |
| Iars | isoleucyl-tRNA synthetase | 63.70 | 32.79 | -1.94 | 0.006973 |
| Spdya | speedy homolog A (Xenopus laevis) | 45.90 | 15.27 | -3.01 | 0.007790 |
| Dnajc6 | DnaJ (Hsp40) homolog, subfamily C, member 6 | 570.73 | 245.53 | -2.32 | 0.000421 |
| LOC100360491 | 60S ribosomal protein L13-like /// ribosomal protein L13 | 2564.84 | 4171.37 | 1.63 | 0.000023 |
| H2afz | H2A histone family, member Z | 2168.30 | 3568.08 | 1.65 | 0.002362 |
| Glul | glutamate-ammonia ligase (glutamine synthetase) | 1283.46 | 2343.73 | 1.83 | 0.000317 |
| Lgals3 | lectin, galactoside-binding, soluble, 3 | 1005.19 | 4950.49 | 4.92 | 0.000033 |
| Igfbp3 | insulin-like growth factor binding protein 3 | 1010.20 | 2741.61 | 2.71 | 0.004869 |
| Dynlt1 | dynein light chain Tctex-type 1 | 615.64 | 1191.81 | 1.94 | 0.000192 |
| Ech1 | enoyl coenzyme A hydratase 1, peroxisomal | 1028.90 | 1793.47 | 1.74 | 0.006929 |
| Eif4ebp1 | eukaryotic translation initiation factor 4E binding protein 1 | 121.89 | 276.70 | 2.27 | 0.003165 |
| Scd | stearoyl-CoA desaturase (delta-9-desaturase) | 305.24 | 111.31 | -2.74 | 0.004933 |
| Grn | granulin | 1008.56 | 4089.94 | 4.06 | 0.000117 |
| Ctsh | cathepsin H | 1171.85 | 3255.15 | 2.78 | 0.000066 |
| Serp1 | stress-associated endoplasmic reticulum protein 1 | 262.78 | 542.67 | 2.07 | 0.000197 |
| Cd36 | CD36 molecule (thrombospondin receptor) | 103.08 | 724.81 | 7.03 | 0.003678 |
| Glrx1 | glutaredoxin 1 | 887.58 | 1567.08 | 1.77 | 0.000615 |
| Pcolce | procollagen C-endopeptidase enhancer | 244.49 | 727.88 | 2.98 | 0.006357 |
| Pdpn | podoplanin | 1274.99 | 2769.96 | 2.17 | 0.000406 |
| Arpc1b | actin related protein 2/3 complex, subunit 1B | 608.47 | 2932.38 | 4.82 | 0.000044 |
| Anpep | alanyl (membrane) aminopeptidase | 81.35 | 556.94 | 6.85 | 0.000034 |
| Timp2 | TIMP metallopeptidase inhibitor 2 | 1206.98 | 3173.70 | 2.63 | 0.000182 |
| Prkab1 | protein kinase, AMP-activated, beta 1 non-catalytic subunit | 300.54 | 560.15 | 1.86 | 0.000009 |
| Nes | nestin | 63.58 | 162.98 | 2.56 | 0.008351 |
| Scarb1 | scavenger receptor class B, member 1 | 97.14 | 224.18 | 2.31 | 0.020844 |
| Plcb4 | phospholipase C, beta 4 | 1067.72 | 668.71 | -1.60 | 0.000037 |
| Lpl | lipoprotein lipase | 78.42 | 210.35 | 2.68 | 0.019949 |
| Nrn1 | neuritin 1 | 988.98 | 326.67 | -3.03 | 0.000033 |
| Il6ra | interleukin 6 receptor, alpha | 152.58 | 607.53 | 3.98 | 0.000116 |
| Pkn1 | protein kinase N1 | 162.66 | 355.06 | 2.18 | 0.000011 |
| Btg2 | BTG family, member 2 | 64.69 | 131.08 | 2.03 | 0.003830 |
| Luzp1 | leucine zipper protein 1 | 859.57 | 436.83 | -1.97 | 0.000106 |
| Nbl1 | neuroblastoma, suppression of tumorigenicity 1 | 204.47 | 387.25 | 1.89 | 0.000786 |
| Lcn2 | lipocalin 2 | 19.12 | 3394.49 | 177.51 | 0.005869 |
| Sqle | squalene epoxidase | 1555.63 | 640.33 | -2.43 | 0.000150 |
| Cyp51 | cytochrome P450, family 51 | 787.14 | 370.63 | -2.12 | 0.000025 |
| Aldh1a1 | aldehyde dehydrogenase 1 family, member A1 | 855.17 | 414.59 | -2.06 | 0.000013 |
| Dusp6 | dual specificity phosphatase 6 | 444.40 | 1221.67 | 2.75 | 0.019399 |
| Dync1i1 | dynein cytoplasmic 1 intermediate chain 1 | 1603.94 | 595.32 | -2.69 | 0.000012 |
| Cfh | complement factor H | 1414.57 | 4484.76 | 3.17 | 0.000004 |
| Erp29 | endoplasmic reticulum protein 29 | 1260.71 | 2145.09 | 1.70 | 0.000079 |
| Arhgap17 | Rho GTPase activating protein 17 | 357.30 | 799.06 | 2.24 | 0.000535 |
| Ddx39 | DEAD (Asp-Glu-Ala-Asp) box polypeptide 39 | 264.78 | 516.60 | 1.95 | 0.001544 |
| Kng1 | kininogen 1 /// kininogen 1-like 1 /// kininogen 2 | 8.73 | 156.12 | 17.89 | 0.003902 |
| Slc7a8 | solute carrier family 7 (cationic amino acid transporter, y+ system), member 8 | 242.49 | 507.46 | 2.09 | 0.000319 |
| Pctp | phosphatidylcholine transfer protein | 84.79 | 234.65 | 2.77 | 0.000007 |
| Chek1 | CHK1 checkpoint homolog (S. pombe) | 46.18 | 90.43 | 1.96 | 0.005311 |
| Rgs2 | regulator of G-protein signaling 2 | 575.96 | 946.64 | 1.64 | 0.000163 |
| Cebpb | CCAAT/enhancer binding protein (C/EBP), beta | 361.69 | 1713.35 | 4.74 | 0.000165 |
| Slco1a4 | solute carrier organic anion transporter family, member 1a4 | 183.11 | 322.42 | 1.76 | 0.004368 |
| Hps1 | Hermansky-Pudlak syndrome 1 homolog (human) | 22.13 | 77.26 | 3.49 | 0.013335 |
| Prkcd | protein kinase C, delta | 263.73 | 932.74 | 3.54 | 0.000010 |
| Adcy3 | adenylate cyclase 3 | 427.74 | 246.80 | -1.73 | 0.000357 |
| Serpini1 | serine (or cysteine) peptidase inhibitor, clade I, member 1 | 568.30 | 216.76 | -2.62 | 0.000087 |
| Calb2 | calbindin 2 | 815.99 | 308.04 | -2.65 | 0.000005 |
| Slfn3 | schlafen 3 | 89.33 | 991.11 | 11.10 | 0.012101 |
| Adam15 | a disintegrin and metallopeptidase domain 15 (metargidin) | 359.14 | 732.03 | 2.04 | 0.000980 |
| Dpysl5 | dihydropyrimidinase-like 5 | 668.22 | 355.73 | -1.88 | 0.003156 |
| Gprasp1 | G protein-coupled receptor associated sorting protein 1 | 2718.82 | 1584.56 | -1.72 | 0.000221 |
| Ril | reversion induced LIM gene | 113.27 | 232.03 | 2.05 | 0.000428 |
| Pcsk2 | proprotein convertase subtilisin/kexin type 2 | 139.21 | 49.18 | -2.83 | 0.000269 |
| Lect1 | leukocyte cell derived chemotaxin 1 | 56.06 | 17.15 | -3.27 | 0.018470 |
| Gria2 | glutamate receptor, ionotropic, AMPA 2 | 722.15 | 364.68 | -1.98 | 0.000347 |
| Adcy8 | adenylate cyclase 8 (brain) | 200.19 | 113.32 | -1.77 | 0.000764 |
| Il1r2 | interleukin 1 receptor, type II | 22.64 | 119.71 | 5.29 | 0.004606 |
| Gpr37 | G protein-coupled receptor 37 | 2704.28 | 1437.35 | -1.88 | 0.000425 |
| Nat1 | N-acetyltransferase 1 (arylamine N-acetyltransferase) | 65.19 | 145.30 | 2.23 | 0.003773 |
| St14 | suppression of tumorigenicity 14 (colon carcinoma) | 93.24 | 683.36 | 7.33 | 0.001224 |
| Inpp5d | inositol polyphosphate-5-phosphatase D | 65.08 | 254.16 | 3.91 | 0.003150 |
| Arhgef9 | Cdc42 guanine nucleotide exchange factor (GEF) 9 | 796.33 | 266.38 | -2.99 | 0.000533 |
| Icam1 | intercellular adhesion molecule 1 | 56.64 | 418.96 | 7.40 | 0.000958 |
| Negr1 | neuronal growth regulator 1 | 781.45 | 278.85 | -2.80 | 0.007570 |
| Gch1 | GTP cyclohydrolase 1 | 58.65 | 231.73 | 3.95 | 0.009330 |
| Dgkb | diacylglycerol kinase, beta | 353.40 | 176.56 | -2.00 | 0.000366 |
| Ina | internexin neuronal intermediate filament protein, alpha | 2852.13 | 1136.62 | -2.51 | 0.000087 |
| Hsd17b7 | hydroxysteroid (17-beta) dehydrogenase 7 | 329.32 | 91.41 | -3.60 | 0.000662 |
| Chga | chromogranin A | 1197.42 | 385.12 | -3.11 | 0.000017 |
| Gpr88 | G-protein coupled receptor 88 | 67.38 | 125.02 | 1.86 | 0.000602 |
| Eif2ak2 | eukaryotic translation initiation factor 2-alpha kinase 2 | 352.75 | 622.38 | 1.76 | 0.000182 |
| Sct | secretin | 23.58 | 56.56 | 2.40 | 0.006411 |
| Kcnk6 | potassium channel, subfamily K, member 6 | 54.13 | 191.31 | 3.53 | 0.002083 |
| Dgkg | diacylglycerol kinase, gamma | 137.83 | 55.60 | -2.48 | 0.000556 |
| Plaur | plasminogen activator, urokinase receptor | 5.75 | 51.65 | 8.98 | 0.001881 |
| Hhex | hematopoietically expressed homeobox | 229.87 | 498.03 | 2.17 | 0.000226 |
| Lyn | v-yes-1 Yamaguchi sarcoma viral related oncogene homolog | 50.47 | 119.30 | 2.36 | 0.000306 |
| Mx2 | myxovirus (influenza virus) resistance 2 | 112.65 | 311.36 | 2.76 | 0.004388 |
| Atp2b2 | ATPase, Ca++ transporting, plasma membrane 2 | 109.39 | 24.55 | -4.46 | 0.010895 |
| Grm1 | glutamate receptor, metabotropic 1 | 413.40 | 144.12 | -2.87 | 0.000028 |
| Grik1 | glutamate receptor, ionotropic, kainate 1 | 101.93 | 42.53 | -2.40 | 0.000956 |
| Myoc | myocilin | 746.01 | 204.02 | -3.66 | 0.006543 |
| Cxcl1 | chemokine (C-X-C motif) ligand 1 (melanoma growth stimulating activity, alpha) | 6.42 | 653.77 | 101.89 | 0.018744 |
| Khdrbs2 | KH domain containing, RNA binding, signal transduction associated 2 | 103.66 | 44.63 | -2.32 | 0.001033 |
| Gng5 | guanine nucleotide binding protein (G protein), gamma 5 | 1716.46 | 2824.70 | 1.65 | 0.000110 |
| Cebpd | CCAAT/enhancer binding protein (C/EBP), delta | 183.58 | 1658.37 | 9.03 | 0.000440 |
| Itgb1 | integrin, beta 1 | 238.42 | 662.22 | 2.78 | 0.005685 |
| Shox2 | short stature homeobox 2 | 43.27 | 14.67 | -2.95 | 0.001466 |
| Stat1 | signal transducer and activator of transcription 1 /// signal transducer and activator of transcription 4 | 65.87 | 304.09 | 4.62 | 0.024167 |
| Acan | aggrecan | 68.13 | 33.40 | -2.04 | 0.013648 |
| Nr1h3 | nuclear receptor subfamily 1, group H, member 3 | 51.50 | 227.70 | 4.42 | 0.000147 |
| Fcnb | ficolin B | 18.40 | 72.43 | 3.94 | 0.022522 |
| Slc32a1 | solute carrier family 32 (GABA vesicular transporter), member 1 | 958.51 | 388.27 | -2.47 | 0.000011 |
| Hnmt | histamine N-methyltransferase | 86.48 | 36.42 | -2.37 | 0.000307 |
| Il2rb | interleukin 2 receptor, beta | 15.89 | 76.59 | 4.82 | 0.024770 |
| Pclo | piccolo (presynaptic cytomatrix protein) | 46.33 | 16.06 | -2.88 | 0.017154 |
| Grin2a | glutamate receptor, ionotropic, N-methyl D-aspartate 2A | 261.38 | 68.66 | -3.81 | 0.000730 |
| Nap1l3 | nucleosome assembly protein 1-like 3 | 1089.39 | 515.89 | -2.11 | 0.000028 |
| Slc25a27 | solute carrier family 25, member 27 | 132.53 | 60.48 | -2.19 | 0.000291 |
| St8sia3 | ST8 alpha-N-acetyl-neuraminide alpha-2,8-sialyltransferase 3 | 86.10 | 37.33 | -2.31 | 0.006036 |
| Stau2 | staufen, RNA binding protein, homolog 2 (Drosophila) | 102.23 | 56.18 | -1.82 | 0.001118 |
| Glra1 | glycine receptor, alpha 1 | 179.53 | 28.87 | -6.22 | 0.007860 |
| Kcnd2 | potassium voltage-gated channel, Shal-related subfamily, member 2 | 671.98 | 219.09 | -3.07 | 0.002963 |
| Plcg2 | phospholipase C, gamma 2 | 17.13 | 58.69 | 3.43 | 0.000636 |
| Akap5 | A kinase (PRKA) anchor protein 5 | 53.35 | 21.80 | -2.45 | 0.021735 |
| Npy5r | neuropeptide Y receptor Y5 | 177.93 | 86.84 | -2.05 | 0.002967 |
| Stk17b | serine/threonine kinase 17b | 44.43 | 105.06 | 2.36 | 0.003882 |
| Slc5a7 | solute carrier family 5 (choline transporter), member 7 | 1049.91 | 495.53 | -2.12 | 0.001545 |
| Cntn6 | contactin 6 | 165.08 | 60.08 | -2.75 | 0.000888 |
| Pla2g4a | phospholipase A2, group IVA (cytosolic, calcium-dependent) | 384.89 | 1141.44 | 2.97 | 0.004319 |
| Lilrb3 | leukocyte immunoglobulin-like receptor, subfamily B (with TM and ITIM domains), member 3 | 8.11 | 189.29 | 23.34 | 0.010143 |
| Rassf9 | Ras association (RalGDS/AF-6) domain family (N-terminal) member 9 | 35.81 | 85.11 | 2.38 | 0.002331 |
| Ehd3 | EH-domain containing 3 | 35.22 | 84.74 | 2.41 | 0.022451 |
| Casp12 | caspase 12 | 50.71 | 140.05 | 2.76 | 0.011281 |
| Pdgfc | platelet derived growth factor C | 3.25 | 13.75 | 4.23 | 0.020623 |
| Cd86 | CD86 molecule | 23.28 | 84.26 | 3.62 | 0.000369 |
| LOC100365624 | myosin IC-like /// myosin IC | 21.71 | 58.09 | 2.68 | 0.018050 |
| Eef2k | eukaryotic elongation factor-2 kinase | 70.14 | 166.74 | 2.38 | 0.001016 |
| Syt4 | synaptotagmin IV | 65.28 | 32.15 | -2.03 | 0.005425 |
| Gpr85 | G protein-coupled receptor 85 | 365.39 | 174.15 | -2.10 | 0.002140 |
| Plau | plasminogen activator, urokinase | 31.66 | 499.03 | 15.76 | 0.002905 |
| Igsf6 | immunoglobulin superfamily, member 6 | 24.64 | 634.99 | 25.78 | 0.001191 |
| Casp3 | caspase 3 | 34.12 | 81.95 | 2.40 | 0.005205 |
| Glra2 | glycine receptor, alpha 2 | 141.68 | 51.46 | -2.75 | 0.001068 |
| Omp | olfactory marker protein | 15.20 | 55.36 | 3.64 | 0.032127 |
| Cd8b | CD8b molecule | 7.52 | 158.21 | 21.04 | 0.000303 |
| Lmbrd1 | LMBR1 domain containing 1 | 1038.51 | 554.40 | -1.87 | 0.000971 |
| Ugt1a1 | UDP glucuronosyltransferase 1 family, polypeptide A1 /// UDP glucuronosyltransferase 1 family, polypeptide A2 /// UDP glycosyltransferase 1 family, polypeptide A3 /// UDP glucuronosyltransferase 1 family, polypeptide A5 /// UDP glucuronosyltransferase 1 family, polypeptide A6 /// UDP glucuronosyltransferase 1 family, polypeptide A7C /// UDP glycosyltransferase 1 family, polypeptide A8 /// UDP glucuronosyltransferase 1 family, polypeptide A9 | 61.57 | 262.61 | 4.27 | 0.001679 |
| Id3 | inhibitor of DNA binding 3 | 403.28 | 1142.90 | 2.83 | 0.000293 |
| Tgm2 | transglutaminase 2, C polypeptide | 16.37 | 74.64 | 4.56 | 0.016799 |
| Junb | jun B proto-oncogene | 14.94 | 52.83 | 3.54 | 0.009389 |
| Fcn1 | ficolin (collagen/fibrinogen domain containing) 1 | 137.34 | 331.31 | 2.41 | 0.000456 |
| Cr1l | complement component (3b/4b) receptor 1-like | 525.97 | 1216.81 | 2.31 | 0.000093 |
| Fxyd2 | FXYD domain-containing ion transport regulator 2 | 36.13 | 209.33 | 5.79 | 0.000120 |
| Ppp2r2b | protein phosphatase 2 (formerly 2A), regulatory subunit B, beta isoform | 1892.99 | 1032.71 | -1.83 | 0.000065 |
| Trim63 | tripartite motif-containing 63 | 8.20 | 26.57 | 3.24 | 0.011788 |
| Rap1b | RAP1B, member of RAS oncogene family | 1839.26 | 3557.62 | 1.93 | 0.000687 |
| Slc7a7 | solute carrier family 7 (cationic amino acid transporter, y+ system), member 7 | 64.21 | 569.70 | 8.87 | 0.000121 |
| Casp4 | caspase 4, apoptosis-related cysteine peptidase | 111.89 | 372.78 | 3.33 | 0.000326 |
| Ykt6 | YKT6 v-SNARE homolog (S. cerevisiae) | 222.28 | 108.77 | -2.04 | 0.004139 |
| Hmgcr | 3-hydroxy-3-methylglutaryl-Coenzyme A reductase | 171.78 | 57.32 | -3.00 | 0.002414 |
| Tmeff1 | transmembrane protein with EGF-like and two follistatin-like domains 1 | 464.01 | 206.51 | -2.25 | 0.000902 |
| Col1a2 | collagen, type I, alpha 2 | 837.11 | 1820.22 | 2.17 | 0.045572 |
| Myo9b | myosin IXb | 211.76 | 406.39 | 1.92 | 0.001413 |
| Lbp | lipopolysaccharide binding protein | 22.14 | 636.47 | 28.75 | 0.001551 |
| Fcgrt | Fc fragment of IgG, receptor, transporter, alpha | 198.88 | 500.16 | 2.51 | 0.009286 |
| Prelp | proline/arginine-rich end leucine-rich repeat protein | 55.11 | 187.49 | 3.40 | 0.000002 |
| Prdx4 | peroxiredoxin 4 | 758.40 | 1248.17 | 1.65 | 0.000046 |
| C1s | complement component 1, s subcomponent | 849.05 | 2846.30 | 3.35 | 0.000371 |
| Cdc20 | cell division cycle 20 homolog (S. cerevisiae) | 60.04 | 176.64 | 2.94 | 0.000893 |
| Epb4.1l1 | erythrocyte protein band 4.1-like 1 | 1142.81 | 644.80 | -1.77 | 0.000101 |
| Cyp4f6 | cytochrome P450, family 4, subfamily f, polypeptide 6 | 67.01 | 149.61 | 2.23 | 0.006998 |
| Rnf112 | ring finger protein 112 | 373.63 | 199.30 | -1.87 | 0.000366 |
| Ngef | neuronal guanine nucleotide exchange factor | 542.66 | 245.92 | -2.21 | 0.001330 |
| Sc5dl | sterol-C5-desaturase (ERG3 delta-5-desaturase homolog, S. cerevisiae)-like | 1457.54 | 495.40 | -2.94 | 0.000065 |
| Pmf31 | PMF32 protein | 184.54 | 77.48 | -2.38 | 0.002230 |
| Lgals3bp | lectin, galactoside-binding, soluble, 3 binding protein | 144.03 | 958.68 | 6.66 | 0.001503 |
| Mafb | v-maf musculoaponeurotic fibrosarcoma oncogene homolog B (avian) | 69.94 | 729.21 | 10.43 | 0.015517 |
| Cd44 | Cd44 molecule | 131.89 | 462.33 | 3.51 | 0.001494 |
| Cklf | chemokine-like factor | 99.48 | 298.50 | 3.00 | 0.010956 |
| Aspg | asparaginase homolog (S. cerevisiae) | 81.20 | 241.15 | 2.97 | 0.008209 |
| Asrgl1 | asparaginase like 1 | 446.07 | 257.16 | -1.73 | 0.001596 |
| Slc6a15 | solute carrier family 6 (neutral amino acid transporter), member 15 | 561.61 | 276.05 | -2.03 | 0.000478 |
| Cxcl10 | chemokine (C-X-C motif) ligand 10 | 49.07 | 279.37 | 5.69 | 0.027229 |
| Slc38a5 | solute carrier family 38, member 5 | 56.29 | 150.20 | 2.67 | 0.002497 |
| Tlr4 | toll-like receptor 4 | 58.41 | 256.13 | 4.39 | 0.001768 |
| Cklf | chemokine-like factor | 73.96 | 285.68 | 3.86 | 0.015389 |
| Lilrb3l | leukocyte immunoglobulin-like receptor, subfamily B (with TM and ITIM domains), member 3-like | 29.36 | 102.06 | 3.48 | 0.006114 |
| Ifitm3 | interferon induced transmembrane protein 3 | 600.56 | 2710.55 | 4.51 | 0.000855 |
| Slc24a2 | solute carrier family 24 (sodium/potassium/calcium exchanger), member 2 | 1258.34 | 494.08 | -2.55 | 0.000925 |
| LOC100310874 | antisense RNA overlapping MCH | 14.31 | 75.45 | 5.27 | 0.002693 |
| Kif1b | kinesin family member 1B /// phosphogluconate dehydrogenase | 500.08 | 242.07 | -2.07 | 0.010653 |
| Atp2b3 | ATPase, Ca++ transporting, plasma membrane 3 | 1331.85 | 446.02 | -2.99 | 0.000716 |
| Itgam | integrin, alpha M | 73.62 | 366.81 | 4.98 | 0.016479 |
| Syt12 | synaptotagmin XII | 181.02 | 37.64 | -4.81 | 0.002296 |
| RT1-EC2 | RT1 class Ib, locus EC2 | 430.37 | 2070.60 | 4.81 | 0.004160 |
| Tmem37 | transmembrane protein 37 | 46.58 | 215.53 | 4.63 | 0.000398 |
| Elovl6 | ELOVL family member 6, elongation of long chain fatty acids (yeast) | 986.38 | 419.77 | -2.35 | 0.002881 |
| RGD1309537 | similar to Myosin regulatory light chain 2-A, smooth muscle isoform (Myosin RLC-A) | 1364.95 | 3050.26 | 2.23 | 0.000704 |
| Col1a1 | collagen, type I, alpha 1 | 80.27 | 279.93 | 3.49 | 0.026246 |
| Pdcd6ip | programmed cell death 6 interacting protein | 242.41 | 420.10 | 1.73 | 0.002568 |
| Cyp4v3 | cytochrome P450, family 4, subfamily v, polypeptide 3 | 374.78 | 781.41 | 2.08 | 0.002086 |
| Thbs4 | thrombospondin 4 | 74.17 | 278.46 | 3.75 | 0.044032 |
| Rab13 | RAB13, member RAS oncogene family | 228.32 | 620.96 | 2.72 | 0.000052 |
| Vcan | versican | 590.36 | 1122.53 | 1.90 | 0.003289 |
| Col18a1 | collagen, type XVIII, alpha 1 | 49.91 | 315.37 | 6.32 | 0.003553 |
| Tap1 | transporter 1, ATP-binding cassette, sub-family B (MDR/TAP) | 39.39 | 246.23 | 6.25 | 0.000142 |
| Plcb3 | phospholipase C, beta 3 (phosphatidylinositol-specific) | 202.76 | 387.53 | 1.91 | 0.000150 |
| RT1-S3 | RT1 class Ib, locus S3 | 107.61 | 421.90 | 3.92 | 0.002166 |
| Fgfr2 | fibroblast growth factor receptor 2 | 14.98 | 31.12 | 2.08 | 0.005018 |
| Grm3 | glutamate receptor, metabotropic 3 | 524.33 | 220.55 | -2.38 | 0.000816 |
| LOC100364500 | RT1 class I, locus CE11-like /// RT1 class I, locus A3 /// RT1 class I, locus CE10 /// RT1 class I, locus CE2 /// RT1 class Ib, locus EC2 | 321.15 | 1741.05 | 5.42 | 0.006253 |
| RT1-S3 | RT1 class Ib, locus S3 | 201.59 | 811.00 | 4.02 | 0.000175 |
| RT1-S3 | RT1 class Ib, locus S3 | 77.88 | 315.61 | 4.05 | 0.003206 |
| Kcnc2 | potassium voltage gated channel, Shaw-related subfamily, member 2 | 26.38 | 10.81 | -2.44 | 0.017446 |
| RT1-CE12 | RT1 class I, locus CE12 | 242.91 | 1945.75 | 8.01 | 0.022370 |
| Rpsa | ribosomal protein SA | 1757.32 | 3440.18 | 1.96 | 0.000004 |
| RT1-CE5 | RT1 class I, locus CE5 | 50.68 | 124.50 | 2.46 | 0.020223 |
| Mt1a | metallothionein 1a | 20.46 | 90.35 | 4.42 | 0.017051 |
| Mt2A | metallothionein 2A | 1051.47 | 5597.24 | 5.32 | 0.000001 |
| Tcrb | T-cell receptor beta chain | 12.79 | 86.43 | 6.76 | 0.005428 |
| Klra5 | killer cell lectin-like receptor, subfamily A, member 5 /// Ly49 stimulatory receptor 7 | 8.31 | 318.18 | 38.31 | 0.000047 |
| Rps25 | ribosomal protein s25 | 1846.30 | 3338.45 | 1.81 | 0.011169 |
| Spint2 | serine peptidase inhibitor, Kunitz type, 2 | 107.30 | 235.10 | 2.19 | 0.006931 |
| Tagln2 | transgelin 2 | 316.78 | 1207.16 | 3.81 | 0.003063 |
| Np | nucleoside phosphorylase | 1542.51 | 3414.66 | 2.21 | 0.000057 |
| Ns5atp9 | NS5A (hepatitis C virus) transactivated protein 9 | 51.43 | 739.91 | 14.39 | 0.004346 |
| Ly6e | lymphocyte antigen 6 complex, locus E | 139.08 | 269.46 | 1.94 | 0.000382 |
| RGD1566401 | similar to GTL2, imprinted maternally expressed untranslated | 1889.41 | 789.75 | -2.39 | 0.000258 |
| Tax1bp3 | Tax1 (human T-cell leukemia virus type I) binding protein 3 | 319.35 | 1039.74 | 3.26 | 0.000124 |
| Plp2 | proteolipid protein 2 (colonic epithelium-enriched) | 154.42 | 695.19 | 4.50 | 0.001055 |
| Prr13 | proline rich 13 | 813.96 | 1642.75 | 2.02 | 0.000092 |
| Rrbp1 | ribosome binding protein 1 | 595.29 | 1158.66 | 1.95 | 0.000012 |
| Oaf | OAF homolog (Drosophila) | 277.41 | 629.69 | 2.27 | 0.000282 |
| Mxra8 | matrix-remodelling associated 8 | 135.45 | 343.96 | 2.54 | 0.000833 |
| Fkbp10 | FK506 binding protein 10 | 186.38 | 472.72 | 2.54 | 0.008917 |
| Anxa11 | annexin A11 | 909.23 | 1526.67 | 1.68 | 0.000025 |
| Myadm | myeloid-associated differentiation marker | 222.09 | 405.18 | 1.82 | 0.004895 |
| Col18a1 | collagen, type XVIII, alpha 1 | 59.03 | 198.72 | 3.37 | 0.007069 |
| Capg | capping protein (actin filament), gelsolin-like | 178.28 | 882.73 | 4.95 | 0.000073 |
| Fbxo9 | f-box protein 9 | 1415.14 | 685.00 | -2.07 | 0.000063 |
| Fam129b | family with sequence similarity 129, member B | 714.15 | 1345.27 | 1.88 | 0.002204 |
| Ube2c | ubiquitin-conjugating enzyme E2C | 24.41 | 536.42 | 21.97 | 0.002465 |
| Cxcl14 | chemokine (C-X-C motif) ligand 14 | 644.26 | 2599.60 | 4.04 | 0.000385 |
| Tnip1 | TNFAIP3 interacting protein 1 | 280.97 | 595.57 | 2.12 | 0.000361 |
| Flnc | filamin C, gamma | 221.09 | 458.05 | 2.07 | 0.003815 |
| Arap1 | ArfGAP with RhoGAP domain, ankyrin repeat and PH domain 1 | 324.06 | 646.97 | 2.00 | 0.000009 |
| Sec61b | Sec61 beta subunit | 1113.97 | 2173.36 | 1.95 | 0.000192 |
| Pptc7 | PTC7 protein phosphatase homolog (S. cerevisiae) | 1211.55 | 720.02 | -1.68 | 0.000279 |
| Cdc42se1 | CDC42 small effector 1 | 128.82 | 231.95 | 1.80 | 0.003930 |
|  | Rn.1322.1 | 323.14 | 633.03 | 1.96 | 0.009569 |
| Tubb2c | tubulin, beta 2c | 148.75 | 509.79 | 3.43 | 0.015414 |
|  | Rn.8802.1 | 856.25 | 1497.85 | 1.75 | 0.000584 |
| Lasp1 | LIM and SH3 protein 1 | 1394.00 | 2401.28 | 1.72 | 0.000709 |
| Ier3 | immediate early response 3 | 310.21 | 1095.16 | 3.53 | 0.004426 |
| Cotl1 | coactosin-like 1 (Dictyostelium) | 1036.75 | 2439.01 | 2.35 | 0.000109 |
| LOC691995 | hypothetical protein LOC691995 | 2023.14 | 1120.14 | -1.81 | 0.000318 |
| Cfd | complement factor D (adipsin) | 40.25 | 674.00 | 16.74 | 0.003805 |
| Tmed3 | transmembrane emp24 protein transport domain containing 3 | 265.67 | 526.11 | 1.98 | 0.001164 |
| Rab6b | RAB6B, member RAS oncogene family | 1820.54 | 976.65 | -1.86 | 0.000042 |
| Top2a | topoisomerase (DNA) II alpha | 42.41 | 826.05 | 19.48 | 0.001635 |
| Dchs1 | dachsous 1 (Drosophila) | 135.86 | 255.98 | 1.88 | 0.002005 |
| Carhsp1 | calcium regulated heat stable protein 1 | 525.09 | 992.58 | 1.89 | 0.000380 |
| Man2a1 | mannosidase, alpha, class 2A, member 1 | 301.24 | 656.00 | 2.18 | 0.005041 |
| Lsp1 | lymphocyte-specific protein 1 | 47.70 | 152.52 | 3.20 | 0.003923 |
| Cdkn1a | cyclin-dependent kinase inhibitor 1A | 275.41 | 517.58 | 1.88 | 0.000340 |
| RT1-T24-3 | RT1 class I, locus T24, gene 3 | 200.99 | 657.91 | 3.27 | 0.008079 |
| Man2b1 | mannosidase, alpha, class 2B, member 1 | 195.99 | 739.89 | 3.78 | 0.001326 |
| Rreb1 | ras responsive element binding protein 1 | 184.51 | 659.65 | 3.58 | 0.000144 |
| Il13ra1 | interleukin 13 receptor, alpha 1 | 203.10 | 939.63 | 4.63 | 0.001698 |
|  | Rn.1130.1 | 419.01 | 727.96 | 1.74 | 0.000393 |
| LOC100188932 | dolichyl-diphosphooligosaccharide--protein glycosyltransferase subunit 4 | 987.29 | 1914.01 | 1.94 | 0.014597 |
| Rras | Harvey rat sarcoma virus oncogene, subgroup R | 99.79 | 221.48 | 2.22 | 0.003476 |
| Fermt3 | fermitin family homolog 3 (Drosophila) | 132.31 | 992.35 | 7.50 | 0.000056 |
|  | Rn.7933.1 | 95.24 | 322.32 | 3.38 | 0.000853 |
|  | Rn.43522.1 | 751.45 | 427.55 | -1.76 | 0.000420 |
| Serpine3 | serpin peptidase inhibitor, clade E (nexin, plasminogen activator inhibitor type 1), member 3 | 33.60 | 5.74 | -5.85 | 0.000694 |
| Hdac1 | histone deacetylase 1 | 276.61 | 506.98 | 1.83 | 0.006085 |
| Iqgap1 | IQ motif containing GTPase activating protein 1 | 50.87 | 148.61 | 2.92 | 0.003551 |
|  | Rn.43993.1 | 212.23 | 821.98 | 3.87 | 0.000377 |
| Iqgap1 | IQ motif containing GTPase activating protein 1 | 233.34 | 701.72 | 3.01 | 0.002367 |
| Akt2 | V-akt murine thymoma viral oncogene homolog 2 | 101.50 | 233.95 | 2.30 | 0.008194 |
| Shisa5 | shisa homolog 5 (Xenopus laevis) | 728.99 | 1488.22 | 2.04 | 0.000521 |
| Csf1r | colony stimulating factor 1 receptor | 1448.73 | 3752.32 | 2.59 | 0.000008 |
|  | Rn.7071.1 | 135.28 | 313.08 | 2.31 | 0.022198 |
| Gadd45g | growth arrest and DNA-damage-inducible, gamma | 113.88 | 290.64 | 2.55 | 0.001499 |
| Bex1 | brain expressed gene 1 | 769.02 | 396.68 | -1.94 | 0.000115 |
| Smpdl3a | sphingomyelin phosphodiesterase, acid-like 3A | 838.22 | 3248.42 | 3.88 | 0.000390 |
|  | Rn.7225.1 | 1549.89 | 719.34 | -2.15 | 0.000015 |
| Ddx19a | DEAD (Asp-Glu-Ala-Asp) box polypeptide 19a | 389.15 | 689.83 | 1.77 | 0.000514 |
| Scamp1 | secretory carrier membrane protein 1 | 1418.40 | 798.11 | -1.78 | 0.000026 |
| LOC685909 | similar to H2A histone family, member V isoform 1 | 797.04 | 1522.76 | 1.91 | 0.000233 |
| Prkch | protein kinase C, eta | 58.52 | 215.11 | 3.68 | 0.000109 |
| Slc44a2 | solute carrier family 44, member 2 | 349.73 | 859.32 | 2.46 | 0.003335 |
| Rgl2 | ral guanine nucleotide dissociation stimulator-like 2 | 231.25 | 504.01 | 2.18 | 0.007115 |
|  | Rn.18855.1 | 783.44 | 265.93 | -2.95 | 0.000023 |
| Bcl2l12 | BCL2-like 12 (proline rich) | 51.99 | 168.62 | 3.24 | 0.000952 |
|  | Rn.10780.2 | 1075.61 | 329.44 | -3.26 | 0.000027 |
|  | Rn.17882.1 | 435.51 | 890.59 | 2.04 | 0.018872 |
| Pgp | phosphoglycolate phosphatase | 635.84 | 387.78 | -1.64 | 0.000045 |
| Pold4 | polymerase (DNA-directed), delta 4 | 193.05 | 457.37 | 2.37 | 0.000587 |
| RGD1310224 | similar to RIKEN cDNA 1810022C23 | 199.99 | 480.48 | 2.40 | 0.000253 |
| Ggta1 | glycoprotein, alpha-galactosyltransferase 1 | 155.66 | 789.11 | 5.07 | 0.000122 |
|  | Rn.22112.1 | 761.05 | 421.26 | -1.81 | 0.000030 |
| LOC290577 | hypothetical LOC290577 | 161.63 | 347.28 | 2.15 | 0.001439 |
| Loxl1 | lysyl oxidase-like 1 | 233.85 | 532.67 | 2.28 | 0.001006 |
| Angptl4 | angiopoietin-like 4 | 154.52 | 898.48 | 5.81 | 0.000952 |
| Tmem123 | transmembrane protein 123 | 683.22 | 1519.78 | 2.22 | 0.003110 |
| Lama5 | laminin, alpha 5 | 95.28 | 245.24 | 2.57 | 0.028950 |
| Col15a1 | collagen, type XV, alpha 1 | 56.75 | 238.29 | 4.20 | 0.003684 |
| Arhgap25 | Rho GTPase activating protein 25 | 92.92 | 326.59 | 3.51 | 0.000041 |
| RGD1311307 | similar to 1300014I06Rik protein | 263.09 | 517.54 | 1.97 | 0.001188 |
|  | Rn.34678.1 | 45.80 | 91.88 | 2.01 | 0.007823 |
| Ehd2 | EH-domain containing 2 | 155.32 | 397.92 | 2.56 | 0.000171 |
|  | Rn.7825.1 | 34.72 | 103.66 | 2.99 | 0.002428 |
|  | Rn.7947.1 | 521.68 | 1188.78 | 2.28 | 0.002794 |
| Fzd6 | frizzled homolog 6 (Drosophila) | 37.08 | 94.22 | 2.54 | 0.004423 |
| Epb4.9 | erythrocyte protein band 4.9 | 406.98 | 201.71 | -2.02 | 0.000071 |
| Tln1 | talin 1 | 395.74 | 876.67 | 2.22 | 0.000489 |
|  | Rn.4301.1 | 928.33 | 4892.28 | 5.27 | 0.000002 |
|  | Rn.7582.1 | 650.25 | 357.72 | -1.82 | 0.000175 |
| Nampt | nicotinamide phosphoribosyltransferase | 448.31 | 854.75 | 1.91 | 0.016547 |
| LOC305633 | similar to Antxr2 protein | 192.72 | 582.02 | 3.02 | 0.000341 |
| LOC686539 | similar to immunoglobulin superfamily containing leucine-rich repeat | 482.96 | 1317.37 | 2.73 | 0.006668 |
|  | Rn.7736.1 | 185.94 | 422.86 | 2.27 | 0.003860 |
| Bmp1 | bone morphogenetic protein 1 | 223.45 | 452.49 | 2.02 | 0.001844 |
|  | Rn.23344.1 | 730.15 | 385.96 | -1.89 | 0.000338 |
| LOC498368 | similar to RIKEN cDNA 0610040J01 | 200.84 | 379.38 | 1.89 | 0.000859 |
| Ehbp1l1 | EH domain binding protein 1-like 1 | 141.26 | 475.27 | 3.36 | 0.000135 |
| Reep4 | receptor accessory protein 4 | 124.83 | 247.46 | 1.98 | 0.005398 |
| Fbxl6 | F-box and leucine-rich repeat protein 6 | 113.58 | 223.97 | 1.97 | 0.000729 |
|  | Rn.19527.1 | 408.91 | 188.95 | -2.16 | 0.000431 |
| Usp3 | ubiquitin specific peptidase 3 | 143.44 | 278.19 | 1.94 | 0.002723 |
| Il2rg | interleukin 2 receptor, gamma | 11.05 | 181.77 | 16.46 | 0.001355 |
|  | Rn.9149.3 | 1486.77 | 888.51 | -1.67 | 0.000021 |
| Ccnc | cyclin C | 556.87 | 306.54 | -1.82 | 0.000436 |
|  | Rn.17462.1 | 3151.48 | 1708.03 | -1.85 | 0.000034 |
| Kcnc3 | potassium voltage gated channel, Shaw-related subfamily, member 3 | 70.97 | 25.82 | -2.75 | 0.010722 |
| Ccl6 | chemokine (C-C motif) ligand 6 | 178.26 | 1901.84 | 10.67 | 0.000429 |
|  | Rn.4238.1 | 177.87 | 427.05 | 2.40 | 0.000232 |
|  | Rn.6421.1 | 224.26 | 530.74 | 2.37 | 0.000850 |
| Dos | downstream of Stk11 | 976.49 | 442.75 | -2.21 | 0.000159 |
| Pgrmc2 | progesterone receptor membrane component 2 | 781.25 | 438.47 | -1.78 | 0.001213 |
| Casp7 | caspase 7 | 176.09 | 320.35 | 1.82 | 0.001908 |
| Tmem38a | transmembrane protein 38a | 439.85 | 199.34 | -2.21 | 0.000142 |
| Actn1 | actinin, alpha 1 | 341.35 | 601.97 | 1.76 | 0.003879 |
| Vps36 | vacuolar protein sorting 36 homolog (S. cerevisiae) | 238.96 | 467.46 | 1.96 | 0.003624 |
| Lcp1 | lymphocyte cytosolic protein 1 | 521.89 | 2087.88 | 4.00 | 0.000091 |
|  | Rn.38182.1 | 129.61 | 311.71 | 2.41 | 0.012026 |
|  | Rn.46501.1 | 182.12 | 95.26 | -1.91 | 0.000741 |
| Vwf | von Willebrand factor | 98.49 | 287.97 | 2.92 | 0.001333 |
| Icam2 | intercellular adhesion molecule 2 | 132.37 | 271.76 | 2.05 | 0.001096 |
| Cxcr4 | chemokine (C-X-C motif) receptor 4 | 109.93 | 468.75 | 4.26 | 0.000092 |
|  | Rn.8685.1 | 132.59 | 534.31 | 4.03 | 0.000060 |
| Vnn1 | vanin 1 | 28.17 | 125.50 | 4.45 | 0.000182 |
|  | Rn.22489.1 | 207.19 | 390.28 | 1.88 | 0.001012 |
| Sh3bp1 | SH3-domain binding protein 1 | 12.98 | 67.28 | 5.18 | 0.000191 |
|  | Rn.40382.1 | 210.82 | 103.25 | -2.04 | 0.000184 |
|  | Rn.13986.1 | 560.29 | 947.00 | 1.69 | 0.001715 |
| Cyfip1 | cytoplasmic FMR1 interacting protein 1 | 1288.60 | 2392.99 | 1.86 | 0.000225 |
| Ino80c | INO80 complex subunit C | 133.80 | 229.14 | 1.71 | 0.000122 |
| Mif4gd | MIF4G domain containing | 196.90 | 365.69 | 1.86 | 0.000154 |
| Anxa4 | annexin A4 | 623.98 | 1158.19 | 1.86 | 0.006393 |
| Matn2 | matrilin 2 | 228.95 | 612.40 | 2.67 | 0.000356 |
|  | Rn.22244.1 | 526.73 | 294.41 | -1.79 | 0.000161 |
| Tie1 | tyrosine kinase with immunoglobulin-like and EGF-like domains 1 | 84.25 | 284.54 | 3.38 | 0.014434 |
| Dcaf5 | DDB1 and CUL4 associated factor 5 | 279.17 | 146.93 | -1.90 | 0.000364 |
| Plxnd1 | plexin D1 | 53.45 | 164.06 | 3.07 | 0.011192 |
| Tal1 | T-cell acute lymphocytic leukemia 1 | 82.06 | 245.55 | 2.99 | 0.000154 |
| Lrrfip1 | leucine rich repeat (in FLII) interacting protein 1 | 76.19 | 488.28 | 6.41 | 0.000842 |
| Ier5 | immediate early response 5 | 357.39 | 772.31 | 2.16 | 0.000560 |
| Ptpn3 | protein tyrosine phosphatase, non-receptor type 3 | 150.01 | 69.72 | -2.15 | 0.001470 |
| LOC690000 | similar to CG3740-PA | 158.35 | 331.49 | 2.09 | 0.003346 |
|  | Rn.30301.1 | 145.80 | 344.03 | 2.36 | 0.005284 |
|  | Rn.16133.1 | 76.48 | 200.59 | 2.62 | 0.000393 |
|  | Rn.23552.1 | 238.21 | 520.35 | 2.18 | 0.048380 |
|  | Rn.15108.1 | 307.10 | 657.97 | 2.14 | 0.010463 |
| Csrnp1 | cysteine-serine-rich nuclear protein 1 | 54.03 | 113.20 | 2.10 | 0.000477 |
| Bmp7 | bone morphogenetic protein 7 | 75.31 | 375.85 | 4.99 | 0.004166 |
| Rrm2 | ribonucleotide reductase M2 | 55.33 | 1249.33 | 22.58 | 0.001574 |
| Tes | testis derived transcript | 140.32 | 357.96 | 2.55 | 0.025299 |
|  | Rn.11601.1 | 536.56 | 241.69 | -2.22 | 0.000087 |
| RGD1560398 | RGD1560398 | 1566.16 | 911.11 | -1.72 | 0.000597 |
|  | Rn.23543.1 | 65.01 | 160.38 | 2.47 | 0.011608 |
|  | Rn.40977.1 | 28.11 | 71.64 | 2.55 | 0.016665 |
| LOC100360582 | 5',3'-nucleotidase, cytosolic /// similar to 5(3)-deoxyribonucleotidase, cytosolic type (Cytosolic 5,3-pyrimidine nucleotidase) (Deoxy-5-nucleotidase 1) (dNT-1) | 38.78 | 91.67 | 2.36 | 0.003355 |
| Ccdc92 | coiled-coil domain containing 92 | 666.50 | 324.37 | -2.05 | 0.000041 |
|  | Rn.15508.1 | 477.57 | 198.43 | -2.41 | 0.000009 |
|  | Rn.7039.1 | 640.95 | 200.05 | -3.20 | 0.000217 |
| Lnx1 | ligand of numb-protein X 1 | 249.88 | 121.90 | -2.05 | 0.000243 |
| Tmem100 | transmembrane protein 100 | 346.03 | 1037.46 | 3.00 | 0.022156 |
| Rpia | ribose 5-phosphate isomerase A | 270.92 | 507.21 | 1.87 | 0.003417 |
| Cfb | complement factor B | 88.39 | 846.88 | 9.58 | 0.001416 |
| Mylip | myosin regulatory light chain interacting protein | 198.37 | 462.21 | 2.33 | 0.004315 |
|  | Rn.35620.1 | 36.41 | 161.58 | 4.44 | 0.000197 |
| Klf3 | Kruppel-like factor 3 (basic) | 489.59 | 855.71 | 1.75 | 0.000097 |
| Krcc1 | lysine-rich coiled-coil 1 | 610.46 | 1090.39 | 1.79 | 0.001178 |
|  | Rn.22499.1 | 115.56 | 359.51 | 3.11 | 0.003495 |
| Dnajb12 | DnaJ (Hsp40) homolog, subfamily B, member 12 | 57.05 | 274.28 | 4.81 | 0.000511 |
| Cdc20 | Cell division cycle 20 homolog (S. cerevisiae) | 18.95 | 100.73 | 5.31 | 0.000164 |
|  | Rn.15631.1 | 1765.74 | 870.31 | -2.03 | 0.000013 |
|  | Rn.51497.1 | 101.06 | 214.29 | 2.12 | 0.000072 |
| Rnf149 | ring finger protein 149 | 715.45 | 1295.49 | 1.81 | 0.000815 |
| Fbln2 | fibulin 2 | 241.12 | 791.30 | 3.28 | 0.011491 |
| Nfkbia | nuclear factor of kappa light polypeptide gene enhancer in B-cells inhibitor, alpha | 316.24 | 895.28 | 2.83 | 0.020814 |
| Cenpe | centromere protein E | 9.77 | 52.21 | 5.35 | 0.006419 |
| Masp1 | mannan-binding lectin serine peptidase 1 | 173.83 | 355.95 | 2.05 | 0.001876 |
|  | Rn.9513.1 | 1718.89 | 796.72 | -2.16 | 0.000007 |
| Clec4a3 | C-type lectin domain family 4, member a3 | 62.01 | 1441.54 | 23.25 | 0.000736 |
| Tcf19 | transcription factor 19 | 58.73 | 278.71 | 4.75 | 0.001845 |
| Kifap3 | kinesin-associated protein 3 | 1958.45 | 1155.46 | -1.69 | 0.000113 |
|  | Rn.22478.1 | 63.08 | 131.97 | 2.09 | 0.000231 |
| Ccnb2 | cyclin B2 | 53.42 | 582.23 | 10.90 | 0.002157 |
| Calhm2 | calcium homeostasis modulator 2 | 80.45 | 232.59 | 2.89 | 0.000672 |
| Stat2 | signal transducer and activator of transcription 2 | 267.58 | 470.66 | 1.76 | 0.000981 |
| Me3 | malic enzyme 3, NADP(+)-dependent, mitochondrial | 389.84 | 185.97 | -2.10 | 0.000169 |
|  | Rn.17918.1 | 49.00 | 18.51 | -2.65 | 0.014628 |
|  | Rn.13394.1 | 1487.64 | 688.13 | -2.16 | 0.000012 |
| Jakmip1 | janus kinase and microtubule interacting protein 1 | 840.65 | 316.34 | -2.66 | 0.000364 |
| Elk3 | ELK3, member of ETS oncogene family | 223.37 | 685.35 | 3.07 | 0.006795 |
| Znf609 | zinc finger protein 609 | 59.21 | 126.89 | 2.14 | 0.005461 |
|  | Rn.24257.1 | 1097.16 | 497.69 | -2.20 | 0.000018 |
|  | Rn.17111.1 | 322.63 | 588.08 | 1.82 | 0.000264 |
| Apln | apelin | 478.39 | 215.23 | -2.22 | 0.000087 |
| Ctla2a | cytotoxic T lymphocyte-associated protein 2 alpha | 49.55 | 330.76 | 6.68 | 0.000987 |
| Spc25 | SPC25, NDC80 kinetochore complex component, homolog (S. cerevisiae) | 55.99 | 282.08 | 5.04 | 0.001127 |
| Ier5l | immediate early response 5-like | 69.66 | 149.23 | 2.14 | 0.011469 |
|  | Rn.23637.1 | 1203.95 | 699.68 | -1.72 | 0.001631 |
| Fam20c | family with sequence similarity 20, member C | 330.01 | 719.15 | 2.18 | 0.000104 |
|  | Rn.62928.1 | 165.66 | 643.14 | 3.88 | 0.000012 |
| Rag1ap1 | recombination activating gene 1 activating protein 1 | 307.16 | 623.76 | 2.03 | 0.002516 |
| Dclk1 | doublecortin-like kinase 1 | 640.76 | 362.16 | -1.77 | 0.000603 |
| Ebf3 | early B-cell factor 3 | 614.90 | 302.76 | -2.03 | 0.001014 |
| Ypel4 | yippee-like 4 (Drosophila) | 419.10 | 197.70 | -2.12 | 0.001055 |
| Tm7sf2 | transmembrane 7 superfamily member 2 | 689.41 | 272.49 | -2.53 | 0.002768 |
|  | Rn.7895.1 | 264.71 | 146.23 | -1.81 | 0.000414 |
| Lrrc10 | leucine-rich repeat-containing 10 | 22.40 | 54.45 | 2.43 | 0.011871 |
| Dram | damage-regulated autophagy modulator | 73.80 | 351.78 | 4.77 | 0.004459 |
| Rps6ka6 | Ribosomal protein S6 kinase polypeptide 6 | 231.80 | 85.71 | -2.70 | 0.000176 |
|  | Rn.19846.1 | 64.00 | 265.21 | 4.14 | 0.002039 |
| RGD1564228 | similar to Naglu | 103.33 | 411.63 | 3.98 | 0.001770 |
| Sorcs3 | sortilin-related VPS10 domain containing receptor 3 | 123.23 | 54.09 | -2.28 | 0.001306 |
| Cdca7l | cell division cycle associated 7 like | 52.64 | 144.64 | 2.75 | 0.000185 |
| Melk | maternal embryonic leucine zipper kinase | 14.56 | 157.60 | 10.82 | 0.003993 |
| Celsr1 | Cadherin, EGF LAG seven-pass G-type receptor 1 (flamingo homolog, Drosophila) | 38.25 | 115.34 | 3.02 | 0.000060 |
| Znf23 | zinc finger protein 23 (KOX 16) | 811.86 | 445.20 | -1.82 | 0.000658 |
| Tceb3 | transcription elongation factor B (SIII), polypeptide 3 | 118.13 | 312.14 | 2.64 | 0.000195 |
| Elmod1 | ELMO/CED-12 domain containing 1 | 2164.52 | 1136.91 | -1.90 | 0.000125 |
|  | Rn.8017.1 | 386.58 | 201.28 | -1.92 | 0.000300 |
| Ptk7 | PTK7 protein tyrosine kinase 7 | 278.79 | 563.54 | 2.02 | 0.010775 |
|  | Rn.20362.1 | 356.72 | 205.66 | -1.73 | 0.002690 |
|  | Rn.47082.1 | 439.23 | 201.40 | -2.18 | 0.000052 |
|  | Rn.51515.1 | 64.31 | 32.33 | -1.99 | 0.001775 |
| Ppp1r14b | protein phosphatase 1, regulatory (inhibitor) subunit 14B | 533.42 | 1236.29 | 2.32 | 0.002473 |
| Tk1 | thymidine kinase 1, soluble | 51.14 | 160.92 | 3.15 | 0.013765 |
| Pycard | PYD and CARD domain containing | 178.28 | 715.83 | 4.02 | 0.000058 |
| Limd2 | LIM domain containing 2 | 188.15 | 592.23 | 3.15 | 0.000165 |
| Grin1 | glutamate receptor, ionotropic, N-methyl D-aspartate 1 | 178.54 | 71.24 | -2.51 | 0.000423 |
| Pttg1ip | pituitary tumor-transforming 1 interacting protein | 1352.71 | 2505.20 | 1.85 | 0.000114 |
| Fdft1 | farnesyl diphosphate farnesyl transferase 1 | 407.99 | 153.10 | -2.66 | 0.000025 |
| Metrnl | meteorin, glial cell differentiation regulator-like | 215.57 | 456.77 | 2.12 | 0.001361 |
|  | Rn.53381.1 | 96.80 | 37.76 | -2.56 | 0.000993 |
|  | Rn.46179.1 | 598.87 | 343.05 | -1.75 | 0.000038 |
|  | Rn.2157.1 | 280.05 | 734.86 | 2.62 | 0.031072 |
| Elavl4 | ELAV (embryonic lethal, abnormal vision, Drosophila)-like 4 (Hu antigen D) | 895.83 | 372.69 | -2.40 | 0.001380 |
| Cnih4 | cornichon homolog 4 (Drosophila) | 274.66 | 596.07 | 2.17 | 0.001988 |
|  | Rn.61553.1 | 168.36 | 60.29 | -2.79 | 0.021899 |
| Hist1h2bh | histone cluster 1, H2bh | 537.78 | 969.68 | 1.80 | 0.001400 |
| Arpc5 | actin related protein 2/3 complex, subunit 5 | 1470.11 | 2767.65 | 1.88 | 0.000174 |
|  | Rn.7958.1 | 446.09 | 2337.61 | 5.24 | 0.003443 |
| Rbms2 | RNA binding motif, single stranded interacting protein 2 | 182.96 | 395.35 | 2.16 | 0.000043 |
| Tmem140 | transmembrane protein 140 | 90.85 | 339.38 | 3.74 | 0.000053 |
| LOC680692 | similar to Golgi phosphoprotein 2 (Golgi membrane protein GP73) | 726.98 | 1336.48 | 1.84 | 0.000137 |
| RGD1562533 | similar to mKIAA0774 protein | 467.38 | 177.35 | -2.64 | 0.000019 |
|  | Rn.48365.1 | 388.61 | 131.04 | -2.97 | 0.002752 |
| Tspyl4 | TSPY-like 4 | 2108.74 | 831.29 | -2.54 | 0.003791 |
| Efemp1 | EGF-containing fibulin-like extracellular matrix protein 1 | 347.04 | 894.93 | 2.58 | 0.001662 |
| Dixdc1 | DIX domain containing 1 | 1336.74 | 723.09 | -1.85 | 0.000014 |
|  | Rn.20630.1 | 510.64 | 311.31 | -1.64 | 0.000082 |
|  | Rn.13297.1 | 422.79 | 834.18 | 1.97 | 0.004198 |
|  | Rn.43557.1 | 126.09 | 567.54 | 4.50 | 0.012087 |
| Traf4af1 | TRAF4 associated factor 1 | 85.25 | 179.35 | 2.10 | 0.001842 |
|  | Rn.17861.1 | 1549.45 | 772.07 | -2.01 | 0.000183 |
|  | Rn.23225.1 | 164.82 | 55.75 | -2.96 | 0.000189 |
| Ttc9b | tetratricopeptide repeat domain 9B | 155.46 | 53.36 | -2.91 | 0.007175 |
|  | Rn.18819.1 | 1430.56 | 416.38 | -3.44 | 0.000240 |
|  | Rn.6397.1 | 103.52 | 337.16 | 3.26 | 0.020694 |
| Nrsn2 | neurensin 2 | 97.66 | 38.92 | -2.51 | 0.002703 |
| Psd4 | pleckstrin and Sec7 domain containing 4 | 15.44 | 84.89 | 5.50 | 0.000073 |
| Syn1 | Synapsin I | 227.97 | 101.99 | -2.24 | 0.000473 |
| Fkbp15 | FK506 binding protein 15 | 384.51 | 739.67 | 1.92 | 0.001592 |
| Syngr3 | synaptogyrin 3 | 1421.65 | 844.42 | -1.68 | 0.000199 |
| Prdm1 | PR domain containing 1, with ZNF domain | 18.06 | 84.67 | 4.69 | 0.000179 |
| RGD1562552 | similar to hypothetical protein LOC340061 | 87.11 | 299.35 | 3.44 | 0.000108 |
| Pcp4l1 | Purkinje cell protein 4-like 1 | 954.41 | 398.47 | -2.40 | 0.002323 |
|  | Rn.23291.1 | 488.23 | 197.70 | -2.47 | 0.004647 |
| LOC688582 | similar to hemicentin 1 | 18.00 | 4.84 | -3.72 | 0.017650 |
|  | Rn.25146.1 | 1523.74 | 846.18 | -1.80 | 0.000141 |
| RGD1305464 | similar to human chromosome 15 open reading frame 39 | 73.62 | 193.06 | 2.62 | 0.000217 |
|  | Rn.39111.1 | 202.25 | 114.54 | -1.77 | 0.000174 |
| Efcab4a | EF-hand calcium binding domain 4A | 101.56 | 229.67 | 2.26 | 0.001656 |
| Bin2 | bridging integrator 2 | 140.51 | 577.37 | 4.11 | 0.000080 |
| RGD1561672 | similar to novel protein | 68.82 | 33.26 | -2.07 | 0.008070 |
| LOC500013 | similar to sterile alpha motif domain containing 9-like /// similar to sterile alpha motif domain containing 9-like /// similar to mKIAA2005 protein /// sterile alpha motif domain containing 9-like | 358.37 | 773.86 | 2.16 | 0.000671 |
|  | Rn.51774.1 | 291.07 | 961.62 | 3.30 | 0.001971 |
| Ppp4c | protein phosphatase 4, catalytic subunit | 97.26 | 205.25 | 2.11 | 0.000912 |
|  | Rn.47579.1 | 68.43 | 33.09 | -2.07 | 0.003004 |
|  | Rn.12683.1 | 1666.13 | 927.31 | -1.80 | 0.000043 |
| Folr2 | folate receptor 2 (fetal) | 42.44 | 1160.47 | 27.34 | 0.001246 |
| Cacna2d3 | calcium channel, voltage-dependent, alpha2/delta subunit 3 | 577.88 | 232.30 | -2.49 | 0.000007 |
| Lmo4 | LIM domain only 4 | 137.56 | 331.47 | 2.41 | 0.018492 |
| RGD1565591 | similar to Ski protein | 1056.96 | 1788.73 | 1.69 | 0.002518 |
| Adfp | adipose differentiation related protein | 224.46 | 1388.88 | 6.19 | 0.000353 |
| Casp3 | caspase 3 | 163.10 | 346.64 | 2.13 | 0.000627 |
| Sh3d19 | SH3 domain containing 19 | 328.83 | 581.10 | 1.77 | 0.004102 |
|  | Rn.49246.1 | 293.70 | 846.39 | 2.88 | 0.000772 |
|  | Rn.38809.1 | 892.18 | 352.19 | -2.53 | 0.000036 |
| Tmem90b | transmembrane protein 90B | 686.98 | 295.13 | -2.33 | 0.000126 |
| Trip13 | thyroid hormone receptor interactor 13 | 46.93 | 159.32 | 3.39 | 0.012175 |
| Cpxm1 | carboxypeptidase X (M14 family), member 1 | 40.98 | 145.21 | 3.54 | 0.027810 |
| Tradd | TNFRSF1A-associated via death domain | 107.16 | 205.87 | 1.92 | 0.002298 |
| RGD1563888 | similar to DNA segment, Chr 16, ERATO Doi 472, expressed | 677.01 | 284.17 | -2.38 | 0.013987 |
| Stx3 | syntaxin 3 | 51.93 | 106.10 | 2.04 | 0.002885 |
| Epb4.1l1 | erythrocyte protein band 4.1-like 1 | 198.77 | 92.54 | -2.15 | 0.000320 |
| Abhd2 | abhydrolase domain containing 2 | 58.29 | 107.92 | 1.85 | 0.003706 |
| Naaa | N-acylethanolamine acid amidase | 549.15 | 1295.58 | 2.36 | 0.000126 |
|  | Rn.35015.1 | 447.45 | 166.71 | -2.68 | 0.000133 |
| Ube2t | ubiquitin-conjugating enzyme E2T (putative) | 12.49 | 75.35 | 6.03 | 0.000782 |
|  | Rn.22773.1 | 2576.44 | 1158.95 | -2.22 | 0.000004 |
| Rps24 | ribosomal protein S24 | 65.21 | 129.65 | 1.99 | 0.002237 |
|  | Rn.40976.1 | 233.81 | 91.25 | -2.56 | 0.000036 |
|  | Rn.18275.1 | 435.77 | 206.25 | -2.11 | 0.000048 |
| Ms4a6b | membrane-spanning 4-domains, subfamily A, member 6B | 81.51 | 1517.90 | 18.62 | 0.002629 |
|  | Rn.22738.1 | 844.92 | 356.07 | -2.37 | 0.000027 |
| LOC687609 | similar to ras homolog gene family, member f | 228.68 | 112.99 | -2.02 | 0.000050 |
|  | Rn.13339.1 | 30.50 | 238.33 | 7.81 | 0.001245 |
| Stra6 | stimulated by retinoic acid gene 6 | 140.38 | 1170.67 | 8.34 | 0.011267 |
| Pric285 | peroxisomal proliferator-activated receptor A interacting complex 285 | 58.09 | 132.82 | 2.29 | 0.002394 |
| Slc25a18 | solute carrier family 25 (mitochondrial carrier), member 18 | 454.45 | 155.04 | -2.93 | 0.002390 |
|  | Rn.13736.1 | 269.64 | 125.77 | -2.14 | 0.001597 |
|  | Rn.50326.1 | 395.62 | 237.52 | -1.67 | 0.000562 |
|  | Rn.34639.1 | 200.33 | 94.15 | -2.13 | 0.005552 |
| Zfp385b | zinc finger protein 385B | 107.43 | 47.29 | -2.27 | 0.002049 |
| Socs5 | suppressor of cytokine signaling 5 | 439.56 | 228.12 | -1.93 | 0.000014 |
| Stau2 | staufen, RNA binding protein, homolog 2 (Drosophila) | 1603.41 | 927.19 | -1.73 | 0.000026 |
| Ckmt1 | creatine kinase, mitochondrial 1 | 1442.61 | 532.93 | -2.71 | 0.002267 |
| Cndp1 | carnosine dipeptidase 1 (metallopeptidase M20 family) | 78.06 | 616.49 | 7.90 | 0.000048 |
| Rnf165 | ring finger protein 165 | 161.20 | 62.61 | -2.57 | 0.002364 |
| Masp1 | mannan-binding lectin serine peptidase 1 | 207.84 | 521.94 | 2.51 | 0.018486 |
| Mlana | melan-A | 11.11 | 32.64 | 2.94 | 0.018586 |
|  | Rn.12227.1 | 350.05 | 168.84 | -2.07 | 0.000244 |
|  | Rn.34130.1 | 173.41 | 95.43 | -1.82 | 0.000717 |
| Slc30a3 | solute carrier family 30 (zinc transporter), member 3 | 344.00 | 189.61 | -1.81 | 0.000564 |
|  | Rn.45081.1 | 105.63 | 41.23 | -2.56 | 0.000224 |
| LOC100363005 | rCG32052-like | 426.65 | 77.82 | -5.48 | 0.001538 |
|  | Rn.28409.1 | 966.23 | 2371.85 | 2.45 | 0.000004 |
| Rprm | reprimo, TP53 dependent G2 arrest mediator candidate | 224.46 | 94.57 | -2.37 | 0.000930 |
|  | Rn.14656.1 | 1189.91 | 461.92 | -2.58 | 0.000024 |
|  | Rn.53376.1 | 77.38 | 38.46 | -2.01 | 0.016878 |
| Plek | pleckstrin | 275.78 | 1136.55 | 4.12 | 0.001972 |
|  | Rn.20292.1 | 175.97 | 95.37 | -1.85 | 0.002154 |
|  | Rn.22587.1 | 405.50 | 752.31 | 1.86 | 0.000235 |
| Rgs10 | regulator of G-protein signaling 10 | 873.01 | 2206.17 | 2.53 | 0.000154 |
|  | Rn.58985.1 | 98.83 | 210.25 | 2.13 | 0.005632 |
|  | Rn.45210.1 | 116.40 | 59.10 | -1.97 | 0.003722 |
| Bst2 | bone marrow stromal cell antigen 2 | 345.99 | 1069.63 | 3.09 | 0.019230 |
| Tuba8 | tubulin, alpha 8 | 284.77 | 115.30 | -2.47 | 0.000359 |
| Lcp2 | lymphocyte cytosolic protein 2 | 69.17 | 383.15 | 5.54 | 0.000317 |
| Ptprc | protein tyrosine phosphatase, receptor type, C | 208.61 | 1507.50 | 7.23 | 0.000241 |
| Cadps2 | Ca++-dependent secretion activator 2 | 296.43 | 151.32 | -1.96 | 0.000898 |
| Kcnj11 | potassium inwardly rectifying channel, subfamily J, member 11 | 452.52 | 173.11 | -2.61 | 0.000014 |
| Tnf | tumor necrosis factor (TNF superfamily, member 2) | 11.80 | 50.45 | 4.27 | 0.009351 |
| LOC689399 | hypothetical protein LOC689399 | 15.29 | 73.74 | 4.82 | 0.000397 |
| Tlcd1 | TLC domain containing 1 | 224.23 | 87.46 | -2.56 | 0.000231 |
|  | Rn.14864.1 | 211.47 | 364.36 | 1.72 | 0.000042 |
| Selp | selectin P | 25.55 | 78.84 | 3.09 | 0.032478 |
|  | Rn.43312.1 | 101.30 | 53.06 | -1.91 | 0.003218 |
|  | Rn.32174.1 | 72.83 | 428.62 | 5.89 | 0.000638 |
|  | Rn.6545.1 | 181.65 | 437.80 | 2.41 | 0.000671 |
|  | Rn.12640.1 | 325.51 | 161.11 | -2.02 | 0.001885 |
|  | Rn.22747.1 | 740.86 | 357.18 | -2.07 | 0.004130 |
|  | Rn.43236.3 | 188.03 | 106.52 | -1.77 | 0.004786 |
|  | Rn.22980.1 | 88.80 | 47.86 | -1.86 | 0.006626 |
| Tnfrsf1b | tumor necrosis factor receptor superfamily, member 1b | 2.72 | 64.92 | 23.86 | 0.000471 |
|  | Rn.54617.1 | 108.95 | 247.82 | 2.27 | 0.000783 |
| Ms4a11 | membrane-spanning 4-domains, subfamily A, member 11 | 24.29 | 189.78 | 7.81 | 0.010059 |
|  | Rn.251.1 | 294.50 | 99.25 | -2.97 | 0.000266 |
|  | Rn.3617.1 | 1081.09 | 479.73 | -2.25 | 0.000120 |
|  | Rn.14752.1 | 490.94 | 860.22 | 1.75 | 0.000670 |
|  | Rn.21882.2 | 398.48 | 171.04 | -2.33 | 0.000502 |
| Ly86 | lymphocyte antigen 86 | 208.71 | 952.45 | 4.56 | 0.001124 |
| Cfp | complement factor properdin | 8.77 | 157.06 | 17.90 | 0.025026 |
| Scgb1c1 | secretoglobin, family 1C, member 1 | 3.50 | 13.53 | 3.86 | 0.032998 |
| LOC685111 | similar to paired immunoglobin-like type 2 receptor beta /// similar to cell surface receptor FDFACT | 46.77 | 181.89 | 3.89 | 0.000731 |
| RGD1310773 | similar to hypothetical protein FLJ31810 | 143.57 | 48.00 | -2.99 | 0.000031 |
|  | Rn.19450.2 | 93.82 | 261.69 | 2.79 | 0.000043 |
| Lpcat3 | lysophosphatidylcholine acyltransferase 3 | 98.28 | 237.01 | 2.41 | 0.004438 |
|  | Rn.14823.1 | 56.76 | 21.18 | -2.68 | 0.001103 |
| Gpr150 | G protein-coupled receptor 150 | 110.42 | 41.35 | -2.67 | 0.000923 |
|  | Rn.25146.2 | 309.45 | 164.95 | -1.88 | 0.003374 |
| Itsn1 | intersectin 1 (SH3 domain protein) | 144.09 | 258.91 | 1.80 | 0.000255 |
| Csrp3 | cysteine and glycine-rich protein 3 | 12.35 | 44.62 | 3.61 | 0.006201 |
| Fcgr2a | Fc fragment of IgG, low affinity IIa, receptor (CD32) /// Fc gamma receptor II beta | 322.55 | 3192.00 | 9.90 | 0.000597 |
| Camk2b | calcium/calmodulin-dependent protein kinase II beta | 398.68 | 126.39 | -3.15 | 0.003324 |
| Renbp | renin binding protein | 160.19 | 317.52 | 1.98 | 0.004957 |
| Il1b | interleukin 1 beta | 36.09 | 1330.73 | 36.87 | 0.003612 |
| B4galnt1 | beta-1,4-N-acetyl-galactosaminyl transferase 1 | 140.83 | 52.59 | -2.68 | 0.000398 |
| Mmp9 | matrix metallopeptidase 9 | 8.36 | 159.65 | 19.11 | 0.010366 |
| Kcnk13 | potassium channel, subfamily K, member 13 | 72.87 | 213.02 | 2.92 | 0.000056 |
| Mapk12 | mitogen-activated protein kinase 12 | 338.38 | 190.07 | -1.78 | 0.000302 |
| Tpm3 | tropomyosin 3, gamma | 50.84 | 126.13 | 2.48 | 0.002925 |
| Fzd2 | frizzled homolog 2 (Drosophila) | 24.80 | 60.78 | 2.45 | 0.003474 |
| Rpl15 | ribosomal protein L15 | 2475.04 | 4142.52 | 1.67 | 0.000014 |
|  | Rn.32307.1 | 943.05 | 1808.61 | 1.92 | 0.000047 |
| Chchd10 | coiled-coil-helix-coiled-coil-helix domain containing 10 | 3185.25 | 1718.86 | -1.85 | 0.000244 |
| Stxbp1 | syntaxin binding protein 1 | 1241.92 | 649.19 | -1.91 | 0.000339 |
| Axl | Axl receptor tyrosine kinase | 519.18 | 1880.63 | 3.62 | 0.000464 |
| Ak2 | adenylate kinase 2 | 311.69 | 636.27 | 2.04 | 0.000655 |
| Sar1a | SAR1 homolog A (S. cerevisiae) | 292.06 | 526.76 | 1.80 | 0.000950 |
| Cplx1 | complexin 1 | 3310.77 | 1669.07 | -1.98 | 0.000081 |
| Itgb5 | integrin, beta 5 | 212.49 | 444.27 | 2.09 | 0.000126 |
|  | Rn.16640.1 | 1212.51 | 593.38 | -2.04 | 0.000007 |
| Akap13 | A kinase (PRKA) anchor protein 13 | 305.41 | 685.01 | 2.24 | 0.000934 |
| Adarb1 | adenosine deaminase, RNA-specific, B1 | 2368.67 | 1269.99 | -1.87 | 0.000120 |
| B3galnt1 | beta-1,3-N-acetylgalactosaminyltransferase 1 | 753.35 | 1299.24 | 1.72 | 0.005605 |
| Cyb561 | cytochrome b-561 | 372.26 | 190.41 | -1.96 | 0.000121 |
| Fam198b | family with sequence similarity 198, member B | 224.29 | 799.18 | 3.56 | 0.003261 |
| Cxcl13 | chemokine (C-X-C motif) ligand 13 | 55.98 | 4089.67 | 73.05 | 0.005268 |
|  | Rn.14626.1 | 162.66 | 76.37 | -2.13 | 0.000592 |
| sep-06 | Septin 6 | 706.38 | 276.63 | -2.55 | 0.000052 |
| LOC100364294 | armadillo repeat containing 3-like | 49.51 | 150.00 | 3.03 | 0.000007 |
| Fam110b | family with sequence similarity 110, member B | 399.10 | 180.75 | -2.21 | 0.000154 |
|  | Rn.22419.1 | 1185.37 | 622.90 | -1.90 | 0.000789 |
|  | Rn.49222.1 | 145.68 | 50.60 | -2.88 | 0.014820 |
|  | Rn.6416.1 | 14.07 | 80.24 | 5.70 | 0.003004 |
|  | Rn.24016.1 | 109.43 | 35.81 | -3.06 | 0.000780 |
| Wnk2 | WNK lysine deficient protein kinase 2 | 586.01 | 265.78 | -2.20 | 0.000668 |
|  | Rn.63607.1 | 136.51 | 38.12 | -3.58 | 0.000222 |
| Ifnar1 | interferon (alpha, beta and omega) receptor 1 | 96.28 | 221.63 | 2.30 | 0.000009 |
| Rgs1 | regulator of G-protein signaling 1 | 25.61 | 438.16 | 17.11 | 0.000034 |
| Npm1 | nucleophosmin (nucleolar phosphoprotein B23, numatrin) | 186.82 | 505.96 | 2.71 | 0.000563 |
| Coro1b | coronin, actin-binding protein, 1B | 389.28 | 857.63 | 2.20 | 0.000005 |
| Rab11a | RAB11a, member RAS oncogene family | 1387.73 | 2211.86 | 1.59 | 0.000082 |
| Vamp5 | vesicle-associated membrane protein 5 | 304.16 | 732.44 | 2.41 | 0.000240 |
| Nudt4 | nudix (nucleoside diphosphate linked moiety X)-type motif 4 | 529.06 | 1169.22 | 2.21 | 0.000046 |
| H3f3b | H3 histone, family 3B | 1514.33 | 2563.13 | 1.69 | 0.000980 |
| Hdlbp | high density lipoprotein binding protein | 276.17 | 492.30 | 1.78 | 0.000398 |
| Cpsf4 | cleavage and polyadenylation specific factor 4 | 293.71 | 534.57 | 1.82 | 0.000690 |
| Rps5 | ribosomal protein S5 | 2555.27 | 4350.47 | 1.70 | 0.000578 |
| RGD1563348 | similar to Selenoprotein H | 666.97 | 1144.78 | 1.72 | 0.000159 |
| Npc2 | Niemann-Pick disease, type C2 | 2904.27 | 5711.69 | 1.97 | 0.000013 |
| Slc38a6 | solute carrier family 38, member 6 | 115.24 | 220.85 | 1.92 | 0.002385 |
| Slc38a10 | solute carrier family 38, member 10 | 460.72 | 831.78 | 1.81 | 0.000056 |
| Eps15 | epidermal growth factor receptor pathway substrate 15 | 315.64 | 593.80 | 1.88 | 0.000448 |
| Ube2d3 | ubiquitin-conjugating enzyme E2D 3 (UBC4/5 homolog, yeast) | 386.63 | 878.90 | 2.27 | 0.002336 |
| Erap1 | endoplasmic reticulum aminopeptidase 1 | 569.07 | 1243.02 | 2.18 | 0.001188 |
|  | Rn.63390.1 | 269.34 | 82.90 | -3.25 | 0.005057 |
|  | Rn.47760.1 | 69.19 | 31.52 | -2.20 | 0.011138 |
|  | Rn.76386.1 | 41.19 | 17.86 | -2.31 | 0.005856 |
| Shc1 | SHC (Src homology 2 domain containing) transforming protein 1 | 176.07 | 801.07 | 4.55 | 0.000236 |
| Tmem130 | transmembrane protein 130 | 3356.43 | 1813.83 | -1.85 | 0.000131 |
|  | Rn.40782.1 | 1467.51 | 728.96 | -2.01 | 0.000014 |
| Bnip2 | BCL2/adenovirus E1B interacting protein 2 | 112.65 | 257.97 | 2.29 | 0.001866 |
|  | Rn.3093.1 | 264.64 | 101.43 | -2.61 | 0.000691 |
| Fgf12 | Fibroblast growth factor 12 | 606.13 | 302.02 | -2.01 | 0.000014 |
| Chdh | choline dehydrogenase | 51.98 | 169.03 | 3.25 | 0.000006 |
| Rab27a | RAB27A, member RAS oncogene family | 287.58 | 538.20 | 1.87 | 0.001041 |
|  | Rn.8244.1 | 134.37 | 1615.19 | 12.02 | 0.001165 |
|  | Rn.48615.1 | 533.78 | 233.59 | -2.29 | 0.000023 |
| Zc4h2 | zinc finger, C4H2 domain containing | 588.12 | 343.12 | -1.71 | 0.000615 |
| Lpar6 | lysophosphatidic acid receptor 6 | 125.18 | 309.49 | 2.47 | 0.000102 |
| Atp6v1g2 | ATPase, H+ transporting, lysosomal V1 subunit G2 | 1330.57 | 767.64 | -1.73 | 0.001010 |
| Trim25 | tripartite motif-containing 25 | 114.53 | 360.44 | 3.15 | 0.023790 |
| Elovl4 | Elongation of very long chain fatty acids (FEN1/Elo2, SUR4/Elo3, yeast)-like 4 | 954.30 | 435.89 | -2.19 | 0.000201 |
| Nipa2 | non imprinted in Prader-Willi/Angelman syndrome 2 homolog (human) | 344.06 | 717.01 | 2.08 | 0.001893 |
| Gng12 | guanine nucleotide binding protein (G protein), gamma 12 | 465.49 | 881.47 | 1.89 | 0.001384 |
|  | Rn.37275.1 | 97.50 | 190.91 | 1.96 | 0.017781 |
| Lrrc40 | leucine rich repeat containing 40 | 422.13 | 754.72 | 1.79 | 0.000021 |
|  | Rn.24708.1 | 742.71 | 405.65 | -1.83 | 0.000464 |
|  | Rn.24296.1 | 1128.47 | 635.09 | -1.78 | 0.004358 |
|  | Rn.57709.1 | 1340.03 | 478.67 | -2.80 | 0.000722 |
|  | Rn.17666.1 | 101.44 | 289.01 | 2.85 | 0.001315 |
| Ano3 | Anoctamin 3 | 662.83 | 215.91 | -3.07 | 0.000040 |
| RGD1311186 | similar to RIKEN cDNA 1810014F10 gene | 449.67 | 269.13 | -1.67 | 0.001878 |
| Agap3 | ArfGAP with GTPase domain, ankyrin repeat and PH domain 3 | 761.51 | 375.01 | -2.03 | 0.000147 |
| Znrf2 | zinc and ring finger 2 | 178.64 | 332.66 | 1.86 | 0.000082 |
|  | Rn.61635.1 | 142.69 | 56.91 | -2.51 | 0.000578 |
|  | Rn.41806.1 | 438.75 | 171.52 | -2.56 | 0.000376 |
| Flt3 | fms-related tyrosine kinase 3 | 92.62 | 40.04 | -2.31 | 0.000423 |
|  | Rn.29913.1 | 879.91 | 436.28 | -2.02 | 0.000158 |
|  | Rn.16218.1 | 987.80 | 599.34 | -1.65 | 0.000037 |
|  | Rn.17920.1 | 175.97 | 326.10 | 1.85 | 0.002815 |
|  | Rn.15840.1 | 17.50 | 55.73 | 3.18 | 0.000333 |
| Slfn2 | schlafen 2 | 104.56 | 924.37 | 8.84 | 0.000340 |
|  | Rn.15929.1 | 529.44 | 254.39 | -2.08 | 0.001542 |
|  | Rn.17683.1 | 142.86 | 52.05 | -2.74 | 0.006008 |
|  | Rn.28084.1 | 323.63 | 88.45 | -3.66 | 0.002827 |
| Cd86 | CD86 molecule | 42.67 | 362.38 | 8.49 | 0.000222 |
|  | Rn.28251.1 | 82.39 | 170.06 | 2.06 | 0.000265 |
|  | Rn.23377.1 | 47.19 | 100.37 | 2.13 | 0.003452 |
|  | Rn.40749.1 | 266.24 | 139.35 | -1.91 | 0.000061 |
| Cdt1 | chromatin licensing and DNA replication factor 1 | 54.43 | 132.04 | 2.43 | 0.000802 |
|  | Rn.27931.1 | 592.50 | 326.14 | -1.82 | 0.000167 |
|  | Rn.23392.1 | 78.31 | 32.98 | -2.37 | 0.002544 |
| LOC100364559 | gene model 691, (NCBI)-like | 311.15 | 116.06 | -2.68 | 0.000101 |
| Gng13 | guanine nucleotide binding protein (G protein), gamma 13 | 278.60 | 79.15 | -3.52 | 0.000318 |
|  | Rn.35311.1 | 146.04 | 274.80 | 1.88 | 0.008616 |
|  | Rn.37749.1 | 352.65 | 745.21 | 2.11 | 0.000140 |
| LOC679651 | hypothetical protein LOC679651 | 975.49 | 472.34 | -2.07 | 0.000251 |
|  | Rn.26537.1 | 128.62 | 615.29 | 4.78 | 0.027254 |
| Hspa4l | heat shock protein 4 like | 1009.55 | 541.24 | -1.87 | 0.000072 |
|  | Rn.24818.1 | 107.72 | 194.08 | 1.80 | 0.010707 |
|  | Rn.28130.1 | 883.14 | 487.27 | -1.81 | 0.000031 |
|  | Rn.46824.1 | 274.42 | 84.24 | -3.26 | 0.000195 |
| Mad2l1 | MAD2 mitotic arrest deficient-like 1 (yeast) | 21.46 | 47.73 | 2.22 | 0.012234 |
| Nfkbiz | nuclear factor of kappa light polypeptide gene enhancer in B-cells inhibitor, zeta | 122.56 | 767.80 | 6.26 | 0.000425 |
|  | Rn.18164.1 | 33.62 | 110.72 | 3.29 | 0.000757 |
| Chst14 | carbohydrate (N-acetylgalactosamine 4-0) sulfotransferase 14 | 73.95 | 143.25 | 1.94 | 0.003193 |
| Spic | Spi-C transcription factor (Spi-1/PU.1 related) | 7.41 | 22.66 | 3.06 | 0.005217 |
| Tcf7l2 | transcription factor 7-like 2 (T-cell specific, HMG-box) | 21.63 | 75.50 | 3.49 | 0.008077 |
|  | Rn.45926.1 | 719.99 | 368.60 | -1.95 | 0.000228 |
|  | Rn.66290.1 | 1482.19 | 819.22 | -1.81 | 0.000603 |
|  | Rn.11766.1 | 290.31 | 664.07 | 2.29 | 0.000048 |
|  | Rn.64207.1 | 584.07 | 237.89 | -2.46 | 0.008145 |
|  | Rn.35356.1 | 115.34 | 228.33 | 1.98 | 0.009293 |
|  | Rn.38231.1 | 968.66 | 531.42 | -1.82 | 0.000031 |
|  | Rn.47021.1 | 791.73 | 240.88 | -3.29 | 0.000006 |
|  | Rn.41488.1 | 113.75 | 276.12 | 2.43 | 0.003519 |
|  | Rn.23008.1 | 635.90 | 253.24 | -2.51 | 0.002073 |
| Pcnxl2 | pecanex-like 2 (Drosophila) | 127.02 | 57.54 | -2.21 | 0.002669 |
|  | Rn.25296.1 | 465.01 | 206.48 | -2.25 | 0.000447 |
|  | Rn.24020.1 | 407.82 | 147.01 | -2.77 | 0.008267 |
| Rnft2 | ring finger protein, transmembrane 2 | 577.64 | 319.93 | -1.81 | 0.000267 |
| Lrp3 | low density lipoprotein receptor-related protein 3 | 119.21 | 62.08 | -1.92 | 0.007244 |
| Kank4 | KN motif and ankyrin repeat domains 4 | 250.65 | 117.73 | -2.13 | 0.000179 |
| LOC100363332 | caspase recruitment domain family, member 11 | 156.01 | 441.73 | 2.83 | 0.000691 |
|  | Rn.49054.1 | 137.71 | 59.84 | -2.30 | 0.008270 |
|  | Rn.46837.1 | 442.26 | 206.55 | -2.14 | 0.004093 |
|  | Rn.40971.1 | 338.40 | 179.28 | -1.89 | 0.003339 |
|  | Rn.15170.1 | 58.52 | 226.44 | 3.87 | 0.000345 |
| Frrs1 | ferric-chelate reductase 1 | 22.92 | 92.60 | 4.04 | 0.007403 |
| Grem2 | gremlin 2, cysteine knot superfamily, homolog (Xenopus laevis) | 406.69 | 186.69 | -2.18 | 0.000082 |
| Lhfpl2 | lipoma HMGIC fusion partner-like 2 | 141.76 | 477.94 | 3.37 | 0.000458 |
| Dctd | dCMP deaminase | 109.95 | 314.59 | 2.86 | 0.000095 |
| Ms4a7 | membrane-spanning 4-domains, subfamily A, member 7 | 9.41 | 179.50 | 19.07 | 0.003380 |
| Slc43a1 | solute carrier family 43, member 1 | 41.25 | 109.65 | 2.66 | 0.004871 |
| KIFC2 | kinesin family member C2 | 364.54 | 193.52 | -1.88 | 0.000279 |
| Luzp5 | Leucine zipper protein 5 | 43.41 | 97.07 | 2.24 | 0.005229 |
|  | Rn.47833.1 | 6.73 | 23.78 | 3.53 | 0.022453 |
|  | Rn.20910.1 | 1741.35 | 895.82 | -1.94 | 0.000039 |
| Fancd2 | Fanconi anemia, complementation group D2 | 43.88 | 103.96 | 2.37 | 0.001024 |
| Pptc7 | PTC7 protein phosphatase homolog (S. cerevisiae) | 84.40 | 42.21 | -2.00 | 0.012687 |
| Chodl | chondrolectin | 1894.95 | 824.93 | -2.30 | 0.000039 |
|  | Rn.42282.1 | 317.45 | 150.65 | -2.11 | 0.001117 |
|  | Rn.24358.1 | 1256.07 | 630.10 | -1.99 | 0.000088 |
| Ncaph | non-SMC condensin I complex, subunit H | 79.02 | 183.32 | 2.32 | 0.012927 |
| Igsf21 | immunoglobin superfamily, member 21 /// similar to immunoglobin superfamily, member 21 | 293.15 | 167.40 | -1.75 | 0.000657 |
|  | Rn.15598.1 | 369.91 | 193.61 | -1.91 | 0.000253 |
| Rlbp1 | retinaldehyde binding protein 1 | 858.90 | 469.78 | -1.83 | 0.000065 |
| Rassf4 | Ras association (RalGDS/AF-6) domain family member 4 | 177.80 | 377.27 | 2.12 | 0.005484 |
| LOC687183 | similar to CG5645-PA | 104.22 | 254.83 | 2.45 | 0.003579 |
| Nol4 | nucleolar protein 4 | 168.01 | 79.98 | -2.10 | 0.000344 |
|  | Rn.50362.1 | 168.29 | 84.94 | -1.98 | 0.002462 |
| Ogfod1 | 2-oxoglutarate and iron-dependent oxygenase domain containing 1 | 467.76 | 248.20 | -1.88 | 0.000052 |
|  | Rn.33919.1 | 156.96 | 66.01 | -2.38 | 0.000649 |
| Nkrf | NFKB repressing factor | 564.36 | 327.93 | -1.72 | 0.000644 |
|  | Rn.27130.1 | 237.87 | 504.29 | 2.12 | 0.000030 |
| Dapp1 | dual adaptor of phosphotyrosine and 3-phosphoinositides | 36.06 | 81.21 | 2.25 | 0.008201 |
|  | Rn.8800.1 | 103.52 | 228.73 | 2.21 | 0.012870 |
|  | Rn.23485.2 | 707.47 | 368.50 | -1.92 | 0.000277 |
|  | Rn.40894.1 | 186.81 | 98.38 | -1.90 | 0.002520 |
| Tifab | TRAF-interacting protein with forkhead-associated domain, family member B | 14.49 | 121.60 | 8.39 | 0.010299 |
|  | Rn.45384.1 | 197.30 | 108.42 | -1.82 | 0.000396 |
| RGD1304878 | Similar to 2410024A21Rik protein | 30.25 | 10.81 | -2.80 | 0.005084 |
|  | Rn.23777.1 | 16.03 | 316.08 | 19.72 | 0.001785 |
| Ankrd33b | ankyrin repeat domain 33B | 28.00 | 100.37 | 3.59 | 0.002971 |
| Slamf9 | SLAM family member 9 | 27.71 | 435.55 | 15.72 | 0.000256 |
|  | Rn.34019.1 | 662.87 | 267.24 | -2.48 | 0.010910 |
|  | Rn.20751.1 | 297.16 | 121.21 | -2.45 | 0.000701 |
| Fhod1 | formin homology 2 domain containing 1 | 63.76 | 200.12 | 3.14 | 0.000708 |
| Mdfic | MyoD family inhibitor domain containing | 33.28 | 132.83 | 3.99 | 0.001921 |
|  | Rn.23362.1 | 31.84 | 124.22 | 3.90 | 0.005079 |
| Gimap6 | GTPase, IMAP family member 6 | 21.97 | 55.26 | 2.51 | 0.014660 |
| Ppp2r5e | protein phosphatase 2, regulatory subunit B', epsilon isoform | 125.97 | 61.55 | -2.05 | 0.001169 |
|  | Rn.38591.1 | 89.16 | 26.31 | -3.39 | 0.000615 |
|  | Rn.39305.1 | 7.22 | 18.32 | 2.54 | 0.013370 |
|  | Rn.61067.1 | 10.42 | 55.81 | 5.36 | 0.009763 |
| LOC688455 | Hypothetical protein LOC688455 | 57.21 | 30.53 | -1.87 | 0.004128 |
| Fam19a4 | family with sequence similarity 19 (chemokine (C-C motif)-like), member A4 | 123.33 | 38.41 | -3.21 | 0.000007 |
|  | Rn.46461.1 | 143.23 | 66.72 | -2.15 | 0.003445 |
|  | Rn.39066.1 | 1181.09 | 626.21 | -1.89 | 0.003764 |
|  | Rn.57843.1 | 100.29 | 44.06 | -2.28 | 0.009634 |
|  | Rn.46358.1 | 275.76 | 114.60 | -2.41 | 0.001633 |
| Lpxn | leupaxin | 151.72 | 635.12 | 4.19 | 0.000488 |
| Uhrf1 | ubiquitin-like with PHD and ring finger domains 1 | 16.71 | 53.01 | 3.17 | 0.031847 |
| Arhgap21 | Rho GTPase activating protein 21 | 136.59 | 38.98 | -3.50 | 0.031493 |
|  | Rn.20645.1 | 108.85 | 247.95 | 2.28 | 0.010500 |
|  | Rn.59798.1 | 59.59 | 24.17 | -2.47 | 0.006704 |
|  | Rn.50157.1 | 200.85 | 83.37 | -2.41 | 0.017160 |
| RGD1309873 | Similar to hypothetical protein BC010003 | 422.33 | 158.76 | -2.66 | 0.000472 |
| Kcnab1 | potassium voltage-gated channel, shaker-related subfamily, beta member 1 | 1058.79 | 458.18 | -2.31 | 0.000244 |
| LOC500947 | hypothetical gene supported by BC088439 | 130.77 | 55.76 | -2.35 | 0.001795 |
|  | Rn.49521.1 | 49.12 | 12.89 | -3.81 | 0.047697 |
|  | Rn.63591.1 | 42.61 | 12.24 | -3.48 | 0.009655 |
| Pou6f1 | POU class 6 homeobox 1 | 197.85 | 99.86 | -1.98 | 0.000172 |
|  | Rn.59729.1 | 45.67 | 7.20 | -6.34 | 0.004101 |
|  | Rn.46429.1 | 251.71 | 131.46 | -1.91 | 0.000486 |
|  | Rn.50140.1 | 36.33 | 14.96 | -2.43 | 0.030675 |
|  | Rn.58765.1 | 32.88 | 14.07 | -2.34 | 0.022933 |
| H2afx | H2A histone family, member X | 24.42 | 46.59 | 1.91 | 0.002161 |
| LOC679974 | similar to transcription elongation factor A (SII)-like 3 /// hypothetical protein LOC680282 /// transcription elongation factor A (SII)-like 3 | 665.49 | 331.73 | -2.01 | 0.000128 |
| LOC679974 | similar to transcription elongation factor A (SII)-like 3 /// transcription elongation factor A (SII)-like 3 | 1236.09 | 658.54 | -1.88 | 0.000104 |
| LOC680282 | hypothetical protein LOC680282 | 647.40 | 224.26 | -2.89 | 0.000031 |
|  | Rn.46219.1 | 419.53 | 151.48 | -2.77 | 0.000096 |
|  | Rn.18432.1 | 44.52 | 136.48 | 3.07 | 0.011829 |
| Cdk2 | cyclin dependent kinase 2 | 17.00 | 47.90 | 2.82 | 0.021562 |
|  | Rn.35480.1 | 10.84 | 27.91 | 2.57 | 0.021368 |
|  | Rn.50875.1 | 75.62 | 34.32 | -2.20 | 0.004478 |
|  | Rn.32730.1 | 146.95 | 71.34 | -2.06 | 0.005235 |
| Slc35d3 | solute carrier family 35, member D3 | 147.29 | 52.19 | -2.82 | 0.001748 |
|  | Rn.45245.1 | 695.76 | 285.47 | -2.44 | 0.000221 |
| LOC308990 | hypothetical protein LOC308990 | 16.34 | 86.20 | 5.27 | 0.001784 |
| LOC308990 | hypothetical protein LOC308990 | 22.64 | 68.33 | 3.02 | 0.015474 |
| Isy1 | ISY1 splicing factor homolog (S. cerevisiae) | 49.25 | 114.89 | 2.33 | 0.004085 |
| Zfp428 | zinc finger protein 428 | 30.93 | 11.47 | -2.70 | 0.015674 |
|  | Rn.46057.1 | 244.44 | 123.88 | -1.97 | 0.000495 |
| Mab21l2 | mab-21-like 2 (C. elegans) | 135.89 | 58.89 | -2.31 | 0.001435 |
| Cmklr1 | chemokine-like receptor 1 | 82.35 | 153.33 | 1.86 | 0.001354 |
|  | Rn.28042.1 | 361.80 | 135.63 | -2.67 | 0.000163 |
| Dbndd1 | dysbindin (dystrobrevin binding protein 1) domain containing 1 | 198.56 | 84.60 | -2.35 | 0.000070 |
|  | Rn.66605.1 | 64.11 | 29.27 | -2.19 | 0.044516 |
|  | Rn.12349.1 | 81.91 | 36.89 | -2.22 | 0.000667 |
| RGD1560755 | similar to D8Ertd354e protein | 525.28 | 256.17 | -2.05 | 0.013880 |
|  | Rn.40779.1 | 2790.20 | 1145.63 | -2.44 | 0.000196 |
|  | Rn.50688.1 | 11.38 | 62.99 | 5.53 | 0.000624 |
|  | Rn.59850.1 | 144.02 | 54.77 | -2.63 | 0.019699 |
|  | Rn.54092.1 | 52.52 | 122.35 | 2.33 | 0.031787 |
|  | Rn.58744.1 | 176.16 | 67.83 | -2.60 | 0.001529 |
|  | Rn.64322.1 | 27.95 | 88.48 | 3.17 | 0.047353 |
| Cdc45l | CDC45 cell division cycle 45-like (S. cerevisiae) | 46.68 | 109.71 | 2.35 | 0.003475 |
|  | Rn.60839.1 | 140.87 | 59.62 | -2.36 | 0.014795 |
|  | Rn.13581.1 | 117.81 | 215.89 | 1.83 | 0.001836 |
| Prcp | prolylcarboxypeptidase (angiotensinase C) | 134.84 | 505.28 | 3.75 | 0.000825 |
| Ska2 | spindle and kinetochore associated complex subunit 2 | 186.57 | 372.67 | 2.00 | 0.007428 |
|  | Rn.24130.1 | 264.82 | 445.74 | 1.68 | 0.000174 |
|  | Rn.23017.1 | 218.65 | 114.26 | -1.91 | 0.000909 |
| Rtp4 | receptor (chemosensory) transporter protein 4 | 167.13 | 613.72 | 3.67 | 0.002636 |
|  | Rn.63052.1 | 53.86 | 27.54 | -1.96 | 0.001556 |
| Gngt2 | guanine nucleotide binding protein (G protein), gamma transducing activity polypeptide 2 | 49.56 | 802.20 | 16.19 | 0.000590 |
| Vps13d | vacuolar protein sorting 13 homolog D (S. cerevisiae) | 253.31 | 137.12 | -1.85 | 0.008251 |
| Arid5a | AT rich interactive domain 5A (Mrf1 like) | 96.71 | 206.17 | 2.13 | 0.000849 |
| Pprc1 | peroxisome proliferator-activated receptor gamma, coactivator-related 1 | 181.44 | 725.33 | 4.00 | 0.002901 |
| LOC498662 | similar to RIKEN cDNA 2610019F03 | 463.83 | 229.35 | -2.02 | 0.000260 |
|  | Rn.21975.1 | 685.22 | 310.69 | -2.21 | 0.001203 |
|  | Rn.39063.1 | 552.47 | 237.69 | -2.32 | 0.003387 |
|  | Rn.45452.1 | 220.08 | 96.78 | -2.27 | 0.000065 |
| Pgm2l1 | phosphoglucomutase 2-like 1 | 697.96 | 275.70 | -2.53 | 0.001835 |
| Lamc2 | laminin, gamma 2 | 6.41 | 23.17 | 3.61 | 0.002457 |
|  | Rn.43961.1 | 115.29 | 2039.00 | 17.69 | 0.003668 |
| Col15a1 | collagen, type XV, alpha 1 | 31.14 | 166.26 | 5.34 | 0.000941 |
| Extl2 | exostoses (multiple)-like 2 | 520.21 | 282.58 | -1.84 | 0.000081 |
| Cxcl11 | chemokine (C-X-C motif) ligand 11 | 21.10 | 806.52 | 38.23 | 0.005826 |
| RGD1308251 | similar to RIKEN cDNA 2810405K02 | 838.01 | 508.19 | -1.65 | 0.000135 |
|  | Rn.22959.1 | 437.31 | 182.05 | -2.40 | 0.000170 |
| Lppr4 | lipid phosphate phosphatase-related protein type 4 | 779.02 | 451.89 | -1.72 | 0.003991 |
| St6galnac2 | ST6 (alpha-N-acetyl-neuraminyl-2,3-beta-galactosyl-1,3)-N-acetylgalactosaminide alpha-2,6-sialyltransferase 2 | 38.87 | 86.42 | 2.22 | 0.003689 |
| Vil1 | villin 1 | 11.60 | 35.51 | 3.06 | 0.008242 |
| Fcgrt | Fc fragment of IgG, receptor, transporter, alpha | 5.19 | 25.19 | 4.86 | 0.019037 |
|  | Rn.24198.1 | 1012.53 | 528.44 | -1.92 | 0.000778 |
| Ube2ql1 | ubiquitin-conjugating enzyme E2Q family-like 1 | 680.11 | 284.66 | -2.39 | 0.000026 |
| Dem1 | defects in morphology 1 homolog (S. cerevisiae) | 137.24 | 385.53 | 2.81 | 0.000190 |
| Pgrmc2 | progesterone receptor membrane component 2 | 65.32 | 131.04 | 2.01 | 0.002868 |
|  | Rn.46528.1 | 230.74 | 127.96 | -1.80 | 0.000624 |
|  | Rn.44024.1 | 85.02 | 195.60 | 2.30 | 0.002039 |
|  | Rn.28621.1 | 775.03 | 261.34 | -2.97 | 0.000078 |
| Ttk | Ttk protein kinase | 34.30 | 198.02 | 5.77 | 0.002488 |
| Cttnbp2nl | CTTNBP2 N-terminal like | 642.98 | 1345.88 | 2.09 | 0.000379 |
| Gas2 | growth arrest-specific 2 | 374.99 | 149.69 | -2.51 | 0.000417 |
|  | Rn.19477.1 | 247.89 | 77.73 | -3.19 | 0.002497 |
|  | Rn.28677.1 | 252.92 | 92.72 | -2.73 | 0.000270 |
| Ovol1 | Ovo-like 1(Drosophila) | 69.07 | 31.86 | -2.17 | 0.034300 |
| Lix1 | Lix1 homolog (chicken) | 377.00 | 139.09 | -2.71 | 0.000134 |
|  | Rn.37950.1 | 300.66 | 151.65 | -1.98 | 0.000967 |
| Snrpf | small nuclear ribonucleoprotein polypeptide F | 684.96 | 1250.09 | 1.83 | 0.000445 |
|  | Rn.43926.1 | 90.95 | 42.88 | -2.12 | 0.003360 |
| Irf5 | interferon regulatory factor 5 | 70.83 | 179.27 | 2.53 | 0.000367 |
|  | Rn.32562.1 | 1315.61 | 691.41 | -1.90 | 0.000029 |
| Rbm11 | RNA binding motif protein 11 | 274.91 | 98.51 | -2.79 | 0.000561 |
|  | Rn.46445.1 | 103.09 | 55.42 | -1.86 | 0.001994 |
| Mapk1ip1l | mitogen-activated protein kinase 1 interacting protein 1-like | 76.05 | 191.47 | 2.52 | 0.002255 |
| Ccna2 | cyclin A2 | 22.36 | 308.03 | 13.78 | 0.006265 |
|  | Rn.17187.1 | 70.52 | 412.59 | 5.85 | 0.000419 |
| Adamts19 | ADAM metallopeptidase with thrombospondin type 1 motif, 19 | 49.23 | 24.47 | -2.01 | 0.002791 |
|  | Rn.49237.1 | 135.57 | 61.13 | -2.22 | 0.001537 |
| Parp14 | poly (ADP-ribose) polymerase family, member 14 | 11.15 | 51.76 | 4.64 | 0.009531 |
|  | Rn.45877.1 | 73.40 | 36.57 | -2.01 | 0.000741 |
| Fam164a | family with sequence similarity 164, member A | 787.86 | 465.34 | -1.69 | 0.000483 |
| Uba7 | ubiquitin-like modifier activating enzyme 7 | 14.00 | 55.76 | 3.98 | 0.000615 |
| Rdx | Radixin | 492.12 | 199.31 | -2.47 | 0.000039 |
| LOC500974 | Similar to CDNA sequence BC024479 | 84.24 | 42.47 | -1.98 | 0.003385 |
|  | Rn.15637.1 | 167.36 | 67.07 | -2.50 | 0.000922 |
|  | Rn.43480.1 | 199.28 | 373.07 | 1.87 | 0.001212 |
| Mfsd1 | major facilitator superfamily domain containing 1 | 251.81 | 652.03 | 2.59 | 0.002151 |
| Dnase1l1 | deoxyribonuclease 1-like 1 | 207.26 | 468.28 | 2.26 | 0.001385 |
| Tnfsf13 | tumor necrosis factor (ligand) superfamily, member 13 | 133.23 | 298.59 | 2.24 | 0.005435 |
|  | Rn.22843.1 | 34.99 | 71.51 | 2.04 | 0.001029 |
|  | Rn.28595.1 | 136.23 | 63.51 | -2.14 | 0.003360 |
|  | Rn.18546.1 | 33.37 | 126.90 | 3.80 | 0.007966 |
| Cd22 | CD22 molecule | 16.09 | 43.32 | 2.69 | 0.004409 |
|  | Rn.22530.1 | 66.72 | 516.83 | 7.75 | 0.000064 |
| LOC360997 | similar to ATP-binding cassette, sub-family G (WHITE), member 3 | 266.43 | 567.48 | 2.13 | 0.000841 |
|  | Rn.46981.1 | 706.09 | 334.32 | -2.11 | 0.000040 |
| Stx11 | syntaxin 11 | 13.12 | 58.16 | 4.43 | 0.005689 |
|  | Rn.58609.1 | 92.23 | 34.19 | -2.70 | 0.018304 |
| Slc43a2 | solute carrier family 43, member 2 | 223.72 | 430.56 | 1.92 | 0.000117 |
| Ifi44l | Interferon-induced protein 44-like | 60.88 | 195.67 | 3.21 | 0.002520 |
| Pex5l | peroxisomal biogenesis factor 5-like | 184.54 | 43.31 | -4.26 | 0.000456 |
| Stau2 | staufen, RNA binding protein, homolog 2 (Drosophila) | 826.78 | 336.85 | -2.45 | 0.000020 |
| Tnfaip8l2 | tumor necrosis factor, alpha-induced protein 8-like 2 | 46.99 | 468.06 | 9.96 | 0.002763 |
|  | Rn.8827.1 | 259.43 | 117.10 | -2.22 | 0.001902 |
|  | Rn.23792.1 | 515.42 | 234.11 | -2.20 | 0.000002 |
| Lmo4 | LIM domain only 4 | 85.89 | 212.15 | 2.47 | 0.011444 |
|  | Rn.12685.1 | 154.48 | 81.56 | -1.89 | 0.001511 |
|  | Rn.39201.1 | 96.01 | 288.20 | 3.00 | 0.000145 |
|  | Rn.39393.1 | 58.67 | 222.97 | 3.80 | 0.006240 |
|  | Rn.42802.1 | 32.75 | 194.91 | 5.95 | 0.007488 |
|  | Rn.19107.1 | 67.21 | 131.86 | 1.96 | 0.005244 |
|  | Rn.62300.1 | 162.04 | 68.86 | -2.35 | 0.002476 |
|  | Rn.59653.1 | 282.28 | 130.29 | -2.17 | 0.001515 |
| Shisa5 | shisa homolog 5 (Xenopus laevis) | 100.54 | 301.78 | 3.00 | 0.000135 |
| RGD1310110 | similar to 3632451O06Rik protein | 351.81 | 170.39 | -2.06 | 0.000222 |
|  | Rn.19293.1 | 1811.11 | 1049.55 | -1.73 | 0.000136 |
|  | Rn.16031.1 | 399.77 | 218.19 | -1.83 | 0.005225 |
| Ccl7 | chemokine (C-C motif) ligand 7 | 11.55 | 293.55 | 25.41 | 0.049355 |
|  | Rn.45230.1 | 170.17 | 69.55 | -2.45 | 0.000697 |
|  | Rn.46118.1 | 68.12 | 19.68 | -3.46 | 0.004658 |
| Slfn8 | schlafen 8 | 57.07 | 320.78 | 5.62 | 0.008481 |
| LOC305633 | similar to Antxr2 protein | 5.84 | 28.06 | 4.81 | 0.005512 |
|  | Rn.18499.1 | 209.43 | 99.88 | -2.10 | 0.000414 |
|  | Rn.34663.1 | 568.90 | 301.90 | -1.88 | 0.001193 |
| Tcirg1 | T-cell, immune regulator 1, ATPase, H+ transporting, lysosomal V0 subunit A3 | 199.72 | 861.84 | 4.32 | 0.000124 |
| Dennd2d | DENN/MADD domain containing 2D | 18.25 | 37.94 | 2.08 | 0.007383 |
|  | Rn.33111.1 | 452.50 | 252.43 | -1.79 | 0.000068 |
|  | Rn.21213.1 | 26.42 | 55.52 | 2.10 | 0.017019 |
| Pdp2 | pyruvate dehyrogenase phosphatase catalytic subunit 2 | 163.07 | 67.49 | -2.42 | 0.002881 |
| Herc3 | Hect domain and RLD 3 | 318.37 | 144.27 | -2.21 | 0.000366 |
| Ch25h | cholesterol 25-hydroxylase | 169.09 | 872.98 | 5.16 | 0.000514 |
| RGD1565926 | RGD1565926 | 19.78 | 82.26 | 4.16 | 0.003588 |
|  | Rn.12674.1 | 109.47 | 312.48 | 2.85 | 0.000021 |
|  | Rn.43019.1 | 13.03 | 267.48 | 20.53 | 0.012316 |
|  | Rn.19094.1 | 316.29 | 136.60 | -2.32 | 0.003259 |
| Nat8l | N-acetyltransferase 8-like | 1370.42 | 582.39 | -2.35 | 0.000039 |
| Jak2 | Janus kinase 2 | 319.78 | 777.06 | 2.43 | 0.000305 |
|  | Rn.18034.1 | 67.96 | 33.88 | -2.01 | 0.005511 |
|  | Rn.16568.1 | 336.41 | 179.41 | -1.88 | 0.001768 |
| Etv4 | ets variant 4 | 107.51 | 50.22 | -2.14 | 0.003588 |
| Kif5c | kinesin family member 5C | 1759.57 | 692.90 | -2.54 | 0.007695 |
| RGD1563437 | Similar to KIAA1217 | 868.00 | 398.22 | -2.18 | 0.000223 |
|  | Rn.46182.1 | 292.06 | 151.10 | -1.93 | 0.000779 |
| Ndc80 | NDC80 homolog, kinetochore complex component (S. cerevisiae) | 7.28 | 33.01 | 4.53 | 0.026631 |
| RGD1306880 | similar to hypothetical protein MGC47816 | 356.51 | 118.90 | -3.00 | 0.000289 |
| Maff | v-maf musculoaponeurotic fibrosarcoma oncogene homolog F (avian) | 41.64 | 257.30 | 6.18 | 0.003324 |
| RGD1304693 | similar to CG14803-PA | 15.25 | 42.37 | 2.78 | 0.010647 |
|  | Rn.43641.1 | 77.96 | 310.78 | 3.99 | 0.000345 |
|  | Rn.50212.1 | 891.58 | 311.17 | -2.87 | 0.004513 |
| Ttll11 | tubulin tyrosine ligase-like family, member 11 | 187.81 | 84.06 | -2.23 | 0.003801 |
|  | Rn.44160.1 | 61.08 | 233.92 | 3.83 | 0.000090 |
|  | Rn.63248.1 | 19.66 | 64.62 | 3.29 | 0.001550 |
| nod3l | NOD3-like protein | 142.07 | 74.66 | -1.90 | 0.001923 |
|  | Rn.24465.1 | 79.00 | 36.58 | -2.16 | 0.017029 |
|  | Rn.76384.1 | 543.65 | 262.84 | -2.07 | 0.000188 |
| Cdh10 | cadherin 10 | 669.71 | 346.83 | -1.93 | 0.003420 |
|  | Rn.59718.1 | 215.82 | 828.92 | 3.84 | 0.018211 |
|  | Rn.39318.1 | 280.05 | 150.32 | -1.86 | 0.000363 |
| Rbm47 | RNA binding motif protein 47 | 36.66 | 396.84 | 10.82 | 0.004257 |
| Irak3 | interleukin-1 receptor-associated kinase 3 | 79.58 | 231.33 | 2.91 | 0.000837 |
|  | Rn.47079.1 | 1539.02 | 692.13 | -2.22 | 0.000123 |
| Serpinb1a | serine (or cysteine) proteinase inhibitor, clade B, member 1a | 69.78 | 380.54 | 5.45 | 0.009948 |
| Btk | Bruton agammaglobulinemia tyrosine kinase | 34.92 | 239.24 | 6.85 | 0.000017 |
|  | Rn.26991.1 | 27.81 | 8.13 | -3.42 | 0.041225 |
|  | Rn.19821.1 | 123.89 | 58.46 | -2.12 | 0.009491 |
|  | Rn.60059.1 | 253.04 | 79.22 | -3.19 | 0.004297 |
|  | Rn.23216.1 | 212.63 | 900.06 | 4.23 | 0.000418 |
| Rnasel | Ribonuclease L (2',5'-oligoisoadenylate synthetase-dependent) | 197.00 | 394.07 | 2.00 | 0.000039 |
|  | Rn.33320.1 | 149.36 | 363.02 | 2.43 | 0.001414 |
|  | Rn.15974.1 | 52.27 | 191.57 | 3.66 | 0.011527 |
| Loxl2 | Lysyl oxidase-like 2 | 23.09 | 78.46 | 3.40 | 0.013203 |
| Dpep2 | dipeptidase 2 | 3.39 | 25.47 | 7.52 | 0.002268 |
| Hcls1 | hematopoietic cell specific Lyn substrate 1 | 111.79 | 384.12 | 3.44 | 0.000081 |
| Nckap1l | NCK associated protein 1 like | 112.53 | 781.42 | 6.94 | 0.000144 |
|  | Rn.18506.1 | 7.13 | 38.09 | 5.34 | 0.002755 |
|  | Rn.32510.1 | 143.41 | 75.34 | -1.90 | 0.001201 |
|  | Rn.43624.1 | 7.03 | 100.58 | 14.31 | 0.029857 |
| Rnf41 | ring finger protein 41 | 28.63 | 107.72 | 3.76 | 0.000489 |
|  | Rn.28546.1 | 258.42 | 132.84 | -1.95 | 0.000211 |
|  | Rn.79376.1 | 59.33 | 9.02 | -6.57 | 0.001255 |
| Csf1 | colony stimulating factor 1 (macrophage) | 87.67 | 32.18 | -2.72 | 0.016021 |
| St8sia4 | ST8 alpha-N-acetyl-neuraminide alpha-2,8-sialyltransferase 4 | 23.39 | 89.24 | 3.82 | 0.000111 |
| Fes | feline sarcoma oncogene | 64.58 | 397.46 | 6.15 | 0.000002 |
|  | Rn.51702.1 | 120.14 | 60.82 | -1.98 | 0.002540 |
|  | Rn.49823.1 | 47.89 | 8.07 | -5.93 | 0.008502 |
|  | Rn.58537.1 | 93.22 | 38.46 | -2.42 | 0.009379 |
|  | Rn.29413.1 | 10.57 | 25.16 | 2.38 | 0.013313 |
| Rnf180 | ring finger protein 180 | 267.18 | 133.46 | -2.00 | 0.000361 |
|  | Rn.50630.1 | 101.95 | 480.06 | 4.71 | 0.001946 |
|  | Rn.81764.1 | 205.98 | 71.88 | -2.87 | 0.012794 |
|  | Rn.42970.1 | 571.90 | 280.50 | -2.04 | 0.000181 |
|  | Rn.62808.1 | 115.82 | 46.05 | -2.51 | 0.009667 |
|  | Rn.53565.1 | 234.83 | 72.57 | -3.24 | 0.000950 |
|  | Rn.52525.1 | 18.19 | 89.90 | 4.94 | 0.023854 |
| Kif20b | kinesin family member 20B | 46.99 | 190.23 | 4.05 | 0.003643 |
| Gabra1 | gamma-aminobutyric acid (GABA) A receptor, alpha 1 | 722.15 | 243.97 | -2.96 | 0.000004 |
|  | Rn.26628.1 | 242.08 | 138.26 | -1.75 | 0.001006 |
| Ralyl | RALY RNA binding protein-like | 572.56 | 218.57 | -2.62 | 0.000012 |
|  | Rn.50122.1 | 104.59 | 49.03 | -2.13 | 0.005132 |
|  | Rn.28473.1 | 158.68 | 40.17 | -3.95 | 0.008415 |
|  | Rn.15299.1 | 109.36 | 33.16 | -3.30 | 0.005235 |
| LOC365985 | similar to adenylate kinase 5 isoform 1 | 793.95 | 323.30 | -2.46 | 0.000072 |
|  | Rn.58079.1 | 124.36 | 51.27 | -2.43 | 0.000129 |
|  | Rn.58553.1 | 65.54 | 24.59 | -2.67 | 0.008624 |
| LOC684841 | similar to CG31613-PA | 45.50 | 112.71 | 2.48 | 0.000399 |
| Syt16 | Synaptotagmin XVI | 124.69 | 65.69 | -1.90 | 0.002163 |
|  | Rn.44969.1 | 56.35 | 164.02 | 2.91 | 0.002734 |
|  | Rn.28522.1 | 122.42 | 61.30 | -2.00 | 0.011557 |
| Wnt7a | wingless-type MMTV integration site family, member 7A | 86.82 | 43.68 | -1.99 | 0.000895 |
|  | Rn.50176.1 | 135.81 | 42.99 | -3.16 | 0.000060 |
|  | Rn.57696.1 | 69.21 | 26.11 | -2.65 | 0.004311 |
|  | Rn.26610.1 | 410.88 | 149.70 | -2.74 | 0.000017 |
|  | Rn.24765.1 | 216.97 | 71.15 | -3.05 | 0.000037 |
| LOC691889 | similar to ATPase, aminophospholipid transporter-like, class I, type 8A, member 2 | 98.73 | 38.13 | -2.59 | 0.003161 |
|  | Rn.62107.1 | 101.72 | 46.27 | -2.20 | 0.007320 |
| Ifi44 | interferon-induced protein 44 | 273.70 | 704.76 | 2.57 | 0.000319 |
|  | Rn.50159.1 | 415.38 | 199.21 | -2.09 | 0.002161 |
| Tslp | thymic stromal lymphopoietin | 30.45 | 80.55 | 2.65 | 0.000447 |
|  | Rn.33700.1 | 211.80 | 78.25 | -2.71 | 0.000090 |
| Srgap2 | SLIT-ROBO Rho GTPase activating protein 2 | 83.40 | 172.33 | 2.07 | 0.003255 |
|  | Rn.41121.1 | 8.49 | 24.15 | 2.84 | 0.027054 |
|  | Rn.36638.1 | 695.76 | 377.70 | -1.84 | 0.000242 |
|  | Rn.56934.1 | 29.03 | 65.04 | 2.24 | 0.019549 |
|  | Rn.61754.1 | 23.95 | 6.17 | -3.88 | 0.019351 |
| Spdya | speedy homolog A (Xenopus laevis) | 61.20 | 28.98 | -2.11 | 0.012555 |
|  | Rn.12905.1 | 51.65 | 362.51 | 7.02 | 0.000052 |
|  | Rn.61241.1 | 127.47 | 49.46 | -2.58 | 0.001143 |
|  | Rn.33679.1 | 263.02 | 138.16 | -1.90 | 0.004125 |
| Plcxd2 | phosphatidylinositol-specific phospholipase C, X domain containing 2 | 969.52 | 504.70 | -1.92 | 0.000247 |
|  | Rn.15887.1 | 25.47 | 80.84 | 3.17 | 0.028567 |
|  | Rn.20328.1 | 135.16 | 545.83 | 4.04 | 0.000013 |
|  | Rn.21708.1 | 116.34 | 59.19 | -1.97 | 0.001416 |
|  | Rn.44544.1 | 609.49 | 343.31 | -1.78 | 0.001213 |
|  | Rn.49092.1 | 68.30 | 34.02 | -2.01 | 0.008334 |
| Ripk2 | receptor-interacting serine-threonine kinase 2 | 157.92 | 310.60 | 1.97 | 0.004082 |
|  | Rn.33936.1 | 470.73 | 198.86 | -2.37 | 0.009311 |
|  | Rn.23680.1 | 22.94 | 69.72 | 3.04 | 0.000887 |
| Emr1 | EGF-like module containing, mucin-like, hormone receptor-like 1 | 152.38 | 2270.59 | 14.90 | 0.000140 |
|  | Rn.49629.1 | 201.36 | 108.48 | -1.86 | 0.000608 |
|  | Rn.38807.1 | 208.65 | 107.24 | -1.95 | 0.013299 |
|  | Rn.59109.1 | 550.76 | 156.78 | -3.51 | 0.000231 |
|  | Rn.58023.1 | 136.04 | 47.78 | -2.85 | 0.010939 |
|  | Rn.49436.1 | 121.31 | 247.42 | 2.04 | 0.000585 |
| RGD1566130 | similar to mKIAA1940 protein | 281.67 | 109.24 | -2.58 | 0.000284 |
| Ank1 | ankyrin 1, erythrocytic | 1248.66 | 516.53 | -2.42 | 0.000011 |
|  | Rn.56187.1 | 78.23 | 35.69 | -2.19 | 0.007291 |
| Capzb | Capping protein (actin filament) muscle Z-line, beta | 36.02 | 74.20 | 2.06 | 0.031213 |
|  | Rn.26583.1 | 39.65 | 15.40 | -2.57 | 0.005204 |
| Rnd1 | Rho family GTPase 1 | 271.37 | 146.86 | -1.85 | 0.015324 |
|  | Rn.57525.1 | 74.96 | 23.27 | -3.22 | 0.003641 |
|  | Rn.68350.1 | 83.29 | 28.35 | -2.94 | 0.004350 |
| Ddx60 | DEAD (Asp-Glu-Ala-Asp) box polypeptide 60 | 31.42 | 360.49 | 11.47 | 0.000193 |
| Gna14 | guanine nucleotide binding protein, alpha 14 | 109.88 | 51.74 | -2.12 | 0.001903 |
| Nfkbid | nuclear factor of kappa light polypeptide gene enhancer in B-cells inhibitor, delta | 16.04 | 44.29 | 2.76 | 0.001929 |
|  | Rn.45525.1 | 390.25 | 204.96 | -1.90 | 0.000149 |
|  | Rn.57701.1 | 205.14 | 78.15 | -2.62 | 0.011385 |
|  | Rn.62959.1 | 398.06 | 153.79 | -2.59 | 0.004736 |
| Usp13 | ubiquitin specific protease 13 (isopeptidase T-3) | 525.65 | 262.67 | -2.00 | 0.010468 |
|  | Rn.1973.1 | 1007.34 | 401.41 | -2.51 | 0.000089 |
|  | Rn.60024.1 | 340.08 | 136.28 | -2.50 | 0.005919 |
|  | Rn.43173.1 | 43.87 | 19.49 | -2.25 | 0.009343 |
| Snph | syntaphilin | 634.87 | 333.78 | -1.90 | 0.000030 |
| RGD1563222 | similar to RIKEN cDNA A930018P22 | 23.04 | 87.26 | 3.79 | 0.003232 |
|  | Rn.59325.1 | 374.86 | 183.19 | -2.05 | 0.003440 |
| Nrxn3 | neurexin 3 | 30.55 | 11.93 | -2.56 | 0.017923 |
| Elavl2 | ELAV (embryonic lethal, abnormal vision, Drosophila)-like 2 (Hu antigen B) | 179.93 | 86.08 | -2.09 | 0.008788 |
|  | Rn.46984.1 | 1699.25 | 759.68 | -2.24 | 0.000825 |
|  | Rn.47588.1 | 327.96 | 154.72 | -2.12 | 0.001983 |
|  | Rn.47766.1 | 141.24 | 45.97 | -3.07 | 0.005096 |
|  | Rn.57633.1 | 23.32 | 7.40 | -3.15 | 0.021843 |
| RGD1306636 | hypothetical LOC293498 | 57.28 | 18.35 | -3.12 | 0.007338 |
|  | Rn.58112.1 | 820.45 | 436.12 | -1.88 | 0.007083 |
| Slc25a39 | Solute carrier family 25, member 39 | 12.76 | 46.19 | 3.62 | 0.010996 |
|  | Rn.46818.1 | 92.42 | 263.38 | 2.85 | 0.021401 |
|  | Rn.38021.1 | 221.17 | 84.63 | -2.61 | 0.000015 |
|  | Rn.21147.1 | 67.61 | 609.33 | 9.01 | 0.004375 |
| Cdca2 | cell division cycle associated 2 | 14.07 | 35.35 | 2.51 | 0.016294 |
|  | Rn.48117.1 | 18.77 | 48.96 | 2.61 | 0.012538 |
| Nmi | N-myc (and STAT) interactor | 60.66 | 189.08 | 3.12 | 0.001486 |
| Slc5a11 | solute carrier family 5 (sodium/glucose cotransporter), member 11 | 953.63 | 309.82 | -3.08 | 0.000047 |
| RGD1561507 | similar to hypothetical protein FLJ31606 | 256.63 | 147.57 | -1.74 | 0.002199 |
| Hic1 | hypermethylated in cancer 1 | 52.55 | 123.72 | 2.35 | 0.002381 |
|  | Rn.24975.1 | 762.98 | 1339.58 | 1.76 | 0.000613 |
| Sumf2 | sulfatase modifying factor 2 | 136.97 | 277.54 | 2.03 | 0.000461 |
| Sumf2 | sulfatase modifying factor 2 | 121.79 | 274.35 | 2.25 | 0.000090 |
| Uap1l1 | UDP-N-acteylglucosamine pyrophosphorylase 1-like 1 | 251.38 | 446.95 | 1.78 | 0.000431 |
|  | Rn.24648.1 | 611.05 | 1183.96 | 1.94 | 0.000325 |
| LOC685707 | Similar to neuron navigator 1 | 319.29 | 547.11 | 1.71 | 0.001063 |
| Rnls | renalase, FAD-dependent amine oxidase | 140.35 | 387.57 | 2.76 | 0.002014 |
| Arhgap9 | Rho GTPase activating protein 9 | 46.07 | 277.32 | 6.02 | 0.000443 |
| Unc93b1 | unc-93 homolog B1 (C. elegans) | 145.38 | 1342.14 | 9.23 | 0.000179 |
|  | Rn.24009.1 | 1089.75 | 350.98 | -3.10 | 0.000012 |
| Rras2 | related RAS viral (r-ras) oncogene homolog 2 | 219.03 | 637.81 | 2.91 | 0.000047 |
|  | Rn.19507.1 | 39.45 | 534.90 | 13.56 | 0.000119 |
| Scara3 | scavenger receptor class A, member 3 | 194.81 | 523.71 | 2.69 | 0.000057 |
| Ryr2 | ryanodine receptor 2, cardiac | 1012.58 | 384.16 | -2.64 | 0.000016 |
|  | Rn.21317.1 | 68.24 | 24.08 | -2.83 | 0.004609 |
| Ptprj | protein tyrosine phosphatase, receptor type, J | 366.51 | 1049.41 | 2.86 | 0.000001 |
| Ccl6 | chemokine (C-C motif) ligand 6 | 5.35 | 35.28 | 6.60 | 0.046912 |
|  | Rn.15077.1 | 228.67 | 2039.93 | 8.92 | 0.000090 |
| LOC100363005 | rCG32052-like | 2731.75 | 1269.07 | -2.15 | 0.009337 |
|  | Rn.7585.1 | 110.23 | 50.09 | -2.20 | 0.002599 |
|  | Rn.19824.1 | 896.60 | 440.66 | -2.03 | 0.000038 |
| Slc2a9 | solute carrier family 2 (facilitated glucose transporter), member 9 | 16.51 | 50.35 | 3.05 | 0.011342 |
| Nrarp | Notch-regulated ankyrin repeat protein | 126.78 | 63.50 | -2.00 | 0.011122 |
|  | Rn.3724.1 | 22.11 | 203.51 | 9.20 | 0.006151 |
| Clecsf6 | C-type (calcium dependent, carbohydrate recognition domain) lectin, superfamily member 6 | 39.92 | 360.38 | 9.03 | 0.001373 |
| Pion | pigeon homolog (Drosophila) | 34.44 | 286.21 | 8.31 | 0.002078 |
| Hoxc9 | homeobox C9 | 369.13 | 186.39 | -1.98 | 0.002383 |
| Lyve1 | lymphatic vessel endothelial hyaluronan receptor 1 | 209.20 | 590.40 | 2.82 | 0.006228 |
| F8 | Coagulation factor VIII, procoagulant component | 221.11 | 417.14 | 1.89 | 0.005120 |
|  | Rn.23411.1 | 83.39 | 37.65 | -2.21 | 0.004297 |
| Nhlrc2 | NHL repeat containing 2 | 41.37 | 75.06 | 1.81 | 0.001437 |
| RGD1305807 | hypothetical LOC298077 | 6.21 | 81.66 | 13.14 | 0.012059 |
|  | Rn.25185.1 | 540.24 | 275.60 | -1.96 | 0.000521 |
| Klhdc8a | kelch domain containing 8A | 133.04 | 68.43 | -1.94 | 0.002991 |
|  | Rn.53869.1 | 104.19 | 34.91 | -2.98 | 0.002051 |
| Bcl2l14 | Bcl2-like 14 (apoptosis facilitator) | 9.84 | 26.19 | 2.66 | 0.019765 |
| Rarres1 | retinoic acid receptor responder (tazarotene induced) 1 | 609.93 | 2125.71 | 3.49 | 0.000311 |
|  | Rn.13529.1 | 123.08 | 753.87 | 6.13 | 0.000375 |
|  | Rn.53568.1 | 270.87 | 134.59 | -2.01 | 0.000878 |
| Tifa | TRAF-interacting protein with forkhead-associated domain | 322.10 | 890.13 | 2.76 | 0.000034 |
| Isg15 | ISG15 ubiquitin-like modifier | 207.80 | 547.95 | 2.64 | 0.002798 |
| Stap1 | signal transducing adaptor family member 1 | 6.62 | 79.19 | 11.96 | 0.004210 |
|  | Rn.13650.1 | 64.58 | 330.32 | 5.11 | 0.008676 |
| Dram2 | DNA-damage regulated autophagy modulator 2 | 174.88 | 462.74 | 2.65 | 0.000151 |
|  | Rn.3052.1 | 116.12 | 278.64 | 2.40 | 0.002961 |
| Tasp1 | taspase, threonine aspartase 1 | 311.31 | 173.67 | -1.79 | 0.000662 |
|  | Rn.3724.2 | 370.23 | 1613.52 | 4.36 | 0.005443 |
|  | Rn.43466.1 | 732.46 | 399.23 | -1.83 | 0.000128 |
| sep-06 | septin 6 | 21.05 | 94.76 | 4.50 | 0.000066 |
|  | Rn.8243.1 | 239.41 | 95.02 | -2.52 | 0.014494 |
| Hebp2 | heme binding protein 2 | 712.68 | 231.07 | -3.08 | 0.021818 |
| Cxcl9 | chemokine (C-X-C motif) ligand 9 | 7.90 | 62.28 | 7.88 | 0.024321 |
|  | Rn.21519.1 | 231.11 | 93.74 | -2.47 | 0.000706 |
| Col13a1 | collagen, type XIII, alpha 1 | 74.44 | 284.13 | 3.82 | 0.004343 |
|  | Rn.20948.1 | 87.97 | 42.04 | -2.09 | 0.001094 |
| Cenpf | centromere protein F | 9.96 | 249.03 | 25.00 | 0.002765 |
| Gdap1l1 | Ganglioside-induced differentiation-associated protein 1-like 1 | 228.98 | 128.32 | -1.78 | 0.000814 |
| Trem2 | triggering receptor expressed on myeloid cells 2 | 40.24 | 322.91 | 8.02 | 0.000065 |
| Lypd6b | LY6/PLAUR domain containing 6B | 110.98 | 45.37 | -2.45 | 0.000625 |
| Plcxd2 | phosphatidylinositol-specific phospholipase C, X domain containing 2 | 344.23 | 190.37 | -1.81 | 0.005321 |
|  | Rn.18578.1 | 72.02 | 34.90 | -2.06 | 0.002390 |
| Phf11 | PHD finger protein 11 | 42.85 | 126.03 | 2.94 | 0.005686 |
| Il7r | interleukin 7 receptor | 46.05 | 100.82 | 2.19 | 0.016530 |
| Paqr9 | Progestin and adipoQ receptor family member IX | 103.91 | 49.76 | -2.09 | 0.002006 |
| Tox3 | TOX high mobility group box family member 3 | 166.26 | 76.83 | -2.16 | 0.000325 |
| Ankrd55 | ankyrin repeat domain 55 | 62.82 | 25.73 | -2.44 | 0.014853 |
|  | Rn.25444.1 | 33.68 | 759.86 | 22.56 | 0.005194 |
| Dcaf12l1 | DDB1 and CUL4 associated factor 12-like 1 | 586.66 | 274.28 | -2.14 | 0.000024 |
| Cst7 | cystatin F (leukocystatin) | 12.22 | 208.07 | 17.02 | 0.010998 |
|  | Rn.13150.1 | 1057.93 | 553.42 | -1.91 | 0.000224 |
|  | Rn.11834.1 | 515.37 | 286.79 | -1.80 | 0.000236 |
| Batf | basic leucine zipper transcription factor, ATF-like | 29.40 | 178.85 | 6.08 | 0.000157 |
| Cfhl1 | complement component factor h-like 1 | 15.38 | 37.39 | 2.43 | 0.007779 |
| Slit2 | slit homolog 2 (Drosophila) | 346.33 | 158.27 | -2.19 | 0.000353 |
| Clec7a | C-type lectin domain family 7, member a | 58.38 | 1970.74 | 33.76 | 0.000075 |
| LOC100360483 | Down syndrome cell adhesion molecule-like | 109.14 | 51.20 | -2.13 | 0.006782 |
|  | Rn.35579.1 | 150.96 | 66.29 | -2.28 | 0.006648 |
| Rps6ka3 | ribosomal protein S6 kinase polypeptide 3 | 169.29 | 358.72 | 2.12 | 0.003817 |
| Fam168a | family with sequence similarity 168, member A | 474.38 | 155.04 | -3.06 | 0.027500 |
| LOC100362342 | rCG47764-like | 48.09 | 158.22 | 3.29 | 0.000415 |
| Iffo2 | Intermediate filament family orphan 2 | 136.42 | 298.16 | 2.19 | 0.020150 |
| Mboat1 | membrane bound O-acyltransferase domain containing 1 | 41.40 | 266.68 | 6.44 | 0.002281 |
|  | Rn.20545.1 | 214.67 | 30.10 | -7.13 | 0.000156 |
|  | Rn.21668.1 | 72.90 | 33.44 | -2.18 | 0.007527 |
| RGD1310429 | similar to Protein Njmu-R1 | 147.33 | 69.35 | -2.12 | 0.003490 |
| Ppfia3 | Protein tyrosine phosphatase, receptor type, f polypeptide (PTPRF), interacting protein (liprin), alpha 3 | 143.04 | 69.70 | -2.05 | 0.001205 |
| Sgsm1 | small G protein signaling modulator 1 | 393.56 | 192.89 | -2.04 | 0.000132 |
| LOC100359879 | RIKEN cDNA A830041P22-like | 152.69 | 65.31 | -2.34 | 0.001029 |
|  | Rn.17927.1 | 14.39 | 325.46 | 22.62 | 0.010525 |
|  | Rn.52051.1 | 31.00 | 13.44 | -2.31 | 0.023982 |
| Cttnbp2nl | CTTNBP2 N-terminal like | 48.35 | 142.15 | 2.94 | 0.001163 |
| Npl | N-acetylneuraminate pyruvate lyase | 118.28 | 811.41 | 6.86 | 0.000044 |
| LOC311026 | similar to mKIAA1461 protein | 177.18 | 95.00 | -1.87 | 0.000693 |
|  | Rn.66436.1 | 786.14 | 284.62 | -2.76 | 0.005134 |
| Herc6 | hect domain and RLD 6 | 114.00 | 279.47 | 2.45 | 0.000048 |
| LOC310721 | similar to 4930431B09Rik protein | 66.74 | 263.89 | 3.95 | 0.001210 |
| Epha4 | Eph receptor A4 | 142.19 | 42.67 | -3.33 | 0.011466 |
|  | Rn.15266.1 | 685.20 | 308.93 | -2.22 | 0.000059 |
| Pank1 | pantothenate kinase 1 | 208.11 | 103.44 | -2.01 | 0.000200 |
|  | Rn.17891.1 | 8.20 | 20.64 | 2.52 | 0.020109 |
|  | Rn.17891.1 | 11.46 | 115.52 | 10.08 | 0.000220 |
|  | Rn.56336.1 | 732.51 | 299.57 | -2.45 | 0.001246 |
|  | Rn.15220.1 | 165.96 | 1018.81 | 6.14 | 0.003527 |
| Gpr126 | G protein-coupled receptor 126 | 137.10 | 240.98 | 1.76 | 0.000272 |
| Gpr64 | G protein-coupled receptor 64 | 222.89 | 122.17 | -1.82 | 0.000610 |
| Ahi1 | Abelson helper integration site 1 | 204.68 | 64.59 | -3.17 | 0.004021 |
| Tbc1d10c | TBC1 domain family, member 10C | 11.40 | 39.46 | 3.46 | 0.017561 |
| Galnt13 | UDP-N-acetyl-alpha-D-galactosamine:polypeptide N-acetylgalactosaminyltransferase 13 (GalNAc-T13) | 62.07 | 32.52 | -1.91 | 0.001519 |
|  | Rn.13172.1 | 3800.95 | 1803.39 | -2.11 | 0.000240 |
| Man2a1 | mannosidase, alpha, class 2A, member 1 | 47.98 | 141.53 | 2.95 | 0.009692 |
|  | Rn.18376.1 | 1502.96 | 814.83 | -1.84 | 0.000010 |
| Prcc | papillary renal cell carcinoma (translocation-associated) | 257.25 | 502.61 | 1.95 | 0.013225 |
| Ogfrl1 | opioid growth factor receptor-like 1 | 192.31 | 585.98 | 3.05 | 0.000001 |
| A2bp1 | ataxin 2 binding protein 1 | 1706.74 | 640.68 | -2.66 | 0.001848 |
| Itgb2 | integrin, beta 2 | 383.78 | 1628.87 | 4.24 | 0.001368 |
| Sox4 | SRY (sex determining region Y)-box 4 | 604.58 | 1302.66 | 2.15 | 0.018412 |
|  | Rn.24782.1 | 34.44 | 15.52 | -2.22 | 0.027133 |
| Igj | immunoglobulin joining chain | 13.08 | 60.62 | 4.63 | 0.038881 |
|  | Rn.12545.1 | 1878.77 | 845.83 | -2.22 | 0.000036 |
|  | Rn.24916.1 | 56.38 | 235.59 | 4.18 | 0.009254 |
| Atmin | ATM interactor | 810.85 | 480.78 | -1.69 | 0.000045 |
|  | Rn.22955.1 | 599.13 | 189.96 | -3.15 | 0.000086 |
|  | Rn.44010.1 | 445.69 | 252.74 | -1.76 | 0.000699 |
| LOC688535 | Hypothetical protein LOC688535 | 29.41 | 6.01 | -4.90 | 0.008575 |
|  | Rn.37608.1 | 8.96 | 73.77 | 8.24 | 0.001213 |
|  | Rn.37608.1 | 9.74 | 49.83 | 5.12 | 0.031019 |
| Fmo5 | flavin containing monooxygenase 5 | 37.08 | 79.26 | 2.14 | 0.000951 |
|  | Rn.25081.1 | 3056.63 | 1680.09 | -1.82 | 0.000091 |
| Sars2 | seryl-tRNA synthetase 2, mitochondrial | 98.60 | 29.82 | -3.31 | 0.001205 |
|  | Rn.17214.1 | 434.04 | 785.96 | 1.81 | 0.000097 |
|  | Rn.24242.1 | 695.92 | 282.56 | -2.46 | 0.000017 |
| A2bp1 | ataxin 2 binding protein 1 | 1615.32 | 801.84 | -2.01 | 0.000800 |
|  | Rn.25086.1 | 746.67 | 349.56 | -2.14 | 0.000023 |
|  | Rn.15644.1 | 296.38 | 99.74 | -2.97 | 0.001380 |
| RGD1310571 | similar to hypothetical protein | 390.19 | 221.76 | -1.76 | 0.000135 |
| C7 | complement component 7 /// tubulin, beta 2c | 28.98 | 107.25 | 3.70 | 0.015032 |
|  | Rn.19057.1 | 577.70 | 231.31 | -2.50 | 0.005296 |
| Pdyn | prodynorphin | 279.52 | 499.80 | 1.79 | 0.001299 |
|  | Rn.16134.1 | 128.48 | 335.74 | 2.61 | 0.001491 |
|  | Rn.50972.1 | 133.12 | 69.97 | -1.90 | 0.018932 |
| Tsku | tsukushin | 35.48 | 82.54 | 2.33 | 0.001892 |
|  | Rn.29866.1 | 460.58 | 207.43 | -2.22 | 0.003543 |
| Dsn1 | DSN1, MIND kinetochore complex component, homolog (S. cerevisiae) | 10.58 | 52.32 | 4.94 | 0.001287 |
| Atcay | ataxia, cerebellar, Cayman type | 385.83 | 155.91 | -2.47 | 0.000376 |
|  | Rn.21415.1 | 289.58 | 119.16 | -2.43 | 0.000369 |
| Abca1 | ATP-binding cassette, sub-family A (ABC1), member 1 | 80.40 | 1144.28 | 14.23 | 0.000984 |
|  | Rn.14987.2 | 317.28 | 127.81 | -2.48 | 0.000070 |
| Lnx2 | ligand of numb-protein X 2 | 132.17 | 220.06 | 1.66 | 0.000206 |
|  | Rn.30219.1 | 107.78 | 203.30 | 1.89 | 0.005014 |
|  | Rn.11709.1 | 80.55 | 35.25 | -2.28 | 0.000745 |
| Samd14 | sterile alpha motif domain containing 14 | 115.54 | 54.06 | -2.14 | 0.000696 |
| Ptafr | platelet-activating factor receptor | 382.33 | 1423.68 | 3.72 | 0.000000 |
| RGD1560394 | RGD1560394 | 305.23 | 164.00 | -1.86 | 0.007718 |
| Fam123a | family with sequence similarity 123A | 472.23 | 217.72 | -2.17 | 0.006774 |
| C2 | complement component 2 | 140.41 | 1872.02 | 13.33 | 0.001978 |
| C2 | complement component 2 | 84.20 | 1002.48 | 11.91 | 0.001246 |
| Sv2a | synaptic vesicle glycoprotein 2a | 1553.26 | 894.15 | -1.74 | 0.000035 |
| Tes | testis derived transcript | 304.63 | 780.69 | 2.56 | 0.000181 |
| Sash3 | SAM and SH3 domain containing 3 | 89.32 | 179.16 | 2.01 | 0.002153 |
| Hhatl | hedgehog acyltransferase-like | 437.87 | 157.75 | -2.78 | 0.000347 |
|  | Rn.24407.1 | 81.90 | 171.52 | 2.09 | 0.007411 |
| Npas2 | neuronal PAS domain protein 2 | 284.02 | 140.13 | -2.03 | 0.000173 |
| Irf9 | interferon regulatory factor 9 | 154.53 | 422.49 | 2.73 | 0.002025 |
|  | Rn.24654.1 | 279.70 | 154.90 | -1.81 | 0.001411 |
| Irak2 | interleukin-1 receptor-associated kinase 2 | 111.22 | 239.62 | 2.15 | 0.002914 |
| Rab9b | RAB9B, member RAS oncogene family | 1433.65 | 614.90 | -2.33 | 0.000031 |
| Il6st | interleukin 6 signal transducer | 34.63 | 84.98 | 2.45 | 0.003438 |
| Bcl11a | B-cell CLL/lymphoma 11A (zinc finger protein) | 207.12 | 91.27 | -2.27 | 0.001571 |
|  | Rn.249.1 | 456.49 | 203.15 | -2.25 | 0.000173 |
|  | Rn.24826.1 | 2729.46 | 1127.93 | -2.42 | 0.000006 |
| Ube2k | ubiquitin-conjugating enzyme E2K (UBC1 homolog, yeast) | 173.68 | 97.68 | -1.78 | 0.000606 |
| Nrip3 | nuclear receptor interacting protein 3 | 500.43 | 270.07 | -1.85 | 0.000247 |
|  | Rn.20948.2 | 220.00 | 118.27 | -1.86 | 0.000319 |
| Dhfr | dihydrofolate reductase | 35.29 | 104.14 | 2.95 | 0.008459 |
|  | Rn.32156.1 | 279.95 | 104.05 | -2.69 | 0.001629 |
|  | Rn.21974.1 | 632.44 | 251.18 | -2.52 | 0.001606 |
| Ddx19a | DEAD (Asp-Glu-Ala-Asp) box polypeptide 19a /// DEAD (Asp-Glu-Ala-As) box polypeptide 19B | 88.43 | 191.33 | 2.16 | 0.000220 |
| Cldn18 | claudin 18 | 10.36 | 28.43 | 2.74 | 0.012821 |
|  | Rn.35484.1 | 844.58 | 313.15 | -2.70 | 0.000046 |
| Man1a1 | mannosidase, alpha, class 1A, member 1 | 99.28 | 309.95 | 3.12 | 0.000164 |
| Rad51 | RAD51 homolog (RecA homolog, E. coli) (S. cerevisiae) | 86.42 | 192.40 | 2.23 | 0.001771 |
| LOC680531 | similar to CG3880-PA | 23.30 | 93.49 | 4.01 | 0.005699 |
| Isy1 | ISY1 splicing factor homolog (S. cerevisiae) | 57.98 | 118.85 | 2.05 | 0.001775 |
| Bclaf1 | BCL2-associated transcription factor 1 | 107.93 | 210.23 | 1.95 | 0.012243 |
|  | Rn.24481.1 | 145.73 | 407.75 | 2.80 | 0.001380 |
|  | Rn.12772.1 | 581.34 | 332.01 | -1.75 | 0.000164 |
| Eya2 | Eyes absent homolog 2 (Drosophila) | 234.80 | 482.40 | 2.05 | 0.000610 |
|  | Rn.35743.1 | 2288.55 | 1071.69 | -2.14 | 0.000025 |
| Nuak2 | NUAK family, SNF1-like kinase, 2 | 91.31 | 217.66 | 2.38 | 0.000338 |
| Il10rb | interleukin 10 receptor, beta | 175.79 | 629.95 | 3.58 | 0.000936 |
| Dok3 | docking protein 3 | 23.64 | 163.08 | 6.90 | 0.000116 |
| Stt3b | STT3, subunit of the oligosaccharyltransferase complex, homolog B (S. cerevisiae) | 125.17 | 270.63 | 2.16 | 0.001465 |
|  | Rn.16087.1 | 306.21 | 150.72 | -2.03 | 0.000268 |
|  | Rn.7606.1 | 458.86 | 187.18 | -2.45 | 0.001148 |
| Tnip2 | TNFAIP3 interacting protein 2 | 155.89 | 319.68 | 2.05 | 0.000306 |
| Laptm5 | lysosomal protein transmembrane 5 | 374.08 | 1484.88 | 3.97 | 0.000844 |
| Nap1l2 | nucleosome assembly protein 1-like 2 | 1580.30 | 739.32 | -2.14 | 0.001081 |
|  | Rn.19587.1 | 101.89 | 291.17 | 2.86 | 0.000063 |
| Rnf208 | ring finger protein 208 | 541.00 | 236.17 | -2.29 | 0.000103 |
| Rit2 | Ras-like without CAAX 2 | 506.67 | 236.58 | -2.14 | 0.003146 |
| Map2k4 | Mitogen activated protein kinase kinase 4 | 177.24 | 90.17 | -1.97 | 0.013485 |
| Actr3b | ARP3 actin-related protein 3 homolog B (yeast) | 265.56 | 155.38 | -1.71 | 0.000574 |
| RGD1305733 | similar to RIKEN cDNA 2900011O08 | 1005.84 | 510.14 | -1.97 | 0.000003 |
| Gria4 | glutamate receptor, ionotropic, AMPA4 | 767.99 | 261.26 | -2.94 | 0.001078 |
|  | Rn.15062.1 | 85.34 | 154.77 | 1.81 | 0.003364 |
| Nmt1 | N-myristoyltransferase 1 | 12.91 | 38.39 | 2.97 | 0.009976 |
| Rgs7bp | regulator of G-protein signaling 7 binding protein | 656.33 | 173.82 | -3.78 | 0.001680 |
| Elavl2 | ELAV (embryonic lethal, abnormal vision, Drosophila)-like 2 (Hu antigen B) | 1632.39 | 699.01 | -2.34 | 0.000056 |
| Ect2 | epithelial cell transforming sequence 2 oncogene | 10.40 | 240.88 | 23.16 | 0.003377 |
|  | Rn.11467.1 | 584.37 | 248.36 | -2.35 | 0.000015 |
|  | Rn.20709.1 | 88.93 | 34.58 | -2.57 | 0.005796 |
| Elavl2 | ELAV (embryonic lethal, abnormal vision, Drosophila)-like 2 (Hu antigen B) | 1305.46 | 451.69 | -2.89 | 0.000609 |
|  | Rn.23863.1 | 159.65 | 327.34 | 2.05 | 0.003737 |
|  | Rn.16235.1 | 290.85 | 122.09 | -2.38 | 0.001398 |
| Lef1 | Lymphoid enhancer binding factor 1 | 143.97 | 300.26 | 2.09 | 0.026436 |
| Rab32 | RAB32, member RAS oncogene family | 126.51 | 837.93 | 6.62 | 0.017143 |
| LOC641520 | Popeye domain-containing 3 | 63.92 | 27.58 | -2.32 | 0.000278 |
|  | Rn.9516.1 | 387.04 | 213.44 | -1.81 | 0.000644 |
|  | Rn.23892.1 | 350.48 | 129.22 | -2.71 | 0.000931 |
| Zfyve1 | zinc finger, FYVE domain containing 1 | 4.22 | 21.44 | 5.08 | 0.003275 |
| Fam55b | family with sequence similarity 55, member B | 109.90 | 600.12 | 5.46 | 0.000039 |
|  | Rn.21999.2 | 109.70 | 230.87 | 2.10 | 0.000421 |
| Fosl2 | Fos-like antigen 2 | 7.48 | 21.15 | 2.83 | 0.020632 |
|  | Rn.6508.1 | 35.33 | 89.40 | 2.53 | 0.000881 |
| Lmo2 | LIM domain only 2 | 411.68 | 1213.74 | 2.95 | 0.003192 |
| Eif5a2 | eukaryotic translation initiation factor 5A2 | 1007.36 | 454.31 | -2.22 | 0.000005 |
|  | Rn.16708.1 | 27.81 | 88.69 | 3.19 | 0.003712 |
| Upk1b | uroplakin 1B | 110.98 | 212.10 | 1.91 | 0.002432 |
| RGD1306991 | similar to Protein C20orf103 precursor | 3621.44 | 1816.10 | -1.99 | 0.000008 |
|  | Rn.35760.1 | 29.86 | 204.03 | 6.83 | 0.005121 |
| Flywch1 | FLYWCH-type zinc finger 1 | 373.69 | 150.90 | -2.48 | 0.000915 |
|  | Rn.12534.1 | 369.28 | 649.11 | 1.76 | 0.002055 |
| Abi3 | ABI family, member 3 | 104.36 | 396.72 | 3.80 | 0.000007 |
|  | Rn.24298.1 | 114.59 | 32.01 | -3.58 | 0.000115 |
|  | Rn.12124.1 | 170.49 | 83.87 | -2.03 | 0.001637 |
| Nuf2 | NUF2, NDC80 kinetochore complex component, homolog (S. cerevisiae) | 51.90 | 268.16 | 5.17 | 0.000714 |
| Snx9 | sorting nexin 9 | 257.85 | 465.16 | 1.80 | 0.007021 |
|  | Rn.41190.1 | 541.64 | 218.88 | -2.47 | 0.000416 |
| Mical1 | microtubule associated monoxygenase, calponin and LIM domain containing 1 | 60.86 | 189.85 | 3.12 | 0.000686 |
| Cdca2 | cell division cycle associated 2 | 22.66 | 54.50 | 2.41 | 0.008372 |
|  | Rn.20081.1 | 1243.12 | 2511.53 | 2.02 | 0.000042 |
|  | Rn.36267.1 | 101.16 | 204.45 | 2.02 | 0.027011 |
| Syt3 | Synaptotagmin III | 299.04 | 132.86 | -2.25 | 0.002113 |
|  | Rn.20788.1 | 220.07 | 77.57 | -2.84 | 0.000074 |
| Sox4 | SRY (sex determining region Y)-box 4 | 113.38 | 265.21 | 2.34 | 0.008348 |
| Gpr158 | G protein-coupled receptor 158 | 788.85 | 435.65 | -1.81 | 0.000327 |
| Atl1 | atlastin GTPase 1 | 997.02 | 519.57 | -1.92 | 0.000010 |
| Lrrk1 | Leucine-rich repeat kinase 1 | 135.04 | 393.38 | 2.91 | 0.000025 |
|  | Rn.7560.1 | 871.16 | 286.22 | -3.04 | 0.000018 |
|  | Rn.24011.1 | 316.55 | 123.54 | -2.56 | 0.000495 |
| Cthrc1 | collagen triple helix repeat containing 1 | 28.80 | 269.01 | 9.34 | 0.028215 |
| Ckap2 | cytoskeleton associated protein 2 | 25.16 | 175.29 | 6.97 | 0.000213 |
| Gmip | Gem-interacting protein | 91.38 | 204.11 | 2.23 | 0.000218 |
|  | Rn.12513.1 | 9.10 | 40.81 | 4.49 | 0.000389 |
| Nt5e | 5' nucleotidase, ecto | 179.38 | 602.15 | 3.36 | 0.000846 |
|  | Rn.20769.1 | 26.12 | 8.89 | -2.94 | 0.001705 |
|  | Rn.48398.1 | 958.11 | 562.92 | -1.70 | 0.000036 |
|  | Rn.37608.2 | 8.41 | 62.50 | 7.43 | 0.000278 |
|  | Rn.37608.2 | 25.26 | 151.02 | 5.98 | 0.000032 |
| Kif1a | kinesin family member 1A | 1261.58 | 408.63 | -3.09 | 0.010881 |
|  | Rn.20117.1 | 369.44 | 145.60 | -2.54 | 0.012417 |
| Gja7 | gap junction membrane channel protein alpha 7 | 63.18 | 167.91 | 2.66 | 0.022086 |
| Rab20 | RAB20, member RAS oncogene family | 97.64 | 224.99 | 2.30 | 0.004032 |
| Jakmip2 | Janus kinase and microtubule interacting protein 2 | 514.28 | 258.61 | -1.99 | 0.001654 |
|  | Rn.20313.1 | 694.09 | 293.23 | -2.37 | 0.000021 |
|  | Rn.45040.1 | 368.03 | 781.94 | 2.12 | 0.013930 |
|  | Rn.18088.1 | 10.09 | 46.59 | 4.62 | 0.011206 |
|  | Rn.79618.1 | 126.53 | 233.69 | 1.85 | 0.001994 |
| Ifit2 | interferon-induced protein with tetratricopeptide repeats 2 | 310.01 | 717.27 | 2.31 | 0.000246 |
|  | Rn.24096.1 | 686.04 | 411.27 | -1.67 | 0.000094 |
|  | Rn.24703.1 | 87.89 | 35.46 | -2.48 | 0.001275 |
| Edem1 | ER degradation enhancer, mannosidase alpha-like 1 | 43.86 | 221.26 | 5.05 | 0.002228 |
| Dlgap1 | Discs, large (Drosophila) homolog-associated protein 1 | 544.61 | 271.58 | -2.01 | 0.000602 |
|  | Rn.16520.1 | 558.14 | 249.77 | -2.23 | 0.000214 |
|  | Rn.54031.1 | 221.05 | 103.67 | -2.13 | 0.000546 |
|  | Rn.20061.1 | 69.22 | 167.31 | 2.42 | 0.001551 |
| Gnal | Guanine nucleotide binding protein, alpha stimulating, olfactory type | 146.97 | 64.15 | -2.29 | 0.000131 |
| LOC687121 | similar to Shc SH2-domain binding protein 1 | 26.42 | 210.88 | 7.98 | 0.005740 |
|  | Rn.15616.1 | 566.14 | 239.76 | -2.36 | 0.000268 |
| LOC100362555 | androgen down regulated in mouse prostate | 226.25 | 58.86 | -3.84 | 0.001141 |
| Spsb4 | splA/ryanodine receptor domain and SOCS box containing 4 | 99.59 | 47.86 | -2.08 | 0.001853 |
|  | Rn.19734.1 | 1832.65 | 780.79 | -2.35 | 0.000078 |
| Nono | non-POU domain containing, octamer-binding | 75.57 | 163.99 | 2.17 | 0.004744 |
| Racgap1 | Rac GTPase-activating protein 1 | 20.83 | 56.76 | 2.73 | 0.002706 |
| Ppp1r3b | protein phosphatase 1, regulatory (inhibitor) subunit 3B | 49.20 | 111.66 | 2.27 | 0.029377 |
|  | Rn.16810.1 | 19.19 | 53.34 | 2.78 | 0.000681 |
|  | Rn.7074.1 | 201.68 | 66.66 | -3.03 | 0.000484 |
| Nusap1 | nucleolar and spindle associated protein 1 | 18.99 | 301.40 | 15.87 | 0.009738 |
|  | Rn.49589.1 | 182.64 | 79.08 | -2.31 | 0.000081 |
| Rell2 | RELT-like 2 | 1175.30 | 574.19 | -2.05 | 0.000011 |
|  | Rn.25219.2 | 104.98 | 53.71 | -1.95 | 0.003836 |
| Dok1 | docking protein 1 | 41.57 | 123.73 | 2.98 | 0.006129 |
|  | Rn.16262.1 | 33.77 | 270.80 | 8.02 | 0.000590 |
| Myo1f | myosin IF | 42.66 | 578.98 | 13.57 | 0.001748 |
|  | Rn.45890.1 | 316.86 | 151.71 | -2.09 | 0.000648 |
| Slc45a1 | solute carrier family 45, member 1 | 378.13 | 194.32 | -1.95 | 0.000131 |
|  | Rn.12451.1 | 305.90 | 142.92 | -2.14 | 0.000696 |
|  | Rn.24418.1 | 33.42 | 13.14 | -2.54 | 0.003548 |
| Cdc45l | CDC45 cell division cycle 45-like (S. cerevisiae) | 7.99 | 24.32 | 3.04 | 0.010004 |
|  | Rn.11905.1 | 894.59 | 362.70 | -2.47 | 0.000027 |
| Nckap1l | NCK associated protein 1 like | 143.20 | 611.00 | 4.27 | 0.000003 |
| Papd4 | PAP associated domain containing 4 | 112.35 | 251.47 | 2.24 | 0.000898 |
| Mthfs | 5,10-methenyltetrahydrofolate synthetase (5-formyltetrahydrofolate cyclo-ligase) | 6.57 | 24.66 | 3.75 | 0.011535 |
| Abca1 | ATP-binding cassette, sub-family A (ABC1), member 1 | 15.74 | 243.88 | 15.50 | 0.000880 |
| Sox7 | SRY (sex determining region Y)-box 7 | 33.28 | 88.40 | 2.66 | 0.001628 |
| Tmem181 | transmembrane protein 181 | 209.55 | 356.01 | 1.70 | 0.000872 |
| LOC100359879 | RIKEN cDNA A830041P22-like | 152.51 | 61.79 | -2.47 | 0.002055 |
|  | Rn.11851.1 | 68.50 | 189.14 | 2.76 | 0.000658 |
|  | Rn.34755.1 | 1103.03 | 602.64 | -1.83 | 0.000026 |
| Abca5 | ATP-binding cassette, sub-family A (ABC1), member 5 | 500.18 | 233.41 | -2.14 | 0.000740 |
|  | Rn.39323.1 | 348.72 | 155.46 | -2.24 | 0.000001 |
|  | Rn.41341.1 | 331.73 | 179.15 | -1.85 | 0.000158 |
|  | Rn.43963.1 | 86.76 | 192.34 | 2.22 | 0.002215 |
|  | Rn.53012.1 | 378.72 | 163.62 | -2.31 | 0.000232 |
| Fam105a | family with sequence similarity 105, member A | 94.40 | 1005.00 | 10.65 | 0.000859 |
| LOC100363441 | hypothetical protein LOC100363441 | 145.81 | 52.85 | -2.76 | 0.002304 |
| Hapln1 | hyaluronan and proteoglycan link protein 1 | 18.36 | 5.09 | -3.61 | 0.018905 |
| Pon3 | paraoxonase 3 | 130.56 | 238.46 | 1.83 | 0.002324 |
| LOC363326 | hypothetical LOC363326 | 59.42 | 122.63 | 2.06 | 0.001097 |
| Fam159b | family with sequence similarity 159, member B | 27.46 | 10.92 | -2.52 | 0.041782 |
|  | Rn.51677.1 | 476.57 | 132.77 | -3.59 | 0.001956 |
| Pear1 | platelet endothelial aggregation receptor 1 | 32.64 | 81.36 | 2.49 | 0.035345 |
|  | Rn.36267.2 | 347.51 | 959.96 | 2.76 | 0.023819 |
| Fam198b | family with sequence similarity 198, member B | 28.46 | 150.30 | 5.28 | 0.000437 |
|  | Rn.25319.1 | 122.20 | 59.79 | -2.04 | 0.003488 |
|  | Rn.34305.1 | 347.62 | 140.03 | -2.48 | 0.008507 |
|  | Rn.23644.1 | 111.87 | 46.42 | -2.41 | 0.001245 |
|  | Rn.47453.1 | 12.65 | 53.76 | 4.25 | 0.008502 |
| Apeg3 | antisense paternally expressed gene 3 | 211.29 | 95.33 | -2.22 | 0.003383 |
|  | Rn.45881.1 | 668.63 | 251.89 | -2.65 | 0.000004 |
|  | Rn.52159.1 | 143.88 | 43.46 | -3.31 | 0.000027 |
|  | Rn.24343.1 | 699.33 | 416.24 | -1.68 | 0.000197 |
| Scara5 | scavenger receptor class A, member 5 (putative) | 236.56 | 591.70 | 2.50 | 0.003231 |
| Pla2g3 | phospholipase A2, group III | 620.46 | 164.41 | -3.77 | 0.000846 |
| Atrx | Alpha thalassemia/mental retardation syndrome X-linked (RAD54 homolog, S. cerevisiae) | 71.66 | 142.01 | 1.98 | 0.000966 |
|  | Rn.51543.1 | 533.49 | 231.62 | -2.30 | 0.000519 |
|  | Rn.50536.1 | 14.35 | 55.11 | 3.84 | 0.000955 |
| Rhbdf2 | rhomboid 5 homolog 2 (Drosophila) | 39.27 | 142.24 | 3.62 | 0.000164 |
| Tmem132e | transmembrane protein 132E | 169.41 | 84.13 | -2.01 | 0.000424 |
| Cpne4 | copine IV | 135.41 | 46.02 | -2.94 | 0.007808 |
|  | Rn.27650.1 | 101.28 | 50.82 | -1.99 | 0.005431 |
| Cxadr | coxsackie virus and adenovirus receptor | 9.15 | 62.68 | 6.85 | 0.011197 |
|  | Rn.45231.1 | 608.34 | 339.04 | -1.79 | 0.000248 |
| Polm | polymerase (DNA directed), mu | 94.04 | 178.87 | 1.90 | 0.001346 |
| Cd69 | Cd69 molecule | 5.71 | 56.27 | 9.85 | 0.008057 |
|  | Rn.45487.1 | 682.79 | 336.83 | -2.03 | 0.000332 |
|  | Rn.63919.1 | 16.22 | 130.37 | 8.04 | 0.001672 |
| Fas | Fas (TNF receptor superfamily, member 6) | 46.72 | 125.50 | 2.69 | 0.000784 |
| Rab27a | RAB27A, member RAS oncogene family | 32.00 | 64.87 | 2.03 | 0.002176 |
|  | Rn.47252.1 | 151.38 | 61.40 | -2.47 | 0.000428 |
| Entpd3 | ectonucleoside triphosphate diphosphohydrolase 3 | 202.02 | 87.22 | -2.32 | 0.000026 |
| Ephx4 | epoxide hydrolase 4 | 97.13 | 29.09 | -3.34 | 0.001747 |
|  | Rn.47087.1 | 121.95 | 48.81 | -2.50 | 0.000992 |
| Aqp11 | aquaporin 11 | 251.19 | 111.95 | -2.24 | 0.000150 |
| Trpt1 | tRNA phosphotransferase 1 | 113.87 | 51.10 | -2.23 | 0.011226 |
|  | Rn.34831.1 | 106.10 | 262.66 | 2.48 | 0.000141 |
|  | Rn.33281.1 | 69.58 | 219.75 | 3.16 | 0.002089 |
| Ctxn2 | cortexin 2 | 383.46 | 161.89 | -2.37 | 0.000108 |
|  | Rn.16085.1 | 474.03 | 265.02 | -1.79 | 0.000195 |
| Apob48r | apolipoprotein B48 receptor | 32.80 | 64.49 | 1.97 | 0.002975 |
| Lrfn5 | leucine rich repeat and fibronectin type III domain containing 5 | 308.16 | 102.81 | -3.00 | 0.000009 |
| Tlr1 | toll-like receptor 1 | 32.60 | 187.56 | 5.75 | 0.010590 |
|  | Rn.79774.1 | 204.51 | 111.48 | -1.83 | 0.006774 |
| Sh3bp2 | SH3-domain binding protein 2 | 63.08 | 209.26 | 3.32 | 0.000208 |
| Cd6 | Cd6 molecule | 34.33 | 88.48 | 2.58 | 0.001966 |
| Fbxo5 | F-box protein 5 | 102.80 | 204.94 | 1.99 | 0.024912 |
| RGD1309847 | similar to peptidylglycine alpha-amidating monooxygenase COOH-terminal interactor; peptidylglycine alpha-amidating monooxygenase COOH-terminal interactor protein-1 | 42.56 | 138.92 | 3.26 | 0.006124 |
| Jrk | jerky homolog (mouse) | 13.98 | 43.29 | 3.10 | 0.007249 |
|  | Rn.59506.1 | 164.78 | 85.75 | -1.92 | 0.001141 |
| Gsdmd | gasdermin D | 34.10 | 148.84 | 4.36 | 0.002804 |
|  | Rn.15509.1 | 1018.46 | 480.46 | -2.12 | 0.010341 |
| Nudt11 | Nudix (nucleoside diphosphate linked moiety X)-type motif 11 | 471.34 | 254.70 | -1.85 | 0.004909 |
|  | Rn.27256.2 | 553.41 | 215.46 | -2.57 | 0.000258 |
|  | Rn.51942.1 | 68.66 | 211.13 | 3.07 | 0.010247 |
|  | Rn.42004.1 | 329.99 | 165.06 | -2.00 | 0.000322 |
| Hhip | Hedgehog-interacting protein | 306.31 | 64.23 | -4.77 | 0.010322 |
|  | Rn.32617.2 | 503.74 | 192.06 | -2.62 | 0.000001 |
| Csrnp3 | cysteine-serine-rich nuclear protein 3 | 370.70 | 147.09 | -2.52 | 0.021934 |
| Fam55c | family with sequence similarity 55, member C | 627.85 | 376.50 | -1.67 | 0.001122 |
| Gbp4 | guanylate binding protein 4 | 31.10 | 222.45 | 7.15 | 0.000187 |
| Tmem229a | transmembrane protein 229A | 1057.64 | 431.33 | -2.45 | 0.012084 |
| Bub1 | budding uninhibited by benzimidazoles 1 homolog (S. cerevisiae) | 53.76 | 401.83 | 7.47 | 0.002175 |
|  | Rn.46170.1 | 424.02 | 861.33 | 2.03 | 0.000185 |
| Scel | sciellin | 43.48 | 16.64 | -2.61 | 0.006008 |
|  | Rn.74458.1 | 228.93 | 78.71 | -2.91 | 0.013088 |
| Magi2 | membrane associated guanylate kinase, WW and PDZ domain containing 2 | 624.15 | 319.41 | -1.95 | 0.000059 |
|  | Rn.45919.1 | 84.20 | 38.95 | -2.16 | 0.001794 |
|  | Rn.53665.1 | 781.02 | 312.31 | -2.50 | 0.000005 |
| Ebf3 | early B-cell factor 3 | 136.95 | 65.47 | -2.09 | 0.000664 |
| Glt25d1 | glycosyltransferase 25 domain containing 1 | 63.13 | 129.79 | 2.06 | 0.002942 |
|  | Rn.56671.1 | 241.80 | 70.12 | -3.45 | 0.000187 |
|  | Rn.53069.1 | 132.30 | 54.00 | -2.45 | 0.000363 |
| Epsti1 | epithelial stromal interaction 1 (breast) | 132.16 | 594.12 | 4.50 | 0.000081 |
| Runx1t1 | runt-related transcription factor 1; translocated to, 1 (cyclin D-related) | 125.02 | 52.06 | -2.40 | 0.009966 |
| Tcirg1 | T-cell, immune regulator 1, ATPase, H+ transporting, lysosomal V0 subunit A3 | 81.01 | 405.09 | 5.00 | 0.000003 |
| Pcdh20 | protocadherin 20 | 148.48 | 71.56 | -2.07 | 0.001092 |
| A2bp1 | ataxin 2 binding protein 1 | 2455.39 | 1107.81 | -2.22 | 0.016840 |
|  | Rn.78101.1 | 229.70 | 616.79 | 2.69 | 0.000443 |
|  | Rn.68897.1 | 261.49 | 104.29 | -2.51 | 0.000283 |
| Ccdc93 | coiled-coil domain containing 93 | 53.00 | 180.15 | 3.40 | 0.005580 |
| Ccdc93 | coiled-coil domain containing 93 | 84.17 | 284.85 | 3.38 | 0.000434 |
| Dhx58 | DEXH (Asp-Glu-X-His) box polypeptide 58 | 24.98 | 95.26 | 3.81 | 0.000956 |
| Dhx58 | DEXH (Asp-Glu-X-His) box polypeptide 58 | 19.07 | 60.08 | 3.15 | 0.002878 |
|  | Rn.20701.1 | 17.07 | 4.25 | -4.01 | 0.038684 |
|  | Rn.45584.1 | 330.01 | 197.98 | -1.67 | 0.000254 |
| Etv6 | ets variant 6 | 19.36 | 61.82 | 3.19 | 0.002919 |
|  | Rn.79771.1 | 19.25 | 59.36 | 3.08 | 0.001958 |
| LOC688502 | Similar to Protein arginine N-methyltransferase 4 (Heterogeneous nuclear ribonucleoprotein methyltransferase-like protein 4) | 248.27 | 94.19 | -2.64 | 0.000086 |
|  | Rn.40150.1 | 753.18 | 398.44 | -1.89 | 0.000139 |
|  | Rn.45689.1 | 133.68 | 59.96 | -2.23 | 0.002054 |
|  | Rn.70346.1 | 60.42 | 24.03 | -2.51 | 0.005013 |
|  | Rn.75463.2 | 96.59 | 24.54 | -3.94 | 0.001428 |
|  | Rn.74710.1 | 371.94 | 100.27 | -3.71 | 0.001185 |
| Steap4 | STEAP family member 4 | 218.60 | 1098.61 | 5.03 | 0.000295 |
| Cd8a | CD8a molecule | 4.34 | 646.18 | 149.04 | 0.002310 |
| Ccdc109b | coiled-coil domain containing 109B | 39.48 | 148.93 | 3.77 | 0.000162 |
| Ptger2 | Prostaglandin E receptor 2 (subtype EP2) | 69.61 | 199.48 | 2.87 | 0.001079 |
| Dhx58 | DEXH (Asp-Glu-X-His) box polypeptide 58 | 18.84 | 89.72 | 4.76 | 0.000643 |
| Hcst | hematopoietic cell signal transducer | 19.15 | 171.53 | 8.96 | 0.000691 |
|  | Rn.67976.1 | 622.63 | 267.57 | -2.33 | 0.000265 |
|  | Rn.75158.1 | 164.74 | 87.77 | -1.88 | 0.000800 |
|  | Rn.18677.1 | 117.48 | 54.54 | -2.15 | 0.020327 |
| Siglec5 | sialic acid binding Ig-like lectin 5 | 17.07 | 58.97 | 3.45 | 0.000104 |
| Pnmal2 | PNMA-like 2 | 925.92 | 168.92 | -5.48 | 0.019266 |
| Lnx1 | ligand of numb-protein X 1 | 92.73 | 48.38 | -1.92 | 0.016672 |
|  | Rn.23518.3 | 432.62 | 225.43 | -1.92 | 0.003047 |
| Runx1t1 | runt-related transcription factor 1; translocated to, 1 (cyclin D-related) | 197.48 | 92.63 | -2.13 | 0.007165 |
| Ermn | ermin, ERM-like protein | 1820.22 | 1082.13 | -1.68 | 0.000167 |
|  | Rn.15505.1 | 16.99 | 206.70 | 12.16 | 0.005645 |
| RGD1563441 | similar to RIKEN cDNA A030009H04 | 923.30 | 387.47 | -2.38 | 0.000090 |
|  | Rn.40879.1 | 407.70 | 155.44 | -2.62 | 0.000073 |
| LOC100366216 | nuclear antigen Sp100-like | 3.69 | 31.85 | 8.64 | 0.001008 |
|  | Rn.27718.1 | 98.50 | 498.64 | 5.06 | 0.000006 |
|  | Rn.23544.1 | 768.48 | 442.28 | -1.74 | 0.000129 |
| Mcoln2 | mucolipin 2 | 15.28 | 32.32 | 2.11 | 0.020173 |
| LOC679958 | similar to CG10806-PB, isoform B | 91.30 | 47.25 | -1.93 | 0.003330 |
|  | Rn.45443.1 | 904.45 | 366.61 | -2.47 | 0.000008 |
| Gpr84 | G protein-coupled receptor 84 | 105.14 | 510.72 | 4.86 | 0.001056 |
| Kif11 | kinesin family member 11 | 8.37 | 25.85 | 3.09 | 0.009930 |
|  | Rn.53159.1 | 387.72 | 172.44 | -2.25 | 0.000202 |
| Efemp2 | EGF-containing fibulin-like extracellular matrix protein 2 /// EGF-containing fibulin-like extracellular matrix protein 2-like | 104.56 | 297.44 | 2.84 | 0.000689 |
|  | Rn.15457.1 | 96.25 | 28.19 | -3.41 | 0.029118 |
|  | Rn.16405.1 | 316.23 | 140.65 | -2.25 | 0.002666 |
|  | Rn.16817.1 | 33.52 | 72.38 | 2.16 | 0.001920 |
| Cd5l | Cd5 molecule-like | 7.73 | 23.93 | 3.09 | 0.002006 |
|  | Rn.19786.1 | 111.34 | 431.90 | 3.88 | 0.003218 |
|  | Rn.20836.1 | 462.96 | 169.42 | -2.73 | 0.000215 |
| LOC682861 | similar to adenomatosis polyposis coli down-regulated 1 | 229.80 | 440.67 | 1.92 | 0.000111 |
|  | Rn.79975.1 | 25.88 | 205.11 | 7.92 | 0.000304 |
| Ifi204 | interferon activated gene 204 | 17.05 | 437.22 | 25.64 | 0.020116 |
| Dimt1l | DIM1 dimethyladenosine transferase 1-like (S. cerevisiae) | 84.79 | 206.45 | 2.43 | 0.000617 |
| Gdap1 | ganglioside-induced differentiation-associated-protein 1 | 1256.62 | 670.33 | -1.87 | 0.003949 |
|  | Rn.47661.1 | 708.78 | 383.29 | -1.85 | 0.000588 |
|  | Rn.51583.1 | 283.17 | 78.08 | -3.63 | 0.001151 |
|  | Rn.52214.1 | 124.44 | 60.37 | -2.06 | 0.001278 |
|  | Rn.53044.1 | 394.79 | 198.34 | -1.99 | 0.000109 |
| Thsd7b | Thrombospondin, type I, domain containing 7B | 237.10 | 112.26 | -2.11 | 0.002995 |
|  | Rn.57932.2 | 1136.99 | 530.93 | -2.14 | 0.000385 |
| Thbs2 | thrombospondin 2 | 118.22 | 726.27 | 6.14 | 0.018333 |
| Odf3 | outer dense fiber of sperm tails 3 | 17.64 | 2.79 | -6.33 | 0.014749 |
|  | Rn.74455.1 | 1033.63 | 360.57 | -2.87 | 0.000251 |
| Nxph1 | neurexophilin 1 | 526.54 | 282.75 | -1.86 | 0.002293 |
|  | Rn.68075.1 | 48.47 | 120.38 | 2.48 | 0.001076 |
| Secisbp2l | SECIS binding protein 2-like | 183.55 | 48.34 | -3.80 | 0.009391 |
| Sash3 | SAM and SH3 domain containing 3 | 121.52 | 441.45 | 3.63 | 0.000007 |
|  | Rn.81381.1 | 45.42 | 18.62 | -2.44 | 0.006561 |
|  | Rn.28788.1 | 81.23 | 30.87 | -2.63 | 0.002247 |
| Wdr51a | WD repeat domain 51A | 5.92 | 22.65 | 3.83 | 0.039708 |
|  | Rn.40553.1 | 320.99 | 656.53 | 2.05 | 0.004674 |
|  | Rn.51363.1 | 497.70 | 223.52 | -2.23 | 0.000151 |
|  | Rn.12021.1 | 570.62 | 252.85 | -2.26 | 0.000010 |
|  | Rn.23216.2 | 96.91 | 418.64 | 4.32 | 0.004487 |
|  | Rn.23216.2 | 74.62 | 186.34 | 2.50 | 0.002944 |
| LOC100125362 | hypothetical protein LOC100125362 | 938.77 | 386.22 | -2.43 | 0.000749 |
| Csf3r | colony stimulating factor 3 receptor (granulocyte) | 65.02 | 398.54 | 6.13 | 0.002592 |
|  | Rn.28221.2 | 162.57 | 83.09 | -1.96 | 0.000929 |
|  | Rn.26999.1 | 86.13 | 38.83 | -2.22 | 0.002096 |
|  | Rn.14934.2 | 681.23 | 319.91 | -2.13 | 0.001598 |
| Hist2h2be | histone cluster 2, H2be | 212.97 | 405.58 | 1.90 | 0.002233 |
| Ankrd57 | ankyrin repeat domain 57 | 262.45 | 787.28 | 3.00 | 0.000464 |
| LOC689147 | Hypothetical protein LOC689147 | 561.32 | 250.78 | -2.24 | 0.000014 |
| LOC689147 | Hypothetical protein LOC689147 | 116.65 | 59.97 | -1.95 | 0.002954 |
|  | Rn.63988.1 | 224.94 | 76.81 | -2.93 | 0.000067 |
| Lhfpl2 | lipoma HMGIC fusion partner-like 2 | 110.76 | 352.43 | 3.18 | 0.000128 |
|  | Rn.50664.2 | 83.46 | 16.22 | -5.14 | 0.004178 |
|  | Rn.43149.1 | 477.83 | 172.07 | -2.78 | 0.000007 |
|  | Rn.47647.1 | 17.40 | 81.60 | 4.69 | 0.000116 |
|  | Rn.66026.1 | 295.74 | 595.34 | 2.01 | 0.003600 |
| Eya2 | eyes absent homolog 2 (Drosophila) | 21.34 | 66.50 | 3.12 | 0.017962 |
|  | Rn.45492.2 | 395.13 | 1136.40 | 2.88 | 0.002093 |
| Sacs | spastic ataxia of Charlevoix-Saguenay (sacsin) | 128.28 | 65.55 | -1.96 | 0.001658 |
| Nxph1 | neurexophilin 1 | 403.60 | 182.08 | -2.22 | 0.000037 |
| Kcnd2 | Potassium voltage gated channel, Shal-related family, member 2 | 361.96 | 145.72 | -2.48 | 0.003378 |
| LOC100360914 | F-box and WD-40 domain protein 7 /// F-box and WD-40 domain protein 7, archipelago homolog (Drosophila)-like | 254.57 | 94.01 | -2.71 | 0.001625 |
| Ptplad2 | protein tyrosine phosphatase-like A domain containing 2 | 17.43 | 71.82 | 4.12 | 0.001265 |
|  | Rn.39299.2 | 7.87 | 19.08 | 2.42 | 0.007946 |
|  | Rn.62817.1 | 107.70 | 40.40 | -2.67 | 0.010986 |
| Cadps | Ca++-dependent secretion activator | 38.57 | 14.57 | -2.65 | 0.006413 |
| Ssr1 | signal sequence receptor, alpha | 102.06 | 206.65 | 2.02 | 0.001315 |
| Sc5dl | sterol-C5-desaturase (ERG3 delta-5-desaturase homolog, S. cerevisiae)-like | 2389.19 | 940.33 | -2.54 | 0.001660 |
| Abca8a | ATP-binding cassette, sub-family A (ABC1), member 8a | 1433.37 | 2565.54 | 1.79 | 0.000171 |
| Ttc33 | tetratricopeptide repeat domain 33 | 430.46 | 239.21 | -1.80 | 0.000693 |
|  | Rn.64565.1 | 137.76 | 65.19 | -2.11 | 0.000140 |
| RGD1359349 | similar to hypothetical protein MGC34760 | 16.11 | 138.14 | 8.58 | 0.006961 |
|  | Rn.15026.1 | 388.21 | 227.46 | -1.71 | 0.000741 |
| Rerg | RAS-like, estrogen-regulated, growth-inhibitor | 188.32 | 90.50 | -2.08 | 0.002351 |
| Cnnm1 | Cyclin M1 | 94.03 | 37.96 | -2.48 | 0.007800 |
| Pcdha1 | protocadherin alpha 1 /// protocadherin alpha 10 /// protocadherin alpha 11 /// protocadherin alpha 12 /// protocadherin alpha 13 /// protocadherin alpha 2 /// protocadherin alpha 3 /// protocadherin alpha 4 /// protocadherin alpha 5 /// protocadherin alpha 6 /// protocadherin alpha 7 /// protocadherin alpha 8 /// protocadherin alpha 9 /// protocadherin alpha subfamily C, 1 /// protocadherin alpha subfamily C, 2 | 1087.30 | 578.95 | -1.88 | 0.000041 |
| LOC100360116 | hypothetical protein LOC100360116 | 13.08 | 44.28 | 3.38 | 0.019694 |
| Pqlc3 | PQ loop repeat containing 3 | 511.14 | 957.90 | 1.87 | 0.000018 |
| Col16a1 | collagen, type XVI, alpha 1 | 669.84 | 1255.78 | 1.87 | 0.001500 |
|  | Rn.20519.1 | 70.62 | 217.65 | 3.08 | 0.004828 |
|  | Rn.42740.1 | 922.72 | 435.68 | -2.12 | 0.000059 |
|  | Rn.22374.1 | 259.00 | 2040.41 | 7.88 | 0.000658 |
|  | Rn.27824.1 | 662.66 | 297.76 | -2.23 | 0.000019 |
| Cbln1 | cerebellin 1 precursor | 184.82 | 60.04 | -3.08 | 0.000377 |
|  | Rn.20456.1 | 635.75 | 366.21 | -1.74 | 0.005054 |
| Kif11 | kinesin family member 11 | 24.48 | 162.16 | 6.62 | 0.002488 |
|  | Rn.28213.1 | 979.09 | 490.07 | -2.00 | 0.000030 |
|  | Rn.35394.1 | 39.12 | 88.33 | 2.26 | 0.011288 |
|  | Rn.17796.1 | 55.41 | 373.01 | 6.73 | 0.002191 |
| Pcp4l1 | Purkinje cell protein 4-like 1 | 835.09 | 332.27 | -2.51 | 0.000044 |
| Fli1 | Friend leukemia virus integration 1 | 156.57 | 664.67 | 4.25 | 0.010038 |
| Adamts15 | ADAM metallopeptidase with thrombospondin type 1 motif, 15 | 95.38 | 191.07 | 2.00 | 0.010145 |
|  | Rn.34220.1 | 10.87 | 117.72 | 10.83 | 0.003238 |
|  | Rn.9566.1 | 1344.95 | 525.17 | -2.56 | 0.000015 |
| Ppm1h | protein phosphatase 1H (PP2C domain containing) | 69.16 | 151.52 | 2.19 | 0.001507 |
| Lynx1 | Ly6/neurotoxin 1 | 935.59 | 411.51 | -2.27 | 0.000571 |
|  | Rn.18114.1 | 270.03 | 146.16 | -1.85 | 0.001636 |
|  | Rn.46076.1 | 1614.61 | 778.30 | -2.07 | 0.000209 |
|  | Rn.38287.1 | 104.38 | 39.26 | -2.66 | 0.000466 |
|  | Rn.39284.1 | 370.09 | 187.68 | -1.97 | 0.000020 |
| Pcdh8 | Protocadherin 8 | 320.80 | 172.65 | -1.86 | 0.000826 |
| Ccdc69 | coiled-coil domain containing 69 | 32.29 | 119.41 | 3.70 | 0.000548 |
| Slitrk1 | SLIT and NTRK-like family, member 1 | 687.47 | 289.55 | -2.37 | 0.000008 |
|  | Rn.9531.1 | 120.80 | 221.50 | 1.83 | 0.000529 |
| Camta1 | similar to KIAA0833 protein | 1831.11 | 994.55 | -1.84 | 0.000161 |
| Sez6 | seizure related 6 homolog (mouse) | 387.51 | 200.62 | -1.93 | 0.000098 |
| Iqsec3 | IQ motif and Sec7 domain 3 | 415.58 | 215.70 | -1.93 | 0.009661 |
| Kif23 | kinesin family member 23 | 19.30 | 157.23 | 8.15 | 0.002259 |
|  | Rn.46342.1 | 98.71 | 39.52 | -2.50 | 0.009278 |
|  | Rn.37831.1 | 1966.77 | 946.03 | -2.08 | 0.000003 |
| Rgs17 | regulator of G-protein signaling 17 | 362.00 | 124.81 | -2.90 | 0.001434 |
|  | Rn.49180.1 | 31.22 | 71.15 | 2.28 | 0.005122 |
|  | Rn.46298.1 | 90.27 | 28.22 | -3.20 | 0.004306 |
| Arhgap22 | Rho GTPase activating protein 22 | 136.59 | 238.50 | 1.75 | 0.001158 |
|  | Rn.46224.1 | 276.57 | 112.94 | -2.45 | 0.000093 |
|  | Rn.32812.1 | 642.82 | 146.97 | -4.37 | 0.000528 |
| Mmp19 | matrix metallopeptidase 19 | 11.75 | 117.83 | 10.03 | 0.000182 |
|  | Rn.20457.1 | 65.50 | 455.07 | 6.95 | 0.003250 |
| Ggta1 | Glycoprotein, alpha-galactosyltransferase 1 | 14.07 | 52.60 | 3.74 | 0.014218 |
|  | Rn.24253.1 | 797.74 | 395.49 | -2.02 | 0.000052 |
|  | Rn.16850.1 | 57.03 | 132.04 | 2.32 | 0.012329 |
|  | Rn.46334.1 | 82.93 | 30.10 | -2.76 | 0.001814 |
| LOC690700 | similar to similar to RIKEN cDNA 1700001E04 /// similar to Discs large homolog 5 (Placenta and prostate DLG) (Discs large protein P-dlg) | 392.25 | 109.08 | -3.60 | 0.003767 |
|  | Rn.19718.1 | 563.36 | 301.71 | -1.87 | 0.000047 |
|  | Rn.52842.1 | 688.16 | 372.52 | -1.85 | 0.000725 |
| Whsc1 | Wolf-Hirschhorn syndrome candidate 1 (human) | 41.73 | 102.42 | 2.45 | 0.011790 |
|  | Rn.20435.1 | 170.39 | 96.06 | -1.77 | 0.001742 |
| Spry3 | sprouty homolog 3 (Drosophila) | 175.54 | 80.35 | -2.18 | 0.002108 |
| RGD1311892 | similar to hypothetical protein FLJ10901 | 63.57 | 137.43 | 2.16 | 0.020895 |
| Pcdh20 | protocadherin 20 | 152.61 | 66.66 | -2.29 | 0.000602 |
| Fam26f | family with sequence similarity 26, member F | 37.13 | 83.36 | 2.25 | 0.010547 |
|  | Rn.20252.1 | 213.49 | 684.57 | 3.21 | 0.005703 |
| Camk1g | calcium/calmodulin-dependent protein kinase IG | 278.05 | 111.30 | -2.50 | 0.000941 |
| Etv1 | ets variant 1 | 282.83 | 140.34 | -2.02 | 0.001471 |
|  | Rn.45419.1 | 391.05 | 154.54 | -2.53 | 0.005648 |
| Srgap2 | SLIT-ROBO Rho GTPase activating protein 2 | 29.65 | 67.32 | 2.27 | 0.005118 |
| Zfyve9 | zinc finger, FYVE domain containing 9 | 39.81 | 12.82 | -3.11 | 0.044673 |
| LOC100363969 | zinc finger protein 457-like /// similar to zinc finger protein 458 | 19.33 | 50.54 | 2.61 | 0.003591 |
|  | Rn.12695.1 | 176.87 | 79.98 | -2.21 | 0.001692 |
|  | Rn.39112.1 | 4495.49 | 1692.85 | -2.66 | 0.016727 |
|  | Rn.33730.1 | 462.06 | 229.73 | -2.01 | 0.000035 |
| Ska1 | spindle and kinetochore associated complex subunit 1 | 21.14 | 82.61 | 3.91 | 0.004916 |
| Hoxb5 | homeo box B5 | 233.86 | 122.92 | -1.90 | 0.004438 |
|  | Rn.45499.1 | 902.74 | 298.95 | -3.02 | 0.000017 |
|  | Rn.18897.1 | 318.64 | 176.97 | -1.80 | 0.000397 |
|  | Rn.61873.1 | 50.46 | 19.90 | -2.54 | 0.017614 |
| Crtac1 | cartilage acidic protein 1 | 324.46 | 114.09 | -2.84 | 0.000142 |
|  | Rn.43762.1 | 7.90 | 27.07 | 3.43 | 0.002118 |
| Thsd7a | thrombospondin, type I, domain containing 7A | 711.98 | 280.70 | -2.54 | 0.006814 |
| LOC679811 | similar to RIKEN cDNA D930015E06 | 18.52 | 57.44 | 3.10 | 0.003064 |
| Nckipsd | NCK interacting protein with SH3 domain | 931.08 | 554.65 | -1.68 | 0.000066 |
| Pltp | phospholipid transfer protein | 311.68 | 2366.63 | 7.59 | 0.000159 |
|  | Rn.13674.1 | 201.31 | 114.80 | -1.75 | 0.000282 |
|  | Rn.63969.1 | 223.23 | 97.05 | -2.30 | 0.001510 |
| Ebi3 | Epstein-Barr virus induced 3 | 59.90 | 293.12 | 4.89 | 0.000157 |
| Ebpl | emopamil binding protein-like | 288.76 | 574.14 | 1.99 | 0.000065 |
| Ddx58 | DEAD (Asp-Glu-Ala-Asp) box polypeptide 58 | 137.06 | 321.22 | 2.34 | 0.000509 |
|  | Rn.48992.1 | 22.33 | 43.10 | 1.93 | 0.005454 |
| Dzip1 | DAZ interacting protein 1 | 856.17 | 383.87 | -2.23 | 0.000034 |
|  | Rn.55798.1 | 72.06 | 137.56 | 1.91 | 0.008721 |
|  | Rn.54591.1 | 213.75 | 124.23 | -1.72 | 0.001133 |
|  | Rn.19658.1 | 888.25 | 439.90 | -2.02 | 0.000041 |
| Irgm | immunity-related GTPase family, M | 87.86 | 183.65 | 2.09 | 0.006671 |
|  | Rn.51282.1 | 245.83 | 73.78 | -3.33 | 0.002200 |
| Centd3 | centaurin, delta 3 | 76.70 | 168.41 | 2.20 | 0.003753 |
| Phf17 | PHD finger protein 17 | 175.37 | 67.94 | -2.58 | 0.000105 |
| Lrrc40 | Leucine rich repeat containing 40 | 185.56 | 75.50 | -2.46 | 0.000312 |
|  | Rn.16889.1 | 445.03 | 153.65 | -2.90 | 0.000002 |
| Nab2 | Ngfi-A binding protein 2 | 16.98 | 48.15 | 2.84 | 0.001039 |
|  | Rn.17646.1 | 297.94 | 172.00 | -1.73 | 0.000204 |
| Kcnq2 | potassium voltage-gated channel, KQT-like subfamily, member 2 | 192.81 | 96.38 | -2.00 | 0.001023 |
|  | Rn.16287.1 | 1488.25 | 661.60 | -2.25 | 0.000081 |
|  | Rn.20691.1 | 78.63 | 188.38 | 2.40 | 0.000225 |
| Mum1l1 | melanoma associated antigen (mutated) 1-like 1 | 142.74 | 49.57 | -2.88 | 0.000060 |
| Hapln4 | hyaluronan and proteoglycan link protein 4 | 972.54 | 365.19 | -2.66 | 0.000001 |
|  | Rn.34078.1 | 97.66 | 200.64 | 2.05 | 0.001811 |
|  | Rn.31458.1 | 22.43 | 66.14 | 2.95 | 0.003424 |
|  | Rn.17858.1 | 7.93 | 51.18 | 6.46 | 0.005273 |
| Gpr75 | G protein-coupled receptor 75 | 55.14 | 22.90 | -2.41 | 0.001385 |
|  | Rn.38860.1 | 551.75 | 152.43 | -3.62 | 0.017304 |
| Ss18 | synovial sarcoma translocation, Chromosome 18 | 66.86 | 168.12 | 2.51 | 0.001919 |
| Tbx18 | T-box18 | 24.84 | 93.77 | 3.77 | 0.023211 |
|  | Rn.46939.1 | 190.58 | 89.17 | -2.14 | 0.002141 |
| Arhgap15 | Rho GTPase activating protein 15 /// rho GTPase-activating protein 15-like | 63.97 | 199.64 | 3.12 | 0.000007 |
| Gabrg2 | gamma-aminobutyric acid (GABA) A receptor, gamma 2 | 892.72 | 307.89 | -2.90 | 0.000003 |
|  | Rn.15458.1 | 47.17 | 112.89 | 2.39 | 0.009776 |
|  | Rn.50516.1 | 415.30 | 219.55 | -1.89 | 0.000701 |
| MAST1 | microtubule associated serine/threonine kinase 1 | 307.06 | 107.37 | -2.86 | 0.000041 |
|  | Rn.21199.1 | 203.59 | 110.14 | -1.85 | 0.001510 |
|  | Rn.19506.1 | 61.69 | 29.75 | -2.07 | 0.001406 |
|  | Rn.28177.1 | 186.10 | 101.90 | -1.83 | 0.000313 |
|  | Rn.45204.1 | 1060.88 | 591.60 | -1.79 | 0.000120 |
| Mab21l2 | mab-21-like 2 (C. elegans) | 185.04 | 65.88 | -2.81 | 0.000929 |
|  | Rn.47716.1 | 217.77 | 76.18 | -2.86 | 0.004632 |
| Ncf4 | neutrophil cytosolic factor 4 | 49.02 | 581.26 | 11.86 | 0.002619 |
|  | Rn.15212.1 | 89.98 | 159.36 | 1.77 | 0.000088 |
| Nxnl2 | nucleoredoxin-like 2 | 37.58 | 13.20 | -2.85 | 0.023390 |
|  | Rn.62732.1 | 103.17 | 51.07 | -2.02 | 0.001934 |
| Ptpn4 | protein tyrosine phosphatase, non-receptor type 4 | 148.15 | 45.07 | -3.29 | 0.015695 |
|  | Rn.53284.1 | 147.59 | 53.13 | -2.78 | 0.000269 |
| Slc12a7 | solute carrier family 12 (potassium/chloride transporters), member 7 | 39.04 | 5.66 | -6.89 | 0.001097 |
|  | Rn.12095.1 | 46.14 | 354.65 | 7.69 | 0.000684 |
|  | Rn.20433.1 | 650.82 | 217.58 | -2.99 | 0.006828 |
|  | Rn.9217.1 | 907.72 | 469.29 | -1.93 | 0.000393 |
|  | Rn.64391.1 | 46.84 | 17.05 | -2.75 | 0.011086 |
| Kif1b | kinesin family member 1B /// phosphogluconate dehydrogenase | 895.74 | 305.94 | -2.93 | 0.010266 |
|  | Rn.45026.1 | 5.74 | 19.01 | 3.31 | 0.004580 |
|  | Rn.57308.1 | 29.65 | 10.80 | -2.75 | 0.020482 |
|  | Rn.46367.1 | 302.42 | 165.78 | -1.82 | 0.000314 |
| Bcl11b | B-cell CLL/lymphoma 11B (zinc finger protein) | 104.05 | 38.65 | -2.69 | 0.005808 |
|  | Rn.72370.1 | 34.48 | 90.88 | 2.64 | 0.001122 |
|  | Rn.57436.1 | 132.54 | 40.45 | -3.28 | 0.002484 |
| Six4 | SIX homeobox 4 | 154.00 | 75.25 | -2.05 | 0.017012 |
|  | Rn.6731.1 | 74.05 | 767.88 | 10.37 | 0.000049 |
| Nrip3 | nuclear receptor interacting protein 3 | 123.32 | 49.95 | -2.47 | 0.018783 |
|  | Rn.30602.1 | 321.82 | 588.50 | 1.83 | 0.000152 |
|  | Rn.42371.1 | 19.30 | 46.77 | 2.42 | 0.001758 |
|  | Rn.53535.1 | 270.89 | 114.90 | -2.36 | 0.000176 |
| Tmem22 | transmembrane protein 22 | 369.84 | 197.37 | -1.87 | 0.000162 |
| Rab33a | RAB33A, member of RAS oncogene family | 932.24 | 545.47 | -1.71 | 0.000350 |
|  | Rn.61399.1 | 21.67 | 7.02 | -3.09 | 0.010137 |
| Sbno2 | strawberry notch homolog 2 (Drosophila) | 12.45 | 102.04 | 8.19 | 0.007075 |
|  | Rn.16727.1 | 92.99 | 46.60 | -2.00 | 0.002153 |
|  | Rn.57841.1 | 118.60 | 55.12 | -2.15 | 0.006232 |
|  | Rn.33237.1 | 28.04 | 87.95 | 3.14 | 0.003491 |
| Srgap2 | SLIT-ROBO Rho GTPase activating protein 2 | 151.84 | 282.45 | 1.86 | 0.000875 |
|  | Rn.55980.1 | 602.80 | 253.63 | -2.38 | 0.000192 |
|  | Rn.47224.1 | 113.71 | 37.78 | -3.01 | 0.001587 |
|  | Rn.49062.1 | 59.22 | 22.37 | -2.65 | 0.017257 |
|  | Rn.35726.1 | 1178.41 | 435.20 | -2.71 | 0.004272 |
| Chi3l1 | chitinase 3-like 1 | 331.99 | 1237.79 | 3.73 | 0.028027 |
| Ccl9 | chemokine (C-C motif) ligand 9 | 13.08 | 337.51 | 25.80 | 0.011754 |
| Fam132a | family with sequence similarity 132, member A | 89.18 | 47.31 | -1.89 | 0.001112 |
|  | Rn.26742.1 | 577.80 | 265.70 | -2.17 | 0.000132 |
|  | Rn.21571.1 | 402.42 | 104.02 | -3.87 | 0.003627 |
| Letm2 | leucine zipper-EF-hand containing transmembrane protein 2 | 303.33 | 151.27 | -2.01 | 0.000177 |
| Tlr2 | toll-like receptor 2 | 35.97 | 626.49 | 17.42 | 0.000143 |
|  | Rn.18590.1 | 147.92 | 34.54 | -4.28 | 0.000839 |
| Nos1ap | nitric oxide synthase 1 (neuronal) adaptor protein | 316.99 | 104.65 | -3.03 | 0.001980 |
| Sh3tc1 | SH3 domain and tetratricopeptide repeats 1 | 89.24 | 245.38 | 2.75 | 0.000692 |
|  | Rn.67025.1 | 550.14 | 251.54 | -2.19 | 0.000094 |
|  | Rn.64479.1 | 5.49 | 32.67 | 5.95 | 0.000379 |
|  | Rn.47845.1 | 392.66 | 221.39 | -1.77 | 0.000512 |
|  | Rn.58010.1 | 294.84 | 115.79 | -2.55 | 0.005890 |
|  | Rn.34662.1 | 196.13 | 85.42 | -2.30 | 0.000143 |
|  | Rn.23173.2 | 13.40 | 28.28 | 2.11 | 0.008886 |
|  | Rn.20064.1 | 26.64 | 93.90 | 3.52 | 0.008560 |
| Map7d2 | MAP7 domain containing 2 | 1667.59 | 794.31 | -2.10 | 0.000075 |
| Rab6b | RAB6B, member RAS oncogene family | 132.31 | 58.29 | -2.27 | 0.001419 |
| Cryzl1 | crystallin, zeta (quinone reductase)-like 1 | 876.41 | 459.92 | -1.91 | 0.000112 |
| Klra17 | killer cell lectin-like receptor, subfamily A, member 17 /// immunoreceptor Ly49si3-like /// hypothetical protein LOC497796 /// similar to immunoreceptor Ly49si1 /// Ly49 inhibitory receptor 5 /// immunoreceptor Ly49si1 /// immunoreceptor Ly49si2 /// immunoreceptor Ly49si3 /// similar to immunoreceptor Ly49si3 | 41.71 | 739.13 | 17.72 | 0.006798 |
| Stard4 | StAR-related lipid transfer (START) domain containing 4 | 516.51 | 261.39 | -1.98 | 0.000434 |
|  | Rn.18407.1 | 25.37 | 66.90 | 2.64 | 0.023471 |
| Csf2ra | Granulocyte-macrophage colony stimulating receptor alpha | 63.66 | 607.37 | 9.54 | 0.002564 |
|  | Rn.3287.2 | 290.75 | 549.04 | 1.89 | 0.000586 |
| MGC105649 | hypothetical LOC302884 | 30.04 | 250.92 | 8.35 | 0.003418 |
|  | Rn.17556.2 | 8.83 | 58.07 | 6.57 | 0.000563 |
| Etv1 | Ets variant 1 | 116.90 | 52.49 | -2.23 | 0.005263 |
|  | Rn.23854.1 | 237.16 | 669.02 | 2.82 | 0.002366 |
| Obfc2a | oligonucleotide/oligosaccharide-binding fold containing 2A | 61.00 | 169.01 | 2.77 | 0.000700 |
|  | Rn.27192.1 | 641.61 | 1750.01 | 2.73 | 0.000255 |
|  | Rn.82246.1 | 9.09 | 258.56 | 28.44 | 0.001277 |
|  | Rn.19395.1 | 14.63 | 68.16 | 4.66 | 0.000117 |
|  | Rn.45378.1 | 271.53 | 153.98 | -1.76 | 0.003861 |
|  | Rn.2746.1 | 106.10 | 478.29 | 4.51 | 0.009740 |
|  | Rn.40579.1 | 95.51 | 197.62 | 2.07 | 0.001031 |
| LOC691143 | Similar to Serum amyloid A-3 protein precursor | 16.82 | 1132.09 | 67.32 | 0.033560 |
| Mrc1 | mannose receptor, C type 1 | 90.20 | 791.38 | 8.77 | 0.009812 |
|  | Rn.12670.1 | 159.23 | 758.75 | 4.76 | 0.000443 |
| Gpr182 | G protein-coupled receptor 182 | 83.55 | 198.86 | 2.38 | 0.006814 |
| Rassf1 | Ras association (RalGDS/AF-6) domain family member 1 | 40.57 | 97.11 | 2.39 | 0.007790 |
| Mthfs | 5,10-methenyltetrahydrofolate synthetase (5-formyltetrahydrofolate cyclo-ligase) | 186.33 | 348.71 | 1.87 | 0.000099 |
| Dapp1 | dual adaptor of phosphotyrosine and 3-phosphoinositides | 102.60 | 329.17 | 3.21 | 0.000013 |
| Nusap1 | nucleolar and spindle associated protein 1 | 13.30 | 140.36 | 10.56 | 0.012334 |
|  | Rn.55343.1 | 193.77 | 92.05 | -2.11 | 0.000397 |
|  | Rn.15979.1 | 229.85 | 489.19 | 2.13 | 0.001207 |
| Adam8 | ADAM metallopeptidase domain 8 | 63.93 | 268.33 | 4.20 | 0.001320 |
| Lrrc8c | leucine rich repeat containing 8 family, member C | 34.86 | 89.76 | 2.57 | 0.000964 |
| Trem2 | triggering receptor expressed on myeloid cells 2 | 50.70 | 510.68 | 10.07 | 0.000413 |
|  | Rn.45578.1 | 503.83 | 295.47 | -1.71 | 0.000307 |
| RGD1561145 | similar to novel protein | 21.72 | 79.04 | 3.64 | 0.000749 |
| Snhg11 | small nucleolar RNA host gene 11 | 1795.29 | 685.89 | -2.62 | 0.000575 |
| Angptl1 | angiopoietin-like 1 | 273.48 | 99.62 | -2.75 | 0.030531 |
| Fgf9 | fibroblast growth factor 9 | 1267.99 | 431.33 | -2.94 | 0.000293 |
|  | Rn.20780.1 | 146.22 | 79.14 | -1.85 | 0.000736 |
|  | Rn.23123.1 | 139.82 | 52.96 | -2.64 | 0.000103 |
|  | Rn.34582.1 | 780.91 | 401.03 | -1.95 | 0.000185 |
| Pds5b | PDS5, regulator of cohesion maintenance, homolog B (S. cerevisiae) | 839.95 | 461.17 | -1.82 | 0.000199 |
| Fgl2 | fibrinogen-like 2 | 924.53 | 1844.28 | 1.99 | 0.002049 |
| Prc1 | protein regulator of cytokinesis 1 | 32.63 | 384.91 | 11.80 | 0.000531 |
|  | Rn.17699.1 | 808.94 | 482.51 | -1.68 | 0.000183 |
| Znf710 | zinc finger protein 710 | 408.16 | 917.79 | 2.25 | 0.000237 |
|  | Rn.23625.1 | 318.30 | 805.47 | 2.53 | 0.000637 |
|  | Rn.22115.1 | 760.30 | 422.68 | -1.80 | 0.000144 |
|  | Rn.9241.1 | 358.76 | 823.75 | 2.30 | 0.000068 |
|  | Rn.11575.1 | 109.96 | 405.74 | 3.69 | 0.004929 |
| Ssbp2 | single-stranded DNA binding protein 2 | 536.76 | 310.05 | -1.73 | 0.000184 |
|  | Rn.70548.1 | 1825.04 | 913.67 | -2.00 | 0.001592 |
| Nsdhl | NAD(P) dependent steroid dehydrogenase-like | 779.60 | 413.11 | -1.89 | 0.000060 |
|  | Rn.15748.1 | 1920.53 | 1115.72 | -1.72 | 0.000186 |
| Nin | ninein (GSK3B interacting protein) | 40.77 | 82.94 | 2.03 | 0.009669 |
|  | Rn.47427.1 | 364.25 | 164.35 | -2.22 | 0.003450 |
| LOC100361631 | hypothetical protein LOC100361631 | 41.41 | 161.94 | 3.91 | 0.003739 |
|  | Rn.33190.1 | 69.60 | 153.06 | 2.20 | 0.009042 |
| Fcgr1a | Fc fragment of IgG, high affinity Ia, receptor (CD64) | 91.20 | 612.49 | 6.72 | 0.000680 |
|  | Rn.24657.1 | 40.70 | 19.99 | -2.04 | 0.012227 |
| Smc2 | structural maintenance of chromosomes 2 | 360.68 | 792.18 | 2.20 | 0.001161 |
| Rnf217 | ring finger protein 217 | 85.98 | 178.40 | 2.07 | 0.001922 |
| Myo1g | myosin IG | 25.38 | 122.22 | 4.82 | 0.000030 |
|  | Rn.35033.1 | 76.48 | 229.93 | 3.01 | 0.001058 |
| Man1c1 | mannosidase, alpha, class 1C, member 1 | 154.22 | 461.84 | 2.99 | 0.006844 |
|  | Rn.19982.1 | 724.22 | 312.30 | -2.32 | 0.000091 |
|  | Rn.20656.1 | 527.54 | 180.13 | -2.93 | 0.000036 |
| Eid2 | EP300 interacting inhibitor of differentiation 2 | 812.40 | 481.23 | -1.69 | 0.000053 |
| Eif5a2 | eukaryotic translation initiation factor 5A2 | 1607.48 | 913.16 | -1.76 | 0.000171 |
|  | Rn.22807.1 | 929.73 | 431.23 | -2.16 | 0.000298 |
| RGD1563072 | similar to hypothetical protein FLJ38984 | 833.54 | 398.14 | -2.09 | 0.000014 |
| Rasgef1a | RasGEF domain family, member 1A | 441.05 | 147.79 | -2.98 | 0.000027 |
| Ppp1r14c | protein phosphatase 1, regulatory (inhibitor) subunit 14c | 194.14 | 104.83 | -1.85 | 0.002941 |
| Hrasls | HRAS-like suppressor | 176.30 | 60.36 | -2.92 | 0.000049 |
|  | Rn.2624.1 | 120.96 | 281.66 | 2.33 | 0.002934 |
|  | Rn.24264.1 | 232.51 | 75.43 | -3.08 | 0.000014 |
| Ubtd1 | ubiquitin domain containing 1 | 82.15 | 185.92 | 2.26 | 0.000085 |
| P4ha3 | Procollagen-proline, 2-oxoglutarate 4-dioxygenase (proline 4-hydroxylase), alpha polypeptide III | 27.26 | 73.12 | 2.68 | 0.013573 |
| Tctex1d1 | Tctex1 domain containing 1 | 125.16 | 55.97 | -2.24 | 0.008824 |
| Zc3h12a | zinc finger CCCH type containing 12A | 20.90 | 185.99 | 8.90 | 0.000698 |
|  | Rn.43156.1 | 401.40 | 148.71 | -2.70 | 0.000909 |
|  | Rn.16334.1 | 494.26 | 916.05 | 1.85 | 0.002853 |
| Nmi | N-myc (and STAT) interactor | 118.75 | 301.77 | 2.54 | 0.000186 |
| Pcdha1 | protocadherin alpha 1 /// protocadherin alpha 10 /// protocadherin alpha 11 /// protocadherin alpha 12 /// protocadherin alpha 13 /// protocadherin alpha 2 /// protocadherin alpha 3 /// protocadherin alpha 4 /// protocadherin alpha 5 /// protocadherin alpha 6 /// protocadherin alpha 7 /// protocadherin alpha 8 /// protocadherin alpha 9 /// protocadherin alpha subfamily C, 1 /// protocadherin alpha subfamily C, 2 | 875.47 | 442.75 | -1.98 | 0.000249 |
|  | Rn.24885.1 | 117.42 | 61.52 | -1.91 | 0.002488 |
| Slc39a6 | solute carrier family 39 (zinc transporter), member 6 | 197.42 | 640.52 | 3.24 | 0.000269 |
| Nfasc | neurofascin | 114.61 | 268.05 | 2.34 | 0.036764 |
|  | Rn.7695.1 | 179.25 | 359.44 | 2.01 | 0.000995 |
|  | Rn.19349.1 | 13.93 | 51.31 | 3.68 | 0.013326 |
|  | Rn.20903.1 | 191.71 | 98.53 | -1.95 | 0.004304 |
| Abhd8 | abhydrolase domain containing 8 | 1189.40 | 557.84 | -2.13 | 0.001874 |
| Lrmp | lymphoid-restricted membrane protein | 110.76 | 273.84 | 2.47 | 0.000316 |
| Abcg3l1 | ATP-binding cassette, sub-family G (WHITE), member 3-like 1 | 162.35 | 348.92 | 2.15 | 0.007976 |
| C2 | complement component 2 | 21.70 | 373.93 | 17.23 | 0.005947 |
|  | Rn.28091.1 | 306.38 | 161.75 | -1.89 | 0.016894 |
| Hpdl | 4-hydroxyphenylpyruvate dioxygenase-like | 100.02 | 35.71 | -2.80 | 0.007469 |
| Efemp2 | EGF-containing fibulin-like extracellular matrix protein 2 | 135.21 | 514.44 | 3.80 | 0.000559 |
|  | Rn.24916.2 | 136.42 | 746.03 | 5.47 | 0.000026 |
|  | Rn.40243.1 | 234.24 | 594.24 | 2.54 | 0.021600 |
| Zfp365 | zinc finger protein 365 | 163.44 | 69.83 | -2.34 | 0.001678 |
|  | Rn.24450.1 | 507.17 | 258.54 | -1.96 | 0.000122 |
|  | Rn.15600.1 | 2150.51 | 1112.00 | -1.93 | 0.008560 |
| RGD1311578 | similar to PRO1853 homolog | 323.90 | 160.08 | -2.02 | 0.000077 |
| Cav1 | caveolin 1, caveolae protein | 76.70 | 160.66 | 2.09 | 0.004541 |
| Ankrd34b | ankyrin repeat domain 34B | 807.00 | 294.36 | -2.74 | 0.000003 |
| Ube2ql1 | ubiquitin-conjugating enzyme E2Q family-like 1 | 269.62 | 130.95 | -2.06 | 0.000321 |
|  | Rn.46260.1 | 275.48 | 112.75 | -2.44 | 0.000947 |
|  | Rn.33681.1 | 52.72 | 232.58 | 4.41 | 0.002894 |
|  | Rn.27183.1 | 1077.98 | 584.65 | -1.84 | 0.000838 |
| LOC503192 | zinc finger protein ZFP | 821.49 | 467.68 | -1.76 | 0.001826 |
| Traf3ip3 | TRAF3 interacting protein 3 | 9.21 | 43.96 | 4.78 | 0.001619 |
|  | Rn.54709.1 | 66.33 | 157.79 | 2.38 | 0.002230 |
| U2af1l4 | U2 small nuclear RNA auxiliary factor 1-like 4 | 46.55 | 23.80 | -1.96 | 0.015888 |
|  | Rn.12322.1 | 45.04 | 142.11 | 3.16 | 0.003851 |
| Swap70 | SWAP switching B-cell complex 70 | 261.39 | 502.58 | 1.92 | 0.002836 |
| Scx | scleraxis | 228.46 | 100.96 | -2.26 | 0.001335 |
| Ccdc125 | coiled-coil domain containing 125 | 186.76 | 520.77 | 2.79 | 0.000165 |
|  | Rn.12804.1 | 234.42 | 89.67 | -2.61 | 0.000152 |
| Rdh10 | Retinol dehydrogenase 10 (all-trans) | 266.43 | 969.34 | 3.64 | 0.004930 |
| Opn3 | opsin 3 | 293.88 | 134.39 | -2.19 | 0.002347 |
|  | Rn.47916.2 | 214.28 | 72.49 | -2.96 | 0.000415 |
|  | Rn.8245.2 | 48.99 | 21.61 | -2.27 | 0.005285 |
| Shd | Src homology 2 domain-containing transforming protein D | 87.71 | 41.32 | -2.12 | 0.007351 |
|  | Rn.51610.1 | 65.58 | 15.62 | -4.20 | 0.001321 |
|  | Rn.65030.1 | 35.36 | 120.80 | 3.42 | 0.000770 |
|  | Rn.19100.1 | 254.31 | 97.58 | -2.61 | 0.001168 |
| Fam3c | family with sequence similarity 3, member C | 499.26 | 1481.00 | 2.97 | 0.002559 |
| RGD1310964 | similar to RIKEN cDNA 9430031J16 | 262.51 | 114.49 | -2.29 | 0.000915 |
|  | Rn.20345.1 | 342.98 | 194.75 | -1.76 | 0.000074 |
| Fam158a | family with sequence similarity 158, member A | 475.83 | 176.37 | -2.70 | 0.000184 |
|  | Rn.15389.1 | 21.52 | 6.74 | -3.19 | 0.015254 |
| Lrrc33 | leucine rich repeat containing 33 | 114.80 | 534.25 | 4.65 | 0.003258 |
|  | Rn.61982.1 | 419.85 | 211.02 | -1.99 | 0.000300 |
|  | Rn.16180.1 | 295.41 | 142.90 | -2.07 | 0.000315 |
| Golsyn | Golgi-localized protein | 694.59 | 347.47 | -2.00 | 0.000025 |
| Gal3st4 | galactose-3-O-sulfotransferase 4 | 59.09 | 127.07 | 2.15 | 0.005887 |
|  | Rn.23866.1 | 200.85 | 388.61 | 1.93 | 0.005137 |
| Zfyve28 | zinc finger, FYVE domain containing 28 | 212.49 | 76.06 | -2.79 | 0.000078 |
|  | Rn.58482.1 | 758.81 | 1240.60 | 1.63 | 0.000216 |
|  | Rn.46061.1 | 277.96 | 104.28 | -2.67 | 0.002971 |
|  | Rn.32972.1 | 167.74 | 51.04 | -3.29 | 0.004351 |
| Aspm | asp (abnormal spindle) homolog, microcephaly associated (Drosophila) | 39.59 | 196.98 | 4.98 | 0.005363 |
| Gpcpd1 | glycerophosphocholine phosphodiesterase GDE1 homolog (S. cerevisiae) | 19.31 | 49.60 | 2.57 | 0.011764 |
| Necab3 | N-terminal EF-hand calcium binding protein 3 | 208.09 | 79.04 | -2.63 | 0.000608 |
| Cnot6l | CCR4-NOT transcription complex, subunit 6-like | 72.03 | 206.05 | 2.86 | 0.005209 |
|  | Rn.73010.1 | 516.52 | 166.75 | -3.10 | 0.000079 |
|  | Rn.16043.1 | 364.41 | 176.54 | -2.06 | 0.001317 |
| Hlx | H2.0-like homeobox | 17.59 | 120.47 | 6.85 | 0.000012 |
| Tubb4 | tubulin, beta 4 | 1189.03 | 635.17 | -1.87 | 0.001613 |
|  | Rn.48124.1 | 563.90 | 219.95 | -2.56 | 0.000146 |
| Ptger4 | Prostaglandin E receptor 4 | 43.00 | 97.43 | 2.27 | 0.004640 |
| Myo10 | myosin X | 161.69 | 317.94 | 1.97 | 0.003316 |
| Blnk | B-cell linker | 342.03 | 1433.87 | 4.19 | 0.000092 |
| Prcp | prolylcarboxypeptidase (angiotensinase C) | 25.68 | 84.11 | 3.28 | 0.008781 |
|  | Rn.23518.2 | 1129.20 | 517.22 | -2.18 | 0.007769 |
| Zmat4 | zinc finger, matrin type 4 | 127.44 | 62.14 | -2.05 | 0.001216 |
|  | Rn.74834.1 | 90.81 | 176.18 | 1.94 | 0.000120 |
| Gpr34 | G protein-coupled receptor 34 | 424.02 | 1052.47 | 2.48 | 0.000160 |
| Hells | helicase, lymphoid specific | 34.96 | 73.82 | 2.11 | 0.006199 |
| Pdzrn4 | PDZ domain containing RING finger 4 | 68.32 | 25.78 | -2.65 | 0.002772 |
| Steap1 | six transmembrane epithelial antigen of the prostate 1 | 23.10 | 97.01 | 4.20 | 0.004441 |
| Bcl2l2 | Bcl2-like 2 | 870.99 | 527.58 | -1.65 | 0.000149 |
| LOC100360933 | cDNA sequence BC048679-like | 28.38 | 11.59 | -2.45 | 0.030425 |
|  | Rn.45654.1 | 60.87 | 21.70 | -2.80 | 0.001477 |
|  | Rn.11836.1 | 14.44 | 48.42 | 3.35 | 0.033195 |
|  | Rn.19575.1 | 888.20 | 471.26 | -1.88 | 0.000041 |
| Lppr5 | lipid phosphate phosphatase-related protein type 5 | 77.56 | 28.86 | -2.69 | 0.048715 |
|  | Rn.52740.1 | 677.85 | 393.28 | -1.72 | 0.000579 |
|  | Rn.34553.1 | 428.33 | 153.12 | -2.80 | 0.000057 |
|  | Rn.18695.1 | 405.42 | 172.79 | -2.35 | 0.000150 |
|  | Rn.76607.1 | 330.18 | 189.02 | -1.75 | 0.000342 |
| Fa2h | fatty acid 2-hydroxylase | 2133.04 | 798.97 | -2.67 | 0.000824 |
| Ttc3 | tetratricopeptide repeat domain 3 | 534.68 | 194.74 | -2.75 | 0.010681 |
| LOC689399 | hypothetical protein LOC689399 | 10.28 | 142.54 | 13.87 | 0.004450 |
|  | Rn.48118.1 | 637.97 | 282.57 | -2.26 | 0.000837 |
| Nkain3 | Na+/K+ transporting ATPase interacting 3 | 33.60 | 13.78 | -2.44 | 0.033929 |
| Rbm43 | RNA binding motif protein 43 | 31.34 | 84.54 | 2.70 | 0.002618 |
|  | Rn.27858.1 | 131.21 | 51.67 | -2.54 | 0.005392 |
|  | Rn.13087.1 | 53.04 | 21.49 | -2.47 | 0.002675 |
| Mlf1ip | myeloid leukemia factor 1 interacting protein | 31.10 | 70.88 | 2.28 | 0.009665 |
| Nkiras1 | NFKB inhibitor interacting Ras-like 1 | 350.48 | 141.44 | -2.48 | 0.000675 |
| Rrm2 | ribonucleotide reductase M2 | 4.58 | 111.72 | 24.37 | 0.013762 |
| Cd180 | CD180 molecule | 117.39 | 210.00 | 1.79 | 0.001674 |
|  | Rn.70382.1 | 152.48 | 70.27 | -2.17 | 0.001076 |
| Runx1t1 | runt-related transcription factor 1; translocated to, 1 (cyclin D-related) | 121.56 | 51.03 | -2.38 | 0.014848 |
| RGD1305110 | similar to KIAA1841 protein | 219.31 | 92.17 | -2.38 | 0.009319 |
| Fam13a1 | family with sequence similarity 13, member A1 | 92.55 | 34.67 | -2.67 | 0.013980 |
| Ccr6 | chemokine (C-C motif) receptor 6 | 15.12 | 40.65 | 2.69 | 0.027662 |
|  | Rn.56493.1 | 1187.37 | 647.02 | -1.84 | 0.000221 |
|  | Rn.41691.1 | 23.83 | 185.06 | 7.77 | 0.002347 |
| Stfa2l3 | stefin A2-like 3 | 13.07 | 156.52 | 11.98 | 0.036969 |
|  | Rn.45407.1 | 303.72 | 91.55 | -3.32 | 0.000128 |
|  | Rn.48356.1 | 238.31 | 83.27 | -2.86 | 0.003459 |
|  | Rn.44086.1 | 102.47 | 193.59 | 1.89 | 0.000594 |
|  | Rn.42032.1 | 24.47 | 3.65 | -6.71 | 0.040806 |
|  | Rn.51315.1 | 195.08 | 366.38 | 1.88 | 0.013923 |
| Cr1l | complement component (3b/4b) receptor 1-like | 24.74 | 55.27 | 2.23 | 0.004637 |
|  | Rn.20530.2 | 406.32 | 207.51 | -1.96 | 0.000780 |
| Rhoh | ras homolog gene family, member H | 37.85 | 93.51 | 2.47 | 0.000554 |
|  | Rn.7033.1 | 247.85 | 142.45 | -1.74 | 0.000468 |
|  | Rn.65520.2 | 37.20 | 177.54 | 4.77 | 0.022947 |
|  | Rn.25499.1 | 19.59 | 46.25 | 2.36 | 0.011073 |
| LOC683008 | similar to spermine synthase /// spermine synthase | 404.59 | 134.84 | -3.00 | 0.003744 |
| Spock3 | sparc/osteonectin, cwcv and kazal-like domains proteoglycan (testican) 3 | 1139.50 | 448.90 | -2.54 | 0.000732 |
|  | Rn.49196.2 | 94.64 | 44.12 | -2.15 | 0.001681 |
|  | Rn.33045.2 | 689.25 | 323.21 | -2.13 | 0.000303 |
| Hoxd1 | homeo box D1 | 79.68 | 34.28 | -2.32 | 0.000530 |
| Bex1 | brain expressed gene 1 /// brain expressed X-linked 2 | 601.00 | 331.91 | -1.81 | 0.000420 |
| Chst8 | carbohydrate (N-acetylgalactosamine 4-0) sulfotransferase 8 | 104.12 | 54.07 | -1.93 | 0.008613 |
| LOC100363805 | Temporarily Assigned Gene name family member (tag-241)-like | 169.24 | 45.91 | -3.69 | 0.014759 |
|  | Rn.48621.1 | 1418.35 | 621.39 | -2.28 | 0.000053 |
|  | Rn.29946.1 | 542.70 | 245.02 | -2.21 | 0.011660 |
| Elovl6 | ELOVL family member 6, elongation of long chain fatty acids (yeast) | 885.57 | 373.17 | -2.37 | 0.002485 |
|  | Rn.28104.1 | 226.75 | 99.55 | -2.28 | 0.004652 |
|  | Rn.41393.1 | 112.77 | 56.42 | -2.00 | 0.005421 |
|  | Rn.46692.1 | 265.81 | 89.61 | -2.97 | 0.000057 |
| Ano3 | anoctamin 3 | 224.11 | 70.86 | -3.16 | 0.043591 |
| Arhgap11a | Rho GTPase activating protein 11A | 264.92 | 574.78 | 2.17 | 0.001973 |
| Ttll7 | tubulin tyrosine ligase-like family, member 7 | 1512.70 | 575.40 | -2.63 | 0.005203 |
|  | Rn.50151.1 | 216.98 | 54.49 | -3.98 | 0.001600 |
|  | Rn.45902.1 | 382.24 | 153.27 | -2.49 | 0.004917 |
|  | Rn.32345.1 | 529.02 | 284.48 | -1.86 | 0.000311 |
|  | Rn.44094.1 | 1621.03 | 626.22 | -2.59 | 0.005087 |
|  | Rn.26663.1 | 1186.68 | 625.92 | -1.90 | 0.000117 |
|  | Rn.55394.1 | 48.00 | 11.05 | -4.34 | 0.004764 |
|  | Rn.47038.1 | 1243.90 | 453.72 | -2.74 | 0.000003 |
|  | Rn.41733.1 | 887.06 | 475.75 | -1.86 | 0.000095 |
|  | Rn.49676.1 | 823.37 | 327.25 | -2.52 | 0.000331 |
|  | Rn.40510.1 | 103.11 | 208.78 | 2.02 | 0.007709 |
|  | Rn.35619.1 | 50.57 | 262.35 | 5.19 | 0.030130 |
|  | Rn.48626.1 | 669.60 | 253.55 | -2.64 | 0.000004 |
|  | Rn.62317.1 | 156.93 | 40.19 | -3.90 | 0.000555 |
| Spire2 | spire homolog 2 (Drosophila) | 88.95 | 41.02 | -2.17 | 0.001283 |
| LOC691221 | similar to CG1998-PA | 61.20 | 163.64 | 2.67 | 0.013849 |
| Xk | X-linked Kx blood group (McLeod syndrome) homolog | 140.83 | 55.05 | -2.56 | 0.001422 |
|  | Rn.57762.1 | 724.10 | 247.30 | -2.93 | 0.000917 |
| Nkiras1 | NFKB inhibitor interacting Ras-like 1 | 855.59 | 290.18 | -2.95 | 0.000014 |
|  | Rn.54009.1 | 161.83 | 91.76 | -1.76 | 0.001097 |
|  | Rn.51154.1 | 725.70 | 391.41 | -1.85 | 0.000855 |
|  | Rn.32352.1 | 114.72 | 18.60 | -6.17 | 0.000210 |
|  | Rn.81605.1 | 102.23 | 46.82 | -2.18 | 0.001071 |
| LOC687856 | similar to Myeloid cell surface antigen CD33 precursor (Siglec-3) | 167.93 | 1196.99 | 7.13 | 0.001428 |
| Oxa1l | oxidase assembly 1-like | 7.76 | 25.32 | 3.26 | 0.003375 |
| Fgd2 | FYVE, RhoGEF and PH domain containing 2 | 47.02 | 357.66 | 7.61 | 0.000224 |
|  | Rn.50850.1 | 141.10 | 50.78 | -2.78 | 0.005139 |
|  | Rn.61595.1 | 28.43 | 65.30 | 2.30 | 0.000388 |
|  | Rn.64294.1 | 352.87 | 163.40 | -2.16 | 0.031779 |
| Fam46a | family with sequence similarity 46, member A | 39.33 | 97.41 | 2.48 | 0.043619 |
| RGD1564677 | Similar to transcription factor ONECUT2 | 77.86 | 37.34 | -2.09 | 0.027550 |
| LOC100363228 | hypothetical LOC100363228 | 46.34 | 105.60 | 2.28 | 0.000406 |
|  | Rn.54795.1 | 31.29 | 69.01 | 2.21 | 0.006594 |
|  | Rn.41439.1 | 39.66 | 12.47 | -3.18 | 0.009382 |
|  | Rn.42963.1 | 134.08 | 73.52 | -1.82 | 0.000518 |
| Arhgap42 | Rho GTPase activating protein 42 | 63.06 | 179.25 | 2.84 | 0.001768 |
|  | Rn.55366.1 | 423.15 | 183.50 | -2.31 | 0.003657 |
|  | Rn.54356.1 | 57.98 | 116.28 | 2.01 | 0.004475 |
| Sp110 | SP110 nuclear body protein | 13.35 | 47.91 | 3.59 | 0.035885 |
|  | Rn.48950.1 | 700.40 | 346.40 | -2.02 | 0.005791 |
|  | Rn.57513.1 | 153.17 | 30.37 | -5.04 | 0.000046 |
|  | Rn.44677.1 | 158.07 | 42.17 | -3.75 | 0.001670 |
|  | Rn.71359.1 | 1553.63 | 297.20 | -5.23 | 0.003486 |
| Rtp3 | receptor (chemosensory) transporter protein 3 | 10.73 | 34.16 | 3.18 | 0.014967 |
|  | Rn.37257.1 | 76.43 | 37.45 | -2.04 | 0.002711 |
|  | Rn.38545.1 | 82.95 | 39.64 | -2.09 | 0.000975 |
|  | Rn.48263.1 | 533.01 | 195.12 | -2.73 | 0.000227 |
|  | Rn.46297.1 | 239.83 | 114.21 | -2.10 | 0.000109 |
|  | Rn.28423.1 | 6.92 | 21.75 | 3.14 | 0.004418 |
| Fam46a | family with sequence similarity 46, member A | 177.66 | 670.82 | 3.78 | 0.002937 |
|  | Rn.48091.1 | 587.94 | 202.98 | -2.90 | 0.000007 |
| Hapln2 | Hyaluronan and proteoglycan link protein 2 | 97.28 | 46.29 | -2.10 | 0.007609 |
| Slc31a2 | solute carrier family 31 (copper transporters), member 2 | 28.88 | 106.99 | 3.70 | 0.000231 |
|  | Rn.58970.1 | 181.28 | 40.03 | -4.53 | 0.019291 |
| Grsf1 | G-rich RNA sequence binding factor 1 | 26.06 | 8.74 | -2.98 | 0.008218 |
|  | Rn.49708.1 | 365.30 | 150.78 | -2.42 | 0.000285 |
|  | Rn.49209.1 | 247.74 | 137.40 | -1.80 | 0.004936 |
| Fam120b | Family with sequence similarity 120B | 272.21 | 155.05 | -1.76 | 0.001726 |
|  | Rn.66861.1 | 241.50 | 86.98 | -2.78 | 0.011037 |
| Clec4a1 | C-type lectin domain family 4, member a1 | 79.93 | 304.46 | 3.81 | 0.000142 |
|  | Rn.63277.1 | 131.35 | 53.96 | -2.43 | 0.014562 |
|  | Rn.74132.1 | 219.67 | 87.38 | -2.51 | 0.000368 |
| Ndufa7 | NADH dehydrogenase (ubiquinone) 1 alpha subcomplex, 7 (B14.5a) | 317.71 | 166.45 | -1.91 | 0.001617 |
| Pds5b | PDS5, regulator of cohesion maintenance, homolog B (S. cerevisiae) | 89.05 | 28.81 | -3.09 | 0.023242 |
| Usp29 | ubiquitin specific peptidase 29 | 378.57 | 95.22 | -3.98 | 0.008365 |
| Runx1t1 | runt-related transcription factor 1; translocated to, 1 (cyclin D-related) | 163.81 | 71.77 | -2.28 | 0.005170 |
| Kcnt1 | potassium channel, subfamily T, member 1 | 315.11 | 145.07 | -2.17 | 0.000045 |
| Cd8a | CD8a molecule | 14.20 | 94.16 | 6.63 | 0.001398 |
|  | Rn.61254.1 | 373.08 | 135.92 | -2.74 | 0.001804 |
| Fcrls | Fc receptor-like S, scavenger receptor | 74.45 | 4306.83 | 57.85 | 0.000117 |
|  | Rn.66874.1 | 234.40 | 99.57 | -2.35 | 0.000969 |
| Ajap1 | adherens junction associated protein 1 | 238.47 | 90.07 | -2.65 | 0.001836 |
|  | Rn.52695.1 | 74.44 | 143.42 | 1.93 | 0.009178 |
|  | Rn.46395.1 | 122.74 | 44.41 | -2.76 | 0.001758 |
| Clec12a | C-type lectin domain family 12, member A | 38.92 | 879.31 | 22.59 | 0.001627 |
|  | Rn.62977.1 | 56.96 | 22.51 | -2.53 | 0.002135 |
|  | Rn.76239.1 | 194.28 | 107.56 | -1.81 | 0.002208 |
| Edem1 | ER degradation enhancer, mannosidase alpha-like 1 | 47.12 | 116.64 | 2.48 | 0.000763 |
| Snhg11 | small nucleolar RNA host gene 11 | 2052.73 | 1113.50 | -1.84 | 0.000265 |
|  | Rn.59670.1 | 214.26 | 55.74 | -3.84 | 0.001498 |
| Gns | Glucosamine (N-acetyl)-6-sulfatase | 132.90 | 533.21 | 4.01 | 0.006479 |
|  | Rn.45956.1 | 168.16 | 76.07 | -2.21 | 0.000980 |
|  | Rn.32473.1 | 142.72 | 70.61 | -2.02 | 0.003830 |
| LOC685157 | similar to paired immunoglobin-like type 2 receptor beta /// similar to paired immunoglobin-like type 2 receptor beta | 58.83 | 159.29 | 2.71 | 0.003010 |
|  | Rn.37909.1 | 2114.53 | 932.71 | -2.27 | 0.007814 |
| Anxa3 | Annexin A3 | 217.25 | 825.46 | 3.80 | 0.000244 |
|  | Rn.34392.1 | 4130.63 | 2269.52 | -1.82 | 0.001151 |
|  | Rn.50256.1 | 107.78 | 57.86 | -1.86 | 0.001783 |
| Chrna7 | Cholinergic receptor, nicotinic, alpha 7 | 538.60 | 237.76 | -2.27 | 0.000059 |
|  | Rn.65797.1 | 237.77 | 124.26 | -1.91 | 0.000357 |
|  | Rn.31927.1 | 103.46 | 310.02 | 3.00 | 0.007813 |
| Ermn | ermin, ERM-like protein | 381.32 | 144.74 | -2.63 | 0.028442 |
| Casc5 | cancer susceptibility candidate 5 | 16.16 | 157.10 | 9.72 | 0.002957 |
|  | Rn.48058.1 | 122.46 | 41.05 | -2.98 | 0.003276 |
|  | Rn.62933.1 | 43.22 | 13.60 | -3.18 | 0.004566 |
|  | Rn.74155.1 | 535.74 | 256.90 | -2.09 | 0.002768 |
|  | Rn.45549.1 | 262.03 | 94.51 | -2.77 | 0.000259 |
| MGC95152 | similar to B230212L03Rik protein | 209.72 | 95.10 | -2.21 | 0.001204 |
| Fam98a | family with sequence similarity 98, member A | 264.63 | 103.87 | -2.55 | 0.010388 |
|  | Rn.51103.1 | 69.50 | 266.24 | 3.83 | 0.013933 |
| LOC100362992 | NKAIN2 transcript-like | 175.98 | 68.60 | -2.57 | 0.000129 |
|  | Rn.50868.1 | 93.83 | 45.25 | -2.07 | 0.010687 |
|  | Rn.60828.1 | 116.90 | 60.81 | -1.92 | 0.002239 |
|  | Rn.54178.1 | 91.42 | 47.78 | -1.91 | 0.018971 |
|  | Rn.38077.1 | 270.30 | 120.31 | -2.25 | 0.000215 |
|  | Rn.45828.1 | 53.83 | 16.33 | -3.30 | 0.005495 |
|  | Rn.48846.1 | 198.02 | 99.19 | -2.00 | 0.003648 |
|  | Rn.46193.1 | 422.76 | 196.38 | -2.15 | 0.000955 |
| Erp44 | endoplasmic reticulum protein 44 | 208.67 | 379.63 | 1.82 | 0.004817 |
| Fbxo9 | f-box protein 9 | 1274.96 | 502.78 | -2.54 | 0.003550 |
|  | Rn.27810.1 | 43.63 | 19.31 | -2.26 | 0.040849 |
|  | Rn.45538.1 | 3360.22 | 962.88 | -3.49 | 0.010407 |
|  | Rn.46336.1 | 494.78 | 190.89 | -2.59 | 0.000011 |
| Cd276 | Cd276 molecule | 18.59 | 35.50 | 1.91 | 0.002930 |
|  | Rn.64211.1 | 37.79 | 16.12 | -2.34 | 0.009734 |
|  | Rn.41780.1 | 254.70 | 113.11 | -2.25 | 0.001922 |
|  | Rn.35380.1 | 17.71 | 51.31 | 2.90 | 0.003105 |
| LOC691653 | Hypothetical protein LOC691653 | 42.82 | 17.59 | -2.43 | 0.015486 |
|  | Rn.44118.1 | 210.86 | 62.02 | -3.40 | 0.000074 |
| Odz2 | Odz, odd Oz/ten-m homolog 2 (Drosophila) | 408.61 | 158.70 | -2.57 | 0.000140 |
|  | Rn.64242.1 | 56.11 | 22.24 | -2.52 | 0.003521 |
| Clca3 | chloride channel calcium activated 3 | 108.40 | 41.37 | -2.62 | 0.005437 |
|  | Rn.56084.1 | 44.53 | 17.24 | -2.58 | 0.014606 |
|  | Rn.61562.1 | 114.11 | 53.36 | -2.14 | 0.000963 |
| Usp35 | ubiquitin specific peptidase 35 | 309.37 | 157.54 | -1.96 | 0.001426 |
| RGD1310778 | similar to Putative protein C21orf45 | 17.22 | 86.56 | 5.03 | 0.004235 |
| Rpa1 | Replication protein A1 | 68.96 | 32.67 | -2.11 | 0.002865 |
| LOC688276 | similar to epidermodysplasia verruciformis 2 | 8.35 | 29.55 | 3.54 | 0.009154 |
| Slit2 | slit homolog 2 (Drosophila) | 540.46 | 234.20 | -2.31 | 0.000517 |
| Rimbp2 | RIMS binding protein 2 | 451.77 | 263.43 | -1.71 | 0.001225 |
|  | Rn.60179.1 | 59.97 | 14.19 | -4.23 | 0.004754 |
|  | Rn.61362.1 | 143.72 | 69.46 | -2.07 | 0.000734 |
| Gpr61 | G protein-coupled receptor 61 | 52.56 | 25.89 | -2.03 | 0.012050 |
| Shank1 | SH3 and multiple ankyrin repeat domains 1 | 875.79 | 417.11 | -2.10 | 0.000311 |
|  | Rn.66180.1 | 73.47 | 34.79 | -2.11 | 0.007182 |
|  | Rn.43420.1 | 6.32 | 28.26 | 4.47 | 0.003614 |
|  | Rn.46540.1 | 172.15 | 65.75 | -2.62 | 0.005851 |
| Nkiras1 | NFKB inhibitor interacting Ras-like 1 | 130.93 | 32.76 | -4.00 | 0.003430 |
|  | Rn.74176.1 | 45.13 | 20.33 | -2.22 | 0.006672 |
|  | Rn.27697.1 | 503.55 | 237.10 | -2.12 | 0.002598 |
|  | Rn.46464.1 | 47.13 | 11.43 | -4.13 | 0.001306 |
|  | Rn.79313.1 | 369.43 | 185.12 | -2.00 | 0.000321 |
|  | Rn.38706.1 | 407.74 | 117.42 | -3.47 | 0.010169 |
| sep-11 | septin 11 | 107.90 | 232.95 | 2.16 | 0.006474 |
|  | Rn.34809.1 | 60.04 | 157.82 | 2.63 | 0.002876 |
| Rasal3 | RAS protein activator like 3 | 47.78 | 245.16 | 5.13 | 0.000006 |
| Galnt14 | UDP-N-acetyl-alpha-D-galactosamine:polypeptide N-acetylgalactosaminyltransferase 14 (GalNAc-T14) | 71.69 | 27.21 | -2.63 | 0.002731 |
| RGD1566036 | similar to RIKEN cDNA 2310008H04 | 148.58 | 263.03 | 1.77 | 0.001945 |
|  | Rn.59532.1 | 160.76 | 61.30 | -2.62 | 0.001812 |
|  | Rn.53091.1 | 49.81 | 24.58 | -2.03 | 0.027532 |
|  | Rn.56899.1 | 19.23 | 5.92 | -3.25 | 0.015568 |
|  | Rn.26625.1 | 418.26 | 162.32 | -2.58 | 0.001765 |
|  | Rn.46729.1 | 41.58 | 14.83 | -2.80 | 0.012243 |
|  | Rn.53637.1 | 89.71 | 42.01 | -2.14 | 0.005503 |
| LOC681383 | similar to Protein C10orf11 homolog | 164.14 | 307.83 | 1.88 | 0.000374 |
|  | Rn.50757.1 | 95.71 | 38.17 | -2.51 | 0.000416 |
|  | Rn.57771.1 | 99.91 | 35.35 | -2.83 | 0.002816 |
| RGD1565926 | RGD1565926 | 8.10 | 25.01 | 3.09 | 0.012736 |
| LOC682033 | similar to Protein phosphatase 2A, 59 kDa regulatory subunit B (PP2A PR59) (PP2A B-PR59) | 33.75 | 70.29 | 2.08 | 0.013047 |
|  | Rn.61219.1 | 44.21 | 15.96 | -2.77 | 0.003234 |
| MGC116202 | hypothetical protein LOC688736 | 246.77 | 104.48 | -2.36 | 0.013452 |
|  | Rn.27270.1 | 747.01 | 313.06 | -2.39 | 0.000211 |
| Aldh3b1 | aldehyde dehydrogenase 3 family, member B1 | 84.72 | 230.44 | 2.72 | 0.000217 |
| LOC687758 | similar to phosphatidylinositol-4-phosphate 5-kinase-like 1 | 864.54 | 333.76 | -2.59 | 0.000126 |
|  | Rn.46840.1 | 46.59 | 8.07 | -5.78 | 0.000847 |
|  | Rn.58618.1 | 181.13 | 92.16 | -1.97 | 0.000472 |
|  | Rn.61292.1 | 89.58 | 41.35 | -2.17 | 0.002978 |
|  | Rn.74937.1 | 169.61 | 90.60 | -1.87 | 0.003326 |
| Enah | enabled homolog (Drosophila) | 238.41 | 83.78 | -2.85 | 0.006363 |
| Vps4a | vacuolar protein sorting 4 homolog A (S. cerevisiae) | 84.26 | 41.50 | -2.03 | 0.002670 |
|  | Rn.53310.1 | 355.18 | 97.39 | -3.65 | 0.037210 |
|  | Rn.62326.1 | 334.36 | 170.26 | -1.96 | 0.000932 |
|  | Rn.58018.1 | 58.67 | 18.95 | -3.10 | 0.016724 |
|  | Rn.61498.1 | 36.42 | 82.25 | 2.26 | 0.016245 |
|  | Rn.38878.1 | 59.17 | 19.19 | -3.08 | 0.006060 |
|  | Rn.46884.1 | 33.04 | 16.27 | -2.03 | 0.007735 |
|  | Rn.51526.1 | 45.95 | 23.07 | -1.99 | 0.014392 |
| LOC290156 | hypothetical LOC290156 /// hypothetical LOC682708 /// similar to RIKEN cDNA A430107P09 gene | 270.73 | 90.26 | -3.00 | 0.006437 |
|  | Rn.51548.1 | 60.87 | 12.73 | -4.78 | 0.001893 |
|  | Rn.62201.1 | 57.16 | 20.98 | -2.72 | 0.014991 |
|  | Rn.53171.1 | 200.43 | 55.76 | -3.59 | 0.002509 |
| Gria4 | glutamate receptor, ionotropic, AMPA4 | 574.86 | 214.49 | -2.68 | 0.000513 |
|  | Rn.63023.1 | 107.36 | 42.71 | -2.51 | 0.002476 |
|  | Rn.62711.1 | 86.61 | 36.71 | -2.36 | 0.011229 |
|  | Rn.48489.1 | 79.69 | 159.82 | 2.01 | 0.000611 |
|  | Rn.57563.1 | 27.39 | 11.09 | -2.47 | 0.012908 |
|  | Rn.59896.1 | 42.17 | 11.80 | -3.57 | 0.041495 |
|  | Rn.61626.1 | 80.19 | 26.66 | -3.01 | 0.004345 |
|  | Rn.45624.1 | 142.45 | 72.71 | -1.96 | 0.008359 |
|  | Rn.64208.1 | 55.84 | 26.40 | -2.12 | 0.008378 |
|  | Rn.50363.1 | 155.08 | 67.34 | -2.30 | 0.000768 |
|  | Rn.49201.1 | 171.93 | 81.90 | -2.10 | 0.002545 |
| Fstl4 | follistatin-like 4 | 103.01 | 58.46 | -1.76 | 0.002660 |
|  | Rn.60594.1 | 595.01 | 117.47 | -5.07 | 0.027516 |
|  | Rn.60525.1 | 526.77 | 181.96 | -2.89 | 0.011641 |
|  | Rn.60547.1 | 41.68 | 17.83 | -2.34 | 0.012964 |
|  | Rn.58180.1 | 51.27 | 20.92 | -2.45 | 0.002699 |
|  | Rn.58253.1 | 80.27 | 27.31 | -2.94 | 0.014793 |
|  | Rn.46760.1 | 244.70 | 74.01 | -3.31 | 0.000008 |
|  | Rn.45416.1 | 18.23 | 6.46 | -2.82 | 0.042597 |
|  | Rn.51499.1 | 10.63 | 30.86 | 2.90 | 0.003588 |
|  | Rn.46664.1 | 32.33 | 14.27 | -2.27 | 0.013350 |
| Zfp207 | Zinc finger protein 207 | 5.90 | 21.82 | 3.70 | 0.010205 |
|  | Rn.61905.1 | 54.64 | 106.14 | 1.94 | 0.008122 |
|  | Rn.41379.1 | 8.25 | 21.33 | 2.59 | 0.017095 |
|  | Rn.48246.1 | 29.46 | 74.06 | 2.51 | 0.027605 |
|  | Rn.31723.1 | 430.52 | 196.70 | -2.19 | 0.000058 |
| Mettl6 | methyltransferase like 6 | 179.16 | 326.99 | 1.83 | 0.000516 |
|  | Rn.46527.1 | 128.49 | 49.24 | -2.61 | 0.021816 |
|  | Rn.55613.1 | 18.81 | 57.74 | 3.07 | 0.000566 |
|  | Rn.50084.1 | 217.81 | 104.82 | -2.08 | 0.000181 |
| Fas | Fas (TNF receptor superfamily, member 6) | 12.55 | 31.83 | 2.54 | 0.004891 |
| Tmem35 | transmembrane protein 35 | 1559.51 | 788.03 | -1.98 | 0.000026 |
| Dclk3 | doublecortin-like kinase 3 | 240.97 | 103.81 | -2.32 | 0.000602 |
| RGD1306880 | similar to hypothetical protein MGC47816 | 343.30 | 137.11 | -2.50 | 0.000695 |
|  | Rn.29216.1 | 17.94 | 57.21 | 3.19 | 0.002210 |
| LOC684568 | similar to solute carrier family 17 (sodium phosphate), member 4 | 53.51 | 28.70 | -1.86 | 0.001738 |
|  | Rn.53297.1 | 29.58 | 100.25 | 3.39 | 0.010714 |
|  | Rn.42112.1 | 125.36 | 243.79 | 1.94 | 0.000450 |
| Igtp | interferon gamma induced GTPase | 106.25 | 383.47 | 3.61 | 0.000082 |
| Pbk | PDZ binding kinase | 82.17 | 287.50 | 3.50 | 0.014143 |
|  | Rn.29191.1 | 17.95 | 37.52 | 2.09 | 0.006978 |
|  | Rn.41129.1 | 313.71 | 145.80 | -2.15 | 0.003764 |
| LOC689663 | Hypothetical protein LOC689663 | 216.86 | 86.48 | -2.51 | 0.007739 |
| Map7d2 | MAP7 domain containing 2 | 1137.04 | 394.62 | -2.88 | 0.000873 |
|  | Rn.27457.1 | 469.44 | 222.09 | -2.11 | 0.000677 |
|  | Rn.72425.1 | 264.92 | 127.47 | -2.08 | 0.000334 |
|  | Rn.69561.1 | 28.49 | 12.90 | -2.21 | 0.015298 |
|  | Rn.61492.1 | 442.02 | 241.03 | -1.83 | 0.000145 |
| LOC497978 | similar to diacylglycerol kinase epsilon | 554.23 | 221.07 | -2.51 | 0.001585 |
|  | Rn.53249.1 | 127.73 | 55.27 | -2.31 | 0.005183 |
|  | Rn.79807.1 | 32.89 | 14.89 | -2.21 | 0.013877 |
|  | Rn.47607.1 | 64.33 | 31.46 | -2.04 | 0.006853 |
|  | Rn.35345.1 | 116.41 | 45.70 | -2.55 | 0.025883 |
|  | Rn.72394.1 | 149.47 | 44.86 | -3.33 | 0.001030 |
|  | Rn.44862.1 | 143.66 | 78.87 | -1.82 | 0.003388 |
| Hmha1 | histocompatibility (minor) HA-1 | 74.43 | 266.20 | 3.58 | 0.000043 |
|  | Rn.45224.1 | 755.83 | 258.27 | -2.93 | 0.019572 |
|  | Rn.28108.1 | 155.03 | 71.34 | -2.17 | 0.001926 |
|  | Rn.62842.1 | 21.08 | 8.75 | -2.41 | 0.036348 |
| Trim2 | tripartite motif-containing 2 | 382.52 | 207.25 | -1.85 | 0.002827 |
|  | Rn.59797.1 | 191.61 | 106.63 | -1.80 | 0.001083 |
| Vezt | vezatin, adherens junctions transmembrane protein | 30.15 | 60.79 | 2.02 | 0.002241 |
|  | Rn.39365.1 | 22.57 | 171.11 | 7.58 | 0.004913 |
| Traf4af1 | TRAF4 associated factor 1 | 3.53 | 26.71 | 7.57 | 0.010165 |
|  | Rn.45049.1 | 16.80 | 54.65 | 3.25 | 0.003463 |
|  | Rn.52745.1 | 237.48 | 67.45 | -3.52 | 0.000203 |
| Gabra2 | gamma-aminobutyric acid (GABA-A) receptor, subunit alpha 2 | 356.54 | 134.23 | -2.66 | 0.005749 |
|  | Rn.43415.1 | 206.69 | 106.91 | -1.93 | 0.000121 |
| Slitrk1 | SLIT and NTRK-like family, member 1 | 562.99 | 235.79 | -2.39 | 0.004138 |
|  | Rn.28663.1 | 426.22 | 178.37 | -2.39 | 0.000437 |
| Frs2 | fibroblast growth factor receptor substrate 2 | 5.45 | 20.30 | 3.72 | 0.046428 |
|  | Rn.50669.1 | 112.55 | 62.50 | -1.80 | 0.002886 |
|  | Rn.50408.1 | 440.75 | 208.27 | -2.12 | 0.000245 |
|  | Rn.51268.1 | 88.10 | 304.69 | 3.46 | 0.000079 |
|  | Rn.62117.1 | 40.65 | 12.91 | -3.15 | 0.017780 |
|  | Rn.76528.1 | 296.45 | 123.42 | -2.40 | 0.014585 |
| Eif4a2 | Eukaryotic translation initiation factor 4A2 | 96.69 | 169.61 | 1.75 | 0.000432 |
|  | Rn.59778.1 | 673.63 | 279.47 | -2.41 | 0.008380 |
|  | Rn.61051.1 | 37.24 | 91.50 | 2.46 | 0.003410 |
| LOC363060 | similar to RIKEN cDNA 1600029D21 | 8.81 | 30.46 | 3.46 | 0.004098 |
|  | Rn.51484.1 | 175.80 | 102.32 | -1.72 | 0.001775 |
|  | Rn.27799.1 | 304.48 | 589.32 | 1.94 | 0.000411 |
|  | Rn.41755.1 | 42.95 | 101.37 | 2.36 | 0.000533 |
|  | Rn.60131.1 | 59.40 | 21.65 | -2.74 | 0.009640 |
| Avil | Advillin | 188.77 | 76.13 | -2.48 | 0.001282 |
|  | Rn.62945.1 | 131.10 | 44.32 | -2.96 | 0.000879 |
|  | Rn.55535.1 | 141.04 | 1304.89 | 9.25 | 0.000028 |
|  | Rn.34472.1 | 218.54 | 125.49 | -1.74 | 0.001702 |
|  | Rn.46497.1 | 17.88 | 95.31 | 5.33 | 0.000184 |
|  | Rn.51888.1 | 143.74 | 56.49 | -2.54 | 0.007886 |
|  | Rn.34551.1 | 85.92 | 38.04 | -2.26 | 0.002070 |
|  | Rn.27629.1 | 392.69 | 172.66 | -2.27 | 0.000138 |
|  | Rn.29109.1 | 77.41 | 136.07 | 1.76 | 0.003188 |
| RGD1564053 | similar to hypothetical protein | 89.27 | 32.69 | -2.73 | 0.000303 |
|  | Rn.40767.1 | 124.50 | 70.68 | -1.76 | 0.002899 |
|  | Rn.79289.1 | 58.61 | 124.48 | 2.12 | 0.001152 |
|  | Rn.46147.1 | 169.07 | 93.96 | -1.80 | 0.000656 |
|  | Rn.46323.1 | 207.41 | 107.73 | -1.93 | 0.000770 |
|  | Rn.64231.1 | 292.98 | 599.67 | 2.05 | 0.000047 |
|  | Rn.62271.1 | 35.96 | 15.02 | -2.39 | 0.036273 |
|  | Rn.54262.1 | 19.63 | 66.64 | 3.39 | 0.013592 |
|  | Rn.15124.1 | 33.79 | 219.17 | 6.49 | 0.000029 |
|  | Rn.61467.1 | 773.93 | 241.07 | -3.21 | 0.007281 |
|  | Rn.60383.1 | 68.09 | 29.37 | -2.32 | 0.004459 |
|  | Rn.59709.1 | 182.37 | 72.66 | -2.51 | 0.008401 |
|  | Rn.62194.1 | 82.76 | 33.30 | -2.48 | 0.008525 |
| Sobpl | sine oculis-binding protein homolog-like (Drosophila) | 135.06 | 58.52 | -2.31 | 0.007353 |
|  | Rn.46466.1 | 169.89 | 87.61 | -1.94 | 0.001528 |
|  | Rn.47810.1 | 12.04 | 40.95 | 3.40 | 0.002433 |
|  | Rn.58221.1 | 44.87 | 15.44 | -2.91 | 0.013514 |
|  | Rn.62531.1 | 68.36 | 29.78 | -2.30 | 0.011114 |
|  | Rn.61331.1 | 206.67 | 77.71 | -2.66 | 0.001439 |
|  | Rn.62827.1 | 564.12 | 233.95 | -2.41 | 0.001474 |
|  | Rn.56632.1 | 55.94 | 17.04 | -3.28 | 0.009309 |
| LOC100362909 | rCG30099-like | 4.53 | 15.43 | 3.41 | 0.016368 |
|  | Rn.47916.1 | 35.08 | 10.83 | -3.24 | 0.002288 |
| Ryr3 | ryanodine receptor 3 | 298.62 | 112.91 | -2.64 | 0.000092 |
| Lrrc24 | leucine rich repeat containing 24 | 141.81 | 66.09 | -2.15 | 0.000897 |
| Bcl3 | B-cell CLL/lymphoma 3 | 45.24 | 184.96 | 4.09 | 0.000302 |
| Rgs17 | regulator of G-protein signaling 17 | 747.41 | 257.46 | -2.90 | 0.004790 |
|  | Rn.46241.1 | 189.77 | 109.31 | -1.74 | 0.000674 |
| Susd2 | sushi domain containing 2 | 628.73 | 355.85 | -1.77 | 0.000327 |
|  | Rn.41974.1 | 12.56 | 53.33 | 4.25 | 0.001107 |
|  | Rn.64130.1 | 155.11 | 57.97 | -2.68 | 0.000135 |
| Cyfip2 | cytoplasmic FMR1 interacting protein 2 | 972.20 | 405.80 | -2.40 | 0.009321 |
| Ankrd34a | ankyrin repeat domain 34A | 146.84 | 70.06 | -2.10 | 0.006947 |
|  | Rn.47602.1 | 199.00 | 92.72 | -2.15 | 0.008316 |
| Pilra | paired immunoglobin-like type 2 receptor alpha | 5.40 | 63.78 | 11.81 | 0.003926 |
| Ccrl2 | chemokine (C-C motif) receptor-like 2 | 13.16 | 67.54 | 5.13 | 0.000619 |
| Mad2l1 | MAD2 mitotic arrest deficient-like 1 (yeast) | 41.34 | 139.69 | 3.38 | 0.000791 |
|  | Rn.7665.1 | 164.40 | 84.66 | -1.94 | 0.016362 |
| Garnl4 | GTPase activating Rap/RanGAP domain-like 4 | 766.03 | 470.80 | -1.63 | 0.000159 |
| Tubb4 | tubulin, beta 4 | 441.41 | 216.51 | -2.04 | 0.000854 |
|  | Rn.46917.1 | 20.55 | 324.88 | 15.81 | 0.000073 |
|  | Rn.34740.1 | 29.09 | 238.25 | 8.19 | 0.001824 |
| Slitrk3 | SLIT and NTRK-like family, member 3 | 184.89 | 58.65 | -3.15 | 0.003657 |
|  | Rn.28606.1 | 275.53 | 101.11 | -2.73 | 0.000092 |
| Vwc2 | von Willebrand factor C domain containing 2 | 335.05 | 168.77 | -1.99 | 0.001081 |
| Fam167a | family with sequence similarity 167, member A | 22.86 | 78.58 | 3.44 | 0.004722 |
|  | Rn.57442.1 | 92.93 | 47.29 | -1.96 | 0.005317 |
|  | Rn.49945.1 | 199.11 | 66.68 | -2.99 | 0.000248 |
| Fsd1 | fibronectin type III and SPRY domain containing 1 | 364.59 | 168.80 | -2.16 | 0.001005 |
|  | Rn.59189.1 | 108.44 | 40.59 | -2.67 | 0.014575 |
